# Exploring necrosis-associated mitochondrial gene signatures: revealing their role in prognosis and immunotherapy of renal clear cell carcinoma

**DOI:** 10.1007/s10238-024-01426-9

**Published:** 2024-07-18

**Authors:** Zhipeng Wang, Fuchun Zheng, Shiliang Wei, Sheng Li, Situ Xiong, Lei Zhang, Liangwei Wan, Songhui Xu, Jun Deng, Xiaoqiang Liu

**Affiliations:** 1 Department of Urology, The First Affiliated Hospital, Jiangxi Medical College, Nanchang University, Nanchang, 330000 China; 2 Jiangxi Provincial Key Laboratory of Urinary System Diseases, Department of Urology, The First Affiliated Hospital, Jiangxi Medical College, Nanchang University, Nanchang, Jiangxi China

**Keywords:** Mitochondrial dysfunction, Necroptosis, ccRCC

## Abstract

Mitochondrial dysfunction and necrotic apoptosis, pivotal in therapeutic strategies for multiple diseases, lack comprehensive understanding in the context of renal clear cell carcinoma (ccRCC). This study explores their potential as valuable tools for ccRCC prediction, prevention, and personalized medical care. Transcriptomic and clinical datasets were acquired from the Cancer Genome Atlas (TCGA) repository. Mitochondrial and necrosis-associated gene sets were sourced from MitoCarta3.0 and the KEGG Pathway databases, respectively. Six necrosis-related mitochondrial genes (nc-MTGs) with prognostic significance were analyzed and screened, and a prognostic model was constructed. The accuracy of the model was verified using external data (E-MTAB-1980). TISCH was used to explore nc-MTGs at the cellular level. Finally, the expression level of BH3 interacting domain death agonist (BID) in ccRCC cell line was detected by real-time fluorescence quantitative polymerase chain reaction (RT-qPCR), and the effect of BID down-regulation on tumor cell migration was verified by transwell assays and wound-healing experiments. We established and validated a prognostic model for clear cell renal cell carcinoma (ccRCC) utilizing six necrosis-related mitochondrial genes (nc-MTGs), affirming its efficacy in evaluating tumor progression. RT-PCR results showed that BID expression was up-regulated in ccRCC tissues compared with controls and exhibited oncogenic effects. In vitro cell function experiments showed that BID may be an important factor affecting the migration of ccRCC. Our study is the first to elucidate the biological functions and prognostic significance of mitochondrial molecules related to necroptosis, providing a new way to evaluate mitochondrial therapeutics in patients with ccRCC.

**Supplementary Information:**

The online version contains supplementary material available at 10.1007/s10238-024-01426-9.

## Introduction

Renal cell carcinoma (RCC) constitutes roughly 2%-3% of adult malignancies, standing as the second most prevalent urinary system malignancy after bladder cancer, with an escalating incidence [1]. Clear cell renal cell carcinoma (ccRCC), the primary subtype, encompasses 70% to 80% of all RCC instances [2]. Early detection poses challenges, with nearly 30% of patients presenting distant metastases at diagnosis. Due to its complex biology, ccRCC commonly exhibits resistance to radiation and chemotherapy, leading to primary treatment options involving radical resection and immunotherapy [3]. Despite surgical efficacy, 20% to 40% of patients experience local recurrence or distant metastasis postoperatively [4].

In the last decade, research on ccRCC has shifted, with a focus on understanding its complex molecular landscape [1]. Studies reveal the intricate interplay of genetic variations, epigenetic changes, and metabolic disruptions influencing tumor initiation, progression, and treatment resistance [2]. Recent findings underscore the critical roles of mitochondrial malfunction and necroptosis in various cancers [3].

Mitochondria, likened to cellular powerhouses, play a crucial role in energy generation, calcium balance, and apoptosis [4]. Dysregulation of cellular metabolism and redox balance is fundamental in neoplastic transformation, linking mitochondrial dysfunction to disorders like diabetes, cancer, and neurodegeneration [5]. Necroptosis, a regulated self-destructive mechanism marked by cell swelling and inflammation-causing component expulsion, becomes active when apoptosis is blocked [6].

However, the exact role and prognostic impact of necrosis-associated mitochondrial genes (nc-MTGs) in ccRCC remain incompletely understood. To address this knowledge gap, we provided insights into the association between ccRCC and these necrosis-related mitochondrial genes. In addition to the analysis of functionally enriched necrosis-related mitochondrial genes (nc-MTGs), we compared the extent of immune checkpoints, immune cell infiltration, immunotherapy efficacy, and drug sensitivity in different risk groups. Furthermore, we employed a nomogram to visually represent the overall survival of ccRCC patients, aiming to provide insights for the development of novel treatment strategies and personalized approaches. Finally, RT-PCR, transwell assays and wound-healing assays were utilized to assess the impact of BID on the migration of ccRCC.

## Methods

### Data acquisition and processing

We acquired RNA sequencing data and clinical parameters for 614 ccRCC samples from TCGA (https://portal.gdc.cancer.gov). Clinical variables included survival status, duration, age, sex, tumor grade, and stage. Strawberry Perl extracted transcriptome TPM data for analysis. E-MTAB-1980 from Array Express (https://www.ebi.ac.uk/arrayexpress/) served as validation data with 101 tumor samples. The R “SVA” package addressed batch effects, removing genes with average expression below 1. MitoCarta3.0 provided 1136 protein-coding human genes with reliable mitochondrial localization. From KEGG Pathway (https://www.genome.jp/dbget-bin/www.bget?pathway+hsa04217), 159 necroptosis-related genes were obtained. In R using the “limma” package, we constructed the TCGA dataset's differential expression matrix. Criteria were set as |log2FC|> 1 and adj.*P*.Val < 0.05. Differences were visualized through volcano plots and Bland–Altman plot.

## Identification of ccRCC necroptosis-related MTGs

I obtained ccRCC-mitochondrial DEGs by intersecting genes encoding mitochondrial proteins and ccRCC-associated DEGs. For ccRCC necroptosis DEGs, an overlap analysis of necroptosis-related genes with ccRCC-associated DEGs was performed. To ensure credibility, GO and KEGG pathway enrichment analyses were conducted. Spearman correlation coefficient, based on expression profiles of ccRCC necroptosis-related DEGs and mitochondrial dysfunction-related DEGs, identified ccRCC necroptosis-related MTGs using specific criteria (|R^2|> 0.3, *p* < 0.05).

## Construction and validation of prognostic signatures based on MTGs

We conducted thorough univariate Cox regression and LASSO regression analyses on 542 cancer patients from the TCGA database to identify the most critical mitochondrial genes (MTGs) for prognosis. Subsequently, we formulated a prognostic model specifically focused on necrosis-related mitochondrial genes (nc-MTGs). The prognostic risk score for an individual patient is derived using the following formula:

$${\text{Risk score}} = \sum\limits_{i = 1}^{n} {{\text{Exp}}_{{\text{i}}} }\upbeta _{{\text{i}}}$$

In the given equation, “Exp” and “β” denote the adjusted expression value and regression coefficient of gene i, respectively. The entire patient cohort was subsequently partitioned into high and low-risk cohorts according to the median risk score. Kaplan–Meier (K-M) survival curves were constructed using the log-rank test to assess overall survival (OS) outcomes. Receiver operating characteristic (ROC) curves were constructed to assess the diagnostic value of MTG-derived prognostic attributes. Heat maps visually represented changes in expression profiles of necroptosis-related MTGs in high- and low-risk groups. Combined with the survival curve, we finally selected a molecule for subsequent experimental verification. For external validation, the E-MTAB-1980 dataset was used, following the same formula and validation techniques. Gene Set Enrichment Analysis (GSEA) was employed to investigate molecular mechanisms distinguishing low- and high-risk cohorts, with significance set at *p* < 0.05.

## Establish and evaluate a nomogram

We performed univariate and multivariate COX regression analyzes on risk scores and clinical data. Using the R "rms" package along with significant clinical variables (*p* < 0.05), we developed a nomogram. This nomogram can be used as a predictive tool to evaluate 1-, 3-, and 5-year overall survival in patients with ccRCC. To verify the accuracy of nomogram predictions, we generated receiver operating characteristic (ROC) curves and calibration plots in the TCGA and E-MTAB-1980 cohorts.

## Analysis of immune cell infiltration and immunotherapy

We utilized a gene set encompassing 28 immune-related cell types [7] and assessed the extent of immune cell infiltration within different risk groups employing the "CIBERSORT" algorithm (*p* < 0.05). The R "fmsb" package facilitated the visualization of these distinctions. Furthermore, we identified potential immune checkpoints from previously published literature. The variances in gene profiles associated with immune checkpoints between the high- and low-risk groups were also examined. Using the TCGA dataset, we predicted prognostic scores for necrosis-related MTGs, evaluated the responsiveness to immunotherapy, and depicted these variances via violin plots.

## Drug sensitivity analysis half inhibitory concentration

The half maximal inhibitory concentration (IC50), a pivotal metric for assessing drug sensitivity, represents the concentration at which a drug can effectively impede 50% of cell or tumor growth. Leveraging the R "prorophetic" package, we conducted IC50 value predictions for drugs across the high-risk and low-risk groups. Any *p*-value below 0.05 was deemed statistically significant, and these differences were illustrated using boxplots.

## Single-cell analysis

Combined with the heat map of nc-MTGs expression profile and survival curve, BID was finally selected for further validation analysis. In our single-cell investigations, we employed three datasets (KIRC_GSE145281_aPDL1, KIRC_GSE139555, and KIRC_GSE121636) from the TISCH database to assess the expression levels, distribution, and quantity of BID within the tumor microenvironment (TME)-related immune cells and various other immune cell types.

## Validation of BH3 interacting domain death agonist (BID) expression in ccRCC cell lines

Fluorescent-based real-time quantitative PCR (RT-qPCR) was employed to evaluate the expression profiles of necrosis-associated mitochondrial genes (nc-MTG). TRIzol reagent was used to extract total RNA, and Takara Prime Script RT kit was used to synthesize cDNA. Subsequently, RT-PCR was performed using SYBR Green (Roche, Switzerland). The relative expression level of genes was calculated using the 2^ − ΔΔCt method, and the reference gene was ACTIN. Primer sequences:

BID_F: TGGGACACTGTGAACCAGGAGT.

BID_R: GAGGAAGCCAAACACCAGTAGG.

## Cell cultivation and transfection with small interfering ribonucleic acid (siRNA)

Renal cancer cell lines 786-O and OS-RC-2 were cultured in RPMI-1640. ACHN, CAKI and HK-2 cell lines were cultured in DMEM. All media were supplemented with 10% fetal bovine serum (FBS, Gibco, Australia) and 1% penicillin–streptomycin. All cell lines were purchased from Procell Life Technology Co., Ltd. (Wuhan, China) (http://www.procell.com.cn/) and cultured in a humidified incubator at 37 °C, 5% CO2. BID siRNA and its siRNA control were transiently transfected using Lipofectamine 2000 (Invitrogen). The SiRNA sequence of BID is as follows:

BID-SiRNA-1 (5'-GUGAGGAGCUUAGCCAGAAAUTT-3').

BID-SiRNA-2 (5'-GGUCGGAGGACCGGAACATT-3').

NC-SiRNA (5'-UUCUCCGAACGUCACGUTT-3').

## Cell migration and proliferation

Transwell and wound-healing assays were performed to evaluate the migration ability of cells. After transfection, 40,000 ACHN and CAKI cells were suspended in serum-depleted medium and inoculated into the upper compartment. At the same time, the lower chamber was filled with 800 μL of culture medium containing 20% FBS. After 24 h, cells were removed, fixed with methanol, and stained with 1% crystal violet solution for 30 min. Randomly selected areas were then imaged under the microscope and cells passing through the chamber were quantified. Cells were seeded in 6-well plates at 50% density, incubated overnight and transfected with small interfering fragments to achieve 90% coverage. Subsequently, meticulously generate two vertical incisions in each well using a 200-µl pipette tip, delicately rinse cells with PBS to remove cellular debris, and cultivate in serum-deprived DMEM. Finally, images of wound healing at 0 and 24 h were taken under microscopic observation. 1,000 transfected ACHN and CAKI cells were seeded into each well of a 6-well plate and cultured for 14 days. Subsequently, they were fixed with 4% paraformaldehyde for 20 min and stained with 1% crystal violet for 30 min. In addition, transfected cells were seeded in 96-well plates at a density of 5000 cells per well. After 24 h, stain according to the instructions is provided in the Edu assay kit (C10310-1, RiboBio).

## Statistical analysis

All bioinformatics results were analyzed using R software and experimental data visualized using GraphPad Prism 9.5.1. Student's t test was used to compare means between two groups, while one-way/two-way ANOVA was used to compare means between multiple groups, with a *p*-value of 0.05 considered statistically significant.

## Results

### Basic information

Supplementary Fig. 1 encapsulates the comprehensive flow chart outlining our study. We procured the gene expression profiles from 614 renal clear cell tumor patient samples sourced from the TCGA database, encompassing 542 tumors and 72 adjacent normal patients. Samples lacking clinical information were omitted from the analysis. Additionally, the expression profile data of 101 ccRCC patient tumor samples were sourced from the ArrayExpress database, serving as a test set for external validation. Data on 537 cases of clear cell renal cell carcinoma were downloaded from the TCIA database, including information on immunotherapy.

## Identification of Mito-DGEs and Necr-DGEs

**Fig. 1:**
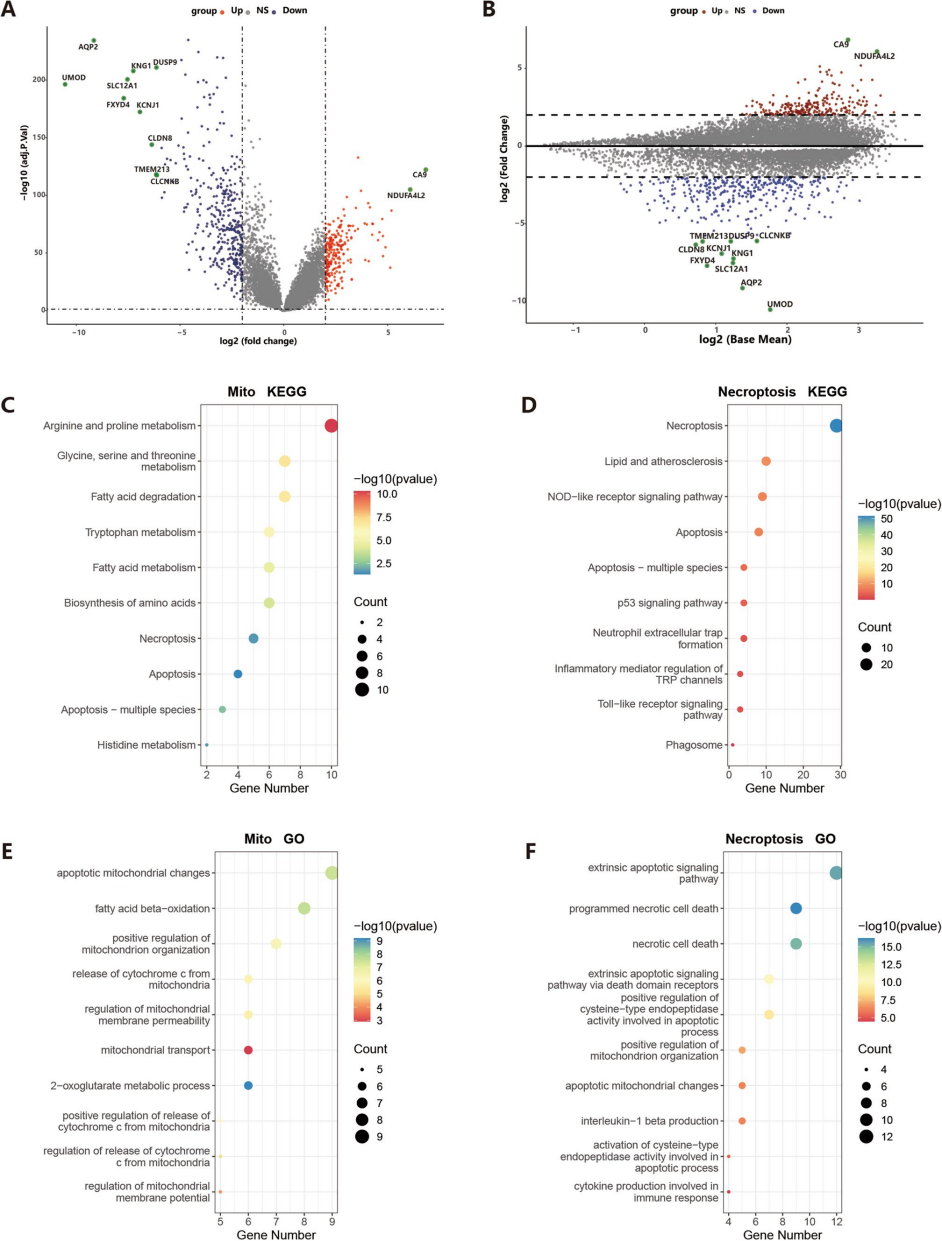
DEGs identification and enrichment analysis. **A** Volcano plot showing DEGs in the ccRCC and the normal samples. **B** Bland–Altman plot showing DEGs in the ccRCC and the normal samples. **C**–**D** KEGG enrichment results of 122 ccRCC-Mito DEGs (**C**) and 29 ccRCC-Necroptosis DEGs (**D**). (E–F) GO enrichment results of 122 ccRCC-Mito DEGs (**E**) and 29 ccRCC-Necroptosis DEGs (**F**)

We selected 1,136 mitochondrial dysfunction-related genes and 159 necroptosis-related genes from the Mito Carta 3.0 and KEGG Pathway databases, respectively. After excluding genes with expression levels lower than 1 in the TCGA data set, the R "limma" package was employed for conducting differential analysis between the tumor group and the normal group, identifying 2,580 differentially expressed genes (DEGs)—1,372 up-regulated genes and 1,208 down-regulated genes(Supplementary Table S1). Volcano plots and Bland–Altman plots visualized the differences (Fig. 1A–B). Functional exploration through GO and KEGG enrichment analysis revealed overexpressed pathways in mitochondrial DEGs, including apoptosis, necroptosis, and fatty acid metabolism (Fig. 1C). The most abundant GO pathways included mitochondrial apoptotic changes, mitochondrial transport, and fatty acid β-oxidation (Fig. 1D). For Necr-DEGs, KEGG analysis identified pathways related to necroptosis, inflammatory mediator regulation of TRP channels, and p53 signaling (Fig. 1E). GO analysis highlighted pathways related to programmed cell death, apoptosis, and cytokine production in the immune response (Fig. 1F). Correlation analysis led to the identification of 113 necrosis-related mitochondrial genes (nc-MTGs), suggesting an interplay between necroptosis, mitochondrial dysfunction, and immune responses in ccRCC.

## Construct and validate a model of necrosis-related mitochondrial dysfunction

**Fig. 2:**
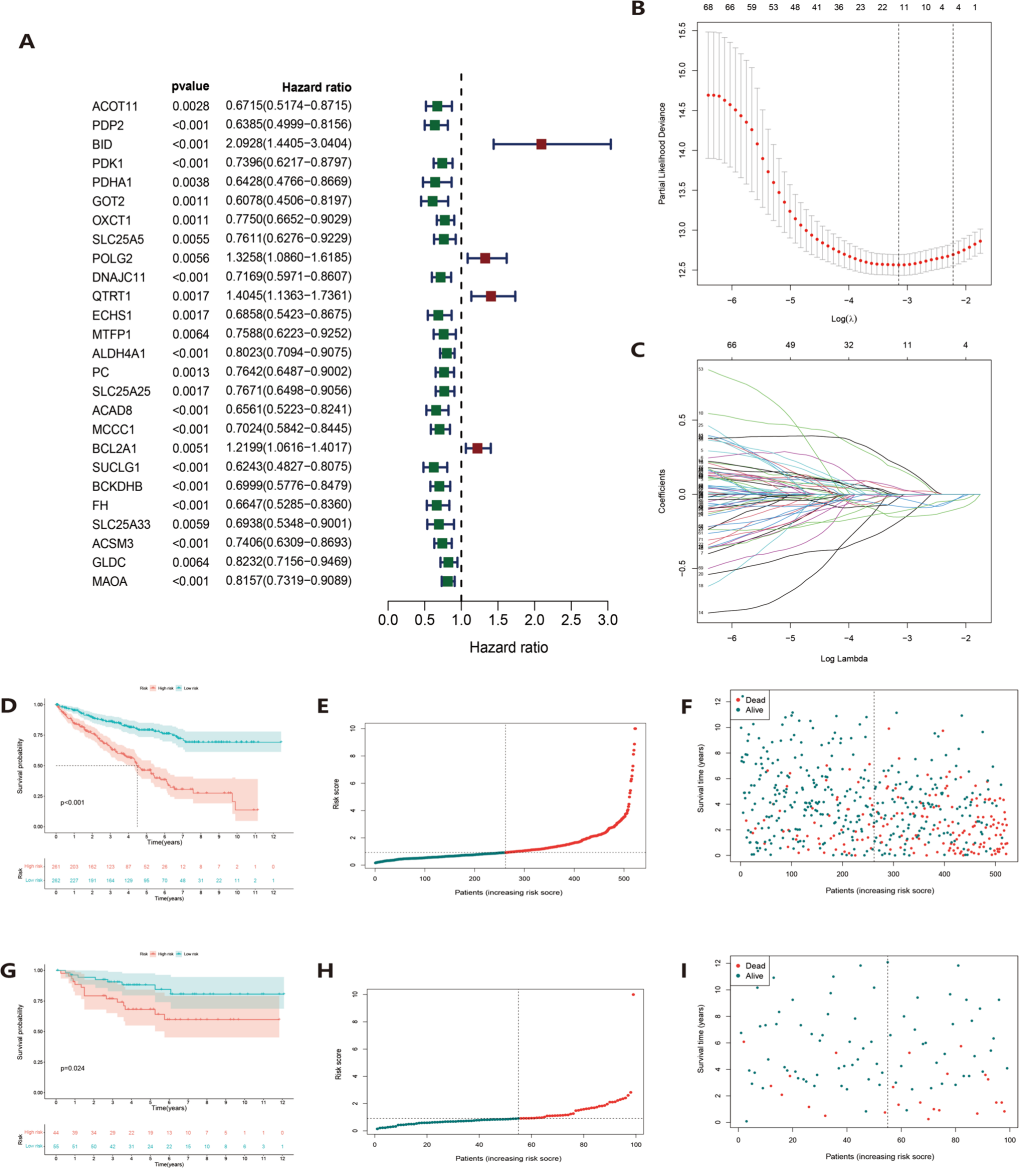
**A** Univariate Cox regression analysis shows that 26 necrosis-related MTGs are significantly associated with OS. **B**–**C** LASSO coefficient curves were constructed based on 26 predicted necrosis-related MTGs and 6 genes were selected. **D**–**F** Kaplan–Meier curves, distribution of risk scores, and distribution of patients in the training group. **G**–**I** Kaplan–Meier curves, risk score distribution, and test group patient distribution

**Fig. 3:**
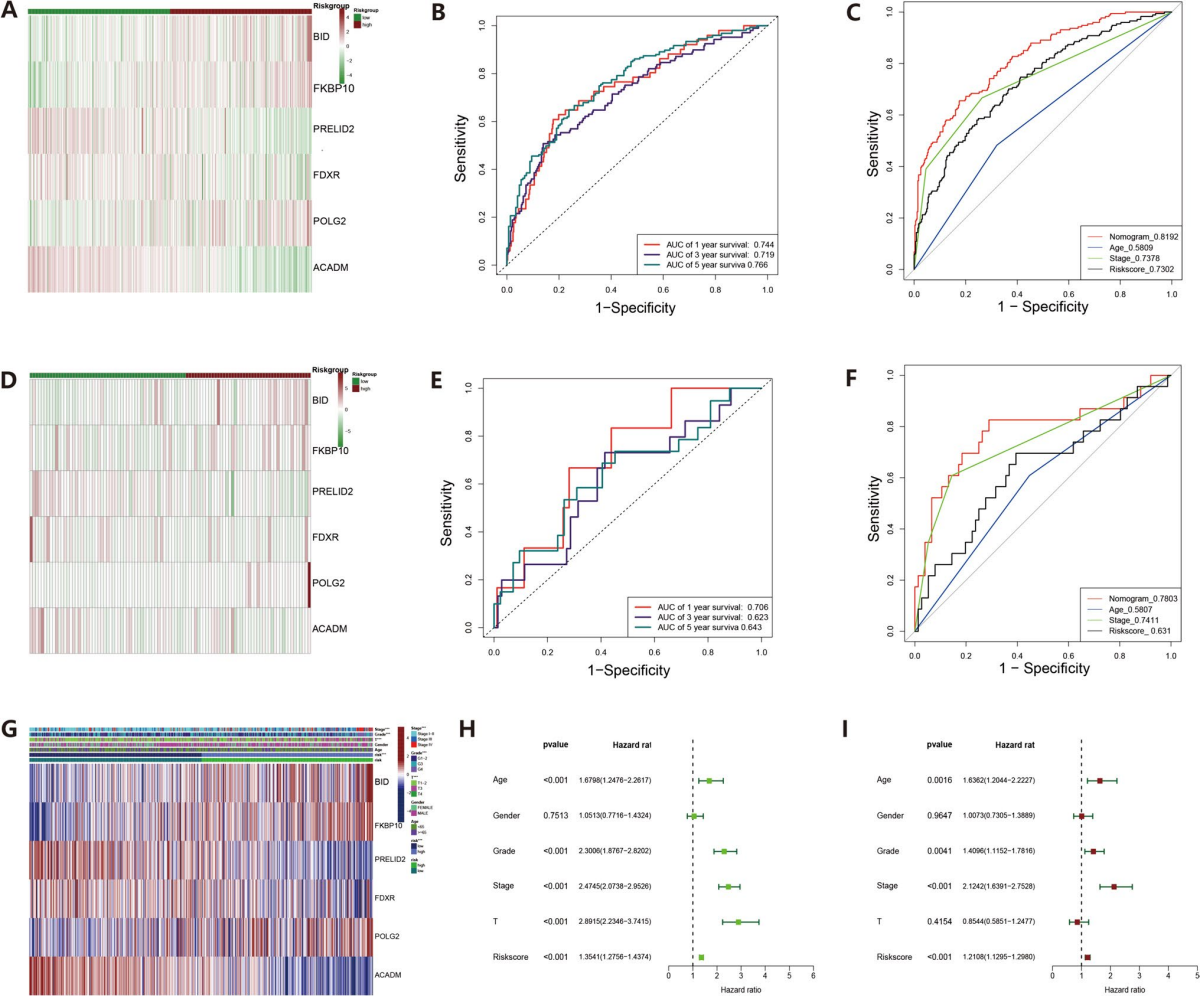
Prognostic analysis of nc-MTGs in training group and test group. **A**, **D** Heatmap shows the difference of 6 nc-MTGs in training group and test group. **B**, **E** Receiver operating characteristic (ROC) analysis of training group and test group. **C**, **F** ROC curve analysis compares the predictive ability of nomogram and other clinical pathological indicators in the training group and test group. **G** Heat map showing the differences of 6 nc-MTGs among different clinical groups. **H** Univariate prognostic analysis. **I** Multivariate prognostic analysis

To improve the accuracy of the prediction model, we use the TCGA cohort for training and the E-MTAB-1980 cohort for testing. Univariate Cox regression identified 26 mitochondrial molecules associated with necroptosis. Four, namely BID, POLG2, QTRT1, and BCL2A1, were designated as high-risk prognostic MTGs, whereas the others were low-risk MTGs (*p* < 0.01, Fig. 2A). LASSO regression detailed in Supplementary Table S2 highlighted six necrosis-related MTGs as prognostic factors in ccRCC patients. The reliability of the model was verified by cross-validation (Fig. 2B–C). Patients were divided into high-risk and low-risk groups based on the median risk score. Kaplan–Meier survival analysis showed that patients with low-risk ccRCC had significantly improved overall survival (Fig. 2D). Scatter plots and risk factor correlation plots demonstrated significant correlations between survival time and risk scores in ccRCC patients. Higher scores were associated with higher risk of death from ccRCC (Fig. 2E–F). A similar approach was followed in the test arm, with Fig. 3G–I illustrating that patients with low-risk ccRCC had higher overall survival and increased mortality with increasing risk score.

## Independent prognostic analysis of nc-MTGs

**Fig. 4:**
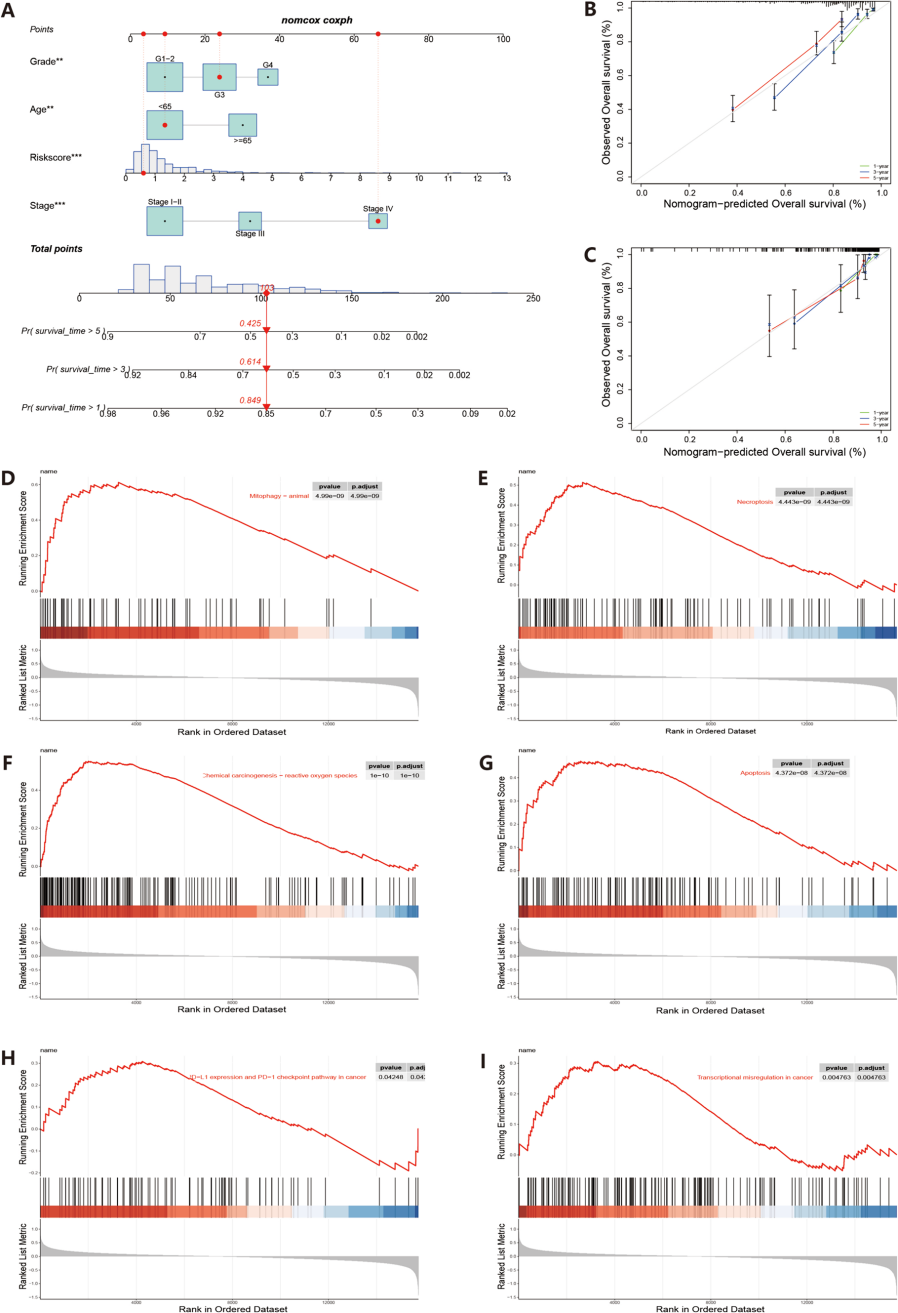
Clinical correlation analysis and GSEA. **A** Nomogram constructed to predict survival based on training group. **B**–**C** Training and test set calibration curves. **D**–**I** GSEA analysis

The heatmap (Fig. 3A) reveals high expression of three necrosis-related mitochondrial genes (nc-MTGs)—PRELID2, FDXR, and ACADM in the low-risk group, while BID, FKBP10, and POLG2 exhibit significant expression in the high-risk group. This pattern persists within the test cohort (Fig. 3D). These findings establish the 6 nc-MTGs as distinctive prognostic markers in ccRCC patients. Univariate (nc-MTGs (HR: 1.354, 95% CI: 1.276–1.437)) and multivariate Cox analyses (nc-MTGs (HR: 1.211, 95% CI: 1.130–1.298)) both confirm that nc-MTGs are independent prognostic risk factors in ccRCC patients (Fig. 3H–I). The nomogram (Fig. 4A) was used to predict short-term, mid-term, and long-term survival rates. The area under the curve (AUC) values of the training group were 0.744, 0.719, and 0.766 (Fig. 3B), and the AUC values of the test group were 0.706, 0.623, and 0.643 (Fig. 3E). The AUC value of the nomogram outperformed age, pathological stage, and single risk score (Fig. 3C). Calibration plots validated the model in both groups, suggesting good discriminatory power (Fig. 4B–C). The risk score model held significant weight in the combined nomogram. These findings underscore the clinical value of the nomogram in predicting survival outcomes.

## GSEA analysis of necroptosis-related MTGs

GSEA analysis further elucidated the biological functions based on necroptosis-related mitochondrial molecules. GSEA showed that the nc-MTGs prognostic model mainly regulates cancer and apoptosis-related pathways, such as necroptosis, apoptosis, mitophagy, tumor transcriptional dysregulation, chemical carcinogenesis-reactive oxygen species, and immune checkpoint pathways (Fig. 4D–I).

## Application of risk model to analyze immune characteristics of ccRCC patients

The study explored variations in immune cell infiltration and the impact of immunotherapy across diverse risk groups. Utilizing a 28-gene set of immune-related cell types [8] and the "CIBERSORT" algorithm (*p* < 0.05) (Supplementary Fig. 2A), we observed higher prevalence of macrophage M0, T cell CD4 memory activation, and Tregs in the high-risk group (Supplementary Fig. 2B–D). In contrast, mast cell quiescence, monocytes, CD4 + T cells, macrophages M1, and dendritic cells were more prominent in the low-risk group (Supplementary Fig. 2E–I). Additionally, the study investigated the regulatory potential of immune checkpoint inhibitor (ICI) genes on immune cell infiltration in tumor tissues [9]. Comparative analysis of immune checkpoint-related genes showed that representative genes of the high-risk group were significantly up-regulated, including CD40LG, CTLA4, LGALS9, PD-1, TDO2, TIGIT, TNFRSF4, TNFRSF18, and TNFRSF14 (Supplementary Fig. 3).

## Immunophenotypic scoring (*IPS*)and drug sensitivity

The study gathered data from 537 clear cell renal cell carcinoma cases inclusive of immunotherapy details sourced from the TCIA database. Using IPS files analyzed via the R "ggpubr" package, We evaluated response to immunotherapy in different risk groups. Remarkably, patients with lower risk scores demonstrated greater sensitivity to immunotherapy, and this variance held statistical significance (Supplementary Fig. 4A–D). Furthermore, the research extended to scrutinize differences in drug sensitivity among the two groups of ccRCC patients, aiming to enhance treatment efficacy. Notably, drugs like AKT.inhibitor.VIII and Nutlin.3a displayed higher IC50 values in high-risk patients compared to low-risk patients. In contrast, the IC50 values of JNK.Inhibitor.VIII, PD.0325901, Salubrinal, Bosutinib, Dasatinib, PD.0332991, Pazopanib, Sunitinib, Tipifarnib, and Imatinib in the low-risk group were notably higher compared to those in the high-risk group (Supplementary Fig. 4E–P).

## Single-cell level analysis of BID expression signature on KIRC microenvironment cells

**Fig. 5:**
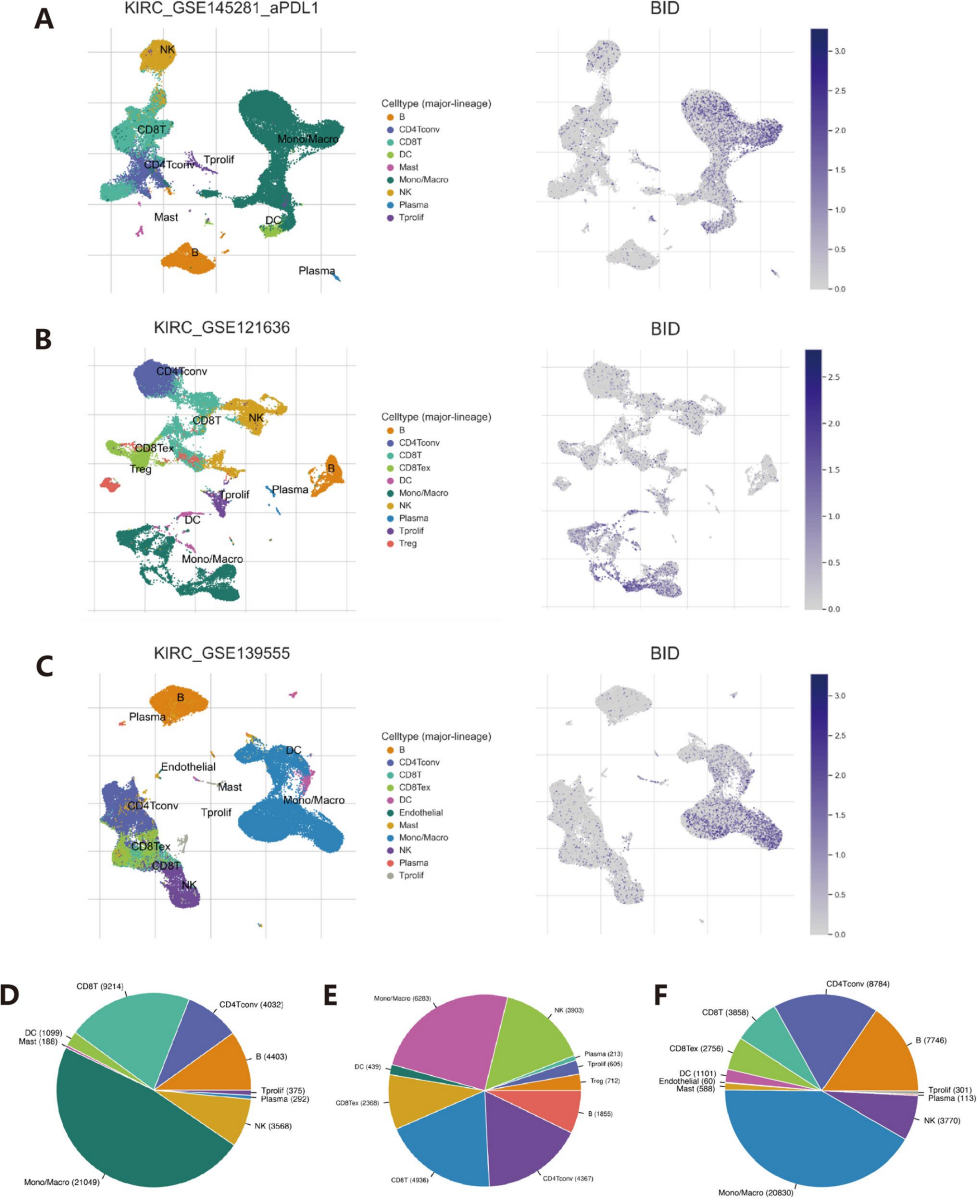
Expression of BID at the level of TME-related immune cells and distribution of immune cells. **A**–**C** Expression of BID in tumor microenvironment (TME)-associated immune cells in different datasets. **D**–**F** Distribution and number of various cells associated with the TME in each dataset

Previous studies have shown that BID, FKBP10, and POLG2 are significantly expressed in high-risk groups (Fig. 3A). We further explored the relationship between these three model genes and patient survival through the TCGA database (Supplementary Fig. 5A–C), and the results showed that significant expression of the three genes was associated with poor prognosis, which further confirmed the accuracy of our results. Reviewing the recent literature, we found that FKBP10 has been experimentally verified to promote the progression of renal clear cell carcinoma[10]. By RT-PCR, we found that BID had higher significance in ACHN and CAKI cell lines (Supplementary Fig. 5D). Therefore, we selected BID for further investigation. The study utilized three datasets (KIRC_GSE145281_aPDL1, KIRC_GSE139555, KIRC_GSE121636) from the TISCH database to explore the expression of BID within single-cell TME-related immune cells. The findings indicated that BID was primarily prevalent in monocytes/macrophages (Mono/Macro) and exhibited expression within dendritic cell (DC) clusters (Fig. 5A–C). Furthermore, moderate to lower levels of BID expression were observed in NK cells, CD8 T cells, and CD4 Tconv cells. The distribution and proportions of various immune cells associated with the TME are visualized in Fig. 5D–F. KIRC_GSE145281_aPDL1 primarily represents patients with metastatic renal cancer who underwent immunotherapy, while KIRC_GSE121636 and KIRC_GSE139555 refer to primary renal cancer patients who did not receive immunotherapy. Across these datasets, monocytes/macrophages (Mono/Macro) were the predominant cell type, followed by CD8 T cells and CD4 Tconv cells. It's speculated that the distribution of BID expression might correlate with these observed immune cell distributions.

## RT-PCR and cell functional analysis

**Fig. 6:**
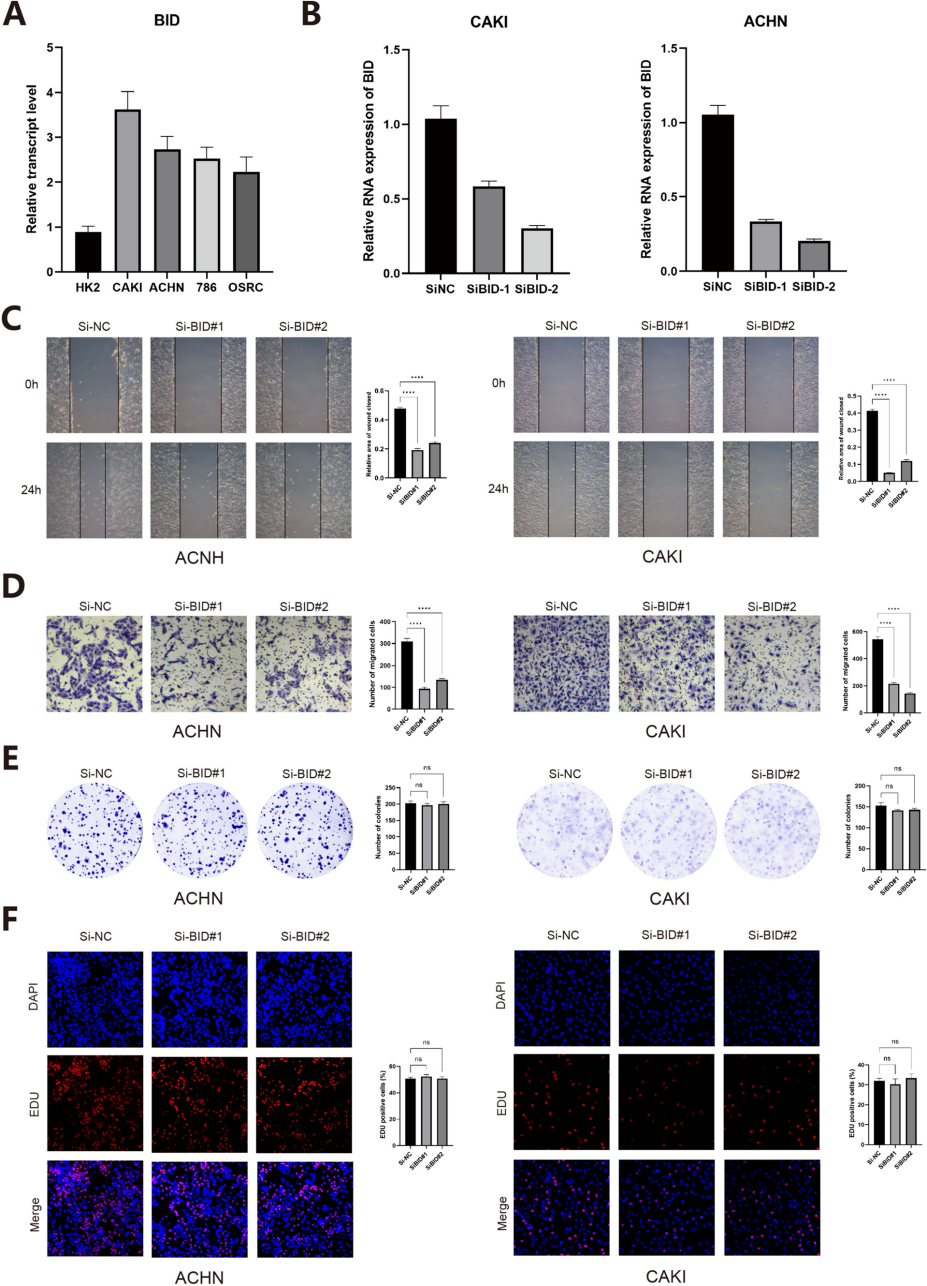
Functional verification of knockout BID. **A**–**B** BID expression in renal cell carcinoma cell lines was detected using qPCR and interference efficiency of small interfering RNAs (siRNAs) in ACHN and CAKI cell lines was also verified. **C**–**D** Effect of BID knockdown on ACHN and CAKI cell migration and corresponding quantitative analysis. **E**–**F** Effect of BID knockdown on ACHN and CAKI cell proliferation and corresponding quantitative analysis

To explore the impact of nc-MTG on cell function in ccRCC, BID was used as a target to further verify its effect on in vitro cell experiments. PCR experiments showed increased BID expression in ccRCC cell lines compared with normal kidney cells (HK-2) (Fig. 6A). To elucidate BID's role in ccRCC, a BID knockdown siRNA transfection model was established. The effectiveness of knockdown in ACHN and CAKI cells was confirmed by PCR (Fig. 6B). Wound healing and transwell assays demonstrated that reduced BID expression impaired cell migration in ACHN and CAKI cells (Fig. 6C–D). However, knocking down BID did not have a significant effect on proliferation of ACHN and CAKI cells, as observed in colony formation and Edu experiments (Fig. 6E–F). Taken together, these data clearly indicate that BID affects the migration of kidney cancer cells. These findings highlight BID's significant role in promoting ccRCC progression, suggesting its potential as a targeted therapy for personalized treatment.

## Discussion

Clear cell renal cell carcinoma (ccRCC) is a primary renal subtype that is often asymptomatic initially, leading to late diagnosis. Despite effective resection methods, the complex biology of cancer can lead to postoperative recurrence or distant metastasis. Furthermore, ccRCC exhibits significant resistance to conventional radiotherapy and chemotherapy [11]. Mitochondria are important eukaryotic organelles that play an important role in various types of apoptosis, including necroptosis [12]. Necroptosis is characterized by caspase-independent features, similar to necrosis with features such as cell swelling and membrane rupture [8].

Recent studies explore the role of mitochondrial genes (MTGs) in clear cell renal cell carcinoma (ccRCC). Zhang et al. identified MFN2 as a potential prognostic marker [13]. Yan et al. found that inhibiting NR3C1 restricts ccRCC proliferation and migration via stress-mitophagy [11]. Luo et al. proposed MFN2's role in regulating ccRCC progression through mitochondria-dependent EGFR dephosphorylation [14]. Li et al. developed a risk prediction model for necroptosis-related genes in ccRCC [15]. Despite these findings, the prognostic relevance of necroptosis-related mitochondrial genes (nc-MTGs) in ccRCC remains limited. This study aims to further explore the association between nc-MTGs and ccRCC prognosis.

In this study, we analyzed the TCGA database to extract Differentially Expressed Genes (DEGs). Integrating Mito Carta 3.0 and KEGG Pathway datasets, we identified six necroptosis-related mitochondrial genes (nc-MTGs) linked to prognosis—BID, FKBP10, PRELID2, FDXR, POLG2, and ACADM. Using single-factor Cox, LASSO, and multi-factor Cox regression analyses, we established a prognostic model for ccRCC, categorizing patients into high and low-risk groups. Kaplan–Meier, heatmap visualization, and ROC analysis validated the model's robust predictive capacity. External validation used the E-MTAB-1980 dataset. Analysis of immune-related factors and drug sensitivities highlighted differences between the different groups. Elevated BID, FKBP10, and POLG2 expression in the high-risk group suggests their role as risk factors, associated with a poorer ccRCC prognosis.

Recent studies have found that BID is one of the only proteins in BH45 that controls BAK and BAX and is considered to play a key role in the apoptosis signaling pathway [16]. Hu et al. contend that elevated expression of BID is linked to unfavorable prognosis in ccRCC patients [17]. The FK506-binding protein (FKBP) is a family of intracellular receptors that can bind specifically to the immunosuppressant FK506 and rapamycin [18]. Cai et al. proposed that FKBP10 promotes glioma cell proliferation by activating the AKT-CREB-PCNA axis [19]. High VSX1 expression promotes the aggressiveness of clear cell renal cell carcinoma through transcriptional regulation of FKBP10[20]. Polymorphisms in POLG2 gene thought to be genetically associated with aggressive bladder cancer in Japanese men [21]. Inhibition of ACADM-mediated fatty acid oxidation by Ma et al. promotes hepatocellular carcinoma through the CAV1/SREBP1 signaling pathway [22], but its role in ccRCC requires further study. Wang et al. claimed that PRELID2 is involved in follicle development in polycystic ovarian syndrome (PCOS) [23], but the role of PRELID2 in tumors requires further exploration. Ferredoxin reductase (FDXR) is a target of p53 and regulates p53-dependent apoptosis. Zhang et al. suggest that FDXR and p53 regulate each other and that the FDXR-p53 loop is critical for tumor suppression through iron homeostasis [24].

Through a meticulous examination of pertinent clinical parameters, we identified four autonomous prognostic factors (age, grade, stage, and risk score) pivotal in assessing the predictability of our risk model. The receiver operating characteristic (ROC) curve substantiated the model's robustness in forecasting 1-, 3-, and 5-year survival rates. To enhance the model's practical utility in clinical settings, a nomogram was employed to graphically represent the survival prognosis for ccRCC patients. Its validity was rigorously assessed and confirmed using an external dataset (E-MTAB-1980), establishing the nomogram's prowess in forecasting patient outcomes as evidenced by the calibration curves.

The GO and KEGG analyses delineated the engagement of these necrosis-related mitochondrial genes (nc-MTGs) in fundamental cellular processes. These genes are implicated in apoptosis, redox reactions, dysregulation of cancer transcription, immune checkpoint pathways, and the chemical induction of reactive oxygen species. Mitochondria, recognized as pivotal organelles, play a multifaceted role by generating energy and actively participating in a spectrum of biological processes. Their malfunction can lead to damage in enzymes of the tricarboxylic acid (TCA) cycle, leakage in the electron respiratory chain, and consequent oxidative stress. This results in a significant alteration in cellular metabolism and signal transduction pathways, contributing notably to apoptosis and resistance to therapy, prominently influential in the development of various human cancers [25]. Simultaneously, mitochondria serve as synthetic organelles capable of not only producing adenosine triphosphate (ATP) but also synthesizing macromolecules. This versatility allows them to cater to the diverse metabolic requisites of different immune cells [26]. Singhal et al. identified metformin, an anti-diabetic medication acting on mitochondrial complex I, as an immunomodulator in tuberculosis [27]. This finding suggests that targeting human mitochondrial metabolism can influence the immune response in disease scenarios. In our study focused on clear cell renal cell carcinoma (ccRCC), we observed promising results for immunotherapy and targeted therapy. Up to now, more than ten kinds of targeted drugs and immune drugs have been approved for the treatment of advanced renal cell carcinoma at home and abroad. Tyrosine kinase inhibitors and VEGF antibodies are widely used as first-line or sequential therapy for renal cell carcinoma. To date, nine drugs have been approved by the U.S. Food and Drug Administration (FDA) for first-line or second-line treatment of advanced RCC: sunitinib, sorafenib, pazopanib, axitinib, temsirolimus, everolimus, bevacizumab + interferon, cabozantinib, and Lenvatinib [28]. At present, the effective rate of targeted therapy is about 30%. Most targeted drugs have significant effect on medium- and low-risk patients, but the effect is not obvious in high-risk patients [29]. Immunotherapy drugs used in the treatment of renal cell carcinoma in China include nivolumab, pembrolizumab, interleukin 2 and recombinant human interferon α2b.For medium to high-risk patients, dual immunotherapy is superior to single targeted therapy [30]. Three large Phase III clinical studies of PD1/PD-L1 in combination with TKI for first-line treatment of RCC, Immotion-151, JAVELIN Renal 101, and KEYNOTE-426, have also achieved breakthrough results, which also provide clinicians with more treatment options [29, 31, 32].

Targeting mitochondrial metabolism to influence immune responses in disease situations remains to be further explored, so we analyzed the relationship between necrosis-associated mitochondrial genes (nc-MTGs) and the tumor immune microenvironment. Immunocyte infiltration analysis showed that higher risk scores were positively correlated with T cell CD4 memory activation, Tregs, and macrophage M0, and negatively correlated with mast cell quiescence, lymphocytes, CD4 + T cells, macrophages M1, and dendritic cells. Increased infiltration of Treg cells has been demonstrated in progressive ccRCC, suggesting an association with poor prognosis. [33, 34]. Conversely, stasis in dendritic cells, mast cells, and eosinophils has exhibited a notable correlation with enhanced prognosis [35]. IFNγ and lipopolysaccharide (LPS) have demonstrated the capability to instigate a proinflammatory macrophage phenotype (M1), and several factors associated with M1 have been linked to protracted survival in ccRCC [36]. We also used the TISCH database to probe BID expression in single-cell immune populations associated with tumor microenvironment (TME). Our results showed that BID is mainly present in monocytes/macrophages (Mono/Macro) and dendritic cells (DC), and previous studies have demonstrated the role of the necrosis-apoptosis axis in macrophages. Our results therefore support the conclusion that BID overexpression is positively correlated with risk score and suggests a poor prognosis. With the approval of immune checkpoint inhibitors targeting PD-L/CTLA-4, overall survival has improved for many cancers [37, 38]. When we compared immunotherapy response among different risk groups, we noted that in CTLA-4 positive/PD-L renal cell carcinoma patients, the response rate to immunotherapy was significantly higher in the high-risk group than in the low-risk group, while there was no discrepancy in the efficacy of immunotherapy among different risk groups in CTLA-4 negative/PD-L patients. Examination of immune checkpoint-related genes revealed that the expression levels of CD40LG, CTLA4, LGALS9, PD-1, TDO2, TIGIT, TNFRSF4, TNFRSF18, and TNFRSF14 were significantly increased in the high-risk group. We generally believe that the higher the expression of immunosuppressive molecules, the better the effectiveness of immune checkpoint inhibitors. Therefore, whether we believe that immune checkpoint expression levels are not the only determinant of immunotherapy, the immune microenvironment of the tumor may be related to this, and as mentioned earlier, a high proportion of patients are in an immunosuppressive microenvironment. In addition, our analysis delves into the relationship between different risk groups and drug sensitivity. The study found that different targeted drugs have different efficacy in different risk groups. For example, high-risk populations are more sensitive to NK inhibitors VIII, PD.0325901, Salubrinal, Bosutinib, Dasatinib, PD.0332991, Pazopanib, Sunitinib, Tipifarinib, and Imatinib. In summary, immunization + targeted therapy is also a good option for high-risk patients. This further increases the reliability of our findings and provides insights into clinical treatment.

We started our experiments with several renal carcinoma cell lines. From these cell lines, we isolated RNA and performed a comparative analysis of BH3 interacting domain death agonist (BID) expression between normal and tumor cells. The results of our PCR analysis clearly indicate that BID is up-regulated in tumor tissue. Subsequently, we will explore the effects of BID reduction on tumor cells by functional experiments in CAKI and ACHN cell lines, where we reduced BID expression by small interfering RNA (siRNA). The results showed that the intervention significantly inhibited renal cell migration, but had no effect on cell proliferation. Simultaneously, we are endeavoring to achieve a more comprehensive understanding of the underlying mechanisms involved in this context.

Our study shows that nc-MTGs have important clinical significance in the prognostic assessment of ccRCC patients. The key roles of these molecules in apoptosis, energy metabolism and antioxidant stress make them potential therapeutic targets and prognostic markers. In clinical settings, by detecting the expression levels of these nc-MTGs, high-risk patients can be identified earlier and more accurately for individualized treatment. As biomarkers, nc-MTGs can provide detailed information about the metabolic state and apoptosis mechanism of tumor cells, complementing and improving the existing prognostic assessment system. By incorporating these molecules into the assessment system for ccRCC patients, we can more accurately predict patient progression and treatment response, optimize treatment strategies, and improve outcomes.

Although genes associated with mitochondrial dysfunction and necrotizing apoptosis provide valuable insights into tumor prognosis and the immune microenvironment, it must be acknowledged that our study has certain limitations. Firstly, our observation that BID expression interferes with tumor cell migration without affecting proliferation merits further exploration. Second, our study is based on public transcriptome data supplemented by basic in vitro experiments. Therefore, more complex in vitro studies and animal experiments are necessary to further explore the underlying mechanisms of mitochondrial dysfunction in ccRCC. Finally, since this is a retrospective study, prospective trials involving larger patient cohorts are needed to improve the reliability of the scoring system.

## Conclusion

In summary, our study identified mitochondrial features in ccRCC patients. On one hand, it enhances the predictability of survival outcomes in ccRCC patients; on the other hand, the six nc-MTGs (BID, FKBP10, PRELID2, FDXR, POLG2, and ACADM) within this feature can also assess the immune status and immune checkpoints of ccRCC patients. The screened BID gene was confirmed to be involved in enhancing tumor cell migration through in vitro experiments. This discovery holds promising prospects for unveiling new potential targets and treatment strategies in the realm of renal cell carcinoma therapy.

## Supplementary Information

Below is the link to the electronic supplementary material.

Supplementary file1 (JPG 60 KB)

Supplementary file2 (JPG 2448 KB)

Supplementary file3 (JPG 3045 KB)

Supplementary file4 (JPG 3346 KB)

Supplementary file5 (JPG 323 KB)

Supplementary file6 (DOCX 489 KB)

Supplementary file7 (DOCX 14 KB)

## Data Availability

All data are from open access databases. TCGA database, Array Express database, MitoCarta3.0 databases, KEGG Pathway databases, and TCIA database.
