# Supplementary figures and images for: Exploring necrosis-associated mitochondrial gene signatures: revealing their role in prognosis and immunotherapy of renal clear cell carcinoma
Source: Clin Exp Med. 2024 Jul 18;24(1):161. doi: 10.1007/s10238-024-01426-9 (PMC11258092; doi:10.1007/s10238-024-01426-9)

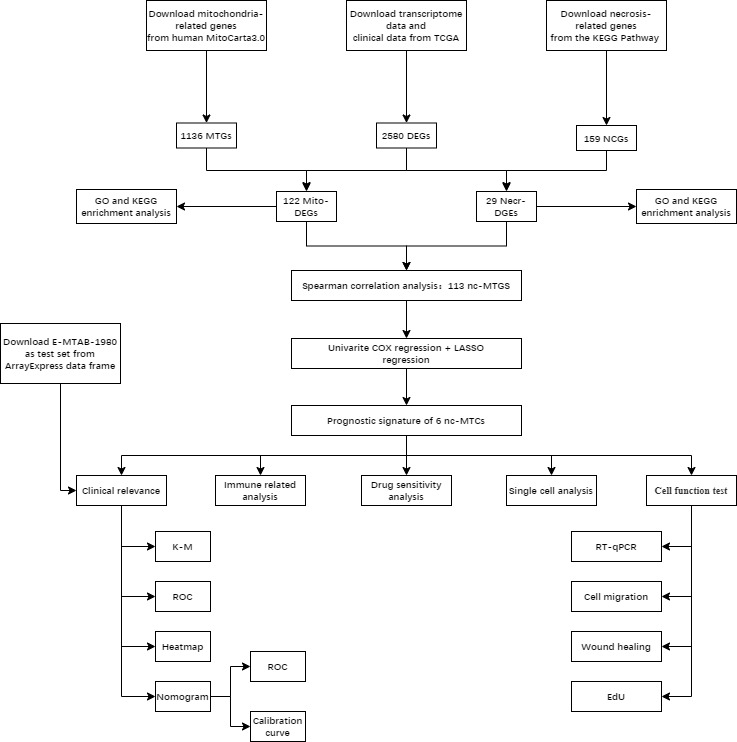

Supplement: Supplementary file 1 — Supplementary file1 (JPG 60 KB) [file 10238_2024_1426_MOESM1_ESM.jpg]

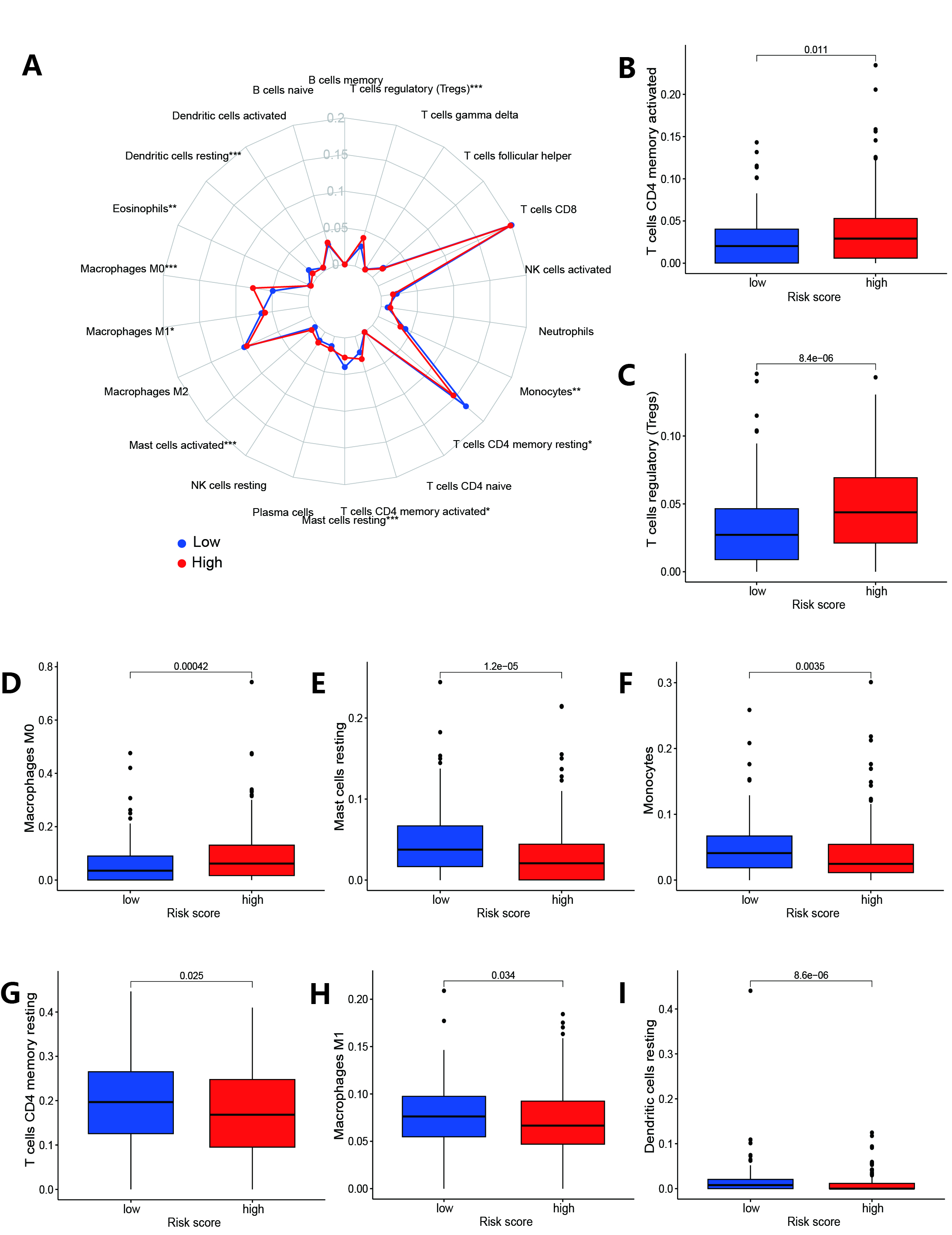

Supplement: Supplementary file 2 — Supplementary file2 (JPG 2448 KB) [file 10238_2024_1426_MOESM2_ESM.jpg]

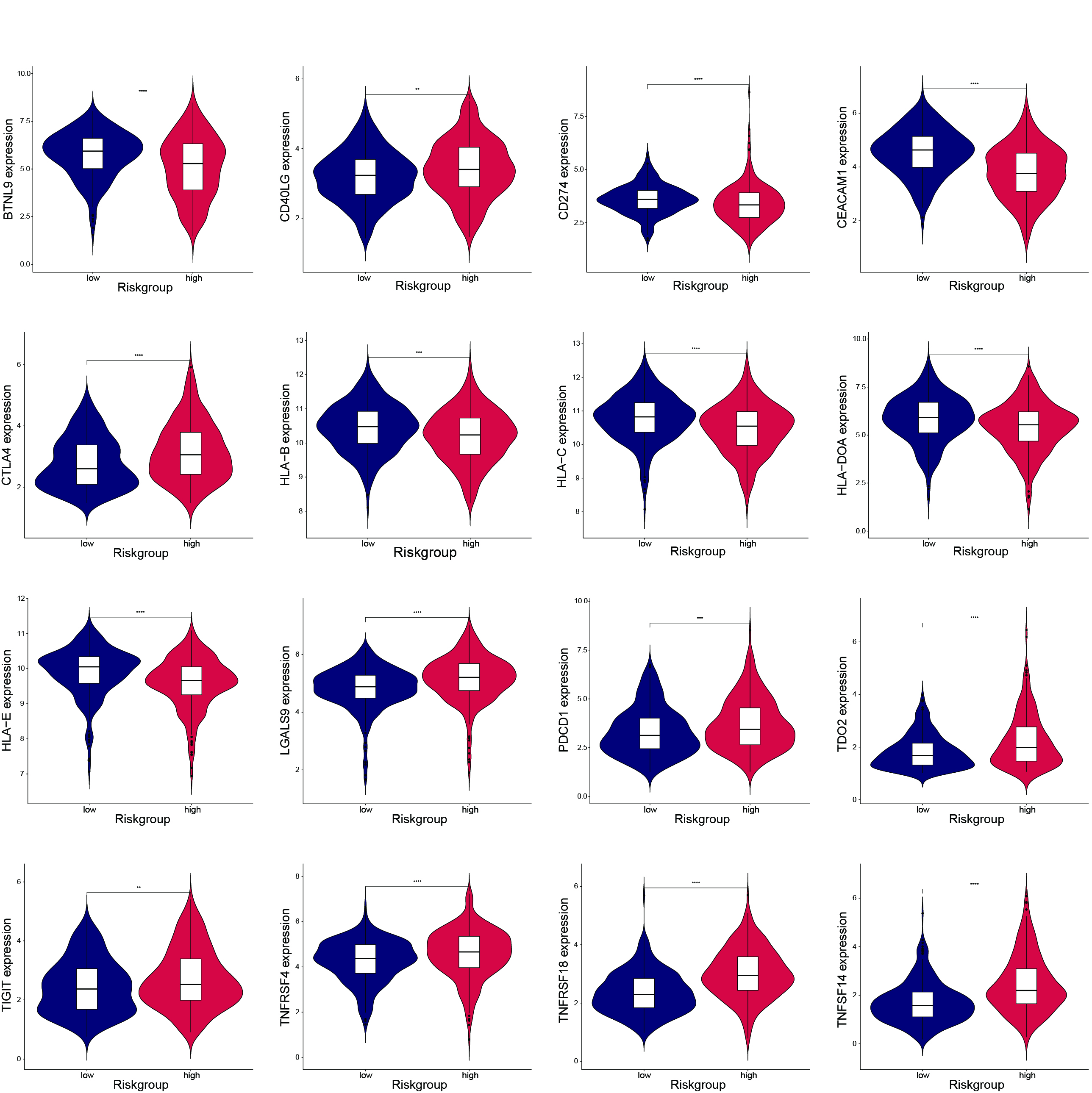

Supplement: Supplementary file 3 — Supplementary file3 (JPG 3045 KB) [file 10238_2024_1426_MOESM3_ESM.jpg]

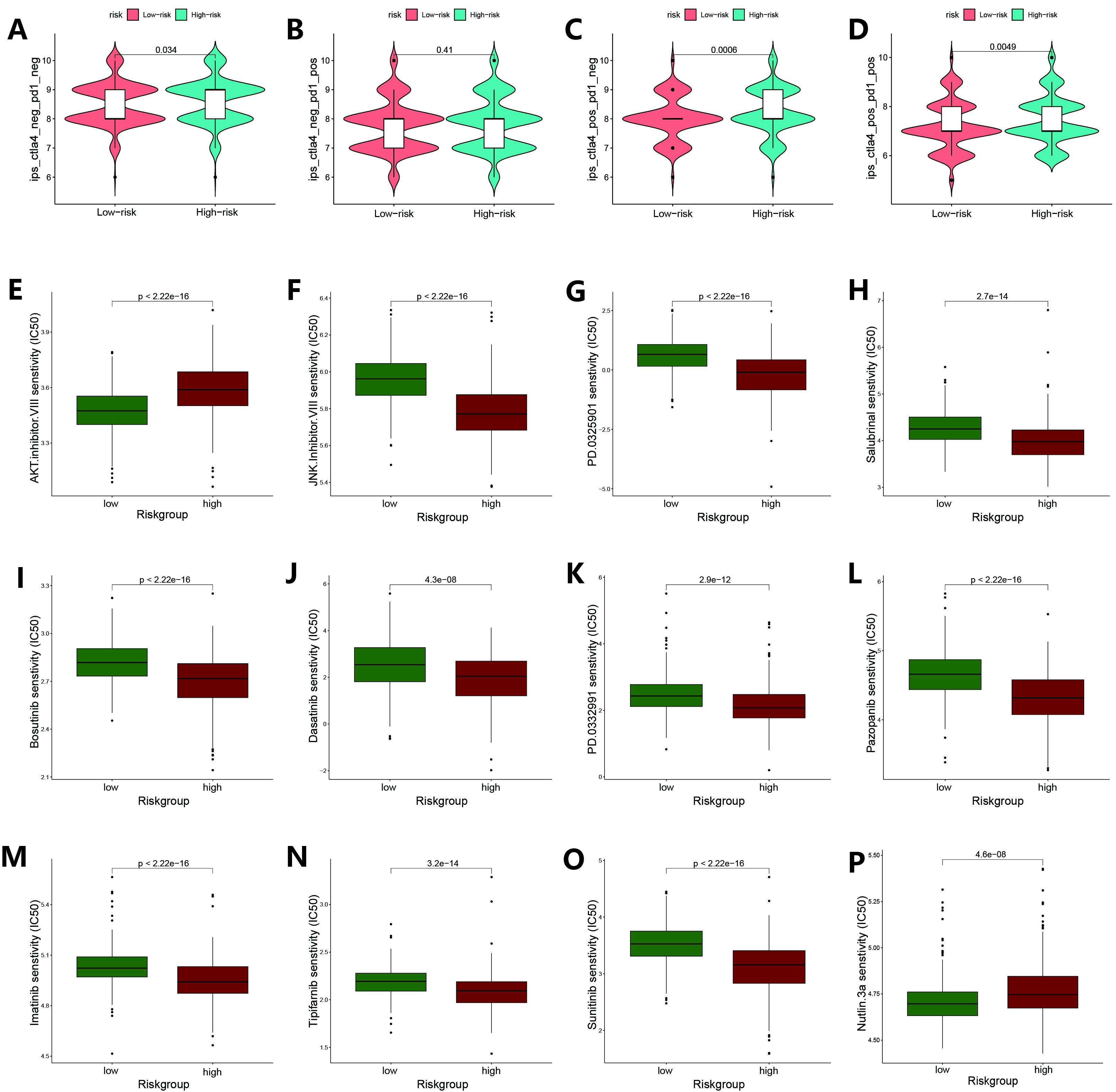

Supplement: Supplementary file 4 — Supplementary file4 (JPG 3346 KB) [file 10238_2024_1426_MOESM4_ESM.jpg]

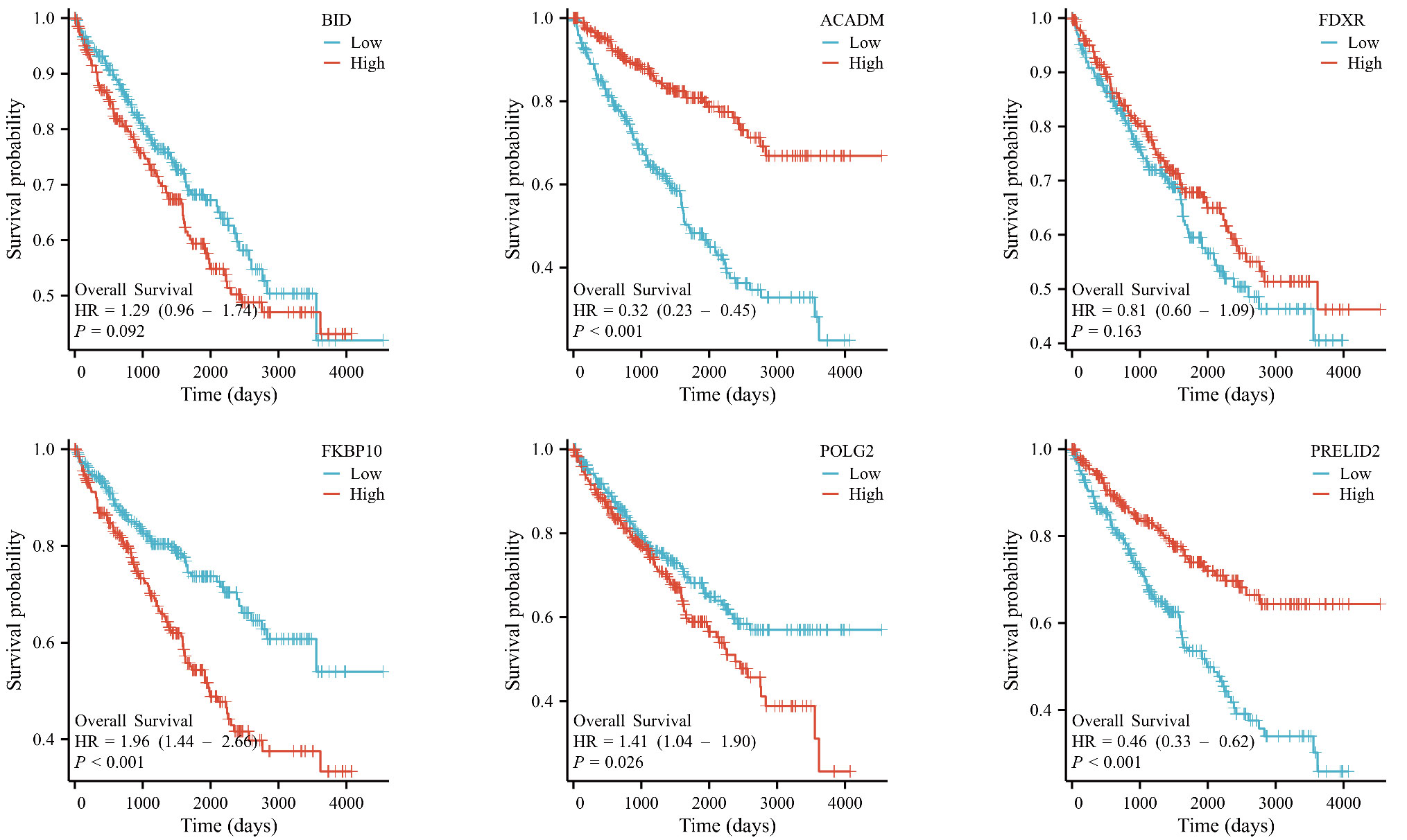

Supplement: Supplementary file 5 — Supplementary file5 (JPG 323 KB) [file 10238_2024_1426_MOESM5_ESM.jpg]
