# Supplementary material for: Exploring necrosis-associated mitochondrial gene signatures: revealing their role in prognosis and immunotherapy of renal clear cell carcinoma
Source: Clin Exp Med. 2024 Jul 18;24(1):161. doi: 10.1007/s10238-024-01426-9 (PMC11258092; doi:10.1007/s10238-024-01426-9)
Supplement: Supplementary file 6 — Supplementary file6 (DOCX 489 KB) [file 10238_2024_1426_MOESM6_ESM.docx]

| Gene | logFC | AveExpr | t | P.Value | adj.P.Val | B |
| --- | --- | --- | --- | --- | --- | --- |
| SLC9A4 | -4.603742372 | 1.396901043 | -54.96610482 | 1.14E-239 | 1.80E-235 | 538.1913489 |
| AQP2 | -9.15008604 | 2.595444543 | -54.78610055 | 6.10E-239 | 4.80E-235 | 536.5212276 |
| HS6ST2 | -4.113845327 | 1.69640532 | -52.30606539 | 9.69E-229 | 5.09E-225 | 513.1326466 |
| ATP12A | -3.20789883 | 1.167318886 | -51.17030493 | 5.77E-224 | 2.27E-220 | 502.1820187 |
| GPC5 | -2.924620093 | 2.933481012 | -51.03516696 | 2.16E-223 | 6.79E-220 | 500.868881 |
| MUC15 | -4.931184777 | 1.160379793 | -50.58369147 | 1.79E-221 | 4.70E-218 | 496.4660753 |
| DUSP9 | -6.137992188 | 2.315895821 | -49.08343254 | 5.10E-215 | 1.15E-211 | 481.659284 |
| KNG1 | -7.250549144 | 2.376984087 | -48.36983059 | 6.59E-212 | 1.30E-208 | 474.5204089 |
| KCNJ10 | -4.741551256 | 2.692532467 | -47.62622781 | 1.23E-208 | 2.15E-205 | 467.0149242 |
| GP2 | -2.793777637 | 0.819085045 | -47.00569911 | 6.95E-206 | 1.09E-202 | 460.6994228 |
| SLC12A1 | -7.532077457 | 2.365799668 | -46.67998905 | 1.97E-204 | 2.82E-201 | 457.3653597 |
| ESRRB | -3.868298063 | 2.029139021 | -46.51343369 | 1.10E-203 | 1.44E-200 | 455.6553523 |
| HRG | -4.132703825 | 1.049291357 | -46.14618419 | 4.87E-202 | 5.90E-199 | 451.8726236 |
| TFAP2B | -3.729267298 | 1.03093753 | -46.08585873 | 9.10E-202 | 1.02E-198 | 451.2496526 |
| UMOD | -10.5347125 | 3.401041604 | -45.69572316 | 5.24E-200 | 5.50E-197 | 447.2098048 |
| TRPV6 | -1.843640524 | 1.505951879 | -45.41849746 | 9.44E-199 | 9.29E-196 | 444.3275595 |
| RALYL | -3.208849399 | 2.518468446 | -45.07375378 | 3.48E-197 | 3.23E-194 | 440.7299052 |
| CA10 | -3.381282557 | 1.207476452 | -43.83167206 | 1.75E-191 | 1.53E-188 | 427.643982 |
| ELF5 | -3.582710904 | 1.833896556 | -43.7021426 | 6.94E-191 | 5.75E-188 | 426.2681391 |
| NELL1 | -3.837814192 | 2.162846648 | -43.25281865 | 8.46E-189 | 6.66E-186 | 421.4790851 |
| IRX2 | -3.542163601 | 2.378420115 | -43.0906461 | 4.82E-188 | 3.61E-185 | 419.7443373 |
| FXYD4 | -7.710309914 | 1.838654645 | -43.04155046 | 8.17E-188 | 5.85E-185 | 419.2185106 |
| NRK | -3.563551103 | 1.259612448 | -41.85812989 | 2.96E-182 | 2.03E-179 | 406.4517774 |
| CASR | -4.160459322 | 1.318743025 | -41.35222822 | 7.45E-180 | 4.89E-177 | 400.9402606 |
| KCNJ1 | -6.930643247 | 2.119550009 | -40.52399223 | 6.78E-176 | 4.27E-173 | 391.8476635 |
| PIK3C2G | -2.959168501 | 0.841317588 | -40.51088015 | 7.84E-176 | 4.75E-173 | 391.703024 |
| CLDN16 | -4.919946615 | 1.677486136 | -39.3203426 | 4.48E-170 | 2.61E-167 | 378.4808324 |
| ACOT12 | -1.686988488 | 2.211221684 | -38.97109017 | 2.26E-168 | 1.27E-165 | 374.5686408 |
| TYRP1 | -3.070919803 | 1.557674 | -38.49891961 | 4.65E-166 | 2.52E-163 | 369.2556885 |
| OVCH2 | -2.892133392 | 0.961259807 | -38.44645605 | 8.41E-166 | 4.41E-163 | 368.663671 |
| IRX1 | -3.949727207 | 1.335959667 | -37.94340998 | 2.53E-163 | 1.29E-160 | 362.9700747 |
| KCTD8 | -1.88111271 | 0.69644968 | -37.56288314 | 1.94E-161 | 9.53E-159 | 358.6427835 |
| PTGER1 | -3.660788167 | 1.208823897 | -37.42565404 | 9.29E-161 | 4.44E-158 | 357.0779528 |
| COL4A6 | -3.213403981 | 1.181191961 | -37.34592329 | 2.31E-160 | 1.07E-157 | 356.1677401 |
| RANBP3L | -4.612848429 | 1.784376489 | -36.82977225 | 8.66E-158 | 3.89E-155 | 350.2569504 |
| SIM2 | -2.514277535 | 1.835888369 | -36.28649826 | 4.57E-155 | 2.00E-152 | 344.0014917 |
| TPPP2 | -1.155521076 | 0.720337333 | -35.71595869 | 3.43E-152 | 1.46E-149 | 337.3950114 |
| RASL11B | -1.571849875 | 0.705604786 | -35.24195287 | 8.64E-150 | 3.58E-147 | 331.8779007 |
| FAM3B | -3.590150488 | 1.602752553 | -35.18001464 | 1.78E-149 | 7.20E-147 | 331.1550984 |
| CLDN8 | -6.359784004 | 1.64728296 | -34.74871626 | 2.80E-147 | 1.10E-144 | 326.1100399 |
| GGT6 | -5.073252184 | 2.735172789 | -34.57966874 | 2.04E-146 | 7.84E-144 | 324.1269881 |
| NAT8L | -3.85914912 | 2.124456781 | -34.54505579 | 3.07E-146 | 1.15E-143 | 323.7205645 |
| CLDN19 | -4.833661744 | 2.116123256 | -34.28145913 | 6.85E-145 | 2.51E-142 | 320.6211225 |
| CLUL1 | -1.474825305 | 1.679410504 | -34.25053514 | 9.87E-145 | 3.53E-142 | 320.2570138 |
| SCNN1G | -5.885940628 | 2.440315439 | -34.17223055 | 2.49E-144 | 8.70E-142 | 319.3345677 |
| MCCD1 | -4.813467212 | 1.587243559 | -33.19270037 | 2.76E-139 | 9.45E-137 | 307.740349 |
| NHLRC4 | -2.492319473 | 1.734446748 | -32.75907195 | 4.88E-137 | 1.64E-134 | 302.5759553 |
| EHF | -4.520833364 | 2.483901536 | -32.73430341 | 6.56E-137 | 2.15E-134 | 302.280397 |
| GABRA2 | -2.26381243 | 1.529326936 | -32.66568809 | 1.49E-136 | 4.79E-134 | 301.4613044 |
| SPAG4 | 3.577155479 | 5.402319366 | 32.55370666 | 5.70E-136 | 1.79E-133 | 300.1235277 |
| ADH1C | -4.080498598 | 2.148060335 | -32.3625987 | 5.63E-135 | 1.74E-132 | 297.837625 |
| SFRP1 | -5.625930507 | 4.133221333 | -32.3461255 | 6.86E-135 | 2.08E-132 | 297.640417 |
| CALB1 | -4.791746039 | 1.228333857 | -32.30923508 | 1.07E-134 | 3.17E-132 | 297.1986898 |
| TNNI1 | -2.094488992 | 2.37546909 | -32.19530487 | 4.19E-134 | 1.22E-131 | 295.8336551 |
| CLCNKA | -5.454894931 | 1.962799693 | -32.1344128 | 8.71E-134 | 2.49E-131 | 295.1035758 |
| BSND | -3.887495861 | 1.687134587 | -32.04402671 | 2.58E-133 | 7.26E-131 | 294.019218 |
| EGF | -5.204099101 | 3.336322273 | -31.60284182 | 5.25E-131 | 1.45E-128 | 288.7153141 |
| SLC2A12 | -2.376713771 | 1.896651928 | -31.52456502 | 1.35E-130 | 3.67E-128 | 287.7723932 |
| SERPINA5 | -5.72415887 | 2.991414859 | -31.41191084 | 5.26E-130 | 1.40E-127 | 286.4143857 |
| SCNN1B | -4.697714173 | 1.816763386 | -31.10807235 | 2.08E-128 | 5.45E-126 | 282.7460515 |
| FRMD7 | -1.912625328 | 1.956654352 | -31.02132575 | 5.94E-128 | 1.53E-125 | 281.6972403 |
| MAPK4 | -2.536273507 | 0.87391122 | -31.00718119 | 7.05E-128 | 1.79E-125 | 281.5261631 |
| FLRT1 | -2.150419837 | 1.147625183 | -30.9787846 | 9.95E-128 | 2.49E-125 | 281.1826567 |
| EPN3 | -2.936258547 | 1.76722551 | -30.82353836 | 6.54E-127 | 1.61E-124 | 279.3034553 |
| PRR15 | -3.59185804 | 2.373394869 | -30.77509747 | 1.18E-126 | 2.85E-124 | 278.716674 |
| PAK6 | -2.334742611 | 1.514033541 | -30.60845136 | 8.91E-126 | 2.13E-123 | 276.6965216 |
| TMPRSS2 | -4.618235801 | 1.733375978 | -30.56003048 | 1.60E-125 | 3.77E-123 | 276.1091093 |
| CA9 | 6.839121576 | 7.249738162 | 30.52304643 | 2.52E-125 | 5.83E-123 | 275.6603109 |
| PRDM16 | -2.702540389 | 2.909552958 | -30.45319718 | 5.88E-125 | 1.34E-122 | 274.8123891 |
| TNNC1 | -2.889126726 | 2.74364586 | -30.44706201 | 6.34E-125 | 1.43E-122 | 274.7378933 |
| KLRG2 | -2.89508467 | 1.885685357 | -30.4251851 | 8.27E-125 | 1.83E-122 | 274.4722297 |
| F11 | -3.392150399 | 1.860732415 | -29.96527315 | 2.25E-122 | 4.91E-120 | 268.8783954 |
| EPB41L4B | -2.790567984 | 1.835533676 | -29.91319149 | 4.24E-122 | 9.15E-120 | 268.2438912 |
| TMEM213 | -6.149287154 | 1.760465626 | -29.73832394 | 3.59E-121 | 7.64E-119 | 266.1119944 |
| WNK4 | -3.477693884 | 3.120524559 | -29.63741449 | 1.23E-120 | 2.59E-118 | 264.8807162 |
| CLCNKB | -6.116075454 | 2.986452111 | -29.6163636 | 1.60E-120 | 3.31E-118 | 264.6237625 |
| SPTBN2 | -4.473536776 | 2.0551829 | -29.1703374 | 3.76E-118 | 7.69E-116 | 259.1719524 |
| GAL3ST3 | -2.080672458 | 1.439857345 | -29.15774746 | 4.39E-118 | 8.85E-116 | 259.0178626 |
| NOS1 | -1.728824428 | 2.15895619 | -29.12109334 | 6.87E-118 | 1.37E-115 | 258.5691868 |
| ERBB4 | -3.035832796 | 1.871486067 | -29.07379778 | 1.23E-117 | 2.42E-115 | 257.9901167 |
| SOST | -3.238692422 | 2.592086424 | -28.91725964 | 8.39E-117 | 1.63E-114 | 256.0724525 |
| SLC4A9 | -2.919302067 | 0.961159582 | -28.79469476 | 3.78E-116 | 7.26E-114 | 254.5698546 |
| NR0B2 | -4.165113129 | 1.488703768 | -28.7693746 | 5.16E-116 | 9.79E-114 | 254.2593186 |
| KLHL14 | -2.603366778 | 2.607693389 | -28.76355852 | 5.54E-116 | 1.04E-113 | 254.1879823 |
| SLC4A1 | -5.582105716 | 2.754544679 | -28.76039747 | 5.76E-116 | 1.07E-113 | 254.14921 |
| ITLN1 | -2.084141484 | 2.056243432 | -28.61944494 | 3.26E-115 | 5.97E-113 | 252.4196952 |
| SLC12A3 | -5.323212516 | 2.818979534 | -28.53941304 | 8.72E-115 | 1.58E-112 | 251.437139 |
| SLC13A2 | -3.853859039 | 2.133373544 | -28.27698841 | 2.21E-113 | 3.95E-111 | 248.2126357 |
| GRHL2 | -3.026363409 | 2.11509421 | -28.20077453 | 5.64E-113 | 9.98E-111 | 247.2754161 |
| MFSD6L | -2.353411786 | 0.957032998 | -28.18585855 | 6.78E-113 | 1.19E-110 | 247.0919522 |
| NOS1AP | -1.154606984 | 0.909284181 | -28.13145754 | 1.33E-112 | 2.29E-110 | 246.4227235 |
| RBP2 | -1.903590044 | 1.356842957 | -28.04833693 | 3.69E-112 | 6.32E-110 | 245.3998756 |
| LRRC2 | -2.552640759 | 2.179502074 | -27.99795684 | 6.87E-112 | 1.16E-109 | 244.7797351 |
| FGF9 | -4.007601502 | 1.513140806 | -27.8462522 | 4.47E-111 | 7.48E-109 | 242.9115473 |
| FOLR3 | -2.728754416 | 2.01056079 | -27.82862928 | 5.55E-111 | 9.20E-109 | 242.6944491 |
| MYO3B | -3.164074187 | 1.889449206 | -27.82784411 | 5.61E-111 | 9.20E-109 | 242.6847762 |
| PCDH9 | -1.384259185 | 2.085369918 | -27.70819381 | 2.46E-110 | 3.99E-108 | 241.21037 |
| CHL1 | -3.031264737 | 1.840364574 | -27.68628533 | 3.22E-110 | 5.17E-108 | 240.9403219 |
| TMEM45B | -3.632357793 | 2.258758327 | -27.62237229 | 7.09E-110 | 1.13E-107 | 240.1523818 |
| FAM167A | -3.007849498 | 1.952806245 | -27.60177079 | 9.14E-110 | 1.44E-107 | 239.8983574 |
| CHGB | -3.1915848 | 2.372187572 | -27.55783042 | 1.57E-109 | 2.45E-107 | 239.3564873 |
| PLA2G4F | -3.509438076 | 1.104547207 | -27.4308926 | 7.55E-109 | 1.17E-106 | 237.7905831 |
| NDUFA4L2 | 6.085764556 | 9.612723765 | 27.26672849 | 5.75E-108 | 8.79E-106 | 235.7643609 |
| AQP6 | -4.22138116 | 2.018834041 | -27.24069771 | 7.94E-108 | 1.20E-105 | 235.4429622 |
| TMPRSS4 | -2.760006812 | 1.549673253 | -27.22269109 | 9.92E-108 | 1.49E-105 | 235.2206196 |
| CEL | -2.59604586 | 2.566977904 | -27.09200387 | 5.00E-107 | 7.42E-105 | 233.6065049 |
| TREM2 | 3.714062602 | 5.480807833 | 27.05817822 | 7.60E-107 | 1.12E-104 | 233.1886098 |
| DDN | -2.796177697 | 1.312534966 | -27.0510602 | 8.30E-107 | 1.21E-104 | 233.1006651 |
| MRAP2 | -2.900931306 | 2.754114841 | -27.0379282 | 9.76E-107 | 1.41E-104 | 232.938411 |
| PROM2 | -4.462079719 | 3.110720756 | -26.9723612 | 2.20E-106 | 3.15E-104 | 232.1281867 |
| VTCN1 | -4.21874873 | 2.80235597 | -26.8359085 | 1.19E-105 | 1.69E-103 | 230.4414793 |
| KLK6 | -2.524014148 | 1.543681548 | -26.82291017 | 1.40E-105 | 1.97E-103 | 230.280769 |
| ATP6V0A4 | -5.753601135 | 1.80948714 | -26.81498975 | 1.54E-105 | 2.15E-103 | 230.1828383 |
| TCF21 | -2.83356619 | 3.362640608 | -26.80462487 | 1.75E-105 | 2.42E-103 | 230.0546802 |
| FAM81A | -2.018248013 | 2.071855447 | -26.78376696 | 2.27E-105 | 3.11E-103 | 229.7967675 |
| FAM83B | -2.38099226 | 1.168815462 | -26.7506772 | 3.42E-105 | 4.65E-103 | 229.3875731 |
| USP44 | -1.201006954 | 1.68692485 | -26.70295604 | 6.18E-105 | 8.32E-103 | 228.7973752 |
| PRRG2 | -2.950408684 | 2.813766801 | -26.60545039 | 2.07E-104 | 2.76E-102 | 227.5912154 |
| SLC15A2 | -2.801945293 | 3.031716012 | -26.57761079 | 2.92E-104 | 3.87E-102 | 227.2467764 |
| OVOL2 | -1.995994692 | 0.850618639 | -26.57063587 | 3.19E-104 | 4.18E-102 | 227.1604767 |
| BMP7 | -2.677836301 | 2.061120797 | -26.53298152 | 5.08E-104 | 6.62E-102 | 226.694558 |
| SYT7 | -2.884201671 | 1.572664025 | -26.5241892 | 5.67E-104 | 7.32E-102 | 226.5857589 |
| SLC26A7 | -4.259279837 | 1.975402098 | -26.51827143 | 6.10E-104 | 7.81E-102 | 226.5125292 |
| SLC9A2 | -2.869653511 | 1.163956915 | -26.48804922 | 8.87E-104 | 1.13E-101 | 226.1385252 |
| FAM169A | -2.252795163 | 1.671612188 | -26.46444216 | 1.19E-103 | 1.50E-101 | 225.8463646 |
| TAGLN3 | -2.529619368 | 1.629525406 | -26.34932872 | 4.96E-103 | 6.20E-101 | 224.4214785 |
| NPHS2 | -4.400966645 | 1.926568954 | -26.30059571 | 9.07E-103 | 1.13E-100 | 223.8181383 |
| GCGR | -3.2392715 | 1.620236161 | -26.18288577 | 3.91E-102 | 4.81E-100 | 222.3605534 |
| ENPP6 | -2.599954888 | 2.330929971 | -26.17108726 | 4.52E-102 | 5.52E-100 | 222.2144337 |
| KSR2 | -1.365629852 | 1.590972402 | -26.15804042 | 5.32E-102 | 6.44E-100 | 222.0528495 |
| HSPA2 | -3.365100735 | 3.611612748 | -26.11925295 | 8.61E-102 | 1.03E-99 | 221.5724436 |
| AGBL4 | -1.740442549 | 2.155571702 | -26.11880334 | 8.66E-102 | 1.03E-99 | 221.5668747 |
| NOL3 | 3.035806512 | 6.808225994 | 26.02158787 | 2.89E-101 | 3.43E-99 | 220.3626375 |
| GADL1 | -1.067709543 | 1.727933473 | -26.01624136 | 3.09E-101 | 3.63E-99 | 220.296402 |
| SLC4A11 | -2.866450451 | 2.408981794 | -25.98663386 | 4.46E-101 | 5.21E-99 | 219.9295963 |
| GPC3 | -4.055600044 | 4.153145572 | -25.97720415 | 5.02E-101 | 5.81E-99 | 219.8127679 |
| LHX1 | -2.668825283 | 1.876942875 | -25.94207579 | 7.76E-101 | 8.92E-99 | 219.377531 |
| RHCG | -5.869572388 | 2.651834601 | -25.91407857 | 1.10E-100 | 1.25E-98 | 219.0306284 |
| C1orf226 | -2.344514745 | 3.924733408 | -25.75221204 | 8.20E-100 | 9.29E-98 | 217.0246826 |
| WNT9B | -2.133635248 | 1.487479182 | -25.71627557 | 1.28E-99 | 1.44E-97 | 216.579267 |
| ALDH3B2 | -1.224102602 | 1.412915802 | -25.61712017 | 4.39E-99 | 4.90E-97 | 215.3501633 |
| PROX1 | -2.669346855 | 2.043861266 | -25.59588959 | 5.71E-99 | 6.34E-97 | 215.0869731 |
| PAPPA | -2.459354243 | 2.502277035 | -25.58297247 | 6.71E-99 | 7.39E-97 | 214.9268393 |
| KLK7 | -2.419946472 | 0.793743784 | -25.46059868 | 3.07E-98 | 3.35E-96 | 213.409644 |
| CNTN1 | -2.923285767 | 2.386920719 | -25.45754633 | 3.19E-98 | 3.46E-96 | 213.3717981 |
| OXGR1 | -2.503920332 | 0.971033778 | -25.44543859 | 3.70E-98 | 3.99E-96 | 213.2216739 |
| LMO3 | -1.655222248 | 2.695086785 | -25.20764381 | 7.11E-97 | 7.56E-95 | 210.2728974 |
| SUSD4 | -3.230368564 | 2.888574812 | -25.08889866 | 3.11E-96 | 3.28E-94 | 208.8001968 |
| PPP1R1B | -1.796394865 | 1.541436876 | -25.04123884 | 5.62E-96 | 5.90E-94 | 208.2090832 |
| STC2 | 4.064916227 | 6.247543028 | 25.03363944 | 6.18E-96 | 6.44E-94 | 208.1148283 |
| FREM1 | -2.951015791 | 2.180413559 | -25.02008936 | 7.31E-96 | 7.57E-94 | 207.9467671 |
| RAB25 | -4.632895706 | 2.948818262 | -24.89033722 | 3.67E-95 | 3.77E-93 | 206.3374137 |
| DMRT2 | -4.009337664 | 1.246190657 | -24.88492387 | 3.92E-95 | 4.01E-93 | 206.2702692 |
| NT5C1A | -1.01220664 | 1.29900014 | -24.82555411 | 8.20E-95 | 8.33E-93 | 205.5338719 |
| CCNI2 | -1.371303961 | 1.933509008 | -24.79152352 | 1.25E-94 | 1.26E-92 | 205.1117687 |
| MPP7 | -2.568297319 | 2.374921521 | -24.76951329 | 1.65E-94 | 1.65E-92 | 204.8387614 |
| CYP2B6 | -2.681311167 | 1.897298031 | -24.69727455 | 4.04E-94 | 4.02E-92 | 203.9427379 |
| GATA3 | -3.457078188 | 3.072392579 | -24.56515056 | 2.09E-93 | 2.05E-91 | 202.303944 |
| RNF150 | -2.814224818 | 3.089196831 | -24.50396987 | 4.46E-93 | 4.36E-91 | 201.5451139 |
| MYLK3 | -1.237614745 | 1.554965713 | -24.46264045 | 7.46E-93 | 7.25E-91 | 201.0325121 |
| FER1L6 | -1.633704179 | 0.757181375 | -24.45556209 | 8.14E-93 | 7.87E-91 | 200.9447215 |
| WNT8B | -1.310585855 | 1.237878951 | -24.45282973 | 8.42E-93 | 8.09E-91 | 200.910833 |
| TFCP2L1 | -4.698248641 | 2.748606343 | -24.38165109 | 2.04E-92 | 1.95E-90 | 200.0280476 |
| TDGF1 | -3.279540187 | 1.845065805 | -24.37422982 | 2.24E-92 | 2.12E-90 | 199.9360083 |
| APOC1 | 4.14644546 | 5.629200344 | 24.36274672 | 2.58E-92 | 2.42E-90 | 199.7935947 |
| FOXI1 | -4.786143782 | 2.285429566 | -24.29874154 | 5.72E-92 | 5.33E-90 | 198.9998225 |
| TRPV5 | -1.808836674 | 0.827580027 | -24.26436188 | 8.76E-92 | 8.12E-90 | 198.573473 |
| INPP5J | -3.5676344 | 2.73198334 | -24.25659201 | 9.65E-92 | 8.86E-90 | 198.4771189 |
| SCARB1 | 3.472021789 | 6.301836497 | 24.25638554 | 9.68E-92 | 8.86E-90 | 198.4745584 |
| SIAH3 | -1.532997056 | 1.016264708 | -24.22020865 | 1.52E-91 | 1.38E-89 | 198.0259376 |
| NIPAL1 | -2.364866042 | 2.222838205 | -24.14940445 | 3.66E-91 | 3.31E-89 | 197.1479565 |
| ENTPD3 | -1.340742513 | 0.802159597 | -24.10306366 | 6.50E-91 | 5.85E-89 | 196.5733596 |
| GRM1 | -1.786492937 | 0.835084476 | -24.10003796 | 6.75E-91 | 6.04E-89 | 196.5358438 |
| S100A2 | -3.660091638 | 3.301215119 | -24.08326509 | 8.31E-91 | 7.40E-89 | 196.3278788 |
| TSPAN8 | -4.14675608 | 2.002467415 | -24.03558388 | 1.50E-90 | 1.33E-88 | 195.7367069 |
| ANKRD34B | -1.37681657 | 0.708918812 | -24.03400046 | 1.53E-90 | 1.35E-88 | 195.7170756 |
| CXCR4 | 2.881207705 | 8.383396623 | 24.02828968 | 1.65E-90 | 1.44E-88 | 195.6462734 |
| C9orf135 | -1.484147466 | 2.107627761 | -24.00798576 | 2.12E-90 | 1.84E-88 | 195.3945497 |
| TMEM61 | -2.582412664 | 2.105654053 | -23.96359828 | 3.68E-90 | 3.18E-88 | 194.8442666 |
| LIPH | -3.229651582 | 2.603546008 | -23.96108371 | 3.79E-90 | 3.26E-88 | 194.8130938 |
| SCN7A | -1.044289993 | 1.756520479 | -23.95073039 | 4.31E-90 | 3.69E-88 | 194.6847462 |
| CACNA2D2 | -1.518650902 | 1.893032788 | -23.94613542 | 4.57E-90 | 3.89E-88 | 194.6277841 |
| GRIK5 | -2.6120182 | 2.293669933 | -23.93188452 | 5.45E-90 | 4.62E-88 | 194.4511235 |
| MYBPH | -1.204046697 | 1.1797692 | -23.92388909 | 6.02E-90 | 5.07E-88 | 194.35201 |
| NPHS1 | -3.134704345 | 1.972312489 | -23.88483596 | 9.78E-90 | 8.19E-88 | 193.867914 |
| TJP3 | -2.671450904 | 2.630189575 | -23.86584517 | 1.24E-89 | 1.03E-87 | 193.6325177 |
| ANGPTL4 | 5.184455793 | 8.212089959 | 23.82617073 | 2.03E-89 | 1.68E-87 | 193.1407642 |
| LYPD6B | -3.075606137 | 1.336283099 | -23.71044518 | 8.53E-89 | 7.03E-87 | 191.7065657 |
| ATP6V1C2 | -2.869416585 | 2.179750726 | -23.69850815 | 9.89E-89 | 8.11E-87 | 191.5586458 |
| ATP6V0D2 | -4.807248829 | 3.968218036 | -23.65249482 | 1.75E-88 | 1.43E-86 | 190.9884939 |
| HEPACAM2 | -4.149663529 | 2.255496536 | -23.63325553 | 2.22E-88 | 1.80E-86 | 190.7501146 |
| ZNF488 | -1.29387066 | 2.242013457 | -23.59265367 | 3.68E-88 | 2.97E-86 | 190.2470783 |
| EYA4 | -2.097438766 | 1.267644047 | -23.49878653 | 1.18E-87 | 9.48E-86 | 189.0842764 |
| AMPH | -1.833657366 | 1.787481328 | -23.48249185 | 1.44E-87 | 1.15E-85 | 188.8824465 |
| PRSS22 | -2.215204385 | 1.878747925 | -23.46258161 | 1.85E-87 | 1.47E-85 | 188.6358436 |
| RASSF10 | -2.297035577 | 1.514328357 | -23.44912884 | 2.19E-87 | 1.73E-85 | 188.4692275 |
| GSTM3 | -3.056160428 | 5.468465792 | -23.40350614 | 3.85E-87 | 3.03E-85 | 187.904218 |
| DDB2 | 1.826571307 | 6.057307702 | 23.40107582 | 3.97E-87 | 3.11E-85 | 187.8741217 |
| SCN2A | -1.228570611 | 0.675619749 | -23.30824295 | 1.26E-86 | 9.79E-85 | 186.7246454 |
| MAP3K15 | -1.747260329 | 1.775613273 | -23.30708478 | 1.27E-86 | 9.88E-85 | 186.7103064 |
| CHP2 | -1.310685363 | 1.515385955 | -23.29407446 | 1.50E-86 | 1.16E-84 | 186.5492322 |
| ESRP1 | -3.479447666 | 1.585903943 | -23.25187089 | 2.53E-86 | 1.94E-84 | 186.0267693 |
| CWH43 | -3.065267456 | 2.503534962 | -23.10534767 | 1.56E-85 | 1.18E-83 | 184.2133495 |
| ESRRG | -2.905564355 | 2.913263667 | -23.05624152 | 2.86E-85 | 2.16E-83 | 183.6057708 |
| TMEM30B | -3.374061952 | 2.373829161 | -23.0519187 | 3.02E-85 | 2.27E-83 | 183.5522899 |
| L1CAM | -4.05145654 | 2.954516416 | -23.03749067 | 3.61E-85 | 2.69E-83 | 183.3737952 |
| PPP2R2B | -1.134972839 | 1.033417668 | -23.03743728 | 3.61E-85 | 2.69E-83 | 183.3731348 |
| ODF3B | 2.922697188 | 4.956459342 | 23.01015414 | 5.06E-85 | 3.76E-83 | 183.0356269 |
| C10orf82 | -1.057272917 | 0.64210191 | -23.00684697 | 5.27E-85 | 3.90E-83 | 182.9947173 |
| AKAP3 | -1.426350893 | 2.897290187 | -22.993645 | 6.21E-85 | 4.57E-83 | 182.8314137 |
| AVPR2 | -2.663329777 | 3.172970949 | -22.992845 | 6.27E-85 | 4.59E-83 | 182.8215182 |
| NTNG1 | -2.074652922 | 1.674318355 | -22.97198563 | 8.12E-85 | 5.92E-83 | 182.5635104 |
| RHBG | -3.307773765 | 1.577071602 | -22.8402688 | 4.16E-84 | 3.02E-82 | 180.9347326 |
| RIMBP2 | -1.244610498 | 1.929098738 | -22.77244294 | 9.63E-84 | 6.95E-82 | 180.0963083 |
| SLC6A17 | -1.508241624 | 1.370464531 | -22.62359138 | 6.08E-83 | 4.37E-81 | 178.2570377 |
| SRGAP3 | -1.539915089 | 1.336955466 | -22.59762767 | 8.39E-83 | 6.00E-81 | 177.9363293 |
| VIM | 2.618755601 | 9.52582184 | 22.59404936 | 8.77E-83 | 6.25E-81 | 177.892132 |
| PAPPA2 | -2.971368833 | 2.144466876 | -22.45428317 | 4.94E-82 | 3.51E-80 | 176.1663334 |
| COL23A1 | 4.740086627 | 6.800676805 | 22.4536649 | 4.98E-82 | 3.52E-80 | 176.1587014 |
| C15orf56 | -1.68378468 | 1.290176092 | -22.36798072 | 1.44E-81 | 1.01E-79 | 175.1012133 |
| FGF1 | -3.161124793 | 3.193798794 | -22.36582424 | 1.48E-81 | 1.03E-79 | 175.0746039 |
| DUSP26 | -1.768529353 | 1.581827211 | -22.26758969 | 4.97E-81 | 3.43E-79 | 173.8627438 |
| REEP6 | -2.433665597 | 3.37988885 | -22.26317764 | 5.25E-81 | 3.61E-79 | 173.8083281 |
| SPTB | -1.183651718 | 1.860082183 | -22.2375236 | 7.21E-81 | 4.93E-79 | 173.4919491 |
| CA8 | -2.343665881 | 2.916516827 | -22.15732544 | 1.94E-80 | 1.32E-78 | 172.503159 |
| SLC16A3 | 2.771764968 | 5.207108727 | 22.14886024 | 2.16E-80 | 1.46E-78 | 172.3988116 |
| TYMP | 2.770058302 | 5.898040957 | 22.13229336 | 2.65E-80 | 1.79E-78 | 172.1946109 |
| EMX1 | -2.368325789 | 3.751293762 | -22.09128346 | 4.39E-80 | 2.95E-78 | 171.6892033 |
| HLA-F | 2.392394896 | 6.80084614 | 22.08591811 | 4.69E-80 | 3.14E-78 | 171.6230884 |
| IYD | -2.182394116 | 1.992382638 | -22.04983945 | 7.32E-80 | 4.89E-78 | 171.1785548 |
| TTC36 | -1.245082955 | 2.409908683 | -22.0471342 | 7.57E-80 | 5.03E-78 | 171.1452261 |
| ASB15 | -1.836328634 | 1.46908914 | -22.04143114 | 8.12E-80 | 5.38E-78 | 171.074966 |
| C16orf89 | -3.47309009 | 2.281004915 | -22.0335726 | 8.95E-80 | 5.90E-78 | 170.9781545 |
| SLC4A8 | -1.980635873 | 2.073967005 | -21.99215112 | 1.49E-79 | 9.79E-78 | 170.4679391 |
| ARRB2 | 1.863030624 | 5.600147812 | 21.99101848 | 1.51E-79 | 9.89E-78 | 170.4539892 |
| CRHBP | -3.130446474 | 2.096430555 | -21.98975306 | 1.54E-79 | 1.00E-77 | 170.4384042 |
| TTPA | -1.158953138 | 1.683288027 | -21.92167001 | 3.56E-79 | 2.30E-77 | 169.6000404 |
| KCNK13 | -2.217431136 | 2.133571972 | -21.88002213 | 5.95E-79 | 3.83E-77 | 169.0873509 |
| RPS6KA6 | -2.574648518 | 1.724716977 | -21.7877643 | 1.86E-78 | 1.19E-76 | 167.9520806 |
| EGLN3 | 3.89287538 | 7.587862416 | 21.73271342 | 3.66E-78 | 2.33E-76 | 167.2749485 |
| NNMT | 4.262737053 | 9.180088164 | 21.70125815 | 5.40E-78 | 3.43E-76 | 166.8881451 |
| HLA-B | 2.012842831 | 10.0782653 | 21.69958954 | 5.51E-78 | 3.48E-76 | 166.8676284 |
| HOXB9 | -3.690256002 | 2.518399454 | -21.68749319 | 6.39E-78 | 4.03E-76 | 166.7189015 |
| MAP6 | -1.878858763 | 2.832478151 | -21.63899661 | 1.16E-77 | 7.29E-76 | 166.1227379 |
| FABP6 | 4.293824647 | 4.772085012 | 21.63337104 | 1.25E-77 | 7.78E-76 | 166.0535948 |
| ANKRD2 | -3.271641008 | 2.694067341 | -21.61786388 | 1.51E-77 | 9.38E-76 | 165.8630114 |
| LILRB1 | 2.150246308 | 2.942936656 | 21.58933596 | 2.14E-77 | 1.33E-75 | 165.5124509 |
| MPPED2 | -1.98121837 | 2.298374875 | -21.57909406 | 2.43E-77 | 1.50E-75 | 165.3866104 |
| DNER | -3.449304936 | 1.669591624 | -21.57339027 | 2.61E-77 | 1.60E-75 | 165.3165325 |
| HSF4 | 3.998277931 | 6.36414235 | 21.56885721 | 2.76E-77 | 1.69E-75 | 165.2608402 |
| RIPPLY1 | -1.374668469 | 1.778339001 | -21.55441676 | 3.29E-77 | 2.01E-75 | 165.0834386 |
| RPRM | -1.71601233 | 1.380187278 | -21.55055454 | 3.45E-77 | 2.10E-75 | 165.0359937 |
| CPAMD8 | -2.617554664 | 2.424179311 | -21.53862682 | 4.00E-77 | 2.42E-75 | 164.889477 |
| MCF2L-AS1 | -1.918217904 | 1.355823348 | -21.53744135 | 4.06E-77 | 2.45E-75 | 164.8749158 |
| FOXJ1 | -2.287406259 | 1.254893691 | -21.52433445 | 4.77E-77 | 2.87E-75 | 164.7139286 |
| SAP30 | 2.229436425 | 6.136592497 | 21.52041912 | 5.00E-77 | 3.00E-75 | 164.6658408 |
| TYROBP | 2.761746892 | 6.897181675 | 21.51485701 | 5.36E-77 | 3.20E-75 | 164.5975293 |
| PCP4 | -4.122843921 | 3.403068467 | -21.50533868 | 6.03E-77 | 3.58E-75 | 164.4806352 |
| CLDN14 | -2.165648548 | 2.476568526 | -21.46790655 | 9.55E-77 | 5.65E-75 | 164.0210036 |
| RAB11FIP4 | -2.051353341 | 2.527761217 | -21.44260443 | 1.30E-76 | 7.69E-75 | 163.7103817 |
| ITGAX | 2.706345384 | 3.971691279 | 21.40027289 | 2.19E-76 | 1.29E-74 | 163.1908159 |
| ATP1A1 | -2.922910992 | 6.535798434 | -21.36788577 | 3.27E-76 | 1.91E-74 | 162.793406 |
| SCD | 3.125404231 | 7.396844152 | 21.36739299 | 3.29E-76 | 1.92E-74 | 162.78736 |
| UPP2 | -2.497334159 | 2.521763866 | -21.33730787 | 4.76E-76 | 2.77E-74 | 162.4182776 |
| CDCA2 | 3.205395617 | 3.996480229 | 21.33126567 | 5.13E-76 | 2.97E-74 | 162.3441617 |
| FCGR3A | 3.327353705 | 6.20139866 | 21.32663717 | 5.43E-76 | 3.13E-74 | 162.2873887 |
| FRG2C | -1.657620002 | 1.203537078 | -21.32383371 | 5.62E-76 | 3.23E-74 | 162.2530025 |
| HK2 | 3.112161443 | 5.87284302 | 21.3003484 | 7.50E-76 | 4.29E-74 | 161.964967 |
| NXPH2 | -2.081088184 | 1.233486879 | -21.29935954 | 7.59E-76 | 4.33E-74 | 161.9528402 |
| CALCA | -2.879502525 | 1.722667684 | -21.28008456 | 9.62E-76 | 5.47E-74 | 161.7164796 |
| LMX1B | -1.625569462 | 1.890427649 | -21.27459663 | 1.03E-75 | 5.83E-74 | 161.6491894 |
| CAPSL | -1.061010684 | 2.17349587 | -21.25608082 | 1.29E-75 | 7.29E-74 | 161.4221775 |
| DIO1 | -3.404117123 | 1.96829916 | -21.25203736 | 1.36E-75 | 7.64E-74 | 161.3726069 |
| SLC47A2 | -2.506853294 | 1.46745276 | -21.24332421 | 1.51E-75 | 8.47E-74 | 161.2657933 |
| SLC22A8 | -3.716375182 | 2.137601383 | -21.23111794 | 1.76E-75 | 9.80E-74 | 161.1161692 |
| COBLL1 | -2.1852006 | 3.710474811 | -21.22221415 | 1.96E-75 | 1.09E-73 | 161.0070352 |
| NETO2 | 2.72768013 | 4.834045659 | 21.16561368 | 3.93E-75 | 2.18E-73 | 160.3134469 |
| DGCR5 | 2.640010937 | 3.833766452 | 21.13911545 | 5.43E-75 | 3.00E-73 | 159.988834 |
| DNASE1 | -2.258215554 | 1.993828983 | -21.10202484 | 8.57E-75 | 4.72E-73 | 159.5345684 |
| ABCA4 | -2.349131103 | 1.201365093 | -21.09925689 | 8.86E-75 | 4.86E-73 | 159.5006731 |
| PEG3 | -1.47204584 | 1.715243885 | -21.08957189 | 9.98E-75 | 5.46E-73 | 159.3820799 |
| HPCAL4 | -2.023880329 | 0.982405272 | -21.0688386 | 1.29E-74 | 7.02E-73 | 159.1282292 |
| SH3GL2 | -3.21142633 | 1.487356614 | -21.06241844 | 1.39E-74 | 7.56E-73 | 159.0496313 |
| SCNN1A | -4.158501482 | 4.090813625 | -21.04712965 | 1.68E-74 | 9.09E-73 | 158.8624759 |
| ADM | 2.69451593 | 8.021706592 | 21.04346603 | 1.76E-74 | 9.48E-73 | 158.8176315 |
| TCEAL2 | -2.735519187 | 1.346383054 | -21.03302012 | 2.00E-74 | 1.07E-72 | 158.6897758 |
| TDRD5 | -1.282391028 | 0.824383898 | -20.9989721 | 3.03E-74 | 1.62E-72 | 158.2731069 |
| CDKN2A | 2.02271151 | 2.96627385 | 20.96457258 | 4.63E-74 | 2.46E-72 | 157.8522491 |
| DOK3 | 1.927736118 | 4.030804121 | 20.94092491 | 6.18E-74 | 3.28E-72 | 157.5630001 |
| B4GALNT2 | -2.675220543 | 1.981620486 | -20.91115009 | 8.90E-74 | 4.71E-72 | 157.1988838 |
| LYPD6 | -1.629971411 | 2.112807149 | -20.90079571 | 1.01E-73 | 5.32E-72 | 157.0722804 |
| DPP6 | -1.643354915 | 2.146686298 | -20.86870323 | 1.50E-73 | 7.86E-72 | 156.6799519 |
| SLC7A13 | -2.563486915 | 1.801240023 | -20.86484743 | 1.57E-73 | 8.22E-72 | 156.6328219 |
| NCCRP1 | -2.55000958 | 1.313384218 | -20.84303463 | 2.05E-73 | 1.07E-71 | 156.3662287 |
| PLEKHD1 | -1.31224267 | 2.415316893 | -20.84303362 | 2.05E-73 | 1.07E-71 | 156.3662164 |
| PSMB8 | 1.643307798 | 6.339204119 | 20.84054689 | 2.11E-73 | 1.10E-71 | 156.3358269 |
| ESM1 | 3.570375115 | 7.968447991 | 20.82220527 | 2.65E-73 | 1.37E-71 | 156.1116996 |
| CASZ1 | -1.437383424 | 1.709000473 | -20.81722228 | 2.81E-73 | 1.45E-71 | 156.0508154 |
| SLC25A35 | -1.418337491 | 2.228300865 | -20.81363285 | 2.94E-73 | 1.51E-71 | 156.0069598 |
| BMPR1B | -3.004010627 | 1.473368 | -20.80129011 | 3.42E-73 | 1.75E-71 | 155.8561662 |
| OLFM4 | -2.38172525 | 1.815357072 | -20.79990915 | 3.48E-73 | 1.77E-71 | 155.8392957 |
| TUBB2B | -2.439554288 | 1.962352755 | -20.79627151 | 3.64E-73 | 1.85E-71 | 155.7948575 |
| CACNA2D3 | -1.448206156 | 1.53076261 | -20.75014218 | 6.40E-73 | 3.24E-71 | 155.2314486 |
| EFHD1 | -2.744001086 | 5.936983621 | -20.7322711 | 7.96E-73 | 4.02E-71 | 155.0132359 |
| MECOM | -2.485000227 | 5.097885549 | -20.7187842 | 9.39E-73 | 4.72E-71 | 154.8485777 |
| INSRR | -1.335956204 | 0.803287761 | -20.69678794 | 1.23E-72 | 6.16E-71 | 154.5800715 |
| SLC7A8 | -3.108861364 | 4.306051514 | -20.67504196 | 1.60E-72 | 8.02E-71 | 154.3146705 |
| ARHGAP22 | 1.509495198 | 2.895379768 | 20.67004488 | 1.70E-72 | 8.50E-71 | 154.2536901 |
| PLCL1 | -2.518116757 | 3.12917242 | -20.66657289 | 1.78E-72 | 8.84E-71 | 154.2113224 |
| SEMA6D | -2.383393977 | 2.347397638 | -20.65526217 | 2.04E-72 | 1.01E-70 | 154.0733094 |
| SLC16A5 | -2.623704493 | 4.177203908 | -20.65411736 | 2.07E-72 | 1.02E-70 | 154.0593413 |
| ARL4D | -2.904401952 | 3.489181365 | -20.64881919 | 2.21E-72 | 1.09E-70 | 153.9946985 |
| VEGFA | 3.143951601 | 8.476051868 | 20.61076973 | 3.52E-72 | 1.73E-70 | 153.5305471 |
| SUCLG1 | -1.67014633 | 5.652998362 | -20.60890729 | 3.60E-72 | 1.76E-70 | 153.5078319 |
| NKD1 | -1.222224234 | 2.112873146 | -20.59462004 | 4.29E-72 | 2.09E-70 | 153.33359 |
| PDE1A | -2.82751949 | 4.153041087 | -20.59136149 | 4.46E-72 | 2.17E-70 | 153.2938531 |
| NUDT10 | -1.968700527 | 1.295831525 | -20.58544856 | 4.80E-72 | 2.32E-70 | 153.2217498 |
| FECH | -1.694611889 | 4.187186676 | -20.53375724 | 9.02E-72 | 4.36E-70 | 152.5915795 |
| COL5A3 | 2.560598428 | 4.806677088 | 20.51806704 | 1.09E-71 | 5.26E-70 | 152.4003578 |
| ACSF2 | -2.367860719 | 4.336346927 | -20.51707395 | 1.11E-71 | 5.31E-70 | 152.3882557 |
| INHBB | 3.427207495 | 4.946679679 | 20.50043323 | 1.36E-71 | 6.49E-70 | 152.1854815 |
| C1orf116 | -2.92368944 | 2.241757903 | -20.4885128 | 1.57E-71 | 7.48E-70 | 152.040245 |
| RASAL3 | 2.194098387 | 4.471795013 | 20.47786083 | 1.79E-71 | 8.49E-70 | 151.9104765 |
| DIRAS1 | -2.205507719 | 1.892315515 | -20.47395131 | 1.87E-71 | 8.88E-70 | 151.8628517 |
| GABRD | 2.818605967 | 2.98376303 | 20.46195383 | 2.17E-71 | 1.03E-69 | 151.7167121 |
| XPNPEP2 | -3.886220449 | 3.587198446 | -20.44973927 | 2.52E-71 | 1.19E-69 | 151.5679448 |
| SSTR5 | -1.671401923 | 1.085407674 | -20.40433591 | 4.38E-71 | 2.06E-69 | 151.0151029 |
| B4GALNT3 | -2.307877423 | 2.550715978 | -20.31811976 | 1.25E-70 | 5.88E-69 | 149.9659673 |
| TMSB10 | 2.169339035 | 11.33088129 | 20.30198291 | 1.53E-70 | 7.13E-69 | 149.7696997 |
| TNFAIP6 | 4.607230312 | 5.931450743 | 20.27291218 | 2.18E-70 | 1.01E-68 | 149.4161987 |
| CNKSR1 | -2.134752922 | 1.388565163 | -20.2720113 | 2.20E-70 | 1.02E-68 | 149.4052456 |
| RHBDF2 | 1.704459622 | 4.770908617 | 20.26806599 | 2.31E-70 | 1.07E-68 | 149.3572786 |
| STAC3 | 1.571274685 | 3.97552249 | 20.24554857 | 3.04E-70 | 1.40E-68 | 149.0835481 |
| TRPV2 | 1.831471233 | 4.234017969 | 20.20908812 | 4.74E-70 | 2.18E-68 | 148.6404495 |
| MAN1C1 | -2.214091795 | 3.721060393 | -20.19785676 | 5.43E-70 | 2.49E-68 | 148.5039887 |
| FMN2 | -1.532091925 | 2.164703057 | -20.17651604 | 7.04E-70 | 3.22E-68 | 148.2447417 |
| ERMP1 | -2.148648262 | 5.424181533 | -20.12633767 | 1.30E-69 | 5.92E-68 | 147.6353945 |
| CAV2 | 1.932006209 | 7.158875468 | 20.10062425 | 1.77E-69 | 8.07E-68 | 147.323261 |
| CD300A | 2.615411666 | 4.014047879 | 20.09937029 | 1.80E-69 | 8.17E-68 | 147.3080412 |
| ENO2 | 3.026958995 | 5.928625414 | 20.08654102 | 2.10E-69 | 9.52E-68 | 147.1523404 |
| TACSTD2 | -4.256787996 | 5.477458809 | -20.07654011 | 2.38E-69 | 1.07E-67 | 147.0309798 |
| PRKAR2B | -1.95887936 | 3.029924634 | -20.02840387 | 4.27E-69 | 1.92E-67 | 146.4470248 |
| SOSTDC1 | -3.54715257 | 2.285374213 | -20.00702282 | 5.54E-69 | 2.48E-67 | 146.1877392 |
| PROZ | -1.932100587 | 2.117042358 | -19.99698377 | 6.25E-69 | 2.80E-67 | 146.0660169 |
| KLHL13 | -1.937109625 | 3.55728301 | -19.99219802 | 6.63E-69 | 2.96E-67 | 146.0079947 |
| SLC25A33 | -1.499201008 | 3.8539243 | -19.98584971 | 7.16E-69 | 3.18E-67 | 145.9310328 |
| ATP6V1B1 | -1.433495679 | 0.725993922 | -19.98190373 | 7.51E-69 | 3.33E-67 | 145.8831974 |
| ANGPTL1 | -3.073924236 | 2.806963633 | -19.96758455 | 8.94E-69 | 3.95E-67 | 145.7096292 |
| MYO1F | 2.061022076 | 3.486109461 | 19.95201911 | 1.08E-68 | 4.76E-67 | 145.5209845 |
| PVT1 | 1.395333275 | 3.026917114 | 19.92110985 | 1.57E-68 | 6.91E-67 | 145.1464744 |
| LAMA4 | 2.642194702 | 4.870888768 | 19.878882 | 2.63E-68 | 1.15E-66 | 144.6350254 |
| SLC5A2 | -2.039218327 | 1.44904259 | -19.86394611 | 3.15E-68 | 1.38E-66 | 144.4541834 |
| C3 | 4.256998301 | 8.238927789 | 19.85771197 | 3.39E-68 | 1.48E-66 | 144.3787099 |
| FCER1G | 2.432122551 | 7.076032738 | 19.85526156 | 3.50E-68 | 1.52E-66 | 144.3490456 |
| AP1M2 | -2.847722906 | 3.829292068 | -19.85112805 | 3.68E-68 | 1.59E-66 | 144.2990074 |
| LY86 | 2.334205126 | 4.85994863 | 19.84422396 | 4.00E-68 | 1.73E-66 | 144.2154353 |
| HLA-A | 1.628812425 | 11.36084372 | 19.79473836 | 7.29E-68 | 3.14E-66 | 143.6166124 |
| RNF43 | -1.370210556 | 1.300265101 | -19.78240741 | 8.46E-68 | 3.64E-66 | 143.4674473 |
| LGALS9 | 2.021661417 | 4.737491827 | 19.77939268 | 8.78E-68 | 3.77E-66 | 143.430982 |
| LGI2 | -2.106413135 | 2.924110569 | -19.73378741 | 1.53E-67 | 6.53E-66 | 142.8795027 |
| NPY5R | -1.490709386 | 1.704041339 | -19.73128222 | 1.57E-67 | 6.71E-66 | 142.8492171 |
| RALGPS1 | -1.553752277 | 2.630837216 | -19.67879579 | 2.97E-67 | 1.26E-65 | 142.2148994 |
| IL2RB | 2.51003956 | 3.801842644 | 19.65821286 | 3.81E-67 | 1.61E-65 | 141.9662516 |
| PARVG | 1.802737689 | 3.380705606 | 19.64892245 | 4.27E-67 | 1.80E-65 | 141.85404 |
| SHMT2 | 1.876710827 | 6.168849836 | 19.64207667 | 4.63E-67 | 1.95E-65 | 141.7713631 |
| ST8SIA4 | 2.755831361 | 5.001935239 | 19.6365254 | 4.96E-67 | 2.08E-65 | 141.7043248 |
| CD70 | 4.772736009 | 5.536270563 | 19.63642832 | 4.96E-67 | 2.08E-65 | 141.7031524 |
| HSD11B2 | -3.625514664 | 6.239474075 | -19.62068502 | 6.00E-67 | 2.51E-65 | 141.5130569 |
| FAM78A | 1.853572338 | 3.156609194 | 19.5810335 | 9.70E-67 | 4.04E-65 | 141.0344322 |
| PIK3R5 | 1.982669107 | 2.925114828 | 19.57586587 | 1.03E-66 | 4.29E-65 | 140.9720714 |
| STRA6 | -1.694087681 | 1.927682773 | -19.54181823 | 1.56E-66 | 6.44E-65 | 140.561294 |
| SLC36A2 | -3.154716166 | 1.888424298 | -19.53437638 | 1.71E-66 | 7.03E-65 | 140.4715318 |
| DCXR | -1.921081622 | 5.519395002 | -19.52021328 | 2.02E-66 | 8.32E-65 | 140.3007214 |
| SLC1A4 | 2.516121397 | 4.037906658 | 19.45594826 | 4.40E-66 | 1.80E-64 | 139.5260358 |
| PLEKHB1 | -2.301756928 | 2.455681422 | -19.45155006 | 4.64E-66 | 1.90E-64 | 139.4730396 |
| SHISA2 | -2.070452112 | 1.450070508 | -19.43694194 | 5.54E-66 | 2.25E-64 | 139.2970393 |
| SEMA5B | 3.254551683 | 5.910185892 | 19.43488189 | 5.68E-66 | 2.30E-64 | 139.272222 |
| RGS1 | 3.032021502 | 6.519401967 | 19.43240967 | 5.85E-66 | 2.37E-64 | 139.2424403 |
| LAPTM5 | 2.473603262 | 7.717520505 | 19.41291626 | 7.40E-66 | 2.99E-64 | 139.0076435 |
| IGSF6 | 2.24093302 | 4.480743249 | 19.40344524 | 8.30E-66 | 3.34E-64 | 138.8935862 |
| PCCB | -1.743268633 | 4.429654804 | -19.3809621 | 1.09E-65 | 4.37E-64 | 138.6228802 |
| GMPR | -2.363046926 | 3.24097053 | -19.36582498 | 1.31E-65 | 5.24E-64 | 138.4406659 |
| RGS19 | 1.64920069 | 5.107146063 | 19.36274079 | 1.36E-65 | 5.42E-64 | 138.4035438 |
| LST1 | 1.998445215 | 5.015633663 | 19.34721815 | 1.64E-65 | 6.52E-64 | 138.2167318 |
| CD300LF | 2.057647568 | 2.700797014 | 19.33499829 | 1.90E-65 | 7.54E-64 | 138.0696936 |
| HADH | -1.489103672 | 4.467679461 | -19.32673295 | 2.09E-65 | 8.31E-64 | 137.9702519 |
| KCTD1 | -1.885456029 | 2.84614701 | -19.31603194 | 2.38E-65 | 9.40E-64 | 137.8415217 |
| COL4A5 | -2.022942543 | 3.936237416 | -19.31568992 | 2.39E-65 | 9.42E-64 | 137.8374075 |
| SIGLEC10 | 2.287569685 | 3.372990317 | 19.30143342 | 2.84E-65 | 1.12E-63 | 137.6659335 |
| LAIR1 | 2.263369159 | 4.433681287 | 19.27436882 | 3.94E-65 | 1.54E-63 | 137.3404918 |
| AQP5 | -1.550336673 | 0.724877647 | -19.27315806 | 4.00E-65 | 1.56E-63 | 137.3259355 |
| CDH3 | -2.491978847 | 3.202847269 | -19.268896 | 4.21E-65 | 1.64E-63 | 137.2746969 |
| SLC14A2 | -2.44310721 | 1.876244565 | -19.25800981 | 4.80E-65 | 1.86E-63 | 137.1438354 |
| IGFBP3 | 3.249862517 | 9.955868277 | 19.25231178 | 5.14E-65 | 1.99E-63 | 137.0753474 |
| TNFRSF14 | 1.943241129 | 6.368580073 | 19.24705428 | 5.47E-65 | 2.11E-63 | 137.0121588 |
| SIM1 | -3.006913207 | 2.655464706 | -19.24356675 | 5.71E-65 | 2.20E-63 | 136.9702454 |
| SPI1 | 2.185268951 | 5.356844744 | 19.22899476 | 6.80E-65 | 2.61E-63 | 136.7951386 |
| CD1D | 1.639188575 | 2.631218848 | 19.2064239 | 8.93E-65 | 3.42E-63 | 136.5239772 |
| ANXA9 | -1.810166498 | 5.457653393 | -19.19236903 | 1.06E-64 | 4.04E-63 | 136.3551651 |
| VWA2 | -1.609053436 | 0.99900368 | -19.13491537 | 2.11E-64 | 8.03E-63 | 135.665416 |
| NAALADL2 | -1.428924331 | 1.83832423 | -19.12668931 | 2.33E-64 | 8.85E-63 | 135.5667023 |
| RBM11 | -1.410193152 | 1.876713747 | -19.07206284 | 4.50E-64 | 1.70E-62 | 134.9114523 |
| CD40 | 1.717442905 | 6.504690754 | 19.06233443 | 5.05E-64 | 1.91E-62 | 134.7948092 |
| HOMER1 | -2.049289895 | 2.217749929 | -19.05937159 | 5.24E-64 | 1.97E-62 | 134.759288 |
| GPR182 | -1.15865912 | 1.448620416 | -19.05507461 | 5.52E-64 | 2.07E-62 | 134.7077743 |
| C2orf15 | -1.329353722 | 1.881637595 | -19.04638373 | 6.12E-64 | 2.30E-62 | 134.6035944 |
| ISG20 | 1.623534414 | 3.332930052 | 19.02489149 | 7.93E-64 | 2.97E-62 | 134.3460135 |
| CLSTN2 | -2.241565084 | 3.078718574 | -19.02461533 | 7.95E-64 | 2.97E-62 | 134.3427043 |
| TNFSF9 | 2.727680593 | 3.49597882 | 19.00407295 | 1.02E-63 | 3.79E-62 | 134.0965786 |
| TNS4 | -1.403831967 | 0.873954605 | -18.99597791 | 1.12E-63 | 4.17E-62 | 133.999608 |
| SLC16A1 | 2.26559776 | 5.241464111 | 18.99122574 | 1.19E-63 | 4.40E-62 | 133.9426866 |
| TNFRSF4 | 2.375791185 | 4.12716475 | 18.97964968 | 1.37E-63 | 5.05E-62 | 133.8040444 |
| GAL3ST4 | 2.247498145 | 4.180867197 | 18.97800868 | 1.39E-63 | 5.13E-62 | 133.7843926 |
| FBXO2 | -2.686020156 | 2.596945618 | -18.97171254 | 1.50E-63 | 5.52E-62 | 133.708997 |
| ENPP3 | 4.539048786 | 6.170796679 | 18.94435652 | 2.09E-63 | 7.65E-62 | 133.3814876 |
| C6orf52 | -1.131527186 | 2.385843781 | -18.94064385 | 2.18E-63 | 7.98E-62 | 133.3370486 |
| PSMB9 | 1.964708366 | 5.865993106 | 18.93999771 | 2.20E-63 | 8.03E-62 | 133.3293149 |
| DHRS11 | -1.474805645 | 3.543783335 | -18.9339971 | 2.36E-63 | 8.61E-62 | 133.2574957 |
| GALNT3 | -2.367926952 | 3.045410413 | -18.93365091 | 2.37E-63 | 8.62E-62 | 133.2533526 |
| GMFG | 2.369528531 | 5.858052115 | 18.92820695 | 2.53E-63 | 9.18E-62 | 133.1882013 |
| NKG7 | 3.095297178 | 6.220139636 | 18.89428813 | 3.80E-63 | 1.38E-61 | 132.7823851 |
| AGAP2 | 1.393550183 | 1.804667639 | 18.88615664 | 4.19E-63 | 1.51E-61 | 132.6851258 |
| SIX4 | -1.52998129 | 1.21269807 | -18.86038274 | 5.71E-63 | 2.06E-61 | 132.3769224 |
| TMEM91 | 2.765580864 | 4.058690115 | 18.80167697 | 1.15E-62 | 4.15E-61 | 131.675341 |
| LAT2 | 1.78506328 | 4.547286072 | 18.7943222 | 1.26E-62 | 4.52E-61 | 131.587487 |
| CTH | -1.584981089 | 2.604447822 | -18.77741461 | 1.54E-62 | 5.52E-61 | 131.3855581 |
| ADORA3 | 2.21848116 | 4.257420449 | 18.75857863 | 1.93E-62 | 6.90E-61 | 131.1606562 |
| ADAP2 | 1.783108534 | 3.825325011 | 18.73899369 | 2.44E-62 | 8.71E-61 | 130.9268768 |
| TNFAIP8L2 | 1.911130594 | 3.598001492 | 18.73613168 | 2.53E-62 | 8.99E-61 | 130.8927194 |
| RASD2 | 2.261981992 | 4.168824121 | 18.72449075 | 2.91E-62 | 1.03E-60 | 130.7538024 |
| ZNF503 | -1.729093857 | 4.160572872 | -18.70233763 | 3.79E-62 | 1.34E-60 | 130.4895037 |
| BHLHE41 | 2.690975953 | 6.448908946 | 18.6974707 | 4.02E-62 | 1.42E-60 | 130.43145 |
| NR1I3 | -1.07773608 | 1.896625128 | -18.6879492 | 4.50E-62 | 1.59E-60 | 130.317888 |
| SLC16A7 | -2.435526447 | 2.521864287 | -18.68431601 | 4.70E-62 | 1.65E-60 | 130.2745594 |
| ERP27 | -3.117187907 | 3.498209106 | -18.68326286 | 4.76E-62 | 1.67E-60 | 130.2620002 |
| FMO5 | -1.990885906 | 3.4040508 | -18.68241222 | 4.81E-62 | 1.68E-60 | 130.2518561 |
| MLKL | 1.596430249 | 4.021691446 | 18.68176014 | 4.85E-62 | 1.69E-60 | 130.2440801 |
| WSCD2 | -1.574039026 | 2.613804355 | -18.68142535 | 4.87E-62 | 1.70E-60 | 130.2400876 |
| MT1H | -4.399416332 | 4.350960289 | -18.67197296 | 5.45E-62 | 1.89E-60 | 130.1273764 |
| ACOT11 | -1.605234068 | 2.707729196 | -18.66753045 | 5.75E-62 | 1.99E-60 | 130.0744088 |
| HS6ST1 | -1.949147146 | 4.627525151 | -18.6609936 | 6.21E-62 | 2.15E-60 | 129.996477 |
| HSPB7 | -2.950318653 | 3.569443589 | -18.64304762 | 7.70E-62 | 2.66E-60 | 129.7825654 |
| DTX1 | -1.951342219 | 2.451417183 | -18.6329237 | 8.69E-62 | 2.99E-60 | 129.661916 |
| LCP2 | 1.93510501 | 5.005631575 | 18.628586 | 9.15E-62 | 3.15E-60 | 129.610228 |
| SLC15A4 | 1.676335214 | 6.29870817 | 18.61944336 | 1.02E-61 | 3.50E-60 | 129.5012954 |
| FSTL4 | -1.328570587 | 2.013937934 | -18.60047652 | 1.28E-61 | 4.38E-60 | 129.2753573 |
| KCNJ12 | -1.519358904 | 1.447843886 | -18.59424171 | 1.38E-61 | 4.71E-60 | 129.2011006 |
| E2F1 | 2.094774287 | 3.882975248 | 18.57535643 | 1.73E-61 | 5.89E-60 | 128.976219 |
| SLC30A2 | -2.848145646 | 2.529703089 | -18.57106041 | 1.82E-61 | 6.19E-60 | 128.9250719 |
| SLC48A1 | -1.300884415 | 5.401138924 | -18.55782118 | 2.13E-61 | 7.23E-60 | 128.7674707 |
| C1QB | 2.936554967 | 8.092030555 | 18.55596094 | 2.18E-61 | 7.38E-60 | 128.7453287 |
| SELPLG | 2.333022096 | 4.607695734 | 18.52005856 | 3.34E-61 | 1.13E-59 | 128.3181146 |
| GAS2L3 | 2.556638915 | 4.391337993 | 18.51858427 | 3.40E-61 | 1.15E-59 | 128.3005766 |
| EPCAM | -3.166988012 | 5.414302517 | -18.51052076 | 3.75E-61 | 1.26E-59 | 128.2046607 |
| FOXA3 | -1.791050618 | 2.171455706 | -18.48459178 | 5.10E-61 | 1.71E-59 | 127.8963144 |
| TAPBP | 1.520505435 | 8.162343321 | 18.48304283 | 5.20E-61 | 1.74E-59 | 127.8778983 |
| LRRN2 | -2.494534873 | 2.960752631 | -18.41385014 | 1.19E-60 | 3.96E-59 | 127.0556873 |
| CD37 | 2.155034452 | 4.694809232 | 18.40326823 | 1.34E-60 | 4.48E-59 | 126.9300215 |
| CYP4F2 | -2.711509742 | 2.741548511 | -18.40216326 | 1.36E-60 | 4.53E-59 | 126.9169006 |
| IKBIP | 1.658433881 | 6.067175571 | 18.39701178 | 1.45E-60 | 4.80E-59 | 126.8557328 |
| GNLY | 2.346017786 | 3.178513281 | 18.34459116 | 2.70E-60 | 8.95E-59 | 126.2335813 |
| MARVELD2 | -2.203383031 | 3.713411769 | -18.32135186 | 3.57E-60 | 1.17E-58 | 125.9579327 |
| FMNL1 | 1.801191471 | 4.205900201 | 18.30424002 | 4.37E-60 | 1.43E-58 | 125.7550294 |
| RUNX3 | 2.222434091 | 4.594489556 | 18.29495332 | 4.88E-60 | 1.60E-58 | 125.6449358 |
| IDO1 | 2.946267628 | 5.674944354 | 18.29293664 | 5.00E-60 | 1.63E-58 | 125.6210303 |
| TUBAL3 | -1.984027899 | 1.834215374 | -18.25702815 | 7.66E-60 | 2.50E-58 | 125.1955054 |
| GPSM3 | 2.034858258 | 5.637738048 | 18.24549657 | 8.78E-60 | 2.86E-58 | 125.0589056 |
| MAL | -4.587002442 | 6.125424676 | -18.23754673 | 9.65E-60 | 3.13E-58 | 124.9647492 |
| OSCAR | 2.062510156 | 3.740693966 | 18.21765462 | 1.22E-59 | 3.96E-58 | 124.729204 |
| PRF1 | 2.618658273 | 4.624067522 | 18.21507418 | 1.26E-59 | 4.08E-58 | 124.6986543 |
| SELENBP1 | -1.605178525 | 5.954265709 | -18.20764896 | 1.38E-59 | 4.43E-58 | 124.6107547 |
| C1orf162 | 2.178235702 | 4.92718408 | 18.2076453 | 1.38E-59 | 4.43E-58 | 124.6107113 |
| SDS | 2.635327216 | 4.089688458 | 18.1743984 | 2.04E-59 | 6.56E-58 | 124.2172667 |
| C5orf38 | -2.094649741 | 1.775634797 | -18.17037357 | 2.14E-59 | 6.87E-58 | 124.1696514 |
| TRADD | 1.357707145 | 5.257702572 | 18.16160264 | 2.38E-59 | 7.61E-58 | 124.065899 |
| CCL5 | 2.857209259 | 7.335557102 | 18.16136719 | 2.38E-59 | 7.62E-58 | 124.063114 |
| CORO1A | 2.267940774 | 5.485133411 | 18.15313557 | 2.63E-59 | 8.38E-58 | 123.9657553 |
| GZMH | 2.436705969 | 4.661915135 | 18.14731037 | 2.82E-59 | 8.96E-58 | 123.8968663 |
| PAQR7 | -1.411777197 | 4.330768723 | -18.13830707 | 3.13E-59 | 9.95E-58 | 123.790406 |
| C1QC | 2.830948859 | 7.589738111 | 18.13102748 | 3.42E-59 | 1.08E-57 | 123.7043398 |
| ZNF395 | 2.57907425 | 7.33843 | 18.12531213 | 3.66E-59 | 1.16E-57 | 123.6367746 |
| CAPS | -2.063174263 | 3.760165687 | -18.11367 | 4.20E-59 | 1.32E-57 | 123.4991651 |
| PRDM1 | 2.016060334 | 4.752062128 | 18.05621092 | 8.29E-59 | 2.61E-57 | 122.8203953 |
| ANGPT2 | 2.742210729 | 5.695007683 | 18.05295674 | 8.62E-59 | 2.71E-57 | 122.7819731 |
| LILRB2 | 1.855935312 | 3.921241025 | 18.03221228 | 1.10E-58 | 3.45E-57 | 122.5370926 |
| SIGLEC8 | 2.641547291 | 3.128419744 | 18.02487513 | 1.20E-58 | 3.75E-57 | 122.4505011 |
| GAS1 | -1.978989468 | 2.497774468 | -17.93489708 | 3.48E-58 | 1.09E-56 | 121.3894869 |
| AGR2 | -2.447283635 | 1.092761071 | -17.92544191 | 3.89E-58 | 1.21E-56 | 121.2780883 |
| SLC26A4 | -1.650938442 | 1.165205025 | -17.92020446 | 4.14E-58 | 1.29E-56 | 121.2163898 |
| GLOD5 | -1.838596286 | 1.650110676 | -17.91552347 | 4.38E-58 | 1.36E-56 | 121.1612514 |
| SASH3 | 2.184169132 | 3.952344579 | 17.90330765 | 5.06E-58 | 1.56E-56 | 121.0173798 |
| KRBA1 | 2.294760671 | 4.639702835 | 17.90050987 | 5.23E-58 | 1.61E-56 | 120.9844333 |
| PIP4K2C | -1.284559136 | 4.964817265 | -17.90034541 | 5.24E-58 | 1.61E-56 | 120.9824967 |
| HCLS1 | 1.962097241 | 5.706257876 | 17.88785518 | 6.07E-58 | 1.87E-56 | 120.8354328 |
| GZMA | 2.735849678 | 4.891794185 | 17.87468413 | 7.09E-58 | 2.18E-56 | 120.6803877 |
| STAMBPL1 | 1.720825518 | 3.030545528 | 17.86571123 | 7.88E-58 | 2.42E-56 | 120.5747824 |
| DTX2 | 1.055583002 | 3.979403072 | 17.84093866 | 1.06E-57 | 3.23E-56 | 120.2833124 |
| C1QA | 2.771391458 | 7.937974548 | 17.83259816 | 1.17E-57 | 3.56E-56 | 120.1852084 |
| TBXAS1 | 1.572928234 | 4.50228324 | 17.82644794 | 1.25E-57 | 3.81E-56 | 120.1128765 |
| THRB | -1.698323507 | 3.204033573 | -17.81993149 | 1.35E-57 | 4.11E-56 | 120.0362462 |
| FOXC1 | -1.974341611 | 3.7479278 | -17.81919858 | 1.36E-57 | 4.13E-56 | 120.0276281 |
| ARRDC2 | 2.037818121 | 5.034914332 | 17.81380366 | 1.45E-57 | 4.40E-56 | 119.9641939 |
| STAP1 | -3.002350015 | 1.702659393 | -17.78806365 | 1.97E-57 | 5.94E-56 | 119.6616242 |
| CR2 | -2.027660138 | 1.430795952 | -17.77531396 | 2.29E-57 | 6.89E-56 | 119.5118056 |
| EDA2R | 1.902757051 | 4.165916416 | 17.77082634 | 2.41E-57 | 7.24E-56 | 119.4590808 |
| IGSF11 | -1.495587255 | 1.524081988 | -17.7622291 | 2.67E-57 | 8.00E-56 | 119.3580842 |
| CAV1 | 2.265544299 | 8.566331296 | 17.75281692 | 2.98E-57 | 8.90E-56 | 119.2475321 |
| ITGB2 | 2.29410246 | 6.490409936 | 17.73736025 | 3.58E-57 | 1.07E-55 | 119.0660246 |
| CD247 | 1.9385807 | 3.401870472 | 17.73619462 | 3.63E-57 | 1.08E-55 | 119.0523387 |
| NPTX2 | 4.897401138 | 6.031836835 | 17.73371315 | 3.74E-57 | 1.11E-55 | 119.0232042 |
| WNT7B | -2.042807351 | 2.222705203 | -17.72957585 | 3.92E-57 | 1.16E-55 | 118.9746317 |
| AIF1L | -3.352416293 | 6.98801305 | -17.72732583 | 4.03E-57 | 1.19E-55 | 118.9482178 |
| FAM181B | -1.171333516 | 1.821506816 | -17.7200565 | 4.39E-57 | 1.29E-55 | 118.8628874 |
| CLDN11 | -1.613480923 | 2.336667922 | -17.70548112 | 5.21E-57 | 1.53E-55 | 118.6918296 |
| PDP2 | -1.490095586 | 2.785347174 | -17.69831513 | 5.67E-57 | 1.67E-55 | 118.6077458 |
| CCDC160 | -2.011592473 | 3.585774709 | -17.6898751 | 6.26E-57 | 1.84E-55 | 118.5087268 |
| PFKFB4 | 2.006590838 | 3.750623002 | 17.68229595 | 6.85E-57 | 2.00E-55 | 118.4198208 |
| LSAMP | -1.731127937 | 1.596961876 | -17.66197363 | 8.69E-57 | 2.54E-55 | 118.1814945 |
| TBL1Y | -1.073887782 | 1.930318301 | -17.65869571 | 9.04E-57 | 2.64E-55 | 118.1430616 |
| ALDH6A1 | -2.441519117 | 4.395019723 | -17.65365518 | 9.59E-57 | 2.79E-55 | 118.0839672 |
| LILRB4 | 2.192630137 | 4.074145226 | 17.65221373 | 9.75E-57 | 2.83E-55 | 118.0670689 |
| CAND2 | -1.259809445 | 2.854492363 | -17.62797882 | 1.30E-56 | 3.76E-55 | 117.7830268 |
| DBT | -1.311514779 | 4.356602327 | -17.62725457 | 1.31E-56 | 3.79E-55 | 117.7745403 |
| DEGS2 | -2.097172115 | 1.97077609 | -17.62500141 | 1.34E-56 | 3.88E-55 | 117.7481393 |
| HPD | -3.877410881 | 3.273035683 | -17.62442556 | 1.35E-56 | 3.90E-55 | 117.741392 |
| KCNQ1 | -1.84052567 | 4.527794061 | -17.62078324 | 1.41E-56 | 4.06E-55 | 117.6987167 |
| SFXN2 | -1.650238316 | 3.452813543 | -17.60394353 | 1.72E-56 | 4.94E-55 | 117.501451 |
| TRPM2 | 1.756046316 | 3.111220266 | 17.59777446 | 1.85E-56 | 5.30E-55 | 117.4292001 |
| TNFRSF10B | 1.405811325 | 5.312124153 | 17.56626214 | 2.68E-56 | 7.66E-55 | 117.060265 |
| PDHB | -1.092800571 | 5.41956784 | -17.56266037 | 2.79E-56 | 7.98E-55 | 117.0181106 |
| DEF6 | 1.900645769 | 4.189831632 | 17.5594374 | 2.90E-56 | 8.27E-55 | 116.9803921 |
| RPS2 | 1.389767522 | 10.15557112 | 17.55813454 | 2.95E-56 | 8.39E-55 | 116.9651453 |
| SLC9A3 | -3.002607278 | 2.681675995 | -17.54628388 | 3.38E-56 | 9.59E-55 | 116.8264799 |
| CYFIP2 | -2.375522192 | 5.519283723 | -17.52986864 | 4.10E-56 | 1.16E-54 | 116.6344554 |
| PFKFB2 | -1.900352854 | 4.709220008 | -17.52427699 | 4.38E-56 | 1.24E-54 | 116.5690584 |
| HECW1 | -1.338880911 | 2.025255814 | -17.52105895 | 4.55E-56 | 1.28E-54 | 116.531425 |
| ALB | -3.376203023 | 1.994376491 | -17.52032329 | 4.59E-56 | 1.29E-54 | 116.5228221 |
| ARHGDIB | 1.628697231 | 8.504738762 | 17.51076165 | 5.13E-56 | 1.44E-54 | 116.4110185 |
| CLEC2B | 1.833536856 | 4.835269868 | 17.50064286 | 5.78E-56 | 1.62E-54 | 116.2927225 |
| SLC37A2 | 2.2314673 | 4.519300302 | 17.5001076 | 5.82E-56 | 1.62E-54 | 116.2864656 |
| RAP1GAP | -2.605405199 | 5.380920213 | -17.48625571 | 6.84E-56 | 1.91E-54 | 116.1245656 |
| NLRC5 | 1.739445025 | 4.65807367 | 17.48440895 | 6.99E-56 | 1.95E-54 | 116.1029841 |
| DGKD | 1.874357152 | 4.610091961 | 17.47469506 | 7.84E-56 | 2.18E-54 | 115.9894785 |
| BID | 1.002422837 | 5.183575025 | 17.4710145 | 8.18E-56 | 2.27E-54 | 115.9464771 |
| FERMT3 | 2.035475835 | 4.455866802 | 17.46975982 | 8.30E-56 | 2.30E-54 | 115.9318189 |
| BBC3 | 1.48743945 | 3.153112188 | 17.46427422 | 8.85E-56 | 2.45E-54 | 115.8677358 |
| TPI1P2 | -1.22179583 | 1.29110811 | -17.46299567 | 8.99E-56 | 2.48E-54 | 115.8528008 |
| AHNAK2 | 2.749213552 | 4.335094252 | 17.44745739 | 1.08E-55 | 2.96E-54 | 115.6713229 |
| BIN2 | 1.656323679 | 3.841137864 | 17.44054785 | 1.17E-55 | 3.21E-54 | 115.5906412 |
| LSP1 | 2.044619898 | 4.106472603 | 17.42336551 | 1.43E-55 | 3.92E-54 | 115.3900521 |
| DPEP2 | 1.448249008 | 2.368972516 | 17.4064687 | 1.74E-55 | 4.76E-54 | 115.1928617 |
| SERPINA4 | -2.347499598 | 2.142897234 | -17.40576484 | 1.76E-55 | 4.79E-54 | 115.1846489 |
| SLC13A3 | -3.905918485 | 2.302731253 | -17.40353985 | 1.80E-55 | 4.91E-54 | 115.1586878 |
| LAD1 | -3.076262201 | 3.276359446 | -17.39294068 | 2.04E-55 | 5.55E-54 | 115.0350327 |
| ACAA1 | -1.469120995 | 4.140234663 | -17.39273375 | 2.05E-55 | 5.56E-54 | 115.0326189 |
| CLIC5 | -2.663376211 | 3.023968804 | -17.38192907 | 2.32E-55 | 6.29E-54 | 114.9065932 |
| DOK7 | -1.346437459 | 2.26614532 | -17.37464997 | 2.53E-55 | 6.84E-54 | 114.8217049 |
| NXPH4 | 3.079036677 | 4.507894963 | 17.35716458 | 3.10E-55 | 8.38E-54 | 114.6178412 |
| MS4A7 | 2.564347531 | 4.811101906 | 17.35256668 | 3.27E-55 | 8.83E-54 | 114.5642455 |
| CBLC | -2.057054609 | 2.533505292 | -17.34426261 | 3.61E-55 | 9.71E-54 | 114.467461 |
| OVOL1 | -1.757583552 | 1.662704987 | -17.33254297 | 4.14E-55 | 1.11E-53 | 114.3308948 |
| HMOX1 | 2.59028464 | 8.569938624 | 17.33243288 | 4.14E-55 | 1.11E-53 | 114.3296121 |
| CGN | -2.458280404 | 3.844237438 | -17.31960155 | 4.81E-55 | 1.29E-53 | 114.1801284 |
| CST7 | 2.79008089 | 5.335483356 | 17.31638462 | 5.00E-55 | 1.33E-53 | 114.1426574 |
| PLXDC1 | 2.08442941 | 4.03178023 | 17.31548801 | 5.05E-55 | 1.35E-53 | 114.132214 |
| LGALS1 | 1.950808269 | 8.207247461 | 17.31534173 | 5.06E-55 | 1.35E-53 | 114.1305103 |
| HCK | 1.976003381 | 4.992951544 | 17.31004359 | 5.38E-55 | 1.43E-53 | 114.0688037 |
| PVALB | -4.20949467 | 2.843628002 | -17.30644494 | 5.61E-55 | 1.49E-53 | 114.0268946 |
| KCNH3 | -1.058344161 | 0.938856169 | -17.3045042 | 5.74E-55 | 1.52E-53 | 114.0042944 |
| MS4A6A | 2.318920241 | 4.712824686 | 17.2856143 | 7.16E-55 | 1.89E-53 | 113.7843643 |
| APBB1IP | 2.430221595 | 5.391294073 | 17.27120644 | 8.47E-55 | 2.23E-53 | 113.6166734 |
| ABAT | -2.198364087 | 4.027215112 | -17.26209108 | 9.42E-55 | 2.48E-53 | 113.5106059 |
| CCM2 | 1.399267291 | 4.644477295 | 17.25910196 | 9.75E-55 | 2.56E-53 | 113.4758285 |
| WAS | 1.92537495 | 5.521683382 | 17.20527318 | 1.83E-54 | 4.80E-53 | 112.8499056 |
| APOO | -1.224985627 | 4.120072065 | -17.19143887 | 2.15E-54 | 5.63E-53 | 112.6891501 |
| ASF1B | 1.799771466 | 4.644071287 | 17.18312003 | 2.37E-54 | 6.19E-53 | 112.5925065 |
| SCN1B | 1.496324293 | 2.756706679 | 17.18176344 | 2.40E-54 | 6.28E-53 | 112.576748 |
| LPCAT1 | 2.195358641 | 7.075640641 | 17.17545851 | 2.59E-54 | 6.74E-53 | 112.5035139 |
| AMFR | -1.258050785 | 6.979767899 | -17.15512789 | 3.28E-54 | 8.52E-53 | 112.2674304 |
| DPP9 | 1.400779826 | 5.105190413 | 17.1209069 | 4.88E-54 | 1.26E-52 | 111.8702717 |
| CPNE6 | -1.206811003 | 1.884590333 | -17.10769452 | 5.69E-54 | 1.47E-52 | 111.7170078 |
| ST6GAL1 | -2.096576573 | 5.497523999 | -17.08839777 | 7.13E-54 | 1.84E-52 | 111.4932405 |
| BTK | 1.689118694 | 3.118678797 | 17.0880808 | 7.15E-54 | 1.84E-52 | 111.4895657 |
| TMEM164 | -1.324291042 | 4.499280065 | -17.06923346 | 8.91E-54 | 2.29E-52 | 111.2710981 |
| PPM1H | -1.968245461 | 3.993957992 | -17.0565441 | 1.03E-53 | 2.65E-52 | 111.1240587 |
| ADAMTS16 | -2.008582477 | 2.266347341 | -17.0542222 | 1.06E-53 | 2.72E-52 | 111.0971577 |
| CSF3R | 2.019585114 | 2.796606075 | 17.04233413 | 1.22E-53 | 3.12E-52 | 110.9594455 |
| COL4A4 | -2.023492267 | 3.975323707 | -17.03988028 | 1.25E-53 | 3.20E-52 | 110.9310242 |
| DACH1 | -1.97260403 | 2.859198162 | -17.03692895 | 1.30E-53 | 3.31E-52 | 110.8968429 |
| BDH1 | -1.753028607 | 1.752333851 | -17.03466846 | 1.33E-53 | 3.39E-52 | 110.8706641 |
| TRPM6 | -1.096290392 | 1.746816998 | -17.03349225 | 1.35E-53 | 3.43E-52 | 110.8570429 |
| EPB41L5 | -1.689550876 | 4.150978274 | -17.01121505 | 1.75E-53 | 4.44E-52 | 110.5991231 |
| APOBEC3G | 1.855866148 | 3.670594407 | 17.00281584 | 1.93E-53 | 4.89E-52 | 110.5019107 |
| FABP1 | -3.111626492 | 2.731922838 | -17.00105238 | 1.97E-53 | 4.97E-52 | 110.4815026 |
| AGRP | -1.004163361 | 2.251884774 | -17.00099672 | 1.97E-53 | 4.97E-52 | 110.4808585 |
| CARD16 | 1.596404072 | 4.742168391 | 16.99726056 | 2.06E-53 | 5.19E-52 | 110.4376233 |
| PDLIM1 | 1.629036208 | 7.034556488 | 16.9907742 | 2.22E-53 | 5.58E-52 | 110.362571 |
| PPM1K | -1.325760416 | 4.250899943 | -16.97959851 | 2.52E-53 | 6.35E-52 | 110.2332835 |
| SYP | -1.18415161 | 1.502219225 | -16.9546867 | 3.37E-53 | 8.46E-52 | 109.9451985 |
| C1QTNF6 | 1.629380332 | 3.828162336 | 16.94859331 | 3.62E-53 | 9.07E-52 | 109.8747567 |
| LILRB3 | 1.631458684 | 3.121494238 | 16.94325044 | 3.85E-53 | 9.63E-52 | 109.8129988 |
| VAV1 | 1.916889385 | 3.145169073 | 16.92320058 | 4.85E-53 | 1.21E-51 | 109.5813066 |
| SLFN13 | 1.759871889 | 4.469487442 | 16.91331797 | 5.44E-53 | 1.36E-51 | 109.4671418 |
| THSD4 | -1.815029603 | 4.292796868 | -16.90609351 | 5.92E-53 | 1.47E-51 | 109.3836996 |
| CORO7 | 1.056888055 | 2.420373577 | 16.8614672 | 9.92E-53 | 2.47E-51 | 108.8685572 |
| ITGAL | 1.947013457 | 4.062778453 | 16.86108078 | 9.97E-53 | 2.48E-51 | 108.8640987 |
| FATE1 | 1.919420273 | 2.75712818 | 16.85847728 | 1.03E-52 | 2.55E-51 | 108.8340609 |
| FAM171A1 | -1.981251736 | 5.28251771 | -16.85680167 | 1.05E-52 | 2.59E-51 | 108.8147296 |
| ZNF728 | -1.073777808 | 0.758110458 | -16.85148022 | 1.11E-52 | 2.75E-51 | 108.7533411 |
| CCDC88B | 1.612062656 | 3.799396088 | 16.85023908 | 1.13E-52 | 2.78E-51 | 108.7390244 |
| LDHA | 1.750877016 | 9.374405313 | 16.84209551 | 1.24E-52 | 3.05E-51 | 108.6450961 |
| MSC | 2.612470667 | 5.70818697 | 16.83998673 | 1.27E-52 | 3.13E-51 | 108.6207761 |
| AEN | 1.25664824 | 5.166210295 | 16.82974645 | 1.43E-52 | 3.51E-51 | 108.5026937 |
| HSPB8 | 2.28140783 | 7.351060104 | 16.82754619 | 1.47E-52 | 3.60E-51 | 108.4773256 |
| PLK2 | 2.080218992 | 6.352571038 | 16.81958824 | 1.61E-52 | 3.94E-51 | 108.3855838 |
| CD2 | 2.663382415 | 5.414519001 | 16.81494704 | 1.70E-52 | 4.15E-51 | 108.3320861 |
| MT1G | -4.673426895 | 4.755199245 | -16.81384227 | 1.72E-52 | 4.20E-51 | 108.3193524 |
| HOXB6 | -1.741433529 | 2.794060746 | -16.81162226 | 1.77E-52 | 4.30E-51 | 108.2937655 |
| OLFML2B | 2.422291461 | 5.373505648 | 16.80802261 | 1.84E-52 | 4.47E-51 | 108.2522802 |
| PCGF1 | 1.179392326 | 4.951973277 | 16.80507625 | 1.91E-52 | 4.62E-51 | 108.2183264 |
| PLEKHO1 | 1.599183525 | 4.795420308 | 16.79040216 | 2.26E-52 | 5.47E-51 | 108.0492549 |
| ARHGAP25 | 1.56949543 | 3.835382266 | 16.78510587 | 2.40E-52 | 5.79E-51 | 107.9882458 |
| RFC2 | 1.065874061 | 5.625823741 | 16.78504415 | 2.40E-52 | 5.79E-51 | 107.9875348 |
| AK3 | -1.358859027 | 6.306187287 | -16.77229562 | 2.78E-52 | 6.70E-51 | 107.8407112 |
| APEH | -1.18528618 | 5.169533751 | -16.75679676 | 3.33E-52 | 8.00E-51 | 107.6622681 |
| EVL | 1.489817092 | 4.407338615 | 16.75186712 | 3.52E-52 | 8.46E-51 | 107.6055246 |
| MYO1G | 1.746911183 | 3.27316184 | 16.74879261 | 3.65E-52 | 8.75E-51 | 107.570138 |
| FKBP11 | 1.49871224 | 4.402728843 | 16.73855824 | 4.11E-52 | 9.83E-51 | 107.4523614 |
| PLOD3 | 1.271878111 | 6.046026365 | 16.7249544 | 4.81E-52 | 1.15E-50 | 107.2958507 |
| NOP16 | 1.5545991 | 4.307859621 | 16.71888259 | 5.16E-52 | 1.23E-50 | 107.2260105 |
| TMCC1 | 1.647027767 | 4.699084182 | 16.71804796 | 5.21E-52 | 1.24E-50 | 107.216411 |
| GABARAPL1 | -1.638995049 | 7.226918975 | -16.71718653 | 5.26E-52 | 1.25E-50 | 107.2065036 |
| TYMS | 1.573054286 | 4.874464146 | 16.7034361 | 6.16E-52 | 1.46E-50 | 107.0483823 |
| ANO5 | -2.077445457 | 1.515528262 | -16.70250399 | 6.23E-52 | 1.48E-50 | 107.0376654 |
| CSTA | 1.713833014 | 2.769499601 | 16.69644681 | 6.68E-52 | 1.58E-50 | 106.9680287 |
| ADH1B | -3.204175003 | 2.883091278 | -16.69301028 | 6.95E-52 | 1.64E-50 | 106.9285246 |
| CYP27B1 | -1.848910394 | 3.360082066 | -16.67859598 | 8.21E-52 | 1.94E-50 | 106.7628606 |
| S100A14 | -2.264773452 | 4.256197547 | -16.66799644 | 9.27E-52 | 2.19E-50 | 106.6410743 |
| RNASET2 | 2.505745924 | 5.375568608 | 16.65369095 | 1.09E-51 | 2.57E-50 | 106.4767538 |
| TMC8 | 1.863973241 | 3.659196678 | 16.64606972 | 1.19E-51 | 2.81E-50 | 106.3892339 |
| NFAM1 | 1.585199478 | 4.286997735 | 16.64089645 | 1.27E-51 | 2.97E-50 | 106.3298344 |
| BTN3A2 | 1.786137732 | 5.14341439 | 16.63938867 | 1.29E-51 | 3.02E-50 | 106.3125233 |
| CORO2B | -1.963724128 | 3.442155804 | -16.6222431 | 1.57E-51 | 3.68E-50 | 106.1157145 |
| RELT | 1.360527202 | 1.98654551 | 16.61572798 | 1.69E-51 | 3.96E-50 | 106.0409496 |
| LOXL2 | 2.874906509 | 5.789902411 | 16.59918758 | 2.05E-51 | 4.78E-50 | 105.8511887 |
| PHYHD1 | -2.443511013 | 3.606918318 | -16.58998856 | 2.28E-51 | 5.30E-50 | 105.7456833 |
| CD52 | 2.188379543 | 5.895723198 | 16.58872212 | 2.31E-51 | 5.37E-50 | 105.7311601 |
| DGKZ | 1.17606708 | 5.407550376 | 16.55519772 | 3.40E-51 | 7.88E-50 | 105.3468628 |
| ARHGAP9 | 1.812153162 | 2.670143168 | 16.55041735 | 3.59E-51 | 8.30E-50 | 105.2920887 |
| COL9A2 | -2.105415736 | 2.972753091 | -16.54726882 | 3.72E-51 | 8.59E-50 | 105.2560156 |
| AFM | -2.586451906 | 1.825867096 | -16.53697064 | 4.19E-51 | 9.66E-50 | 105.1380469 |
| MATK | 1.36674236 | 3.514110131 | 16.53358326 | 4.36E-51 | 1.00E-49 | 105.0992496 |
| EZH2 | 1.603633374 | 4.056954979 | 16.51383077 | 5.47E-51 | 1.25E-49 | 104.8730756 |
| IFFO1 | 1.479500035 | 4.665921958 | 16.51011457 | 5.70E-51 | 1.31E-49 | 104.8305352 |
| EFHD2 | 1.477751219 | 5.84778408 | 16.50860887 | 5.80E-51 | 1.33E-49 | 104.8133001 |
| CDT1 | 1.623643615 | 3.717357487 | 16.50438257 | 6.09E-51 | 1.39E-49 | 104.7649267 |
| CYP17A1 | -2.278064316 | 1.055810444 | -16.50261963 | 6.22E-51 | 1.42E-49 | 104.7447499 |
| VWF | 2.878079431 | 8.696554174 | 16.49928526 | 6.46E-51 | 1.47E-49 | 104.7065903 |
| WIPF1 | 1.38945636 | 5.758941158 | 16.47537471 | 8.50E-51 | 1.93E-49 | 104.4330376 |
| SAMD12 | -1.458202578 | 2.716772544 | -16.46557193 | 9.51E-51 | 2.16E-49 | 104.3209316 |
| TFAP2A | -1.94613434 | 2.352435208 | -16.45896925 | 1.03E-50 | 2.33E-49 | 104.245437 |
| DLGAP3 | -1.149771755 | 0.974093854 | -16.43780716 | 1.31E-50 | 2.96E-49 | 104.0035503 |
| NR3C2 | -2.149310792 | 3.509576987 | -16.43711218 | 1.32E-50 | 2.98E-49 | 103.9956086 |
| CTSW | 2.386036627 | 5.008264104 | 16.42859966 | 1.45E-50 | 3.28E-49 | 103.8983446 |
| NCF4 | 1.730530863 | 3.506022487 | 16.42080572 | 1.59E-50 | 3.59E-49 | 103.8093084 |
| EHD2 | 2.165706984 | 7.584561026 | 16.41333992 | 1.73E-50 | 3.90E-49 | 103.7240362 |
| FA2H | -2.028283172 | 2.044147011 | -16.4063651 | 1.88E-50 | 4.22E-49 | 103.6443855 |
| RNF149 | 1.580809399 | 6.452688363 | 16.39348659 | 2.17E-50 | 4.88E-49 | 103.497351 |
| KCNJ13 | -1.827582406 | 2.199416693 | -16.39302697 | 2.18E-50 | 4.90E-49 | 103.4921044 |
| ILDR1 | -2.007245354 | 4.079062245 | -16.38861997 | 2.30E-50 | 5.15E-49 | 103.4418003 |
| ALX1 | -1.50175938 | 2.055035223 | -16.38723892 | 2.33E-50 | 5.22E-49 | 103.4260374 |
| MAGI3 | -1.743015326 | 3.332003157 | -16.35885111 | 3.23E-50 | 7.22E-49 | 103.1021407 |
| BAX | 1.276936248 | 6.624059248 | 16.34736543 | 3.69E-50 | 8.22E-49 | 102.9711552 |
| CNTN3 | -1.980432889 | 2.324027164 | -16.34588509 | 3.75E-50 | 8.35E-49 | 102.9542757 |
| HOXD8 | -1.938329092 | 4.55189405 | -16.33873188 | 4.07E-50 | 9.05E-49 | 102.87272 |
| MRGPRF | -1.856608013 | 3.309635496 | -16.33804846 | 4.10E-50 | 9.11E-49 | 102.8649289 |
| GLDC | -2.144800933 | 4.051506506 | -16.32973693 | 4.51E-50 | 1.00E-48 | 102.770186 |
| MTCP1 | 1.583147132 | 2.956698973 | 16.3293261 | 4.53E-50 | 1.00E-48 | 102.7655035 |
| VAT1L | -2.304821658 | 2.166135813 | -16.31985112 | 5.05E-50 | 1.12E-48 | 102.6575229 |
| JAK3 | 1.876142586 | 4.689395239 | 16.3172572 | 5.20E-50 | 1.15E-48 | 102.6279658 |
| CGREF1 | 2.498820382 | 5.106836854 | 16.31229011 | 5.50E-50 | 1.21E-48 | 102.5713724 |
| PTP4A3 | 1.730487347 | 5.598755042 | 16.31189877 | 5.53E-50 | 1.22E-48 | 102.5669138 |
| CASP1 | 1.584671733 | 5.042498967 | 16.30659762 | 5.87E-50 | 1.29E-48 | 102.5065223 |
| TBC1D24 | -1.207404826 | 2.476885826 | -16.3055436 | 5.95E-50 | 1.31E-48 | 102.4945156 |
| PTPRE | 1.437302904 | 3.275617315 | 16.30408406 | 6.05E-50 | 1.33E-48 | 102.4778902 |
| CSPG4 | 2.523896732 | 5.24979595 | 16.30006977 | 6.33E-50 | 1.39E-48 | 102.4321666 |
| CYTH4 | 1.715119775 | 3.911669641 | 16.27300065 | 8.62E-50 | 1.89E-48 | 102.1239612 |
| CMTM4 | -1.916615308 | 4.213849011 | -16.27288288 | 8.63E-50 | 1.89E-48 | 102.1226207 |
| PLA2G7 | 2.393856863 | 3.934392192 | 16.26072806 | 9.92E-50 | 2.16E-48 | 101.9842942 |
| GRAMD1A | 1.217757462 | 6.237995514 | 16.2599526 | 1.00E-49 | 2.18E-48 | 101.9754706 |
| TRIB3 | 2.504599737 | 4.861335569 | 16.23463775 | 1.34E-49 | 2.91E-48 | 101.6875156 |
| LTB4R | 1.896390747 | 2.843181103 | 16.23302175 | 1.36E-49 | 2.96E-48 | 101.6691399 |
| VWA5B1 | -1.223141983 | 1.839587619 | -16.22237706 | 1.54E-49 | 3.33E-48 | 101.5481155 |
| EBI3 | 1.830133069 | 4.591563156 | 16.21945052 | 1.59E-49 | 3.44E-48 | 101.5148479 |
| STX4 | 1.115812421 | 6.375021893 | 16.19787011 | 2.03E-49 | 4.40E-48 | 101.2696056 |
| GPD1L | -1.670089348 | 4.827470755 | -16.19561325 | 2.09E-49 | 4.50E-48 | 101.243966 |
| GBP2 | 1.821752267 | 5.586864825 | 16.19491805 | 2.10E-49 | 4.53E-48 | 101.2360684 |
| EPHA1 | -1.28869719 | 2.879072756 | -16.1829527 | 2.41E-49 | 5.19E-48 | 101.1001592 |
| SLC6A3 | 4.778368406 | 5.886582129 | 16.1768853 | 2.58E-49 | 5.55E-48 | 101.0312578 |
| BATF | 2.066612316 | 3.374554063 | 16.16443247 | 2.98E-49 | 6.39E-48 | 100.8898759 |
| MPP2 | -1.21306848 | 2.189327184 | -16.16089531 | 3.10E-49 | 6.65E-48 | 100.8497251 |
| NUDT4 | -1.532823903 | 4.369273622 | -16.1527979 | 3.40E-49 | 7.28E-48 | 100.7578238 |
| POU5F1 | 2.507614846 | 4.84375106 | 16.14785948 | 3.59E-49 | 7.69E-48 | 100.7017844 |
| PDK1 | 1.657770079 | 4.325405989 | 16.14202041 | 3.84E-49 | 8.21E-48 | 100.6355338 |
| P2RX7 | 1.689340256 | 3.231193156 | 16.14048897 | 3.91E-49 | 8.34E-48 | 100.6181595 |
| SLC38A3 | -1.465064835 | 1.779507902 | -16.09626031 | 6.46E-49 | 1.38E-47 | 100.1166715 |
| GPM6B | -1.650988058 | 2.804460441 | -16.08883589 | 7.03E-49 | 1.49E-47 | 100.0325443 |
| GJC1 | 2.171631042 | 4.40443227 | 16.07061052 | 8.65E-49 | 1.84E-47 | 99.82609709 |
| MAP3K9 | -1.300048448 | 1.984401212 | -16.06927497 | 8.79E-49 | 1.86E-47 | 99.81097241 |
| PDHA1 | -1.070884749 | 6.159450565 | -16.06909592 | 8.80E-49 | 1.86E-47 | 99.80894476 |
| GZMB | 1.929518435 | 4.589359547 | 16.04901902 | 1.11E-48 | 2.34E-47 | 99.58164377 |
| NDC80 | 1.430540242 | 2.155059901 | 16.04365851 | 1.18E-48 | 2.48E-47 | 99.52097429 |
| TPX2 | 1.933659751 | 3.544517589 | 16.03987784 | 1.23E-48 | 2.59E-47 | 99.47819031 |
| BMP1 | 1.748631455 | 5.64514925 | 16.03365048 | 1.32E-48 | 2.77E-47 | 99.40772721 |
| C7 | -4.03334386 | 4.925724703 | -16.03300329 | 1.33E-48 | 2.79E-47 | 99.40040484 |
| PRAM1 | 1.414461385 | 3.637023949 | 16.02194634 | 1.50E-48 | 3.16E-47 | 99.27532422 |
| PLA2R1 | -1.785768511 | 2.215651289 | -16.00384311 | 1.85E-48 | 3.87E-47 | 99.07060998 |
| P2RY1 | 1.754853809 | 3.786797214 | 16.00099965 | 1.91E-48 | 3.99E-47 | 99.03846434 |
| CD86 | 1.902775576 | 4.57775944 | 15.99183863 | 2.12E-48 | 4.43E-47 | 98.93491389 |
| CDH16 | -2.543151969 | 7.143936102 | -15.98909442 | 2.18E-48 | 4.56E-47 | 98.90389985 |
| SLC20A2 | -1.257847179 | 4.264084797 | -15.9820124 | 2.37E-48 | 4.93E-47 | 98.82387155 |
| RASSF2 | 1.92898736 | 4.253038758 | 15.97753946 | 2.49E-48 | 5.18E-47 | 98.77333398 |
| GRIK3 | 2.664202758 | 4.160417736 | 15.97736398 | 2.49E-48 | 5.19E-47 | 98.77135144 |
| CCNA2 | 1.541690683 | 4.279957186 | 15.97694409 | 2.51E-48 | 5.21E-47 | 98.76660755 |
| FBXL8 | 1.23489245 | 4.397092386 | 15.97394724 | 2.59E-48 | 5.38E-47 | 98.73275139 |
| ELMO1 | 1.683185766 | 4.588037454 | 15.97016597 | 2.71E-48 | 5.61E-47 | 98.69003725 |
| PRDX4 | 1.353461227 | 7.79445665 | 15.96805884 | 2.77E-48 | 5.73E-47 | 98.66623628 |
| LARS2 | -1.180079847 | 3.34695082 | -15.93560514 | 4.00E-48 | 8.26E-47 | 98.29982301 |
| EMP3 | 1.575900808 | 7.088728634 | 15.92166728 | 4.69E-48 | 9.65E-47 | 98.14255483 |
| PHKA2 | 1.83422249 | 5.170314242 | 15.91934925 | 4.81E-48 | 9.89E-47 | 98.11640476 |
| NCKAP1L | 1.96962919 | 4.008312273 | 15.91185525 | 5.24E-48 | 1.08E-46 | 98.03187482 |
| SLC29A2 | -1.810857745 | 2.362972952 | -15.89350088 | 6.45E-48 | 1.32E-46 | 97.82491342 |
| SNHG12 | 1.989460364 | 3.483279738 | 15.88823847 | 6.84E-48 | 1.40E-46 | 97.76559364 |
| C4orf47 | 2.026029953 | 3.783302591 | 15.88757425 | 6.90E-48 | 1.41E-46 | 97.7581069 |
| GINS2 | 1.314236138 | 4.019831842 | 15.88744896 | 6.91E-48 | 1.41E-46 | 97.7566947 |
| PYGL | 1.862647757 | 5.955342211 | 15.88662697 | 6.97E-48 | 1.42E-46 | 97.74742979 |
| PTTG1 | 1.867630473 | 3.229423628 | 15.88395629 | 7.18E-48 | 1.46E-46 | 97.71732932 |
| SIGLEC9 | 1.602948171 | 2.771734585 | 15.88236094 | 7.31E-48 | 1.49E-46 | 97.69934957 |
| ARHGAP15 | 1.389590276 | 3.476913654 | 15.87526798 | 7.93E-48 | 1.61E-46 | 97.61942044 |
| ATP6V1H | -1.513489871 | 4.818234275 | -15.864338 | 8.97E-48 | 1.82E-46 | 97.49628198 |
| UNC13D | 1.608799957 | 2.751508721 | 15.86302398 | 9.10E-48 | 1.84E-46 | 97.48148041 |
| BTC | -1.622278751 | 3.064947101 | -15.86268492 | 9.14E-48 | 1.85E-46 | 97.47766121 |
| FDX1 | -1.333795529 | 5.0484252 | -15.86027354 | 9.39E-48 | 1.90E-46 | 97.45050035 |
| IL12RB1 | 1.537698684 | 3.175713954 | 15.85837624 | 9.59E-48 | 1.93E-46 | 97.42913106 |
| FXYD5 | 1.842286631 | 6.948055029 | 15.84021778 | 1.18E-47 | 2.37E-46 | 97.22466649 |
| CHST11 | 1.651918443 | 4.035993607 | 15.83922539 | 1.19E-47 | 2.40E-46 | 97.21349499 |
| APOBEC3H | 1.667726189 | 2.216654707 | 15.83904451 | 1.19E-47 | 2.40E-46 | 97.21145884 |
| RAB7B | 1.404152311 | 3.491705237 | 15.81480787 | 1.57E-47 | 3.15E-46 | 96.9387158 |
| KCNMB2 | -1.160471909 | 2.589720391 | -15.81384138 | 1.59E-47 | 3.18E-46 | 96.92784323 |
| RPS19 | 1.282797205 | 9.302070187 | 15.80983113 | 1.66E-47 | 3.32E-46 | 96.88273273 |
| DEPTOR | -1.472558102 | 5.797956631 | -15.78860773 | 2.11E-47 | 4.22E-46 | 96.64407514 |
| CD3D | 2.601764582 | 4.497282275 | 15.78689379 | 2.15E-47 | 4.29E-46 | 96.62480766 |
| NEK6 | 2.348138639 | 6.151208449 | 15.78637941 | 2.16E-47 | 4.31E-46 | 96.61902543 |
| CNGA1 | -1.691539343 | 2.453493875 | -15.78539377 | 2.19E-47 | 4.35E-46 | 96.6079459 |
| RASD1 | -2.618312883 | 6.636732395 | -15.78108813 | 2.30E-47 | 4.57E-46 | 96.55954955 |
| HOXB8 | -2.498715272 | 3.300383991 | -15.76662528 | 2.70E-47 | 5.37E-46 | 96.39702497 |
| PNMT | -1.187824307 | 1.336982357 | -15.76276525 | 2.82E-47 | 5.60E-46 | 96.35365893 |
| FCGR2A | 1.797727865 | 5.705414706 | 15.76175459 | 2.86E-47 | 5.66E-46 | 96.3423053 |
| EFNA3 | 1.626837352 | 2.921319461 | 15.75808994 | 2.98E-47 | 5.89E-46 | 96.30113966 |
| RAB37 | 1.451141697 | 2.859920965 | 15.75454559 | 3.10E-47 | 6.12E-46 | 96.26132927 |
| DPEP1 | -3.55414303 | 3.881065477 | -15.74402855 | 3.49E-47 | 6.88E-46 | 96.14322336 |
| CASP4 | 1.170991 | 5.168407663 | 15.74015913 | 3.64E-47 | 7.18E-46 | 96.09977845 |
| TMEM72 | -3.293118514 | 5.205167408 | -15.73967734 | 3.66E-47 | 7.21E-46 | 96.09436933 |
| PACRG | -2.07248033 | 1.837425581 | -15.73071431 | 4.05E-47 | 7.97E-46 | 95.99375321 |
| TBX21 | 1.292126228 | 2.560880984 | 15.72589736 | 4.28E-47 | 8.40E-46 | 95.93968976 |
| KLHL6 | 1.500607615 | 2.897159546 | 15.72404548 | 4.37E-47 | 8.57E-46 | 95.91890694 |
| CSNK1E | 1.48191166 | 4.503577145 | 15.69876603 | 5.81E-47 | 1.14E-45 | 95.63531088 |
| NAP1L2 | -2.313562178 | 3.53340495 | -15.69579753 | 6.00E-47 | 1.17E-45 | 95.60202177 |
| CLEC2D | 1.570117576 | 3.21613458 | 15.68735072 | 6.60E-47 | 1.29E-45 | 95.50731285 |
| RASSF4 | 1.683092353 | 6.683868246 | 15.67979725 | 7.19E-47 | 1.40E-45 | 95.42263895 |
| TAF1D | 1.263070946 | 5.086538245 | 15.6740348 | 7.67E-47 | 1.49E-45 | 95.35805397 |
| PCYT2 | -1.207451813 | 4.963992576 | -15.66894158 | 8.12E-47 | 1.58E-45 | 95.3009782 |
| CTXN3 | -2.912683533 | 1.671949037 | -15.66368625 | 8.62E-47 | 1.68E-45 | 95.24209409 |
| SCN2B | -1.141897534 | 1.240623034 | -15.66022131 | 8.96E-47 | 1.74E-45 | 95.20327532 |
| EVI2B | 1.917694232 | 5.348482436 | 15.64485077 | 1.06E-46 | 2.06E-45 | 95.0311189 |
| UBE2C | 2.70044752 | 3.944838398 | 15.64476885 | 1.07E-46 | 2.06E-45 | 95.0302016 |
| PSORS1C1 | 1.358548099 | 2.624482684 | 15.63658022 | 1.17E-46 | 2.26E-45 | 94.93851551 |
| GMIP | 1.39531785 | 5.087017718 | 15.63390982 | 1.20E-46 | 2.33E-45 | 94.90862012 |
| SLA2 | 1.746934901 | 2.97192135 | 15.62900071 | 1.27E-46 | 2.46E-45 | 94.85366796 |
| PLAG1 | -1.200701077 | 1.273656409 | -15.62752587 | 1.29E-46 | 2.49E-45 | 94.83716015 |
| PLXNB1 | -1.396147791 | 4.347881435 | -15.62519969 | 1.33E-46 | 2.56E-45 | 94.81112476 |
| PML | 1.711414447 | 5.201316592 | 15.62497949 | 1.33E-46 | 2.56E-45 | 94.80866034 |
| NAV2 | -1.485398767 | 4.224464386 | -15.62033151 | 1.40E-46 | 2.69E-45 | 94.75664391 |
| FKBP10 | 2.423228783 | 6.082060485 | 15.61243218 | 1.53E-46 | 2.94E-45 | 94.66825629 |
| CGNL1 | -2.256379842 | 5.261577379 | -15.60713074 | 1.63E-46 | 3.12E-45 | 94.60894787 |
| TMC4 | -2.691727282 | 3.585024062 | -15.6055093 | 1.66E-46 | 3.17E-45 | 94.59081026 |
| IL10RA | 1.91566322 | 4.398768496 | 15.60346062 | 1.70E-46 | 3.24E-45 | 94.56789467 |
| HOXB5 | -1.752103612 | 2.529070686 | -15.60328279 | 1.70E-46 | 3.24E-45 | 94.56590552 |
| ADK | -1.000275176 | 5.11727212 | -15.60314596 | 1.70E-46 | 3.24E-45 | 94.56437512 |
| ENOX1 | -1.213409043 | 2.695709704 | -15.59402876 | 1.88E-46 | 3.59E-45 | 94.4624107 |
| PGF | 3.218211503 | 5.333007632 | 15.58362546 | 2.12E-46 | 4.03E-45 | 94.34609436 |
| BTN3A1 | 1.637556494 | 5.624028384 | 15.58124286 | 2.18E-46 | 4.13E-45 | 94.31945999 |
| TMEM140 | 1.293478301 | 7.127125185 | 15.56707693 | 2.55E-46 | 4.84E-45 | 94.16113951 |
| MCAM | 2.020193342 | 6.598183977 | 15.56385677 | 2.64E-46 | 5.01E-45 | 94.12515922 |
| CADPS2 | -1.54126776 | 4.280297191 | -15.56144013 | 2.72E-46 | 5.14E-45 | 94.09815913 |
| PADI2 | -2.084292458 | 3.721248586 | -15.55775156 | 2.83E-46 | 5.35E-45 | 94.05695193 |
| PPP1R1A | -3.987051757 | 4.149493552 | -15.55554108 | 2.90E-46 | 5.48E-45 | 94.03225934 |
| ETV7 | 1.915933093 | 3.989294187 | 15.54962111 | 3.10E-46 | 5.85E-45 | 93.96613673 |
| DLX5 | 1.650860092 | 2.126512463 | 15.54636194 | 3.22E-46 | 6.06E-45 | 93.92973836 |
| MAL2 | -2.962824962 | 5.133382978 | -15.54043731 | 3.44E-46 | 6.47E-45 | 93.86358077 |
| ALDH1A2 | -2.430482496 | 2.188377665 | -15.52830288 | 3.94E-46 | 7.40E-45 | 93.72811552 |
| RDH10 | -1.618357326 | 5.095544829 | -15.52822706 | 3.94E-46 | 7.40E-45 | 93.72726917 |
| ITGA5 | 2.101266683 | 6.271742446 | 15.51478999 | 4.58E-46 | 8.59E-45 | 93.57731576 |
| HLX | 1.668629565 | 2.791235348 | 15.50338486 | 5.21E-46 | 9.75E-45 | 93.45008271 |
| TNFSF13B | 2.09214506 | 4.963123065 | 15.50235956 | 5.27E-46 | 9.85E-45 | 93.43864675 |
| DUSP15 | -2.316534431 | 2.730438752 | -15.49616324 | 5.64E-46 | 1.05E-44 | 93.3695412 |
| KIAA1522 | -1.414119521 | 4.717553124 | -15.4957376 | 5.67E-46 | 1.06E-44 | 93.36479456 |
| SHISA3 | -3.023649662 | 4.017438615 | -15.4924066 | 5.89E-46 | 1.10E-44 | 93.32765047 |
| CYP2J2 | 3.922372096 | 6.663693262 | 15.49172676 | 5.93E-46 | 1.10E-44 | 93.32006992 |
| SPARC | 1.897702605 | 9.282909471 | 15.47935208 | 6.81E-46 | 1.27E-44 | 93.18211276 |
| HLA-DPB1 | 1.927891035 | 7.868200884 | 15.4737223 | 7.26E-46 | 1.35E-44 | 93.11936595 |
| BTG2 | -1.739318081 | 7.457066466 | -15.47196859 | 7.40E-46 | 1.37E-44 | 93.09982203 |
| PIK3R6 | 1.594010835 | 2.091759782 | 15.46541251 | 7.96E-46 | 1.48E-44 | 93.02676751 |
| SFTA2 | -1.56528504 | 0.936568275 | -15.44505032 | 1.00E-45 | 1.85E-44 | 92.79995805 |
| DIRAS3 | -1.731899071 | 3.035087528 | -15.44462759 | 1.00E-45 | 1.85E-44 | 92.79525076 |
| KRT7 | -3.308938917 | 2.824913171 | -15.44236744 | 1.03E-45 | 1.90E-44 | 92.77008396 |
| RAC2 | 2.004754974 | 5.718203974 | 15.41311216 | 1.43E-45 | 2.63E-44 | 92.44447238 |
| SYT1 | -1.261320699 | 1.152995511 | -15.40702082 | 1.53E-45 | 2.81E-44 | 92.37671003 |
| ZAP70 | 1.670573128 | 2.263751452 | 15.39103573 | 1.83E-45 | 3.35E-44 | 92.19894237 |
| RAB42 | 3.093652826 | 5.752020671 | 15.38487178 | 1.96E-45 | 3.59E-44 | 92.13041587 |
| CCL4 | 2.286585777 | 4.634460258 | 15.36871397 | 2.35E-45 | 4.29E-44 | 91.95084259 |
| CHAC1 | -1.651072067 | 2.853860869 | -15.36801414 | 2.36E-45 | 4.32E-44 | 91.94306675 |
| ADA | 1.635255754 | 3.840537269 | 15.36442967 | 2.46E-45 | 4.49E-44 | 91.90324223 |
| PRELID2 | 1.350141726 | 3.197472368 | 15.35595018 | 2.70E-45 | 4.93E-44 | 91.80904885 |
| SHROOM3 | -1.969969361 | 3.061430497 | -15.34525464 | 3.05E-45 | 5.55E-44 | 91.69027187 |
| SLC5A11 | -1.245439932 | 0.932101882 | -15.34300153 | 3.12E-45 | 5.68E-44 | 91.66525511 |
| CROT | -1.17664756 | 3.957350448 | -15.3339844 | 3.45E-45 | 6.27E-44 | 91.56515254 |
| FGFBP1 | -1.266983967 | 0.678610397 | -15.33100579 | 3.57E-45 | 6.47E-44 | 91.53209162 |
| MYO3A | 1.640422837 | 3.190837067 | 15.33023095 | 3.60E-45 | 6.52E-44 | 91.52349188 |
| HK3 | 1.556707876 | 2.876062702 | 15.32202013 | 3.95E-45 | 7.13E-44 | 91.43237321 |
| CAB39L | -1.401897839 | 4.071658116 | -15.32199523 | 3.95E-45 | 7.13E-44 | 91.43209699 |
| DNASE1L3 | -2.387819445 | 3.226710689 | -15.3117406 | 4.43E-45 | 7.98E-44 | 91.31832823 |
| UQCRC1 | -1.112837807 | 6.564108171 | -15.29355295 | 5.42E-45 | 9.75E-44 | 91.11663145 |
| ADAMTS7 | 1.547490323 | 3.491807087 | 15.29108069 | 5.57E-45 | 1.00E-43 | 91.08922303 |
| SH3BP2 | 1.533521603 | 6.438476569 | 15.288154 | 5.75E-45 | 1.03E-43 | 91.05677907 |
| SLC25A25 | -1.730353146 | 4.351371536 | -15.26940912 | 7.09E-45 | 1.27E-43 | 90.84904846 |
| OCLN | -1.976409406 | 3.553223634 | -15.26886429 | 7.13E-45 | 1.28E-43 | 90.84301229 |
| GZMM | 1.609187801 | 2.863605496 | 15.26759595 | 7.23E-45 | 1.29E-43 | 90.82896091 |
| TRIM2 | -1.781574599 | 4.621934312 | -15.26472546 | 7.47E-45 | 1.33E-43 | 90.797162 |
| PNCK | 4.086609121 | 4.463655479 | 15.26053658 | 7.82E-45 | 1.40E-43 | 90.75076291 |
| PTH1R | -2.876214404 | 4.343112061 | -15.25556005 | 8.27E-45 | 1.47E-43 | 90.69564676 |
| RAPGEF3 | -1.541144173 | 4.176630245 | -15.25430176 | 8.39E-45 | 1.49E-43 | 90.6817122 |
| GLRX5 | -1.220451625 | 5.007544247 | -15.23624977 | 1.02E-44 | 1.82E-43 | 90.48185784 |
| AOAH | 1.99187175 | 4.708937495 | 15.22889512 | 1.11E-44 | 1.97E-43 | 90.4004648 |
| ACADSB | -1.725965784 | 5.104597415 | -15.22832953 | 1.12E-44 | 1.98E-43 | 90.39420624 |
| ESRP2 | -1.419380266 | 3.907152651 | -15.22759443 | 1.13E-44 | 2.00E-43 | 90.38607211 |
| SH3GL3 | -1.284736751 | 1.379561649 | -15.22282221 | 1.19E-44 | 2.11E-43 | 90.33326998 |
| ASPHD1 | 2.108932319 | 3.386246824 | 15.20935187 | 1.38E-44 | 2.44E-43 | 90.18426845 |
| LDHD | -2.300755304 | 3.659296867 | -15.20687253 | 1.42E-44 | 2.51E-43 | 90.15684982 |
| SPHK2 | -1.024945487 | 3.748060678 | -15.20680862 | 1.42E-44 | 2.51E-43 | 90.15614308 |
| PLXNA4 | -1.225406352 | 2.555484339 | -15.19720376 | 1.58E-44 | 2.78E-43 | 90.04994392 |
| SFXN5 | -1.227960252 | 3.46054615 | -15.19470273 | 1.63E-44 | 2.85E-43 | 90.02229547 |
| PTPN13 | -1.658313474 | 3.822889784 | -15.18369177 | 1.84E-44 | 3.22E-43 | 89.9005958 |
| GOT2 | -1.045276563 | 6.58328553 | -15.18286622 | 1.85E-44 | 3.25E-43 | 89.89147297 |
| IL4R | 1.401304283 | 5.352246983 | 15.17386944 | 2.05E-44 | 3.58E-43 | 89.79206752 |
| LHFPL2 | 1.714816299 | 3.995743311 | 15.17347095 | 2.06E-44 | 3.60E-43 | 89.78766516 |
| TAP1 | 1.647412569 | 5.577217889 | 15.16639345 | 2.23E-44 | 3.88E-43 | 89.70948565 |
| MILR1 | 1.774156533 | 3.497732534 | 15.16287041 | 2.31E-44 | 4.03E-43 | 89.67057555 |
| IL16 | 1.336146683 | 3.537957545 | 15.16261748 | 2.32E-44 | 4.04E-43 | 89.66778219 |
| NUAK2 | -1.969724898 | 4.959656719 | -15.16055181 | 2.37E-44 | 4.13E-43 | 89.64497014 |
| PFKP | 2.014256119 | 6.96867598 | 15.15887847 | 2.42E-44 | 4.20E-43 | 89.62649174 |
| DCAF11 | -1.026819738 | 5.474896968 | -15.15468983 | 2.53E-44 | 4.39E-43 | 89.58024136 |
| FAM111B | 1.429672031 | 2.855157803 | 15.15155198 | 2.62E-44 | 4.55E-43 | 89.54559748 |
| PRR15L | -3.373241794 | 3.961531398 | -15.14014462 | 2.98E-44 | 5.15E-43 | 89.41968056 |
| TOX3 | -2.241210172 | 3.048651455 | -15.13069692 | 3.31E-44 | 5.71E-43 | 89.31542768 |
| APLN | 2.290397837 | 7.179822976 | 15.08851566 | 5.27E-44 | 9.08E-43 | 88.85033191 |
| IFNGR2 | 1.078751734 | 7.105887871 | 15.08436067 | 5.52E-44 | 9.49E-43 | 88.80455066 |
| CD3E | 2.484269961 | 5.398799304 | 15.08393434 | 5.55E-44 | 9.53E-43 | 88.79985359 |
| ARL11 | 1.104020907 | 2.905476393 | 15.08167555 | 5.69E-44 | 9.76E-43 | 88.77496816 |
| ABCC3 | 2.22725763 | 5.951521071 | 15.0799261 | 5.80E-44 | 9.94E-43 | 88.75569535 |
| WASF3 | -1.199187627 | 2.718150258 | -15.0705721 | 6.43E-44 | 1.10E-42 | 88.6526647 |
| UQCRFS1 | -1.305752499 | 5.390847089 | -15.06925625 | 6.53E-44 | 1.12E-42 | 88.63817357 |
| DOK2 | 1.665068764 | 4.28579584 | 15.05068214 | 8.01E-44 | 1.37E-42 | 88.4336826 |
| IL32 | 2.165697216 | 8.469717871 | 15.04679353 | 8.36E-44 | 1.43E-42 | 88.39088577 |
| STAT4 | 1.059937113 | 2.964536531 | 15.04448735 | 8.58E-44 | 1.46E-42 | 88.36550713 |
| LOX | 3.220574513 | 6.631137766 | 15.03407605 | 9.63E-44 | 1.64E-42 | 88.25095705 |
| INPP5D | 1.514500058 | 4.685841004 | 15.03220812 | 9.83E-44 | 1.67E-42 | 88.23040909 |
| PDGFRA | -2.323168988 | 2.925858739 | -15.03159086 | 9.89E-44 | 1.68E-42 | 88.22361918 |
| ANXA4 | 1.66615539 | 7.549551366 | 15.02979286 | 1.01E-43 | 1.71E-42 | 88.2038419 |
| EVI2A | 1.631460654 | 3.131579135 | 15.02531503 | 1.06E-43 | 1.79E-42 | 88.15459253 |
| ORAI3 | 1.13701614 | 5.383334941 | 15.02263919 | 1.09E-43 | 1.85E-42 | 88.12516543 |
| MRO | -1.695653686 | 2.004035405 | -15.02212659 | 1.10E-43 | 1.85E-42 | 88.11952847 |
| TSPAN33 | -1.583064048 | 5.929122209 | -14.999729 | 1.41E-43 | 2.37E-42 | 87.87331462 |
| TAGAP | 1.556078647 | 2.867233872 | 14.99404919 | 1.50E-43 | 2.52E-42 | 87.8109042 |
| ARHGAP30 | 1.786268164 | 4.758075187 | 14.98837578 | 1.59E-43 | 2.68E-42 | 87.74857513 |
| ZMYND15 | 1.184143503 | 2.412113761 | 14.98400409 | 1.67E-43 | 2.81E-42 | 87.70055453 |
| CENPM | 1.306585133 | 3.564004988 | 14.98365675 | 1.68E-43 | 2.82E-42 | 87.69673943 |
| LZTS1 | 2.028667926 | 4.094717541 | 14.98043069 | 1.74E-43 | 2.92E-42 | 87.6613075 |
| PPP1R3G | 1.449140045 | 3.387533308 | 14.96650893 | 2.03E-43 | 3.40E-42 | 87.50844475 |
| CCL11 | -1.962594305 | 1.476131519 | -14.96598789 | 2.04E-43 | 3.41E-42 | 87.502725 |
| LPAR5 | 1.596996328 | 2.807329879 | 14.96072238 | 2.16E-43 | 3.61E-42 | 87.44492719 |
| TRNP1 | -2.393685732 | 3.574644578 | -14.95637613 | 2.27E-43 | 3.79E-42 | 87.39722705 |
| PLEK | 2.034691459 | 4.969264787 | 14.95424915 | 2.32E-43 | 3.87E-42 | 87.37388572 |
| GNA15 | 1.674275209 | 3.480754632 | 14.95204775 | 2.38E-43 | 3.96E-42 | 87.34972931 |
| TOP2A | 1.844577292 | 4.313122008 | 14.94757692 | 2.50E-43 | 4.16E-42 | 87.30067527 |
| RHEBL1 | 1.134708984 | 1.94482495 | 14.94485258 | 2.58E-43 | 4.28E-42 | 87.27078705 |
| PLIN2 | 2.664041542 | 8.689438545 | 14.94266867 | 2.64E-43 | 4.38E-42 | 87.24682961 |
| CD6 | 1.536145271 | 2.452624357 | 14.94172766 | 2.67E-43 | 4.42E-42 | 87.23650733 |
| C3orf52 | -1.454730247 | 2.788784181 | -14.93597109 | 2.84E-43 | 4.71E-42 | 87.17336761 |
| RIN3 | 1.246539477 | 5.311887568 | 14.93094804 | 3.00E-43 | 4.97E-42 | 87.11828274 |
| CXCL9 | 2.878634777 | 4.939032403 | 14.91192291 | 3.70E-43 | 6.12E-42 | 86.90972357 |
| SLC34A1 | -3.224404058 | 2.822090653 | -14.90679929 | 3.92E-43 | 6.46E-42 | 86.85357832 |
| PTPRO | -1.584696625 | 2.185584077 | -14.90005025 | 4.22E-43 | 6.95E-42 | 86.7796352 |
| SLC14A1 | -2.117357164 | 4.079187453 | -14.90002587 | 4.22E-43 | 6.95E-42 | 86.77936813 |
| CD63 | 1.020509413 | 8.952956327 | 14.89981414 | 4.23E-43 | 6.96E-42 | 86.77704863 |
| PAG1 | 1.550700899 | 3.566188812 | 14.89506223 | 4.46E-43 | 7.32E-42 | 86.72499601 |
| TTYH3 | 1.932101149 | 6.706494327 | 14.88052678 | 5.23E-43 | 8.58E-42 | 86.56582282 |
| HAPLN3 | 1.692962087 | 3.768386083 | 14.87666227 | 5.45E-43 | 8.94E-42 | 86.52351599 |
| FER1L4 | 2.168843542 | 3.114238978 | 14.87084169 | 5.82E-43 | 9.51E-42 | 86.45980486 |
| XK | -1.430848739 | 2.440687311 | -14.86351023 | 6.30E-43 | 1.03E-41 | 86.3795726 |
| ZDHHC2 | -1.810233064 | 3.919066434 | -14.85619978 | 6.83E-43 | 1.11E-41 | 86.2995889 |
| TBC1D10C | 1.721695236 | 2.559645573 | 14.85405443 | 6.99E-43 | 1.14E-41 | 86.27612006 |
| TMEM86A | -1.311089067 | 4.5459852 | -14.85297302 | 7.08E-43 | 1.15E-41 | 86.26429069 |
| PNMA2 | 2.501244539 | 4.886147805 | 14.8497644 | 7.33E-43 | 1.19E-41 | 86.22919457 |
| KLC3 | -1.492999632 | 1.184455381 | -14.83978401 | 8.18E-43 | 1.33E-41 | 86.12005129 |
| MELK | 1.394201567 | 2.987232993 | 14.83130463 | 8.98E-43 | 1.46E-41 | 86.02734989 |
| SNX20 | 1.543981353 | 3.603092652 | 14.82658829 | 9.45E-43 | 1.53E-41 | 85.97579914 |
| FUT11 | 1.432774471 | 5.819165299 | 14.82589632 | 9.53E-43 | 1.54E-41 | 85.96823629 |
| CLDN10 | -2.252613989 | 5.414737838 | -14.80424985 | 1.21E-42 | 1.95E-41 | 85.73173949 |
| TRIM9 | 1.728152324 | 3.855741508 | 14.79405784 | 1.35E-42 | 2.18E-41 | 85.62044427 |
| HSPA6 | 1.57225783 | 2.856830443 | 14.7928049 | 1.37E-42 | 2.21E-41 | 85.60676485 |
| SLC1A3 | 2.161965846 | 3.664860334 | 14.79167505 | 1.39E-42 | 2.23E-41 | 85.59442978 |
| NUDT1 | 1.101421229 | 3.975357912 | 14.79056576 | 1.40E-42 | 2.26E-41 | 85.58231959 |
| PLCB2 | 1.647356158 | 3.895108888 | 14.78961683 | 1.42E-42 | 2.28E-41 | 85.57196044 |
| ITGB6 | -2.401658303 | 3.854829589 | -14.77917258 | 1.59E-42 | 2.55E-41 | 85.45796483 |
| ITGAM | 1.73102486 | 4.457136774 | 14.77784724 | 1.61E-42 | 2.58E-41 | 85.44350186 |
| BSPRY | -2.372779798 | 3.675067206 | -14.77712796 | 1.63E-42 | 2.60E-41 | 85.43565295 |
| CD4 | 1.731342093 | 5.317312737 | 14.77437444 | 1.68E-42 | 2.68E-41 | 85.40560747 |
| ABLIM3 | 2.265217583 | 4.95947515 | 14.77192979 | 1.72E-42 | 2.75E-41 | 85.37893454 |
| TRMT1 | 1.017970568 | 5.145500274 | 14.76397119 | 1.88E-42 | 3.00E-41 | 85.29211503 |
| ACSL4 | -1.36449723 | 6.10104521 | -14.76077425 | 1.95E-42 | 3.10E-41 | 85.25724612 |
| FAM151A | -2.969135655 | 3.521937276 | -14.75986714 | 1.96E-42 | 3.13E-41 | 85.24735304 |
| ANK2 | -2.259936464 | 4.175740989 | -14.75435716 | 2.09E-42 | 3.32E-41 | 85.1872662 |
| SUCLG2 | -1.184784793 | 6.588573645 | -14.7516751 | 2.15E-42 | 3.42E-41 | 85.15802204 |
| CD72 | 1.674711241 | 4.142136964 | 14.75094076 | 2.17E-42 | 3.44E-41 | 85.15001542 |
| UXS1 | -1.18689536 | 4.432876424 | -14.74968678 | 2.20E-42 | 3.48E-41 | 85.13634367 |
| EXOSC5 | 1.24422861 | 5.664931336 | 14.74860842 | 2.22E-42 | 3.52E-41 | 85.12458713 |
| CCBE1 | -2.305860136 | 1.328463041 | -14.74714963 | 2.26E-42 | 3.57E-41 | 85.10868362 |
| APOBEC3C | 1.923015009 | 6.823926688 | 14.74443355 | 2.33E-42 | 3.68E-41 | 85.0790754 |
| OLFML2A | 2.271571374 | 6.790169487 | 14.72039736 | 3.03E-42 | 4.77E-41 | 84.81716828 |
| CAPN12 | 2.070598776 | 3.24497246 | 14.7191685 | 3.07E-42 | 4.83E-41 | 84.80378363 |
| PPP1R3C | 1.783319219 | 6.020188367 | 14.71895284 | 3.07E-42 | 4.84E-41 | 84.80143469 |
| RNF166 | 1.091656905 | 3.331563051 | 14.7152136 | 3.20E-42 | 5.03E-41 | 84.76071092 |
| FGD2 | 1.480851898 | 2.833032996 | 14.70171639 | 3.71E-42 | 5.83E-41 | 84.61375485 |
| SERPINH1 | 1.421546913 | 6.863732183 | 14.69512602 | 3.99E-42 | 6.26E-41 | 84.54202319 |
| CD7 | 1.854809272 | 2.751335083 | 14.69207126 | 4.13E-42 | 6.46E-41 | 84.50877939 |
| CHSY3 | 1.556396156 | 2.37343847 | 14.69081383 | 4.18E-42 | 6.55E-41 | 84.49509625 |
| CD96 | 1.659424813 | 3.95816671 | 14.68658969 | 4.38E-42 | 6.85E-41 | 84.44913399 |
| CD48 | 1.616491899 | 3.77931059 | 14.6862664 | 4.40E-42 | 6.87E-41 | 84.44561662 |
| SFXN3 | 1.244829682 | 5.91176277 | 14.68533045 | 4.44E-42 | 6.93E-41 | 84.43543362 |
| BCAM | -1.787411565 | 6.561437996 | -14.68186619 | 4.61E-42 | 7.19E-41 | 84.3977458 |
| OPHN1 | -1.216628957 | 2.279049715 | -14.67101889 | 5.19E-42 | 8.09E-41 | 84.2797652 |
| MMP11 | 1.704215462 | 4.000236009 | 14.67071676 | 5.21E-42 | 8.11E-41 | 84.27647964 |
| CD84 | 2.068601899 | 3.592166445 | 14.66571755 | 5.50E-42 | 8.54E-41 | 84.22212046 |
| B2M | 1.296349919 | 11.52948235 | 14.66160876 | 5.75E-42 | 8.93E-41 | 84.17744992 |
| IPO13 | -1.064184516 | 5.193929505 | -14.6575968 | 6.01E-42 | 9.32E-41 | 84.13383795 |
| CCR5 | 2.15879679 | 5.213394247 | 14.6538883 | 6.26E-42 | 9.69E-41 | 84.09352992 |
| PIP5K1B | -1.556908952 | 2.317246608 | -14.65153535 | 6.42E-42 | 9.94E-41 | 84.06795804 |
| LIMD2 | 1.617726924 | 4.133262922 | 14.64930089 | 6.58E-42 | 1.02E-40 | 84.04367564 |
| IKZF1 | 1.59460017 | 3.474285292 | 14.6470249 | 6.75E-42 | 1.04E-40 | 84.01894386 |
| S1PR4 | 1.378897442 | 3.699746057 | 14.64252388 | 7.09E-42 | 1.09E-40 | 83.97003947 |
| FAM180A | -1.846902071 | 1.39776076 | -14.64057166 | 7.24E-42 | 1.11E-40 | 83.94883048 |
| PSTPIP1 | 1.610886092 | 3.701677498 | 14.63939207 | 7.33E-42 | 1.13E-40 | 83.93601612 |
| OR51E1 | 1.740430868 | 3.161565084 | 14.63484084 | 7.71E-42 | 1.18E-40 | 83.88657862 |
| ARHGAP24 | -1.624788123 | 4.680164919 | -14.63253818 | 7.90E-42 | 1.21E-40 | 83.86156894 |
| BIRC5 | 1.619092294 | 3.801198096 | 14.63200709 | 7.95E-42 | 1.22E-40 | 83.85580096 |
| SEC61G | 1.229604394 | 6.217337389 | 14.62495884 | 8.58E-42 | 1.31E-40 | 83.77926167 |
| RASSF8 | -1.36438965 | 4.833669493 | -14.61976396 | 9.08E-42 | 1.39E-40 | 83.72286007 |
| INO80E | 1.095239458 | 4.061834949 | 14.6013219 | 1.11E-41 | 1.69E-40 | 83.52271045 |
| CLEC7A | 1.803440753 | 4.341630524 | 14.59756306 | 1.16E-41 | 1.76E-40 | 83.48193122 |
| NDUFA4 | -1.058890185 | 8.040288365 | -14.58779675 | 1.29E-41 | 1.96E-40 | 83.37600132 |
| PALM | -1.534601667 | 3.86990779 | -14.57578972 | 1.47E-41 | 2.23E-40 | 83.24581486 |
| CDC45 | 1.400882596 | 1.850400589 | 14.57567772 | 1.47E-41 | 2.23E-40 | 83.24460078 |
| MYBL2 | 1.887426134 | 3.295817156 | 14.5739996 | 1.50E-41 | 2.27E-40 | 83.22640998 |
| NDNF | -3.40757868 | 4.265539851 | -14.57363359 | 1.50E-41 | 2.28E-40 | 83.22244252 |
| C21orf62 | -2.102727066 | 2.824637791 | -14.57350809 | 1.50E-41 | 2.28E-40 | 83.2210822 |
| HTATIP2 | 1.130298711 | 5.49707627 | 14.55491386 | 1.84E-41 | 2.78E-40 | 83.01959207 |
| GAB3 | 1.168664764 | 3.922544617 | 14.55487364 | 1.84E-41 | 2.78E-40 | 83.01915637 |
| EMILIN2 | 1.367370334 | 3.187485344 | 14.55459068 | 1.85E-41 | 2.79E-40 | 83.01609114 |
| DCN | -3.20889772 | 4.533146951 | -14.55041293 | 1.93E-41 | 2.91E-40 | 82.97083814 |
| CTSH | -1.261666623 | 5.037863001 | -14.54865131 | 1.97E-41 | 2.96E-40 | 82.95175839 |
| CDO1 | -1.608670013 | 1.493567086 | -14.54102732 | 2.14E-41 | 3.22E-40 | 82.86919711 |
| AGXT | -1.902965696 | 1.823665351 | -14.53408155 | 2.31E-41 | 3.47E-40 | 82.79399892 |
| CD244 | 1.526201711 | 2.062500125 | 14.52799774 | 2.47E-41 | 3.70E-40 | 82.72814698 |
| UHRF1 | 1.283491403 | 3.098116585 | 14.52691323 | 2.50E-41 | 3.74E-40 | 82.71640955 |
| N4BP2L1 | 1.176156722 | 4.278238307 | 14.52256407 | 2.62E-41 | 3.92E-40 | 82.66934372 |
| RAP2B | 1.184226962 | 4.653150153 | 14.51905786 | 2.72E-41 | 4.07E-40 | 82.63140523 |
| DTL | 1.386075339 | 3.726916258 | 14.50212756 | 3.27E-41 | 4.88E-40 | 82.44827583 |
| SIGLEC1 | 1.918049309 | 2.867678891 | 14.49856415 | 3.40E-41 | 5.06E-40 | 82.40974502 |
| IFI16 | 1.517991824 | 5.310876271 | 14.49638481 | 3.48E-41 | 5.18E-40 | 82.38618233 |
| CKM | -1.048824944 | 0.790958512 | -14.48918177 | 3.76E-41 | 5.60E-40 | 82.30831634 |
| SULT2B1 | -1.453282699 | 1.300575027 | -14.48084309 | 4.12E-41 | 6.12E-40 | 82.21819764 |
| ZC3HC1 | 1.113635251 | 5.599509285 | 14.47949649 | 4.18E-41 | 6.21E-40 | 82.20364695 |
| P4HA1 | 1.563014896 | 6.312345849 | 14.47911287 | 4.19E-41 | 6.23E-40 | 82.19950184 |
| SORD | -1.381596318 | 3.70929982 | -14.47199845 | 4.53E-41 | 6.71E-40 | 82.12263883 |
| IGFBP2 | -2.317962651 | 5.186479517 | -14.46466922 | 4.91E-41 | 7.26E-40 | 82.0434744 |
| RAB3B | -1.070184363 | 0.829320484 | -14.45989132 | 5.17E-41 | 7.63E-40 | 81.99187801 |
| VASH1 | 1.725436434 | 4.698282222 | 14.4333547 | 6.89E-41 | 1.02E-39 | 81.70546302 |
| TMEM116 | -1.144421682 | 4.253336588 | -14.43139801 | 7.04E-41 | 1.04E-39 | 81.68435429 |
| MAP4K1 | 1.51165287 | 2.394126994 | 14.41543216 | 8.37E-41 | 1.23E-39 | 81.51216844 |
| MBOAT2 | -1.393946462 | 2.955027428 | -14.41291493 | 8.60E-41 | 1.27E-39 | 81.48502963 |
| CD300C | 1.724576954 | 2.686071585 | 14.4095929 | 8.91E-41 | 1.31E-39 | 81.44921765 |
| LONRF2 | -1.306030196 | 2.424556164 | -14.40380918 | 9.49E-41 | 1.39E-39 | 81.38687811 |
| SLC30A8 | -1.058953312 | 0.652975884 | -14.39820625 | 1.01E-40 | 1.48E-39 | 81.32649909 |
| HSD3B7 | 1.907526266 | 5.234985933 | 14.39525318 | 1.04E-40 | 1.53E-39 | 81.29468048 |
| PAQR5 | -1.780618958 | 4.6401068 | -14.3936287 | 1.06E-40 | 1.55E-39 | 81.27717844 |
| DOC2A | 2.690926829 | 3.071810606 | 14.39185862 | 1.08E-40 | 1.58E-39 | 81.25810888 |
| SCIN | -2.641144808 | 4.165625995 | -14.3718214 | 1.34E-40 | 1.96E-39 | 81.04232352 |
| THOC6 | 1.015793071 | 5.163031786 | 14.37060938 | 1.36E-40 | 1.98E-39 | 81.02927583 |
| ATRNL1 | -1.481750239 | 1.254247943 | -14.36854386 | 1.39E-40 | 2.03E-39 | 81.00704116 |
| KIFC1 | 1.371786024 | 2.446628455 | 14.36324243 | 1.47E-40 | 2.14E-39 | 80.94998039 |
| LMBR1L | 1.18163886 | 3.70364933 | 14.36187819 | 1.49E-40 | 2.17E-39 | 80.93529833 |
| POLR3B | -1.007526913 | 3.192724941 | -14.35931909 | 1.54E-40 | 2.23E-39 | 80.90775907 |
| RGS10 | 1.531116936 | 5.05141676 | 14.35901891 | 1.54E-40 | 2.24E-39 | 80.90452885 |
| CPEB3 | -1.070542854 | 3.019209997 | -14.35022006 | 1.69E-40 | 2.46E-39 | 80.80986128 |
| CENPH | 1.068478168 | 3.727940416 | 14.34955893 | 1.71E-40 | 2.47E-39 | 80.80274924 |
| THSD7A | -1.765905595 | 4.561825889 | -14.34669168 | 1.76E-40 | 2.55E-39 | 80.77190726 |
| DLL4 | 2.18044075 | 5.646197337 | 14.33820251 | 1.93E-40 | 2.79E-39 | 80.68061007 |
| SIRPB2 | 1.394605874 | 2.890574651 | 14.33691393 | 1.96E-40 | 2.83E-39 | 80.66675434 |
| SLAMF6 | 2.055747365 | 3.444365593 | 14.31697185 | 2.43E-40 | 3.50E-39 | 80.4524022 |
| CNP | -1.070707394 | 5.8407931 | -14.31482522 | 2.48E-40 | 3.58E-39 | 80.4293375 |
| SCPEP1 | -1.178516445 | 6.09388391 | -14.30339738 | 2.81E-40 | 4.05E-39 | 80.30657913 |
| CSF2RA | 1.540652348 | 3.289808487 | 14.28223081 | 3.53E-40 | 5.08E-39 | 80.07933645 |
| SGK2 | -1.67123709 | 3.372184908 | -14.27306832 | 3.89E-40 | 5.60E-39 | 79.98102111 |
| GM2A | 1.373153537 | 6.506262663 | 14.26323329 | 4.33E-40 | 6.22E-39 | 79.87552444 |
| PEBP1 | -1.786286485 | 9.681625209 | -14.26130759 | 4.42E-40 | 6.34E-39 | 79.85487246 |
| ECHS1 | -1.119104964 | 9.209614544 | -14.26125745 | 4.42E-40 | 6.34E-39 | 79.85433472 |
| DEFB1 | -3.523924514 | 8.113584724 | -14.26050651 | 4.46E-40 | 6.39E-39 | 79.84628176 |
| SRL | -1.044279908 | 3.093766235 | -14.25750746 | 4.61E-40 | 6.59E-39 | 79.81412245 |
| EIF4EBP1 | 1.714057948 | 6.749522541 | 14.25057629 | 4.96E-40 | 7.10E-39 | 79.73981146 |
| HLA-DQB1 | 2.165220733 | 7.161320747 | 14.24481311 | 5.28E-40 | 7.55E-39 | 79.67803658 |
| CSRNP3 | -1.00274208 | 2.491685972 | -14.24302229 | 5.38E-40 | 7.69E-39 | 79.65884357 |
| HIGD1A | -1.440814383 | 6.489202882 | -14.23142833 | 6.10E-40 | 8.69E-39 | 79.53461527 |
| KLK1 | -3.120418951 | 1.310222954 | -14.23052075 | 6.16E-40 | 8.77E-39 | 79.52489271 |
| ST6GALNAC2 | -1.222998635 | 1.354213218 | -14.21414504 | 7.35E-40 | 1.04E-38 | 79.349521 |
| WNK1 | -1.382890103 | 5.456028657 | -14.2122407 | 7.50E-40 | 1.06E-38 | 79.32913359 |
| CDK18 | 2.097097053 | 7.339200414 | 14.20919863 | 7.75E-40 | 1.10E-38 | 79.29656877 |
| FCRL6 | 1.506939861 | 2.120197177 | 14.20209582 | 8.36E-40 | 1.18E-38 | 79.22054796 |
| OAS1 | 1.396332823 | 5.62006247 | 14.20001991 | 8.55E-40 | 1.21E-38 | 79.19833338 |
| CD8A | 2.671272048 | 4.948493104 | 14.19904159 | 8.64E-40 | 1.22E-38 | 79.18786478 |
| RELB | 1.165952404 | 5.432046386 | 14.19116912 | 9.40E-40 | 1.33E-38 | 79.10363809 |
| CXCL10 | 2.689395087 | 6.050039771 | 14.1862667 | 9.91E-40 | 1.40E-38 | 79.05119961 |
| BTBD16 | 1.622238557 | 3.04645624 | 14.1827808 | 1.03E-39 | 1.45E-38 | 79.01391846 |
| BAG1 | -1.21623092 | 5.228107549 | -14.17744564 | 1.09E-39 | 1.53E-38 | 78.95686875 |
| NCF1 | 1.272609295 | 3.633489265 | 14.17583551 | 1.11E-39 | 1.56E-38 | 78.93965348 |
| OGFR | 1.009283958 | 5.29129773 | 14.17350448 | 1.14E-39 | 1.60E-38 | 78.91473228 |
| SPC24 | 1.258455027 | 2.90950605 | 14.17114577 | 1.17E-39 | 1.64E-38 | 78.88951717 |
| TIMP1 | 2.063672209 | 8.354876624 | 14.16773873 | 1.21E-39 | 1.70E-38 | 78.85309902 |
| CRABP1 | -1.766998624 | 1.955277762 | -14.16321816 | 1.27E-39 | 1.78E-38 | 78.80478518 |
| S1PR5 | 1.423975785 | 3.809557434 | 14.14453532 | 1.55E-39 | 2.17E-38 | 78.6051944 |
| ABTB2 | -1.673419393 | 3.951090595 | -14.14309224 | 1.58E-39 | 2.20E-38 | 78.58978329 |
| ATP6V0E2 | -1.227627782 | 4.056281483 | -14.13793592 | 1.67E-39 | 2.32E-38 | 78.53472422 |
| MS4A14 | 1.337382086 | 3.292304653 | 14.13563197 | 1.71E-39 | 2.38E-38 | 78.51012609 |
| PYCARD | 1.566936188 | 4.744164591 | 14.13095846 | 1.79E-39 | 2.50E-38 | 78.4602354 |
| CEP55 | 1.552316497 | 3.194513584 | 14.12963613 | 1.82E-39 | 2.53E-38 | 78.44612075 |
| ANXA3 | -2.250784279 | 3.888024851 | -14.12946006 | 1.82E-39 | 2.53E-38 | 78.44424148 |
| GSTO2 | -1.773008282 | 2.369327009 | -14.12511326 | 1.91E-39 | 2.65E-38 | 78.39784849 |
| HRH2 | 2.605201116 | 5.12759329 | 14.11565934 | 2.11E-39 | 2.93E-38 | 78.29697303 |
| FPR3 | 2.12508966 | 4.510475546 | 14.10815272 | 2.29E-39 | 3.17E-38 | 78.21690018 |
| BATF3 | 1.811325705 | 2.907421439 | 14.104538 | 2.38E-39 | 3.29E-38 | 78.17834995 |
| AQP3 | -2.179848629 | 7.588878163 | -14.1029662 | 2.42E-39 | 3.35E-38 | 78.16158853 |
| USP46 | -1.156450029 | 3.849372976 | -14.09725796 | 2.58E-39 | 3.55E-38 | 78.10072495 |
| ADRB1 | -1.442918138 | 1.083032148 | -14.09561238 | 2.62E-39 | 3.61E-38 | 78.08318152 |
| DOK1 | 1.229736639 | 3.915411004 | 14.08170203 | 3.04E-39 | 4.19E-38 | 77.93492537 |
| PRMT6 | -1.422621823 | 4.666310332 | -14.07911606 | 3.13E-39 | 4.30E-38 | 77.90737248 |
| CDKN2C | 1.530096011 | 4.223128643 | 14.06662419 | 3.57E-39 | 4.91E-38 | 77.77431091 |
| KIF4A | 1.270396706 | 3.503151257 | 14.06418802 | 3.67E-39 | 5.03E-38 | 77.74836828 |
| NCF2 | 1.996092511 | 4.618053756 | 14.06093482 | 3.80E-39 | 5.21E-38 | 77.71372869 |
| MICAL1 | 1.271655911 | 4.312716275 | 14.05424931 | 4.08E-39 | 5.58E-38 | 77.64255534 |
| FABP7 | 5.126251462 | 5.507790218 | 14.0540098 | 4.09E-39 | 5.59E-38 | 77.64000585 |
| STOX1 | -1.340665234 | 2.79326817 | -14.04373578 | 4.57E-39 | 6.23E-38 | 77.5306644 |
| DDIT4 | 2.000114685 | 7.938426759 | 14.04226349 | 4.64E-39 | 6.32E-38 | 77.51499884 |
| RALBP1 | -1.209321391 | 5.799160752 | -14.03951797 | 4.78E-39 | 6.51E-38 | 77.48578811 |
| RALGDS | 1.03000083 | 2.863923995 | 14.03804482 | 4.85E-39 | 6.60E-38 | 77.47011592 |
| AURKB | 1.472578391 | 3.226547196 | 14.03249207 | 5.15E-39 | 7.00E-38 | 77.41105008 |
| IQGAP3 | 1.46407026 | 2.634784013 | 14.02987773 | 5.30E-39 | 7.19E-38 | 77.38324494 |
| CXCR3 | 1.975327143 | 4.257075833 | 14.02966475 | 5.31E-39 | 7.20E-38 | 77.38097985 |
| AMBP | -1.898585879 | 3.154243914 | -14.02647052 | 5.49E-39 | 7.45E-38 | 77.34701114 |
| MYO9B | 1.327113951 | 4.45019727 | 14.01196654 | 6.41E-39 | 8.69E-38 | 77.19281995 |
| APOBEC3F | 1.028109197 | 3.318551301 | 14.00917863 | 6.61E-39 | 8.94E-38 | 77.16319126 |
| HPGD | -2.185709993 | 3.399360678 | -14.00173167 | 7.15E-39 | 9.68E-38 | 77.084063 |
| CXCR6 | 1.807784995 | 3.731937178 | 13.99542784 | 7.65E-39 | 1.03E-37 | 77.0170982 |
| SLC27A3 | 1.296263578 | 4.626267982 | 13.98984206 | 8.12E-39 | 1.10E-37 | 76.95777422 |
| CTNNAL1 | -1.482027987 | 4.441623327 | -13.98966758 | 8.13E-39 | 1.10E-37 | 76.95592138 |
| LDLRAD3 | 1.305203917 | 4.396069671 | 13.97545815 | 9.47E-39 | 1.28E-37 | 76.80506566 |
| SLC27A4 | -1.002397728 | 4.709637162 | -13.97360032 | 9.66E-39 | 1.30E-37 | 76.78534769 |
| COL4A3 | -1.736069697 | 2.819377529 | -13.97308703 | 9.71E-39 | 1.31E-37 | 76.77990024 |
| HLA-C | 1.275564988 | 10.47649189 | 13.97231868 | 9.79E-39 | 1.32E-37 | 76.77174595 |
| MXD3 | 1.438345444 | 1.874564944 | 13.96613077 | 1.05E-38 | 1.40E-37 | 76.70608383 |
| SLAMF7 | 2.078686388 | 3.293825596 | 13.95633606 | 1.16E-38 | 1.56E-37 | 76.60217965 |
| GAL3ST1 | 2.489745234 | 7.069972698 | 13.94803482 | 1.27E-38 | 1.70E-37 | 76.51414811 |
| APOBR | 1.408344478 | 3.050243943 | 13.94019865 | 1.38E-38 | 1.84E-37 | 76.43107337 |
| CTSS | 1.810332339 | 6.003298264 | 13.93943237 | 1.39E-38 | 1.85E-37 | 76.422951 |
| CCDC127 | 1.105547503 | 4.427914609 | 13.93917259 | 1.39E-38 | 1.86E-37 | 76.42019745 |
| MKI67 | 1.562804186 | 2.935648258 | 13.92783036 | 1.57E-38 | 2.09E-37 | 76.30000101 |
| BCL2A1 | 1.793781543 | 4.230510516 | 13.92492379 | 1.62E-38 | 2.16E-37 | 76.26920751 |
| GABRP | -1.619667421 | 1.897262171 | -13.91823032 | 1.74E-38 | 2.32E-37 | 76.1983067 |
| TRIP13 | 1.285359571 | 3.06463314 | 13.91719427 | 1.76E-38 | 2.34E-37 | 76.18733392 |
| RASGRP4 | 1.027920607 | 2.576295954 | 13.91358127 | 1.83E-38 | 2.43E-37 | 76.14907196 |
| CD33 | 1.243198384 | 2.804334587 | 13.91302967 | 1.84E-38 | 2.44E-37 | 76.14323097 |
| HCAR1 | -1.288459673 | 2.61348502 | -13.91018845 | 1.90E-38 | 2.51E-37 | 76.11314645 |
| UCHL1 | -3.017146876 | 4.581025623 | -13.90904085 | 1.92E-38 | 2.54E-37 | 76.10099594 |
| PRSS35 | -1.714375659 | 2.525379286 | -13.89878021 | 2.14E-38 | 2.83E-37 | 75.99238163 |
| IL11 | -1.148383618 | 1.352325244 | -13.89303182 | 2.28E-38 | 3.01E-37 | 75.93155017 |
| CDC6 | 1.164587088 | 2.59457108 | 13.87440136 | 2.78E-38 | 3.66E-37 | 75.73448666 |
| CMTM3 | 1.42216107 | 6.015811762 | 13.87347213 | 2.80E-38 | 3.70E-37 | 75.72466129 |
| TPD52L1 | -1.593633673 | 4.019237426 | -13.86804029 | 2.97E-38 | 3.91E-37 | 75.66723408 |
| FOXM1 | 1.679250597 | 2.801064973 | 13.86769522 | 2.98E-38 | 3.92E-37 | 75.66358633 |
| PROCR | 1.531315682 | 5.041773232 | 13.85985742 | 3.24E-38 | 4.26E-37 | 75.58074432 |
| IRF2BPL | -1.393087221 | 4.560998915 | -13.85763858 | 3.32E-38 | 4.36E-37 | 75.55729663 |
| GAS5 | 1.434407062 | 5.737781781 | 13.85113541 | 3.55E-38 | 4.67E-37 | 75.48858549 |
| PARD3B | -1.206561995 | 3.417645135 | -13.8473076 | 3.70E-38 | 4.86E-37 | 75.44814961 |
| SNHG1 | 1.333373016 | 3.973223156 | 13.84625658 | 3.74E-38 | 4.91E-37 | 75.43704786 |
| PLAT | -1.673570473 | 5.248074228 | -13.84571507 | 3.76E-38 | 4.93E-37 | 75.43132828 |
| SORT1 | -1.490162974 | 5.04315466 | -13.82758421 | 4.56E-38 | 5.97E-37 | 75.23988998 |
| CES4A | 2.420128026 | 4.128550135 | 13.82450179 | 4.71E-38 | 6.16E-37 | 75.2073568 |
| DOCK2 | 1.859545303 | 3.004367835 | 13.82401939 | 4.74E-38 | 6.19E-37 | 75.20226566 |
| CDKN2B | 1.540853173 | 3.293161974 | 13.82171087 | 4.86E-38 | 6.34E-37 | 75.17790352 |
| TMEM44 | 1.426548959 | 4.379762717 | 13.80938119 | 5.53E-38 | 7.22E-37 | 75.04782255 |
| PYHIN1 | 1.56492706 | 3.086021241 | 13.80739799 | 5.65E-38 | 7.37E-37 | 75.02690506 |
| CD53 | 1.770884695 | 5.884285958 | 13.80040454 | 6.09E-38 | 7.93E-37 | 74.95315532 |
| GAPDH | 1.253113197 | 10.69476453 | 13.78790984 | 6.95E-38 | 9.04E-37 | 74.82144094 |
| SMPDL3A | 1.791166111 | 7.114879095 | 13.78775512 | 6.96E-38 | 9.05E-37 | 74.81981034 |
| KLRB1 | 1.444191315 | 4.098466414 | 13.78246061 | 7.36E-38 | 9.56E-37 | 74.76401684 |
| EHBP1L1 | 1.370178292 | 4.414846176 | 13.77824871 | 7.69E-38 | 9.99E-37 | 74.71964007 |
| TACC3 | 1.304349775 | 3.270990149 | 13.77641401 | 7.85E-38 | 1.02E-36 | 74.70031183 |
| LDHB | -1.276840907 | 3.884549556 | -13.77411921 | 8.04E-38 | 1.04E-36 | 74.67613842 |
| PILRA | 1.388598986 | 4.430909263 | 13.77256978 | 8.17E-38 | 1.06E-36 | 74.65981787 |
| SKA3 | 1.054323674 | 2.670100503 | 13.77211584 | 8.21E-38 | 1.06E-36 | 74.65503666 |
| ATPAF1 | -1.007821231 | 4.844432755 | -13.77181297 | 8.24E-38 | 1.06E-36 | 74.65184666 |
| REEP4 | 1.068517602 | 4.953749507 | 13.7703596 | 8.36E-38 | 1.08E-36 | 74.63653926 |
| RDH8 | -1.027490047 | 1.298407512 | -13.76666507 | 8.70E-38 | 1.12E-36 | 74.59763117 |
| ERBB2 | -1.51330272 | 6.315442622 | -13.76299402 | 9.04E-38 | 1.17E-36 | 74.55897585 |
| PHF21B | -1.118278625 | 1.277660613 | -13.76228413 | 9.11E-38 | 1.17E-36 | 74.55150149 |
| DISC1 | 1.012346538 | 3.267730312 | 13.76083794 | 9.25E-38 | 1.19E-36 | 74.53627529 |
| SPN | 1.80322838 | 3.042825946 | 13.75993458 | 9.34E-38 | 1.20E-36 | 74.52676479 |
| PPARGC1A | -2.067442146 | 4.156867703 | -13.75719026 | 9.62E-38 | 1.24E-36 | 74.49787459 |
| TSPAN6 | -1.153853904 | 5.762675966 | -13.75441413 | 9.90E-38 | 1.27E-36 | 74.46865275 |
| PNP | -1.319660171 | 5.578877612 | -13.7502336 | 1.03E-37 | 1.33E-36 | 74.4246539 |
| WNK3 | -1.008392467 | 1.691684969 | -13.74762686 | 1.06E-37 | 1.36E-36 | 74.39722227 |
| MRPL33 | -1.148538184 | 5.68966806 | -13.74650515 | 1.08E-37 | 1.38E-36 | 74.38541893 |
| IRF7 | 1.494587217 | 4.437911531 | 13.73941113 | 1.16E-37 | 1.48E-36 | 74.31078337 |
| HOGA1 | -1.874507476 | 5.030351745 | -13.73622365 | 1.20E-37 | 1.53E-36 | 74.27725473 |
| USP53 | -1.380671157 | 3.485299087 | -13.71929681 | 1.44E-37 | 1.83E-36 | 74.09927331 |
| KCNE3 | 1.854844306 | 5.220319863 | 13.7185389 | 1.45E-37 | 1.84E-36 | 74.09130681 |
| C3AR1 | 1.815035616 | 4.96606205 | 13.71106056 | 1.57E-37 | 1.99E-36 | 74.01271343 |
| ACAD8 | -1.026338647 | 3.533428412 | -13.70412452 | 1.68E-37 | 2.14E-36 | 73.93983967 |
| MICALL2 | 1.456519874 | 3.391631059 | 13.70103798 | 1.74E-37 | 2.21E-36 | 73.90741705 |
| PEPD | -1.339376308 | 6.614418677 | -13.70049786 | 1.75E-37 | 2.22E-36 | 73.90174384 |
| PLEKHG2 | 1.460163796 | 4.258596394 | 13.69979933 | 1.76E-37 | 2.24E-36 | 73.89440685 |
| KLHL3 | -1.393429321 | 2.559586963 | -13.69390664 | 1.88E-37 | 2.38E-36 | 73.8325209 |
| SLC43A3 | 1.378924665 | 4.02359392 | 13.69372766 | 1.88E-37 | 2.38E-36 | 73.83064142 |
| STRA8 | 1.918201958 | 2.934834358 | 13.68186829 | 2.13E-37 | 2.69E-36 | 73.70613636 |
| IL4I1 | 1.785913673 | 4.262674834 | 13.67982362 | 2.18E-37 | 2.75E-36 | 73.68467624 |
| BIK | -1.329644789 | 2.096211222 | -13.65904129 | 2.71E-37 | 3.42E-36 | 73.46665007 |
| CHRDL1 | -2.629671489 | 2.954755693 | -13.65554196 | 2.81E-37 | 3.55E-36 | 73.42995622 |
| ALDOB | -4.561458843 | 5.78467941 | -13.64862573 | 3.02E-37 | 3.81E-36 | 73.3574476 |
| SREBF2 | -1.207861176 | 5.521025639 | -13.62905986 | 3.72E-37 | 4.67E-36 | 73.15242893 |
| DAG1 | -1.129434054 | 6.022252031 | -13.62776765 | 3.77E-37 | 4.73E-36 | 73.13889416 |
| TBC1D4 | -1.402477732 | 4.708529321 | -13.62349839 | 3.94E-37 | 4.95E-36 | 73.09418243 |
| UBE2L6 | 1.075268595 | 6.635103345 | 13.62162691 | 4.02E-37 | 5.04E-36 | 73.07458492 |
| GTSE1 | 1.197804794 | 2.400239177 | 13.62056312 | 4.06E-37 | 5.10E-36 | 73.06344594 |
| CPA1 | -1.306114324 | 0.946590926 | -13.61902649 | 4.13E-37 | 5.17E-36 | 73.04735659 |
| SEZ6L2 | 2.360228317 | 4.986314482 | 13.61867031 | 4.15E-37 | 5.19E-36 | 73.04362734 |
| FABP5 | 1.52976513 | 4.121886943 | 13.61755518 | 4.20E-37 | 5.25E-36 | 73.03195206 |
| NT5DC3 | 1.773785061 | 3.432347454 | 13.61191666 | 4.45E-37 | 5.56E-36 | 72.97292545 |
| LPXN | 1.235393121 | 4.411693765 | 13.60974442 | 4.55E-37 | 5.69E-36 | 72.95018896 |
| CD5 | 1.596531699 | 4.600076508 | 13.60873103 | 4.60E-37 | 5.74E-36 | 72.93958272 |
| SH2B2 | 1.104338998 | 3.199746653 | 13.60405101 | 4.84E-37 | 6.03E-36 | 72.89060627 |
| TAC1 | -1.071959624 | 2.090738038 | -13.60337762 | 4.87E-37 | 6.07E-36 | 72.88355991 |
| MPP5 | -1.42361254 | 3.245826315 | -13.59912704 | 5.09E-37 | 6.33E-36 | 72.83908659 |
| SGPP1 | -1.245015074 | 4.709043392 | -13.59397813 | 5.38E-37 | 6.68E-36 | 72.78522415 |
| SH2D2A | 1.65669383 | 4.032544423 | 13.59025377 | 5.59E-37 | 6.94E-36 | 72.74627071 |
| BARX2 | 2.123020742 | 5.862318153 | 13.5845207 | 5.94E-37 | 7.36E-36 | 72.68631916 |
| BAZ1A | 1.256108466 | 4.752363383 | 13.58176413 | 6.11E-37 | 7.57E-36 | 72.65749813 |
| ACBD7 | -1.04138505 | 1.423870649 | -13.57595528 | 6.50E-37 | 8.04E-36 | 72.5967747 |
| NR1H3 | 1.376055122 | 4.888382042 | 13.57502126 | 6.56E-37 | 8.11E-36 | 72.58701215 |
| GPR27 | -1.651392928 | 2.379159204 | -13.56913008 | 6.98E-37 | 8.62E-36 | 72.5254446 |
| HLA-DMA | 1.417543303 | 7.127361404 | 13.56365046 | 7.39E-37 | 9.12E-36 | 72.46819108 |
| TCIRG1 | 1.321159819 | 5.424243304 | 13.54735779 | 8.77E-37 | 1.08E-35 | 72.29803192 |
| KIF13B | -1.418401818 | 4.725395505 | -13.54005872 | 9.47E-37 | 1.17E-35 | 72.22183684 |
| NRP2 | 1.650467079 | 3.973254524 | 13.53620087 | 9.86E-37 | 1.21E-35 | 72.18157367 |
| LRRC25 | 1.912596441 | 4.33243622 | 13.53289293 | 1.02E-36 | 1.25E-35 | 72.14705472 |
| TBX3 | -1.534832402 | 3.746377749 | -13.53075616 | 1.04E-36 | 1.28E-35 | 72.12475954 |
| MYH14 | -2.108485039 | 3.358096433 | -13.5272531 | 1.08E-36 | 1.33E-35 | 72.08821259 |
| AP3S1 | 1.039406675 | 6.288236664 | 13.52650998 | 1.09E-36 | 1.34E-35 | 72.08046034 |
| CD74 | 1.617917853 | 10.18175475 | 13.52317412 | 1.13E-36 | 1.39E-35 | 72.04566353 |
| KIF21B | 1.144116753 | 2.54044676 | 13.52245296 | 1.14E-36 | 1.39E-35 | 72.03814164 |
| RBCK1 | 1.038543601 | 6.928986425 | 13.51352684 | 1.25E-36 | 1.53E-35 | 71.9450576 |
| HES4 | 1.810851417 | 4.965013351 | 13.51348409 | 1.25E-36 | 1.53E-35 | 71.94461188 |
| SPC25 | 1.17892041 | 2.910948881 | 13.51242191 | 1.27E-36 | 1.55E-35 | 71.93353737 |
| NR2C2AP | 1.044738927 | 4.224291412 | 13.49944183 | 1.45E-36 | 1.77E-35 | 71.79824304 |
| SBK1 | -1.172920209 | 1.676225913 | -13.49898826 | 1.46E-36 | 1.78E-35 | 71.79351662 |
| MNDA | 1.902980137 | 5.530765558 | 13.49201419 | 1.57E-36 | 1.91E-35 | 71.72085473 |
| ABCG1 | 1.260158233 | 5.362917877 | 13.49181477 | 1.57E-36 | 1.91E-35 | 71.71877734 |
| ITPRIPL1 | 1.109715729 | 2.23808171 | 13.48998432 | 1.60E-36 | 1.95E-35 | 71.69970967 |
| GCNT4 | -1.491336318 | 3.441930004 | -13.48943317 | 1.61E-36 | 1.95E-35 | 71.69396863 |
| IL18BP | 1.306916616 | 4.981743239 | 13.48912273 | 1.61E-36 | 1.96E-35 | 71.69073499 |
| SNX33 | 1.211160112 | 5.7224837 | 13.48851501 | 1.63E-36 | 1.97E-35 | 71.68440504 |
| LRRC43 | -1.240597097 | 1.399302058 | -13.48733303 | 1.65E-36 | 1.99E-35 | 71.67209389 |
| NLRC4 | 1.14199309 | 3.470346037 | 13.48383715 | 1.71E-36 | 2.07E-35 | 71.63568527 |
| MAN1A1 | -1.458022269 | 6.02785209 | -13.48349696 | 1.71E-36 | 2.07E-35 | 71.63214258 |
| LCP1 | 1.90117218 | 6.640492783 | 13.48159589 | 1.75E-36 | 2.11E-35 | 71.61234598 |
| ARNT2 | -1.825128249 | 4.462973545 | -13.46880386 | 2.00E-36 | 2.41E-35 | 71.47917651 |
| DDX41 | 1.146109388 | 5.83378865 | 13.46751041 | 2.02E-36 | 2.44E-35 | 71.46571515 |
| TNFRSF18 | 1.385252176 | 2.511614379 | 13.46232166 | 2.14E-36 | 2.58E-35 | 71.41172087 |
| EBF2 | 1.771596838 | 2.826899358 | 13.45788474 | 2.24E-36 | 2.70E-35 | 71.36555924 |
| FASLG | 1.632997465 | 3.904258884 | 13.43544404 | 2.83E-36 | 3.41E-35 | 71.13221356 |
| RPL38 | 1.059931392 | 7.107269843 | 13.41313342 | 3.57E-36 | 4.29E-35 | 70.90043133 |
| BCKDHB | -1.23391662 | 5.044099601 | -13.41084433 | 3.66E-36 | 4.39E-35 | 70.87666215 |
| KIF20A | 1.492707619 | 3.627853071 | 13.40756108 | 3.79E-36 | 4.54E-35 | 70.84257386 |
| GPR65 | 1.319641167 | 3.305789302 | 13.40432129 | 3.92E-36 | 4.70E-35 | 70.8089413 |
| EVPL | -1.314634891 | 2.933634971 | -13.40131719 | 4.04E-36 | 4.84E-35 | 70.7777594 |
| HMGN5 | -1.23186952 | 4.262422944 | -13.40098829 | 4.06E-36 | 4.85E-35 | 70.7743457 |
| SLC39A4 | -1.476303237 | 3.953067682 | -13.39947522 | 4.12E-36 | 4.93E-35 | 70.75864203 |
| MS4A4A | 1.783451797 | 4.581250006 | 13.38860451 | 4.62E-36 | 5.51E-35 | 70.64584702 |
| TGFBR3 | -1.429123925 | 5.033365848 | -13.38498791 | 4.79E-36 | 5.72E-35 | 70.60833207 |
| HPSE2 | -1.043674971 | 1.137778901 | -13.37782969 | 5.16E-36 | 6.16E-35 | 70.53409642 |
| PTPN4 | -1.123521413 | 3.458694508 | -13.37690094 | 5.21E-36 | 6.21E-35 | 70.52446621 |
| PARD6A | -1.119215867 | 3.052913863 | -13.37303415 | 5.43E-36 | 6.46E-35 | 70.48437558 |
| PPM1E | -1.102692588 | 1.60451047 | -13.37273753 | 5.45E-36 | 6.48E-35 | 70.48130048 |
| OGDHL | -2.50851058 | 4.643202836 | -13.36956456 | 5.63E-36 | 6.69E-35 | 70.44840854 |
| SLC15A3 | 1.354127391 | 4.874672756 | 13.3685867 | 5.69E-36 | 6.75E-35 | 70.43827256 |
| BCL2L10 | -1.674861374 | 1.741233957 | -13.36483273 | 5.91E-36 | 7.02E-35 | 70.39936485 |
| ARG2 | -2.521269486 | 4.106137923 | -13.36380684 | 5.98E-36 | 7.09E-35 | 70.38873313 |
| HCP5 | 1.207945982 | 4.951772143 | 13.36140218 | 6.13E-36 | 7.26E-35 | 70.36381435 |
| RASSF5 | 1.590011251 | 4.976866786 | 13.3498564 | 6.91E-36 | 8.18E-35 | 70.24420356 |
| MTMR10 | -1.039611381 | 5.620991945 | -13.34828647 | 7.03E-36 | 8.31E-35 | 70.22794398 |
| ACAP1 | 1.218641165 | 3.973265663 | 13.33988002 | 7.67E-36 | 9.07E-35 | 70.14089717 |
| CD276 | 1.150915151 | 6.025216888 | 13.33376223 | 8.17E-36 | 9.64E-35 | 70.07756781 |
| HAPLN1 | 2.060917552 | 2.589266456 | 13.3294152 | 8.55E-36 | 1.01E-34 | 70.03257855 |
| ALS2CL | -1.563963506 | 2.588338036 | -13.32654424 | 8.81E-36 | 1.04E-34 | 70.00287021 |
| PALM3 | -1.940499782 | 4.121250773 | -13.31919266 | 9.51E-36 | 1.12E-34 | 69.92681313 |
| C6orf223 | 2.541943133 | 4.825051999 | 13.31499664 | 9.93E-36 | 1.17E-34 | 69.88341279 |
| HPCAL1 | 1.625288565 | 6.774295455 | 13.31461301 | 9.97E-36 | 1.17E-34 | 69.8794452 |
| SIT1 | 1.841756145 | 3.818561338 | 13.31131333 | 1.03E-35 | 1.21E-34 | 69.84532171 |
| LILRA6 | 1.406109647 | 2.207318031 | 13.31005754 | 1.05E-35 | 1.23E-34 | 69.83233628 |
| PPFIA4 | 2.014481461 | 3.912635152 | 13.30127722 | 1.15E-35 | 1.34E-34 | 69.74156271 |
| KISS1R | 2.906871799 | 2.8638728 | 13.29115682 | 1.27E-35 | 1.49E-34 | 69.63697598 |
| MYH10 | -1.796524012 | 4.911661983 | -13.29046239 | 1.28E-35 | 1.50E-34 | 69.62980117 |
| UCN | 1.458485888 | 2.56544774 | 13.28404732 | 1.37E-35 | 1.60E-34 | 69.56353101 |
| HMGCS2 | -3.802527253 | 4.97767361 | -13.26806457 | 1.62E-35 | 1.89E-34 | 69.39850012 |
| MOXD1 | -2.383385109 | 3.204354145 | -13.26629533 | 1.65E-35 | 1.92E-34 | 69.38023851 |
| POU2F2 | 1.230488326 | 2.553398299 | 13.26532711 | 1.66E-35 | 1.94E-34 | 69.37024539 |
| S100A5 | -1.004640707 | 1.055752281 | -13.26508265 | 1.67E-35 | 1.94E-34 | 69.36772229 |
| PLCXD3 | -1.587966217 | 1.815380761 | -13.2605336 | 1.75E-35 | 2.03E-34 | 69.32077687 |
| GBP5 | 2.06259542 | 4.593026391 | 13.26021871 | 1.75E-35 | 2.04E-34 | 69.31752754 |
| OXCT1 | -2.010577993 | 4.560680009 | -13.25942681 | 1.77E-35 | 2.05E-34 | 69.30935633 |
| TLCD1 | -1.403534239 | 4.371272643 | -13.25224613 | 1.91E-35 | 2.21E-34 | 69.23527437 |
| ARID5A | 1.337834066 | 4.555081166 | 13.25070464 | 1.94E-35 | 2.24E-34 | 69.21937405 |
| AFAP1L2 | -1.523923952 | 4.614522008 | -13.24942078 | 1.96E-35 | 2.27E-34 | 69.20613185 |
| SAT2 | -1.113088749 | 6.079315856 | -13.24547631 | 2.04E-35 | 2.36E-34 | 69.16545171 |
| SLC22A15 | -1.153019574 | 3.298619002 | -13.23907967 | 2.18E-35 | 2.52E-34 | 69.09949623 |
| PLD4 | 1.773186614 | 4.096559461 | 13.23791192 | 2.21E-35 | 2.55E-34 | 69.08745744 |
| RASA3 | 1.318838853 | 5.376028222 | 13.2351021 | 2.28E-35 | 2.63E-34 | 69.05849255 |
| NPNT | -1.993798368 | 5.196758918 | -13.23487429 | 2.28E-35 | 2.63E-34 | 69.05614432 |
| PRR7 | 1.352009057 | 3.7363076 | 13.23045858 | 2.39E-35 | 2.75E-34 | 69.01063246 |
| FDXR | 1.248130577 | 4.139189688 | 13.22044679 | 2.65E-35 | 3.05E-34 | 68.90747426 |
| TSPYL5 | -1.53224416 | 3.593967798 | -13.21712019 | 2.74E-35 | 3.15E-34 | 68.87320763 |
| FJX1 | 1.329944861 | 3.919977761 | 13.2153961 | 2.79E-35 | 3.20E-34 | 68.85545006 |
| FAM193B | 1.724284517 | 5.532772624 | 13.21480893 | 2.81E-35 | 3.22E-34 | 68.84940272 |
| NAT2 | -1.144687618 | 2.165231252 | -13.21389711 | 2.84E-35 | 3.25E-34 | 68.84001205 |
| CTSZ | 1.136512754 | 9.17219652 | 13.21202411 | 2.89E-35 | 3.31E-34 | 68.82072343 |
| SEMA6A | 1.752991648 | 5.45551597 | 13.21038437 | 2.94E-35 | 3.36E-34 | 68.80383825 |
| PLEKHN1 | 1.569084552 | 2.15524942 | 13.20988296 | 2.96E-35 | 3.38E-34 | 68.79867523 |
| RPL18 | 1.482363705 | 8.155044042 | 13.20519172 | 3.10E-35 | 3.54E-34 | 68.75037471 |
| ZNF205 | 1.011538283 | 4.770090933 | 13.19969158 | 3.28E-35 | 3.75E-34 | 68.69375808 |
| SYTL5 | -1.109233049 | 1.050891428 | -13.19764238 | 3.35E-35 | 3.83E-34 | 68.67266765 |
| RHOH | 1.33066976 | 3.585437205 | 13.19690245 | 3.38E-35 | 3.85E-34 | 68.66505275 |
| E2F2 | 1.021964145 | 2.22696754 | 13.18635225 | 3.77E-35 | 4.29E-34 | 68.55650207 |
| RPL37 | 1.021986801 | 7.885752693 | 13.184044 | 3.86E-35 | 4.39E-34 | 68.53275906 |
| ATF4 | 1.358666705 | 7.911159578 | 13.18135308 | 3.97E-35 | 4.51E-34 | 68.50508279 |
| STK33 | -1.358262681 | 2.213096183 | -13.17723045 | 4.14E-35 | 4.70E-34 | 68.46268736 |
| UGT8 | -1.867461103 | 5.725598899 | -13.17221237 | 4.36E-35 | 4.95E-34 | 68.41109355 |
| PTGER3 | -2.698914074 | 4.466571917 | -13.17053392 | 4.44E-35 | 5.03E-34 | 68.39383892 |
| ADAMTS15 | -1.502730853 | 3.648146223 | -13.16035969 | 4.93E-35 | 5.59E-34 | 68.28927288 |
| CADM4 | -2.032344621 | 4.111672541 | -13.16010678 | 4.95E-35 | 5.60E-34 | 68.2866742 |
| PLIN5 | -1.147508342 | 1.94923055 | -13.14735972 | 5.64E-35 | 6.38E-34 | 68.15573126 |
| TGFB1 | 1.495801022 | 7.507194157 | 13.14535336 | 5.76E-35 | 6.51E-34 | 68.13512751 |
| CAMKK1 | 1.107490004 | 3.558609234 | 13.13555195 | 6.37E-35 | 7.20E-34 | 68.03450055 |
| SCGB2A1 | -1.592039115 | 3.120600467 | -13.12743393 | 6.93E-35 | 7.82E-34 | 67.95118809 |
| HLA-G | 2.686538172 | 5.03206209 | 13.12464922 | 7.13E-35 | 8.04E-34 | 67.92261632 |
| TMEM233 | 1.631206759 | 3.071615492 | 13.11770031 | 7.66E-35 | 8.64E-34 | 67.85133349 |
| GAPT | 1.514969615 | 3.371811326 | 13.11511853 | 7.87E-35 | 8.86E-34 | 67.82485472 |
| CLK4 | 1.31502308 | 3.61411164 | 13.1104306 | 8.26E-35 | 9.29E-34 | 67.77678269 |
| FH | -1.056542915 | 6.833032134 | -13.10891981 | 8.39E-35 | 9.43E-34 | 67.76129247 |
| FHOD1 | 1.144975533 | 5.852694722 | 13.10075185 | 9.12E-35 | 1.02E-33 | 67.67756334 |
| CSK | 1.490605524 | 5.221719106 | 13.09842598 | 9.34E-35 | 1.05E-33 | 67.65372638 |
| NINL | -1.1132109 | 4.176186076 | -13.0973476 | 9.45E-35 | 1.06E-33 | 67.64267534 |
| ZNF683 | 1.418899253 | 1.869822545 | 13.09465118 | 9.71E-35 | 1.09E-33 | 67.61504498 |
| PHLDA3 | 1.449740796 | 5.83610848 | 13.08913056 | 1.03E-34 | 1.15E-33 | 67.55848508 |
| CYGB | 1.869307257 | 5.174122179 | 13.08414657 | 1.08E-34 | 1.21E-33 | 67.50743455 |
| RRM2 | 1.388072198 | 2.663431535 | 13.08327628 | 1.09E-34 | 1.22E-33 | 67.49852142 |
| CLEC12A | 1.434888113 | 2.931248648 | 13.0770767 | 1.16E-34 | 1.30E-33 | 67.43503738 |
| HLA-DQA1 | 1.966058163 | 6.596656575 | 13.07143295 | 1.23E-34 | 1.38E-33 | 67.37725998 |
| RCN1 | 1.159201449 | 4.68850455 | 13.07100391 | 1.24E-34 | 1.38E-33 | 67.37286823 |
| COL6A2 | 1.84338338 | 8.081928386 | 13.06459435 | 1.32E-34 | 1.48E-33 | 67.30726918 |
| TNFRSF1B | 1.358940804 | 6.267508206 | 13.05831206 | 1.41E-34 | 1.57E-33 | 67.24299029 |
| ATG16L2 | 1.563168868 | 3.828282733 | 13.05733695 | 1.43E-34 | 1.59E-33 | 67.2330148 |
| AKNA | 1.28171779 | 4.232346951 | 13.05264649 | 1.50E-34 | 1.66E-33 | 67.18503662 |
| GDF6 | 2.298304972 | 3.757722027 | 13.05145318 | 1.52E-34 | 1.68E-33 | 67.17283186 |
| VSIG1 | 1.497086582 | 3.011000178 | 13.0483495 | 1.56E-34 | 1.74E-33 | 67.14109171 |
| ZFAND2A | 1.13859847 | 4.752713833 | 13.04654997 | 1.59E-34 | 1.77E-33 | 67.12269053 |
| VSNL1 | -1.109040895 | 1.826165348 | -13.0461725 | 1.60E-34 | 1.77E-33 | 67.11883087 |
| CD99 | 1.23860906 | 6.285120315 | 13.02997735 | 1.89E-34 | 2.09E-33 | 66.95329397 |
| SLC22A7 | -2.214387051 | 2.237629652 | -13.02910314 | 1.91E-34 | 2.11E-33 | 66.94436171 |
| ENAM | -1.763488766 | 3.061611253 | -13.02676729 | 1.95E-34 | 2.16E-33 | 66.92049661 |
| PDIA5 | 1.489425169 | 6.555556169 | 13.02043827 | 2.08E-34 | 2.30E-33 | 66.855846 |
| SLC25A5 | -1.383539194 | 8.231427463 | -13.01282149 | 2.25E-34 | 2.48E-33 | 66.77806467 |
| KCNE4 | 1.929353985 | 5.148574939 | 13.01029847 | 2.31E-34 | 2.55E-33 | 66.75230566 |
| RUNX1 | 1.599936632 | 5.116315649 | 13.00291045 | 2.50E-34 | 2.74E-33 | 66.67689338 |
| UGT3A2 | -1.276064823 | 1.723324508 | -13.00230158 | 2.51E-34 | 2.76E-33 | 66.67067952 |
| ANKRD9 | -1.293186606 | 2.860104871 | -12.99760659 | 2.63E-34 | 2.89E-33 | 66.62276996 |
| MAMDC2 | -1.356005178 | 2.021776333 | -12.99028701 | 2.84E-34 | 3.11E-33 | 66.54809764 |
| CYP39A1 | -1.685757732 | 2.319557802 | -12.98663592 | 2.95E-34 | 3.23E-33 | 66.5108592 |
| SLC2A3 | 1.938355523 | 5.963977213 | 12.98411239 | 3.03E-34 | 3.31E-33 | 66.48512447 |
| HVCN1 | 1.036580974 | 3.844823616 | 12.97892688 | 3.19E-34 | 3.48E-33 | 66.43225221 |
| FCGR2C | 1.607188109 | 3.091610111 | 12.97860609 | 3.20E-34 | 3.49E-33 | 66.42898169 |
| IL15RA | 1.208293447 | 5.01483224 | 12.97739329 | 3.24E-34 | 3.53E-33 | 66.4166177 |
| LLGL2 | -1.254575983 | 4.728116153 | -12.97601089 | 3.29E-34 | 3.58E-33 | 66.40252551 |
| ALOX5 | 1.949023523 | 5.534589425 | 12.97574912 | 3.30E-34 | 3.59E-33 | 66.39985712 |
| SYNJ2BP | -1.151717915 | 4.235802488 | -12.97059301 | 3.48E-34 | 3.78E-33 | 66.34730385 |
| PLAU | -1.77466217 | 6.027909199 | -12.97021545 | 3.49E-34 | 3.79E-33 | 66.34345609 |
| RNASE6 | 1.648009226 | 6.258582453 | 12.96568001 | 3.65E-34 | 3.97E-33 | 66.29723951 |
| TYRO3 | -1.151382495 | 1.916529365 | -12.96320687 | 3.75E-34 | 4.07E-33 | 66.27204194 |
| GNG7 | -1.23076246 | 4.000820866 | -12.96049918 | 3.85E-34 | 4.18E-33 | 66.24445769 |
| DIRAS2 | 2.173019788 | 4.787865043 | 12.96014333 | 3.87E-34 | 4.19E-33 | 66.24083279 |
| RPL12 | 1.131523751 | 8.812324433 | 12.9551998 | 4.07E-34 | 4.40E-33 | 66.19048062 |
| ADAMTS4 | 2.023637665 | 4.950804703 | 12.94925455 | 4.32E-34 | 4.67E-33 | 66.12993991 |
| MSR1 | 1.839134425 | 4.377861777 | 12.93992552 | 4.76E-34 | 5.14E-33 | 66.03497409 |
| GRB14 | -1.766754905 | 4.123969527 | -12.93349608 | 5.08E-34 | 5.48E-33 | 65.96954778 |
| PRR11 | 1.436504752 | 2.533124862 | 12.92919954 | 5.31E-34 | 5.72E-33 | 65.92583631 |
| PSORS1C3 | 2.454101365 | 4.154678331 | 12.92841501 | 5.35E-34 | 5.76E-33 | 65.9178557 |
| SAMHD1 | 1.414341704 | 5.271065288 | 12.92841302 | 5.35E-34 | 5.76E-33 | 65.91783541 |
| C1orf54 | 1.127125072 | 4.878478066 | 12.91636449 | 6.05E-34 | 6.51E-33 | 65.79530665 |
| LRRK1 | 1.309603945 | 2.829376039 | 12.91597723 | 6.08E-34 | 6.53E-33 | 65.79136945 |
| HJURP | 1.261757123 | 2.681963716 | 12.91388862 | 6.21E-34 | 6.67E-33 | 65.77013614 |
| DUSP4 | 1.384804991 | 3.612756649 | 12.90653134 | 6.69E-34 | 7.19E-33 | 65.69535587 |
| SLA | 1.55298274 | 3.663687453 | 12.90577191 | 6.75E-34 | 7.24E-33 | 65.68763838 |
| SLC44A4 | -2.522332066 | 3.997702254 | -12.90360507 | 6.90E-34 | 7.39E-33 | 65.66561977 |
| KIAA0895L | 1.644141428 | 4.257445357 | 12.90000364 | 7.16E-34 | 7.67E-33 | 65.62902807 |
| GRAMD1C | -1.462338291 | 2.973128953 | -12.89919031 | 7.22E-34 | 7.72E-33 | 65.62076521 |
| MCM5 | 1.209593805 | 4.550798109 | 12.89585372 | 7.47E-34 | 7.99E-33 | 65.5868709 |
| IL21R | 1.295339993 | 2.581444435 | 12.8822502 | 8.58E-34 | 9.17E-33 | 65.44873326 |
| DPT | -1.898664902 | 1.783790464 | -12.87924681 | 8.85E-34 | 9.45E-33 | 65.41824651 |
| GPRIN1 | 1.184192199 | 2.830876747 | 12.87853054 | 8.91E-34 | 9.51E-33 | 65.41097645 |
| CCND1 | 1.936710985 | 8.935923627 | 12.87079303 | 9.64E-34 | 1.03E-32 | 65.33245595 |
| TBX19 | 1.07406927 | 3.385278417 | 12.85691923 | 1.11E-33 | 1.18E-32 | 65.19173228 |
| STMN3 | 1.809828394 | 6.382644816 | 12.85458951 | 1.14E-33 | 1.21E-32 | 65.16811028 |
| ATP6V1A | -1.395659137 | 6.43719804 | -12.85349728 | 1.15E-33 | 1.22E-32 | 65.15703648 |
| CDKL1 | -1.344102039 | 3.700287488 | -12.85149335 | 1.17E-33 | 1.25E-32 | 65.13672069 |
| FOLH1B | -1.153311585 | 1.483200988 | -12.84489111 | 1.26E-33 | 1.33E-32 | 65.06980037 |
| TCL6 | -1.311231978 | 2.00259072 | -12.84134392 | 1.30E-33 | 1.38E-32 | 65.03385428 |
| NDUFS1 | -1.206646476 | 4.789728201 | -12.84099184 | 1.31E-33 | 1.38E-32 | 65.03028668 |
| BRCC3 | -1.019455168 | 4.988434301 | -12.83791499 | 1.35E-33 | 1.43E-32 | 64.99911192 |
| MZT2A | 1.301897502 | 5.479934664 | 12.83693992 | 1.36E-33 | 1.44E-32 | 64.98923338 |
| PCSK6 | 2.059033317 | 4.18769102 | 12.8298128 | 1.46E-33 | 1.55E-32 | 64.91704095 |
| ZMYND12 | -1.155609636 | 3.619575971 | -12.82586469 | 1.52E-33 | 1.61E-32 | 64.87705956 |
| PSAT1 | -2.624439665 | 4.617798056 | -12.82398801 | 1.55E-33 | 1.64E-32 | 64.85805737 |
| STAB1 | 1.610124871 | 4.741005677 | 12.82386483 | 1.56E-33 | 1.64E-32 | 64.85681021 |
| SH3GL1 | 1.065444523 | 6.331740711 | 12.82252233 | 1.58E-33 | 1.66E-32 | 64.84321789 |
| COX4I2 | 2.164177892 | 4.770013184 | 12.82204838 | 1.58E-33 | 1.67E-32 | 64.83841957 |
| CTDSPL | -1.156513478 | 5.420521337 | -12.8134765 | 1.73E-33 | 1.82E-32 | 64.75165382 |
| RIN1 | 1.221684175 | 3.292305256 | 12.80142764 | 1.95E-33 | 2.05E-32 | 64.62975045 |
| FGR | 1.314582886 | 4.473706935 | 12.80113939 | 1.96E-33 | 2.06E-32 | 64.62683489 |
| RAPGEFL1 | 1.021835305 | 2.879100569 | 12.80083173 | 1.97E-33 | 2.06E-32 | 64.6237231 |
| ITGA4 | 1.542706873 | 3.394287468 | 12.80041188 | 1.97E-33 | 2.07E-32 | 64.61947662 |
| SALL3 | -1.248290144 | 0.783155364 | -12.79204012 | 2.15E-33 | 2.25E-32 | 64.5348189 |
| SLFN11 | 1.521279715 | 4.360884178 | 12.7883514 | 2.23E-33 | 2.33E-32 | 64.49752774 |
| COL4A1 | 1.771508353 | 6.996461988 | 12.78345964 | 2.35E-33 | 2.45E-32 | 64.44808393 |
| DNAH11 | 2.107731023 | 3.444461549 | 12.77386881 | 2.59E-33 | 2.70E-32 | 64.351176 |
| FADS3 | 1.098404441 | 4.642634877 | 12.7654348 | 2.82E-33 | 2.94E-32 | 64.26599158 |
| HOXA7 | -1.257732945 | 1.945382023 | -12.76088327 | 2.95E-33 | 3.08E-32 | 64.22003432 |
| PXK | -1.259890863 | 4.054307368 | -12.75306311 | 3.20E-33 | 3.33E-32 | 64.14109545 |
| LTBR | 1.138464663 | 5.68981365 | 12.74357443 | 3.52E-33 | 3.66E-32 | 64.04535201 |
| SERPINB9 | 1.454541106 | 4.583561422 | 12.72370616 | 4.30E-33 | 4.47E-32 | 63.84500987 |
| FCHO1 | 1.297439172 | 3.177865887 | 12.71527586 | 4.69E-33 | 4.86E-32 | 63.76005784 |
| MFAP3L | -1.572376561 | 4.828359039 | -12.71440315 | 4.73E-33 | 4.90E-32 | 63.75126545 |
| FBP1 | -2.089108904 | 5.778358988 | -12.71280445 | 4.81E-33 | 4.97E-32 | 63.73515969 |
| AK7 | -1.180917326 | 3.158316345 | -12.70778044 | 5.06E-33 | 5.23E-32 | 63.68455415 |
| HUNK | -1.430397359 | 3.264260325 | -12.69405841 | 5.81E-33 | 6.00E-32 | 63.54639542 |
| ASAP3 | -1.127304372 | 5.085626491 | -12.6926982 | 5.89E-33 | 6.08E-32 | 63.53270504 |
| CDKN3 | 1.144025142 | 3.071887386 | 12.6918617 | 5.94E-33 | 6.13E-32 | 63.52428615 |
| RPL36 | 1.090886565 | 8.569976831 | 12.69100158 | 5.99E-33 | 6.18E-32 | 63.51562991 |
| CD27 | 1.987402095 | 4.664481797 | 12.6887502 | 6.13E-33 | 6.31E-32 | 63.49297364 |
| ABCA1 | 1.506502973 | 4.73101113 | 12.68547003 | 6.34E-33 | 6.52E-32 | 63.45996862 |
| NRG3 | 1.166614273 | 3.147429134 | 12.68053751 | 6.66E-33 | 6.85E-32 | 63.41034702 |
| PAQR4 | 1.097795847 | 3.136569095 | 12.67915245 | 6.76E-33 | 6.95E-32 | 63.39641519 |
| NTRK2 | -1.957668806 | 3.359478288 | -12.67813494 | 6.83E-33 | 7.01E-32 | 63.38618103 |
| CD200 | 1.457872343 | 6.45972809 | 12.67578928 | 6.99E-33 | 7.18E-32 | 63.36259008 |
| STK10 | 1.247335341 | 4.838701543 | 12.67534144 | 7.02E-33 | 7.20E-32 | 63.35808636 |
| FBXO17 | 1.461212043 | 6.632076186 | 12.6664916 | 7.68E-33 | 7.87E-32 | 63.26910584 |
| OAT | -1.214206914 | 5.260259822 | -12.66552805 | 7.75E-33 | 7.95E-32 | 63.25942012 |
| PPP1R13L | 1.44139573 | 5.432516376 | 12.66292479 | 7.96E-33 | 8.15E-32 | 63.23325375 |
| PREX1 | 1.293369053 | 5.887444085 | 12.65994573 | 8.21E-33 | 8.39E-32 | 63.20331409 |
| LNX1 | -1.368374394 | 2.844440522 | -12.65976359 | 8.22E-33 | 8.40E-32 | 63.20148371 |
| BIRC3 | 2.043566021 | 5.632464106 | 12.65906907 | 8.28E-33 | 8.45E-32 | 63.19450434 |
| PTPN22 | 1.325519993 | 2.756212043 | 12.65754224 | 8.41E-33 | 8.58E-32 | 63.17916187 |
| GPRC5A | -2.247727947 | 3.516539282 | -12.65565836 | 8.57E-33 | 8.74E-32 | 63.16023293 |
| GRB10 | 1.456832136 | 6.872196698 | 12.65386206 | 8.73E-33 | 8.89E-32 | 63.14218567 |
| SLC2A1 | 1.863007429 | 6.435183249 | 12.65378619 | 8.73E-33 | 8.89E-32 | 63.14142341 |
| HLA-DQB2 | 2.321742041 | 4.368810137 | 12.64561534 | 9.48E-33 | 9.65E-32 | 63.05935054 |
| PCCA | -1.357713856 | 3.931784538 | -12.63965272 | 1.01E-32 | 1.02E-31 | 62.99947806 |
| MCOLN3 | -1.345836218 | 2.685048155 | -12.62636522 | 1.15E-32 | 1.17E-31 | 62.866114 |
| CXCL11 | 2.129544185 | 3.886325149 | 12.62050538 | 1.22E-32 | 1.24E-31 | 62.80732609 |
| PTGDR | 1.033060389 | 2.621171686 | 12.61910153 | 1.24E-32 | 1.26E-31 | 62.79324459 |
| BTBD19 | 1.628415041 | 3.932078348 | 12.61435084 | 1.30E-32 | 1.32E-31 | 62.745599 |
| PLXND1 | 1.452989648 | 6.415711781 | 12.61345909 | 1.31E-32 | 1.33E-31 | 62.73665667 |
| TNFRSF1A | 1.011792796 | 8.089356793 | 12.61021638 | 1.36E-32 | 1.37E-31 | 62.70414232 |
| PTPRC | 1.874157498 | 5.338898891 | 12.60466026 | 1.43E-32 | 1.45E-31 | 62.64844308 |
| IL17RE | -1.338817976 | 2.440213925 | -12.59800137 | 1.53E-32 | 1.55E-31 | 62.58170781 |
| BUB1 | 1.406249662 | 1.953231029 | 12.59526441 | 1.58E-32 | 1.59E-31 | 62.55428403 |
| CCNB2 | 1.288327146 | 2.4442653 | 12.5935934 | 1.60E-32 | 1.61E-31 | 62.53754262 |
| GATA2 | -1.540397943 | 3.528251535 | -12.59019116 | 1.66E-32 | 1.67E-31 | 62.50346053 |
| EMB | 1.560137639 | 3.773618473 | 12.58324664 | 1.78E-32 | 1.79E-31 | 62.43391027 |
| TNK2 | 1.093007446 | 4.100980622 | 12.57639412 | 1.91E-32 | 1.91E-31 | 62.36530365 |
| CRB2 | -1.575781329 | 2.852041391 | -12.56984494 | 2.04E-32 | 2.04E-31 | 62.29975456 |
| SLC38A4 | -2.022358012 | 2.113880188 | -12.5680793 | 2.07E-32 | 2.08E-31 | 62.28208628 |
| MARVELD3 | -1.272038418 | 2.74174254 | -12.56662097 | 2.10E-32 | 2.11E-31 | 62.26749419 |
| C1QTNF7 | -1.288052019 | 1.972216849 | -12.56095472 | 2.23E-32 | 2.23E-31 | 62.21080703 |
| IKZF3 | 1.524980611 | 2.846684993 | 12.55837662 | 2.29E-32 | 2.28E-31 | 62.18501984 |
| PDE1B | 1.208678263 | 2.657120356 | 12.55019369 | 2.48E-32 | 2.48E-31 | 62.10319168 |
| SPCS1 | -1.340045651 | 5.233541026 | -12.54393558 | 2.64E-32 | 2.64E-31 | 62.04063273 |
| CD3G | 1.623895721 | 2.779788351 | 12.53713906 | 2.83E-32 | 2.82E-31 | 61.9727126 |
| CD180 | 1.649346477 | 2.438934717 | 12.53480775 | 2.90E-32 | 2.88E-31 | 61.94941992 |
| ALAD | -1.209551629 | 4.972016047 | -12.53083975 | 3.01E-32 | 3.00E-31 | 61.90978065 |
| MITD1 | 1.354755325 | 4.864353745 | 12.52057363 | 3.34E-32 | 3.32E-31 | 61.80725941 |
| EOMES | 1.727042686 | 2.385891186 | 12.50627044 | 3.86E-32 | 3.83E-31 | 61.66450577 |
| SIPA1 | 1.130210901 | 5.404462841 | 12.50586125 | 3.87E-32 | 3.85E-31 | 61.6604232 |
| ZHX3 | -1.112499593 | 4.828568706 | -12.49781 | 4.20E-32 | 4.16E-31 | 61.58011151 |
| MFSD3 | -1.071230544 | 3.843190853 | -12.49558176 | 4.29E-32 | 4.26E-31 | 61.55789012 |
| SUSD1 | -1.435931956 | 3.497598911 | -12.49391118 | 4.37E-32 | 4.32E-31 | 61.5412317 |
| P4HB | 1.147583854 | 8.81945128 | 12.49359963 | 4.38E-32 | 4.34E-31 | 61.53812512 |
| IL3RA | 1.306572727 | 5.103136094 | 12.4840569 | 4.82E-32 | 4.76E-31 | 61.44299484 |
| SLC29A4 | 1.718050867 | 4.906948969 | 12.48393548 | 4.83E-32 | 4.76E-31 | 61.44178468 |
| HPCA | 1.112030571 | 2.968485792 | 12.47829047 | 5.11E-32 | 5.04E-31 | 61.38553091 |
| PSMB10 | 1.234613656 | 4.869804794 | 12.47586981 | 5.23E-32 | 5.16E-31 | 61.36141319 |
| JAG2 | 1.444909527 | 5.059618772 | 12.46964585 | 5.57E-32 | 5.48E-31 | 61.29941485 |
| AXL | 1.485399946 | 5.38353516 | 12.46208797 | 6.01E-32 | 5.90E-31 | 61.22415379 |
| LILRA2 | 1.030549309 | 1.829966163 | 12.45905434 | 6.19E-32 | 6.08E-31 | 61.19395274 |
| RPL18A | 1.048596998 | 7.554805112 | 12.45708028 | 6.32E-32 | 6.20E-31 | 61.1743025 |
| MIR155HG | 2.203549189 | 3.2005323 | 12.44878838 | 6.86E-32 | 6.72E-31 | 61.09178321 |
| HLA-E | 1.120870746 | 9.613507376 | 12.44878051 | 6.86E-32 | 6.72E-31 | 61.09170497 |
| TNNI2 | 1.215041797 | 1.738573519 | 12.44566541 | 7.08E-32 | 6.93E-31 | 61.06071263 |
| GNRH1 | 1.33450726 | 2.524152198 | 12.43154552 | 8.15E-32 | 7.97E-31 | 60.92029112 |
| ATP1B1 | -1.347433651 | 9.577517342 | -12.42922076 | 8.35E-32 | 8.15E-31 | 60.89718069 |
| LRRC19 | -2.363979815 | 4.316237757 | -12.42154265 | 9.01E-32 | 8.78E-31 | 60.82087092 |
| BLNK | -1.166540748 | 3.549599603 | -12.4205002 | 9.11E-32 | 8.87E-31 | 60.81051251 |
| TRPA1 | 1.778070402 | 2.136041968 | 12.41983141 | 9.17E-32 | 8.92E-31 | 60.80386736 |
| TNFSF8 | 1.521087198 | 2.530032712 | 12.41358518 | 9.76E-32 | 9.49E-31 | 60.74181438 |
| DLGAP5 | 1.378728367 | 1.866385432 | 12.40418997 | 1.07E-31 | 1.04E-30 | 60.64851329 |
| POU2F3 | -1.062398657 | 0.775242347 | -12.40369866 | 1.08E-31 | 1.05E-30 | 60.64363542 |
| MYOZ1 | -1.318862545 | 2.305244732 | -12.39764638 | 1.14E-31 | 1.11E-30 | 60.58355586 |
| NCAPG | 1.087941124 | 2.51418804 | 12.3943441 | 1.18E-31 | 1.15E-30 | 60.55078226 |
| RPL22L1 | 1.12214348 | 4.413600062 | 12.38580776 | 1.29E-31 | 1.25E-30 | 60.46608744 |
| CRTAM | 1.607777408 | 2.100112302 | 12.38529159 | 1.29E-31 | 1.25E-30 | 60.46096737 |
| CHEK2 | 1.068191721 | 3.21769673 | 12.3700073 | 1.51E-31 | 1.45E-30 | 60.30941291 |
| SAMD14 | 1.141303988 | 2.008721377 | 12.36922331 | 1.52E-31 | 1.47E-30 | 60.30164217 |
| TMEM45A | 2.196854917 | 4.367942256 | 12.36787971 | 1.54E-31 | 1.48E-30 | 60.2883253 |
| ENTPD1 | 1.091184408 | 4.480089914 | 12.36176453 | 1.64E-31 | 1.58E-30 | 60.22772688 |
| SLC41A2 | 1.104974493 | 5.153405389 | 12.35778953 | 1.70E-31 | 1.64E-30 | 60.1883462 |
| C16orf54 | 1.24594924 | 3.443936908 | 12.35178641 | 1.81E-31 | 1.74E-30 | 60.12888726 |
| BNIP3 | 1.394569132 | 6.294068797 | 12.35047234 | 1.83E-31 | 1.76E-30 | 60.11587411 |
| SH2D1A | 1.443220209 | 2.866942488 | 12.34145058 | 2.00E-31 | 1.92E-30 | 60.02655505 |
| VEPH1 | -1.384765792 | 3.433396349 | -12.3309445 | 2.22E-31 | 2.13E-30 | 59.92259008 |
| SLC11A1 | 1.271274824 | 3.456705861 | 12.33021453 | 2.24E-31 | 2.15E-30 | 59.91536857 |
| VDR | -1.416897629 | 5.015280006 | -12.32758682 | 2.30E-31 | 2.20E-30 | 59.8893748 |
| SLC16A10 | -1.207056829 | 2.755489576 | -12.31906441 | 2.50E-31 | 2.39E-30 | 59.80509279 |
| SLC5A3 | -1.783850229 | 5.358018699 | -12.31851496 | 2.52E-31 | 2.40E-30 | 59.79966029 |
| SCD5 | -2.328813405 | 5.035870482 | -12.31306403 | 2.66E-31 | 2.53E-30 | 59.74577344 |
| HLA-J | 1.225823164 | 3.312244655 | 12.30978201 | 2.75E-31 | 2.61E-30 | 59.71333501 |
| MICB | 1.184649394 | 2.84792631 | 12.30966951 | 2.75E-31 | 2.62E-30 | 59.71222319 |
| TRAM1L1 | -1.287229858 | 3.183003164 | -12.30950706 | 2.75E-31 | 2.62E-30 | 59.71061773 |
| SLC43A1 | -1.134776541 | 3.444181537 | -12.30737093 | 2.81E-31 | 2.67E-30 | 59.68950802 |
| UBASH3A | 1.209844115 | 3.160244873 | 12.29865928 | 3.07E-31 | 2.91E-30 | 59.60344035 |
| SIRPG | 1.975792385 | 2.837784406 | 12.29384954 | 3.22E-31 | 3.05E-30 | 59.55593788 |
| USP2 | -1.987112482 | 3.91176458 | -12.27910191 | 3.72E-31 | 3.53E-30 | 59.41035575 |
| HLA-DPA1 | 1.737160061 | 7.997721594 | 12.27808488 | 3.76E-31 | 3.56E-30 | 59.40032001 |
| TROAP | 1.519038571 | 2.138813061 | 12.26343078 | 4.35E-31 | 4.12E-30 | 59.25577339 |
| RPL28 | 1.084065914 | 8.595648215 | 12.26308254 | 4.37E-31 | 4.13E-30 | 59.2523397 |
| SLC9A9 | 1.758708641 | 3.86039095 | 12.26167959 | 4.43E-31 | 4.19E-30 | 59.23850683 |
| SLC25A4 | -1.159515145 | 6.085196416 | -12.24469218 | 5.24E-31 | 4.95E-30 | 59.07109039 |
| CA2 | -1.619686366 | 7.268960306 | -12.2440013 | 5.28E-31 | 4.98E-30 | 59.0642845 |
| SLC17A9 | 1.562869622 | 3.494851248 | 12.24191091 | 5.39E-31 | 5.08E-30 | 59.04369347 |
| BNIPL | -1.036678559 | 2.098626399 | -12.24042174 | 5.47E-31 | 5.15E-30 | 59.02902603 |
| P4HA2 | 1.547440316 | 4.596377817 | 12.23707323 | 5.65E-31 | 5.31E-30 | 58.99604895 |
| TNFAIP3 | 1.313808309 | 4.911381488 | 12.23603639 | 5.71E-31 | 5.36E-30 | 58.98583903 |
| PLXNA3 | 1.13502709 | 3.944721028 | 12.23516017 | 5.76E-31 | 5.41E-30 | 58.97721114 |
| CTHRC1 | 2.445985031 | 5.33340722 | 12.23297096 | 5.89E-31 | 5.52E-30 | 58.95565626 |
| TLR2 | 1.342715763 | 4.691309024 | 12.22988583 | 6.07E-31 | 5.69E-30 | 58.92528413 |
| COL5A2 | 1.738393882 | 6.489319564 | 12.22934952 | 6.10E-31 | 5.72E-30 | 58.92000475 |
| SVIP | -1.144770187 | 4.280907104 | -12.22931548 | 6.10E-31 | 5.72E-30 | 58.91966972 |
| PTGFR | -1.502170526 | 2.790029714 | -12.2220447 | 6.56E-31 | 6.14E-30 | 58.84811171 |
| ADAMTS10 | 1.568003469 | 3.337044202 | 12.21879638 | 6.77E-31 | 6.33E-30 | 58.81615049 |
| SCOC | -1.03494201 | 4.630843531 | -12.21829571 | 6.81E-31 | 6.36E-30 | 58.81122478 |
| MAPK8IP3 | 1.640547339 | 4.247800072 | 12.21792722 | 6.83E-31 | 6.38E-30 | 58.80759946 |
| ITK | 1.192535236 | 1.912922074 | 12.21677221 | 6.91E-31 | 6.45E-30 | 58.79623679 |
| MAP4K4 | 1.189402458 | 4.809843071 | 12.2146148 | 7.06E-31 | 6.59E-30 | 58.77501437 |
| MYC | 1.619262716 | 6.393826881 | 12.21208314 | 7.24E-31 | 6.75E-30 | 58.75011347 |
| NMB | 1.873779083 | 5.491950155 | 12.21010588 | 7.38E-31 | 6.88E-30 | 58.73066764 |
| IDH2 | -1.197512497 | 7.891146783 | -12.20718175 | 7.60E-31 | 7.08E-30 | 58.70191318 |
| ZNF692 | 1.376813861 | 5.01395289 | 12.20019591 | 8.14E-31 | 7.57E-30 | 58.63323498 |
| NNT | -1.327084612 | 5.584598701 | -12.1982515 | 8.30E-31 | 7.71E-30 | 58.61412355 |
| ADAM8 | 1.355663955 | 3.09761885 | 12.1971868 | 8.39E-31 | 7.79E-30 | 58.60365956 |
| MTFP1 | 1.167853094 | 2.435812195 | 12.19582502 | 8.50E-31 | 7.89E-30 | 58.5902766 |
| FRK | -1.175131588 | 4.702806328 | -12.18637495 | 9.33E-31 | 8.65E-30 | 58.49743083 |
| RIMKLA | 1.981494762 | 3.989793892 | 12.18294619 | 9.66E-31 | 8.94E-30 | 58.46375454 |
| COL8A1 | 1.865522477 | 5.519971273 | 12.17905905 | 1.00E-30 | 9.27E-30 | 58.42558313 |
| CD14 | 1.684882977 | 6.883092293 | 12.17353623 | 1.06E-30 | 9.78E-30 | 58.3713624 |
| RAB24 | 1.100243677 | 2.603360753 | 12.17323624 | 1.06E-30 | 9.80E-30 | 58.36841765 |
| NFKBIE | 1.393032568 | 5.644329597 | 12.17100235 | 1.09E-30 | 1.00E-29 | 58.34649089 |
| FDFT1 | -1.102002288 | 5.129105605 | -12.16796072 | 1.12E-30 | 1.03E-29 | 58.31663962 |
| PTGR1 | -1.277927054 | 5.805701075 | -12.16659259 | 1.13E-30 | 1.04E-29 | 58.30321398 |
| LMNB1 | 1.546579857 | 4.328051664 | 12.16649523 | 1.14E-30 | 1.04E-29 | 58.30225861 |
| PBK | 1.208717253 | 3.189887154 | 12.16575632 | 1.14E-30 | 1.05E-29 | 58.29500794 |
| SCARF1 | 1.303441216 | 5.493179375 | 12.1650132 | 1.15E-30 | 1.06E-29 | 58.28771632 |
| ANXA1 | 1.643332053 | 6.311874029 | 12.16411088 | 1.16E-30 | 1.07E-29 | 58.27886292 |
| IRF6 | -2.041080175 | 4.123557115 | -12.15938844 | 1.22E-30 | 1.12E-29 | 58.23253392 |
| HDAC11 | -1.018676964 | 4.405004157 | -12.15457806 | 1.28E-30 | 1.17E-29 | 58.18535344 |
| AVPR1B | 1.840034144 | 3.013886474 | 12.14556877 | 1.40E-30 | 1.28E-29 | 58.0970205 |
| ASS1 | -1.855549453 | 7.220091898 | -12.14401643 | 1.42E-30 | 1.30E-29 | 58.08180444 |
| DNAJC11 | -1.152422482 | 4.32097583 | -12.14301197 | 1.43E-30 | 1.31E-29 | 58.07195941 |
| SFTA1P | 1.005591928 | 1.83505343 | 12.1417803 | 1.45E-30 | 1.33E-29 | 58.05988795 |
| GPR4 | 1.650783188 | 5.979149426 | 12.1327242 | 1.59E-30 | 1.45E-29 | 57.97115362 |
| TSPAN4 | 1.162692654 | 6.934546205 | 12.11969822 | 1.80E-30 | 1.64E-29 | 57.84359249 |
| GDF7 | -1.21162987 | 1.831735161 | -12.11821994 | 1.83E-30 | 1.67E-29 | 57.82912131 |
| ARHGEF1 | 1.295830863 | 4.600953007 | 12.10976195 | 1.99E-30 | 1.81E-29 | 57.74634496 |
| WT1 | -1.710722537 | 2.546273988 | -12.10835115 | 2.02E-30 | 1.84E-29 | 57.73254129 |
| AUH | -1.124886566 | 6.08635782 | -12.1069316 | 2.04E-30 | 1.86E-29 | 57.71865296 |
| CEACAM21 | 1.061834903 | 2.059123533 | 12.1061232 | 2.06E-30 | 1.87E-29 | 57.71074431 |
| RPS8 | 1.016947874 | 9.186386633 | 12.1008692 | 2.17E-30 | 1.97E-29 | 57.6593521 |
| ARHGEF6 | 1.116162815 | 5.489653997 | 12.09402451 | 2.32E-30 | 2.10E-29 | 57.59242106 |
| CYS1 | -2.34043914 | 6.7580942 | -12.09023397 | 2.41E-30 | 2.18E-29 | 57.55536515 |
| SEMA6B | 1.434425502 | 4.555042737 | 12.08538288 | 2.53E-30 | 2.29E-29 | 57.50795181 |
| PRSS8 | -1.908803413 | 5.209486382 | -12.08517364 | 2.53E-30 | 2.29E-29 | 57.50590701 |
| HHLA2 | 2.789938766 | 5.232836792 | 12.08362834 | 2.57E-30 | 2.33E-29 | 57.49080627 |
| THBS3 | 1.083779271 | 4.559386095 | 12.08094107 | 2.64E-30 | 2.39E-29 | 57.46454899 |
| HLA-DRB1 | 1.60455409 | 10.99820996 | 12.07668812 | 2.75E-30 | 2.48E-29 | 57.42300092 |
| P2RY8 | 1.544036794 | 4.975269291 | 12.07222884 | 2.87E-30 | 2.59E-29 | 57.37944674 |
| DOCK10 | 1.179564343 | 4.49482078 | 12.06820334 | 2.99E-30 | 2.69E-29 | 57.34013784 |
| CSF1R | 1.602987064 | 6.384607407 | 12.06578114 | 3.06E-30 | 2.75E-29 | 57.31648899 |
| QRFPR | 2.029838531 | 4.069890969 | 12.06511405 | 3.08E-30 | 2.77E-29 | 57.30997646 |
| HCG27 | 1.366656919 | 1.9293111 | 12.06272468 | 3.16E-30 | 2.84E-29 | 57.28665188 |
| MYO5C | -1.302870898 | 2.876351223 | -12.06026989 | 3.23E-30 | 2.90E-29 | 57.26269164 |
| ADAMTSL4 | 1.620452095 | 3.957327567 | 12.05758158 | 3.32E-30 | 2.98E-29 | 57.23645555 |
| LY96 | 1.531461231 | 4.832855146 | 12.05269934 | 3.48E-30 | 3.12E-29 | 57.18881737 |
| B3GALT2 | -1.027770865 | 1.371181163 | -12.04909366 | 3.61E-30 | 3.23E-29 | 57.15364278 |
| HLA-DRA | 1.562447858 | 11.17284842 | 12.04862869 | 3.62E-30 | 3.25E-29 | 57.14910742 |
| MYL5 | 1.321178307 | 3.718423195 | 12.04561554 | 3.73E-30 | 3.34E-29 | 57.11971888 |
| RAB33A | 1.362691207 | 2.438386265 | 12.03589593 | 4.11E-30 | 3.67E-29 | 57.02495036 |
| CAMK1D | 1.134602009 | 4.677288019 | 12.0349203 | 4.15E-30 | 3.70E-29 | 57.01544031 |
| SYPL2 | -1.649314545 | 3.084939702 | -12.02971032 | 4.36E-30 | 3.89E-29 | 56.96466381 |
| JMY | -1.074219445 | 4.291563737 | -12.02367134 | 4.63E-30 | 4.13E-29 | 56.90582481 |
| ANKZF1 | 1.206523098 | 5.098020013 | 12.02256675 | 4.68E-30 | 4.17E-29 | 56.89506459 |
| GRAMD4 | 1.543607211 | 5.791508668 | 12.01793937 | 4.90E-30 | 4.36E-29 | 56.84999408 |
| GLTP | -1.175901061 | 6.401153892 | -12.01521072 | 5.03E-30 | 4.47E-29 | 56.8234222 |
| FOXI2 | -1.648127694 | 2.616187794 | -12.01161374 | 5.21E-30 | 4.63E-29 | 56.78840007 |
| ARHGEF38 | -1.090400902 | 1.494264074 | -11.9991056 | 5.89E-30 | 5.22E-29 | 56.66666494 |
| ADAMTS2 | 1.574574915 | 4.006352316 | 11.99361968 | 6.22E-30 | 5.50E-29 | 56.61329813 |
| FAM124A | -1.059374412 | 3.056679602 | -11.99192532 | 6.32E-30 | 5.59E-29 | 56.5968185 |
| ENTPD8 | -1.088257404 | 2.373271416 | -11.99021589 | 6.43E-30 | 5.68E-29 | 56.58019387 |
| PNPLA1 | -1.072838996 | 1.154525877 | -11.98825173 | 6.55E-30 | 5.78E-29 | 56.56109366 |
| DYNLL2 | -1.213198409 | 5.527459646 | -11.98614803 | 6.69E-30 | 5.90E-29 | 56.54063862 |
| CDS1 | -1.451011092 | 4.436180206 | -11.98358008 | 6.86E-30 | 6.05E-29 | 56.5156726 |
| SEMA3B | -1.595432696 | 3.431789055 | -11.97567061 | 7.41E-30 | 6.52E-29 | 56.4387964 |
| PLK1 | 1.092995168 | 2.838681726 | 11.97342971 | 7.57E-30 | 6.65E-29 | 56.41702167 |
| PDE7A | 1.154222826 | 4.012178178 | 11.97125335 | 7.74E-30 | 6.79E-29 | 56.39587645 |
| MRPS6 | -1.242607531 | 5.806022074 | -11.96660621 | 8.10E-30 | 7.09E-29 | 56.35073355 |
| VAV3 | -1.467806351 | 5.444788112 | -11.96569369 | 8.17E-30 | 7.15E-29 | 56.34187055 |
| RHOBTB3 | -1.710042859 | 3.586724518 | -11.95423347 | 9.14E-30 | 7.97E-29 | 56.23059646 |
| UNC5B | 1.606875535 | 5.891805143 | 11.95095039 | 9.43E-30 | 8.23E-29 | 56.19873135 |
| NINJ2 | 1.371714046 | 2.566629605 | 11.94893988 | 9.62E-30 | 8.38E-29 | 56.17922026 |
| STK32A | -1.326008035 | 1.718000847 | -11.94651443 | 9.85E-30 | 8.58E-29 | 56.15568515 |
| RNASE2 | 1.499702542 | 3.006433674 | 11.94439748 | 1.01E-29 | 8.75E-29 | 56.13514592 |
| WLS | -1.492778115 | 6.285390962 | -11.9437719 | 1.01E-29 | 8.80E-29 | 56.12907686 |
| HOXA11 | -1.144225213 | 1.773534137 | -11.94128365 | 1.04E-29 | 9.00E-29 | 56.10493892 |
| PCDHB10 | 1.191295006 | 3.366270503 | 11.92316519 | 1.24E-29 | 1.07E-28 | 55.92927047 |
| PM20D1 | -1.206169968 | 2.388356435 | -11.92241374 | 1.25E-29 | 1.08E-28 | 55.92198836 |
| RTP4 | 1.501272 | 4.464398186 | 11.91689646 | 1.32E-29 | 1.14E-28 | 55.8685304 |
| PATL2 | 1.124874389 | 2.670213481 | 11.91472175 | 1.34E-29 | 1.16E-28 | 55.84746351 |
| PRKCQ | -1.434214332 | 3.09359253 | -11.9143533 | 1.35E-29 | 1.17E-28 | 55.84389452 |
| NHSL1 | -1.164600679 | 3.772688412 | -11.91294861 | 1.37E-29 | 1.18E-28 | 55.83028854 |
| TBX15 | 1.510649058 | 2.851885171 | 11.904301 | 1.49E-29 | 1.29E-28 | 55.74654873 |
| PRPS2 | -1.017614857 | 5.737316 | -11.90357234 | 1.50E-29 | 1.29E-28 | 55.73949446 |
| LGI4 | 2.748256653 | 4.499428881 | 11.88954331 | 1.72E-29 | 1.48E-28 | 55.60372955 |
| DUSP10 | 1.110841898 | 4.096375627 | 11.88576126 | 1.78E-29 | 1.54E-28 | 55.5671461 |
| RGS14 | 1.208792899 | 4.592387219 | 11.88241831 | 1.84E-29 | 1.59E-28 | 55.53481619 |
| KIF18B | 1.228777647 | 1.518572822 | 11.88011565 | 1.88E-29 | 1.62E-28 | 55.51255032 |
| NLGN1 | 1.504223736 | 4.202003029 | 11.87931569 | 1.90E-29 | 1.63E-28 | 55.50481563 |
| MYO6 | -1.188795306 | 5.568977115 | -11.87285353 | 2.02E-29 | 1.74E-28 | 55.44234588 |
| FAM163A | 1.044410579 | 2.161451618 | 11.8639599 | 2.20E-29 | 1.89E-28 | 55.35640611 |
| PLEKHO2 | 2.069345915 | 5.618607914 | 11.86023715 | 2.28E-29 | 1.96E-28 | 55.32044482 |
| MUC1 | -2.05522664 | 6.582963688 | -11.85174829 | 2.48E-29 | 2.13E-28 | 55.23847022 |
| FAS | 1.395192144 | 5.199275464 | 11.85119103 | 2.49E-29 | 2.14E-28 | 55.23309014 |
| SEMA4A | -1.244384116 | 3.840955096 | -11.85013118 | 2.52E-29 | 2.16E-28 | 55.22285834 |
| FBLN5 | -2.032622508 | 5.68117265 | -11.84676739 | 2.60E-29 | 2.23E-28 | 55.19038813 |
| C11orf21 | 1.047883302 | 1.467779322 | 11.84577498 | 2.63E-29 | 2.25E-28 | 55.18080962 |
| LINGO1 | 1.44796152 | 3.97342105 | 11.84169314 | 2.73E-29 | 2.34E-28 | 55.141418 |
| L2HGDH | -1.160698136 | 3.998234753 | -11.8399271 | 2.78E-29 | 2.38E-28 | 55.12437748 |
| SIRPB1 | 1.231668807 | 2.965581152 | 11.83650303 | 2.88E-29 | 2.45E-28 | 55.09134336 |
| KITLG | -1.571759122 | 5.505887036 | -11.8359863 | 2.89E-29 | 2.47E-28 | 55.08635863 |
| HS3ST2 | 1.949773942 | 3.462620183 | 11.83356297 | 2.96E-29 | 2.52E-28 | 55.06298354 |
| BTBD11 | -1.42303171 | 2.159627103 | -11.83320961 | 2.97E-29 | 2.53E-28 | 55.05957536 |
| TFAP2C | -1.363238363 | 2.151794551 | -11.8279684 | 3.12E-29 | 2.66E-28 | 55.00903054 |
| TNFSF14 | 1.527289861 | 1.877405895 | 11.82521389 | 3.21E-29 | 2.73E-28 | 54.98247245 |
| CLMN | -1.549038961 | 3.848916151 | -11.82477663 | 3.22E-29 | 2.74E-28 | 54.97825689 |
| BPHL | -1.104900107 | 3.777098109 | -11.82340112 | 3.27E-29 | 2.78E-28 | 54.96499647 |
| ACADM | -1.405361414 | 5.689018454 | -11.82155992 | 3.32E-29 | 2.82E-28 | 54.94724812 |
| PROC | -1.7746026 | 3.213279548 | -11.82028419 | 3.37E-29 | 2.86E-28 | 54.93495161 |
| NDRG2 | -1.343261531 | 6.24301809 | -11.81898582 | 3.41E-29 | 2.89E-28 | 54.92243779 |
| ALDH4A1 | -1.86133087 | 6.26893656 | -11.81255283 | 3.63E-29 | 3.07E-28 | 54.86044874 |
| IFI44 | 1.387305323 | 5.453192472 | 11.8092783 | 3.75E-29 | 3.17E-28 | 54.82890317 |
| DNAJC6 | -1.362320662 | 3.379233979 | -11.80899304 | 3.76E-29 | 3.18E-28 | 54.82615538 |
| FAM102A | -1.224415649 | 5.890644547 | -11.80572778 | 3.88E-29 | 3.28E-28 | 54.79470506 |
| HSD17B7 | 1.182634919 | 4.356078733 | 11.80511355 | 3.90E-29 | 3.29E-28 | 54.78878945 |
| SYT10 | -1.202896354 | 0.937549481 | -11.79917954 | 4.13E-29 | 3.49E-28 | 54.73165027 |
| IL1RL1 | -2.03406336 | 2.973307856 | -11.79270176 | 4.40E-29 | 3.71E-28 | 54.66929562 |
| GPR183 | 1.539235991 | 4.177753513 | 11.78660516 | 4.67E-29 | 3.93E-28 | 54.61063006 |
| ITPR2 | -1.357698323 | 3.960915239 | -11.78286182 | 4.84E-29 | 4.07E-28 | 54.57461853 |
| CLSPN | 1.211995159 | 1.755898572 | 11.78008362 | 4.97E-29 | 4.17E-28 | 54.54789659 |
| CXorf21 | 1.323389807 | 2.843013206 | 11.77958928 | 4.99E-29 | 4.18E-28 | 54.54314214 |
| CBLN3 | 1.180280358 | 3.660093761 | 11.77772548 | 5.08E-29 | 4.26E-28 | 54.52521798 |
| GZMK | 2.017809027 | 3.647356373 | 11.7772774 | 5.11E-29 | 4.28E-28 | 54.52090901 |
| AGR3 | -1.22541186 | 0.856391073 | -11.77403881 | 5.27E-29 | 4.41E-28 | 54.4897685 |
| PTPN3 | -1.339067019 | 4.408091149 | -11.76486743 | 5.76E-29 | 4.82E-28 | 54.40161078 |
| HIBADH | -1.065932043 | 7.08641525 | -11.75780087 | 6.17E-29 | 5.15E-28 | 54.33371487 |
| NAPEPLD | -1.03181276 | 4.102075236 | -11.75735229 | 6.19E-29 | 5.17E-28 | 54.32940575 |
| POPDC2 | 1.174400102 | 4.174616006 | 11.75475977 | 6.35E-29 | 5.30E-28 | 54.30450374 |
| NCAPH | 1.022517393 | 1.579252858 | 11.75368338 | 6.42E-29 | 5.35E-28 | 54.29416565 |
| ABCA8 | -1.516638493 | 3.228594563 | -11.7470564 | 6.84E-29 | 5.70E-28 | 54.23053076 |
| C5orf46 | 3.273468484 | 3.503227222 | 11.74676988 | 6.86E-29 | 5.72E-28 | 54.22777999 |
| SOD3 | -1.59801636 | 5.204850996 | -11.74392257 | 7.05E-29 | 5.87E-28 | 54.20044636 |
| EXOC3L4 | 1.685044403 | 3.119824628 | 11.7433228 | 7.09E-29 | 5.90E-28 | 54.19468922 |
| TUBB6 | 1.222324316 | 5.04387245 | 11.73825839 | 7.45E-29 | 6.19E-28 | 54.14608394 |
| TRGV7 | 1.251862582 | 2.025774599 | 11.73523705 | 7.67E-29 | 6.37E-28 | 54.11709308 |
| PCSK1N | -2.637563135 | 4.089939982 | -11.73328864 | 7.81E-29 | 6.49E-28 | 54.09839996 |
| GALM | -1.041006679 | 5.817934635 | -11.72208862 | 8.71E-29 | 7.22E-28 | 53.99098458 |
| RPL35 | 1.007667296 | 9.098974273 | 11.7107139 | 9.72E-29 | 8.05E-28 | 53.88196039 |
| BCR | -1.084735346 | 4.23263352 | -11.70718265 | 1.01E-28 | 8.31E-28 | 53.84812779 |
| BMP6 | -1.530051649 | 4.146330221 | -11.69940744 | 1.08E-28 | 8.95E-28 | 53.77365706 |
| SUCNR1 | -1.912805583 | 4.5158305 | -11.69847287 | 1.09E-28 | 9.02E-28 | 53.76470783 |
| NANOS1 | -1.053784968 | 1.561056753 | -11.68726662 | 1.22E-28 | 1.00E-27 | 53.65743557 |
| DEGS1 | 1.159873659 | 6.575517897 | 11.6846243 | 1.25E-28 | 1.03E-27 | 53.63215139 |
| TMIGD2 | 1.084200986 | 1.739793576 | 11.68422684 | 1.25E-28 | 1.03E-27 | 53.62834844 |
| NUP62 | 1.039053355 | 4.812945968 | 11.6759919 | 1.36E-28 | 1.12E-27 | 53.54957387 |
| ABCB1 | -1.953978442 | 5.38425904 | -11.6751468 | 1.37E-28 | 1.13E-27 | 53.54149168 |
| ZNF83 | 1.381432294 | 4.686020577 | 11.67368993 | 1.39E-28 | 1.14E-27 | 53.5275598 |
| ANLN | 1.413787258 | 2.339036225 | 11.6692062 | 1.45E-28 | 1.19E-27 | 53.4846892 |
| SLC34A3 | -1.711456184 | 2.37948041 | -11.66907963 | 1.45E-28 | 1.19E-27 | 53.48347914 |
| CDHR3 | 1.149627686 | 2.857799287 | 11.66853506 | 1.46E-28 | 1.20E-27 | 53.47827309 |
| MFNG | 1.059103761 | 6.245529338 | 11.66670351 | 1.48E-28 | 1.22E-27 | 53.46076463 |
| BCAS1 | -1.328487402 | 1.532802304 | -11.66489651 | 1.51E-28 | 1.24E-27 | 53.4434925 |
| TRIM14 | 1.299053508 | 6.288057998 | 11.65960471 | 1.59E-28 | 1.30E-27 | 53.39292102 |
| HCST | 1.231514545 | 3.281824949 | 11.65518447 | 1.66E-28 | 1.36E-27 | 53.35068999 |
| CP | 3.267133482 | 5.296934294 | 11.65503487 | 1.66E-28 | 1.36E-27 | 53.34926087 |
| PCK2 | -1.350785873 | 5.182730921 | -11.64851212 | 1.77E-28 | 1.45E-27 | 53.28696147 |
| MMP14 | 1.851731515 | 7.022403351 | 11.63853274 | 1.95E-28 | 1.59E-27 | 53.19169063 |
| FREM2 | -1.608292408 | 3.959210529 | -11.63458724 | 2.02E-28 | 1.65E-27 | 53.15403824 |
| CHODL | -1.350114751 | 2.757867722 | -11.62681345 | 2.18E-28 | 1.77E-27 | 53.07987591 |
| MCTP2 | 1.066652018 | 3.271003308 | 11.62306679 | 2.26E-28 | 1.84E-27 | 53.04414385 |
| BTN2A2 | 1.045712369 | 3.598597999 | 11.62265586 | 2.27E-28 | 1.85E-27 | 53.04022522 |
| PXDN | 1.547625039 | 5.534134616 | 11.62196003 | 2.28E-28 | 1.86E-27 | 53.03359006 |
| CAT | -1.216732646 | 8.577155393 | -11.6208105 | 2.31E-28 | 1.88E-27 | 53.02262908 |
| MYO15B | 1.585543458 | 6.034848853 | 11.61522687 | 2.44E-28 | 1.98E-27 | 52.96939816 |
| FHOD3 | -1.241174302 | 4.351624308 | -11.60182322 | 2.77E-28 | 2.25E-27 | 52.8416827 |
| FAM107A | -1.642492638 | 4.555310681 | -11.60082418 | 2.80E-28 | 2.27E-27 | 52.83216721 |
| RBM47 | -1.137177055 | 5.199744265 | -11.59327511 | 3.01E-28 | 2.43E-27 | 52.76028219 |
| TWSG1 | -1.158951181 | 5.039922587 | -11.59222226 | 3.04E-28 | 2.46E-27 | 52.75025897 |
| MTTP | -1.647192858 | 2.48427489 | -11.58869292 | 3.14E-28 | 2.54E-27 | 52.71666349 |
| PELI2 | -1.17988728 | 3.215667298 | -11.58854528 | 3.15E-28 | 2.54E-27 | 52.71525828 |
| SLC19A2 | -1.473239757 | 3.741338308 | -11.58279599 | 3.32E-28 | 2.68E-27 | 52.66054588 |
| ISG15 | 1.446855869 | 7.378137278 | 11.57458812 | 3.60E-28 | 2.90E-27 | 52.58246694 |
| TOX2 | 1.286827096 | 2.950192475 | 11.57350475 | 3.63E-28 | 2.93E-27 | 52.57216375 |
| CORO1C | 1.061927899 | 6.602580829 | 11.56953178 | 3.77E-28 | 3.04E-27 | 52.53438515 |
| UST | -1.412143359 | 3.6102198 | -11.56488526 | 3.94E-28 | 3.18E-27 | 52.49021238 |
| PI16 | -1.501559724 | 2.192958593 | -11.5623043 | 4.04E-28 | 3.26E-27 | 52.46568102 |
| TIGIT | 1.343900878 | 2.404038154 | 11.56058849 | 4.11E-28 | 3.31E-27 | 52.44937466 |
| HEY1 | 1.262991154 | 4.185790631 | 11.56052816 | 4.11E-28 | 3.31E-27 | 52.44880139 |
| GLIPR1 | 1.274112094 | 4.107374206 | 11.55610136 | 4.29E-28 | 3.45E-27 | 52.40673824 |
| FMNL3 | 1.118918882 | 4.891455492 | 11.55093542 | 4.51E-28 | 3.62E-27 | 52.35766488 |
| PCDH17 | 1.64236308 | 4.575215021 | 11.54732399 | 4.67E-28 | 3.74E-27 | 52.32336685 |
| ANGPTL3 | -2.343991335 | 2.73031172 | -11.54262382 | 4.88E-28 | 3.91E-27 | 52.27873924 |
| PRRG1 | -1.17674614 | 4.046314707 | -11.5418739 | 4.92E-28 | 3.94E-27 | 52.27161994 |
| ACSM3 | -1.29314827 | 3.947197037 | -11.54182036 | 4.92E-28 | 3.94E-27 | 52.27111167 |
| CENPF | 1.121666818 | 3.20102411 | 11.52550003 | 5.75E-28 | 4.60E-27 | 52.11624989 |
| ABHD11-AS1 | 1.504945942 | 2.552247375 | 11.52079032 | 6.01E-28 | 4.80E-27 | 52.07158627 |
| APOL2 | 1.316260688 | 5.969477934 | 11.52070518 | 6.02E-28 | 4.81E-27 | 52.070779 |
| NFKB2 | 1.117032664 | 6.269121072 | 11.51880785 | 6.13E-28 | 4.89E-27 | 52.05278945 |
| ST3GAL4 | -1.203721738 | 4.599639606 | -11.51709832 | 6.23E-28 | 4.97E-27 | 52.03658212 |
| QTRT1 | 1.083361818 | 4.914784741 | 11.51040084 | 6.64E-28 | 5.29E-27 | 51.97310123 |
| NCR1 | 1.006985743 | 1.795407893 | 11.509948 | 6.67E-28 | 5.31E-27 | 51.96881001 |
| TPSAB1 | 1.757815627 | 4.285028253 | 11.50313984 | 7.12E-28 | 5.67E-27 | 51.90430612 |
| COL1A1 | 2.454197902 | 6.973770978 | 11.50269229 | 7.15E-28 | 5.69E-27 | 51.90006667 |
| CYP1A1 | -1.038186119 | 0.738060114 | -11.50129225 | 7.24E-28 | 5.76E-27 | 51.88680535 |
| TBC1D9 | -1.126740447 | 5.303923388 | -11.50009979 | 7.33E-28 | 5.82E-27 | 51.87551112 |
| CNTNAP1 | 1.103765825 | 2.456927078 | 11.49565561 | 7.64E-28 | 6.07E-27 | 51.83342525 |
| TM7SF2 | -1.17322025 | 4.781712903 | -11.49211675 | 7.91E-28 | 6.28E-27 | 51.79992019 |
| ITM2C | -1.395475313 | 7.332345626 | -11.48767443 | 8.25E-28 | 6.55E-27 | 51.75787088 |
| SIRPA | 1.389937175 | 6.938925315 | 11.48656611 | 8.33E-28 | 6.61E-27 | 51.74738151 |
| EPS8L1 | -1.669250439 | 3.215570629 | -11.47962438 | 8.90E-28 | 7.05E-27 | 51.68169878 |
| CDKL2 | -1.12453149 | 2.58464886 | -11.47473521 | 9.33E-28 | 7.38E-27 | 51.63545289 |
| SBNO2 | 1.065854882 | 5.336882676 | 11.47467906 | 9.33E-28 | 7.38E-27 | 51.63492186 |
| RGS18 | 1.260360409 | 2.616231944 | 11.4736012 | 9.43E-28 | 7.45E-27 | 51.62472828 |
| GFI1 | 1.097419015 | 1.642235686 | 11.47086541 | 9.68E-28 | 7.65E-27 | 51.59885802 |
| DAO | -2.006636103 | 3.069643567 | -11.46956161 | 9.80E-28 | 7.74E-27 | 51.58653041 |
| HAVCR2 | 2.053629675 | 6.398549087 | 11.46859132 | 9.89E-28 | 7.80E-27 | 51.57735681 |
| TRIM22 | 1.301576762 | 5.640183318 | 11.45228426 | 1.16E-27 | 9.09E-27 | 51.42325639 |
| PRSS3 | -1.06664556 | 1.845868534 | -11.45185674 | 1.16E-27 | 9.12E-27 | 51.41921824 |
| ELOVL2 | 1.397582894 | 3.08995103 | 11.4364117 | 1.34E-27 | 1.06E-26 | 51.27339857 |
| KCNMA1 | 1.391601292 | 2.969854545 | 11.42924558 | 1.44E-27 | 1.13E-26 | 51.20578519 |
| MAP3K12 | 1.027809397 | 2.898420725 | 11.42780688 | 1.46E-27 | 1.14E-26 | 51.19221422 |
| B3GNT8 | -1.023076577 | 3.2561982 | -11.42772711 | 1.46E-27 | 1.14E-26 | 51.19146181 |
| CLNK | -1.576711744 | 1.122240363 | -11.42042819 | 1.56E-27 | 1.22E-26 | 51.12262966 |
| AFAP1L1 | 1.351933898 | 4.601349037 | 11.4193735 | 1.58E-27 | 1.24E-26 | 51.11268586 |
| CDKN1C | -1.504040752 | 4.487188738 | -11.41817568 | 1.60E-27 | 1.25E-26 | 51.10139323 |
| P2RY13 | 1.471499744 | 3.268669376 | 11.40518296 | 1.81E-27 | 1.41E-26 | 50.9789525 |
| ACAN | 1.821312001 | 3.673806438 | 11.40258262 | 1.85E-27 | 1.44E-26 | 50.95445836 |
| TNIP1 | 1.099156255 | 7.853882745 | 11.40180799 | 1.87E-27 | 1.45E-26 | 50.94716234 |
| SEL1L3 | 1.497080997 | 6.628634923 | 11.39903611 | 1.92E-27 | 1.49E-26 | 50.92105751 |
| DDX56 | 1.039947896 | 5.507684071 | 11.39636697 | 1.96E-27 | 1.53E-26 | 50.89592411 |
| MS4A4E | 1.100949961 | 1.980998946 | 11.39456317 | 2.00E-27 | 1.55E-26 | 50.87894113 |
| FCGR2B | 1.258808254 | 3.019595313 | 11.39237368 | 2.04E-27 | 1.58E-26 | 50.85832932 |
| MT1F | -2.027107 | 4.796575547 | -11.38842593 | 2.12E-27 | 1.64E-26 | 50.82117171 |
| PRKCA | -1.183770367 | 3.410794816 | -11.38757594 | 2.14E-27 | 1.66E-26 | 50.81317241 |
| SACS | 1.182588398 | 3.370763871 | 11.3640155 | 2.67E-27 | 2.07E-26 | 50.59159867 |
| CYP26B1 | -1.367169202 | 2.530382824 | -11.347407 | 3.12E-27 | 2.41E-26 | 50.4355844 |
| CREB5 | 1.248558454 | 4.552635921 | 11.34413238 | 3.22E-27 | 2.49E-26 | 50.4048414 |
| ARHGAP33 | 1.303316406 | 4.273480031 | 11.34322398 | 3.25E-27 | 2.51E-26 | 50.39631408 |
| NTN4 | -1.722936819 | 6.516771307 | -11.33171391 | 3.62E-27 | 2.79E-26 | 50.28830617 |
| NCS1 | -1.214796314 | 3.591660392 | -11.3308464 | 3.65E-27 | 2.81E-26 | 50.28016857 |
| SLC43A2 | -1.014751811 | 5.499619703 | -11.32759693 | 3.77E-27 | 2.89E-26 | 50.24969086 |
| SYNM | 1.087402754 | 4.554330814 | 11.32235377 | 3.96E-27 | 3.04E-26 | 50.2005257 |
| NLRP2 | -1.136861224 | 2.213651781 | -11.31829845 | 4.11E-27 | 3.15E-26 | 50.16250915 |
| PPP1R3B | 1.247758864 | 6.116545665 | 11.31472475 | 4.25E-27 | 3.26E-26 | 50.12901496 |
| ASPM | 1.110432661 | 2.517856129 | 11.30438504 | 4.69E-27 | 3.58E-26 | 50.03214606 |
| CYBA | 1.175139648 | 7.253303204 | 11.30404483 | 4.70E-27 | 3.59E-26 | 50.02895975 |
| CENPA | 1.079551437 | 1.903555094 | 11.3034253 | 4.73E-27 | 3.61E-26 | 50.02315751 |
| CCL18 | 2.807580769 | 3.389133175 | 11.30270507 | 4.76E-27 | 3.63E-26 | 50.01641254 |
| GIMAP4 | 1.241068172 | 6.257375676 | 11.29930137 | 4.92E-27 | 3.75E-26 | 49.98454026 |
| AKIRIN1 | -1.043097235 | 6.243164687 | -11.29622482 | 5.06E-27 | 3.86E-26 | 49.95573688 |
| CDCA5 | 1.04908898 | 3.715302213 | 11.29525849 | 5.11E-27 | 3.89E-26 | 49.94669101 |
| CDH1 | -1.576704201 | 7.03920075 | -11.29304448 | 5.22E-27 | 3.97E-26 | 49.92596725 |
| MCCC1 | -1.041196085 | 4.870006124 | -11.28787365 | 5.48E-27 | 4.17E-26 | 49.87757735 |
| GCM1 | -1.02160838 | 0.897453398 | -11.28756263 | 5.50E-27 | 4.18E-26 | 49.87466716 |
| HMGCR | -1.092795129 | 4.028058967 | -11.28258646 | 5.76E-27 | 4.37E-26 | 49.82811352 |
| TOM1L2 | -1.008263931 | 5.029997507 | -11.28149655 | 5.82E-27 | 4.42E-26 | 49.81791885 |
| CHST13 | 1.808613971 | 4.0167087 | 11.27726465 | 6.06E-27 | 4.59E-26 | 49.77834117 |
| PLOD2 | 1.457585319 | 7.142761901 | 11.27428402 | 6.23E-27 | 4.71E-26 | 49.75047151 |
| ABI3 | 1.084898327 | 5.329807927 | 11.27052669 | 6.45E-27 | 4.88E-26 | 49.71534645 |
| MARCKS | 1.087881773 | 6.395405862 | 11.27050349 | 6.45E-27 | 4.88E-26 | 49.71512963 |
| RGS5 | 2.155172743 | 9.479346986 | 11.26970256 | 6.50E-27 | 4.91E-26 | 49.70764322 |
| OSMR | 1.441831289 | 6.123988126 | 11.26736985 | 6.65E-27 | 5.02E-26 | 49.68584091 |
| TMEM155 | 1.131096685 | 2.735043999 | 11.26445717 | 6.83E-27 | 5.15E-26 | 49.65862236 |
| STARD3 | 1.111798347 | 4.472755248 | 11.26413311 | 6.85E-27 | 5.17E-26 | 49.65559427 |
| SDR42E1 | -1.080962441 | 2.332664923 | -11.26260966 | 6.95E-27 | 5.24E-26 | 49.64135997 |
| NEDD4L | -1.106696061 | 4.226066334 | -11.2537103 | 7.56E-27 | 5.69E-26 | 49.55823425 |
| PLA2G4A | -1.395965138 | 4.337862434 | -11.25301228 | 7.61E-27 | 5.72E-26 | 49.55171612 |
| SIGLEC7 | 1.203818288 | 2.422509292 | 11.25100909 | 7.75E-27 | 5.83E-26 | 49.53301172 |
| CARD11 | 1.531169548 | 3.70524167 | 11.24978221 | 7.84E-27 | 5.89E-26 | 49.52155709 |
| KIT | -1.727757466 | 4.55628327 | -11.24574049 | 8.15E-27 | 6.11E-26 | 49.48382777 |
| CDH6 | 1.923145638 | 6.378560155 | 11.233887 | 9.11E-27 | 6.81E-26 | 49.37322741 |
| OASL | 1.29276936 | 3.610788269 | 11.22504051 | 9.90E-27 | 7.38E-26 | 49.29073439 |
| PLEKHG4 | 1.411251804 | 3.275265022 | 11.22163799 | 1.02E-26 | 7.61E-26 | 49.25901744 |
| HLA-L | 1.083704437 | 3.230759 | 11.22152409 | 1.02E-26 | 7.62E-26 | 49.25795579 |
| CD36 | 2.085816487 | 5.472251574 | 11.22025175 | 1.04E-26 | 7.71E-26 | 49.24609731 |
| ST14 | -1.707406221 | 5.480640313 | -11.21942294 | 1.04E-26 | 7.76E-26 | 49.23837307 |
| INF2 | 1.177367729 | 6.139780588 | 11.20350858 | 1.21E-26 | 9.00E-26 | 49.09012948 |
| C4B | 2.104230992 | 4.576450083 | 11.20230907 | 1.22E-26 | 9.10E-26 | 49.07896165 |
| TMEM38A | -1.671129471 | 4.238163299 | -11.19749605 | 1.28E-26 | 9.50E-26 | 49.03415854 |
| WDR72 | -1.907249591 | 3.999356135 | -11.19710835 | 1.29E-26 | 9.53E-26 | 49.0305501 |
| PLOD1 | 1.185062726 | 7.181982554 | 11.19392678 | 1.33E-26 | 9.81E-26 | 49.00094137 |
| HTRA4 | 1.186402329 | 2.271002332 | 11.19283803 | 1.34E-26 | 9.91E-26 | 48.99081041 |
| NAP1L3 | -1.333913042 | 2.548445694 | -11.19015362 | 1.37E-26 | 1.02E-25 | 48.9658343 |
| IDI1 | -1.035857528 | 4.745017011 | -11.18398819 | 1.45E-26 | 1.07E-25 | 48.90848554 |
| YEATS2 | 1.131285158 | 4.105468489 | 11.18300401 | 1.47E-26 | 1.08E-25 | 48.89933291 |
| LYNX1 | -1.159317787 | 3.416915015 | -11.18299673 | 1.47E-26 | 1.08E-25 | 48.89926524 |
| WDR90 | 1.023007986 | 4.219121267 | 11.18292567 | 1.47E-26 | 1.08E-25 | 48.89860444 |
| PARP15 | 1.01292083 | 1.998170454 | 11.18250746 | 1.47E-26 | 1.09E-25 | 48.89471539 |
| NFKBID | 1.484774598 | 3.712689197 | 11.1806207 | 1.50E-26 | 1.11E-25 | 48.87717106 |
| COL4A2 | 1.576755664 | 6.929153075 | 11.17188962 | 1.63E-26 | 1.20E-25 | 48.79600942 |
| APCDD1L | -1.692349356 | 2.218857477 | -11.16924611 | 1.67E-26 | 1.23E-25 | 48.77144437 |
| LOXL3 | 1.017543063 | 3.174890703 | 11.16598315 | 1.72E-26 | 1.26E-25 | 48.74112829 |
| KCNJ2 | 1.407134902 | 3.881922581 | 11.16135872 | 1.80E-26 | 1.32E-25 | 48.69817297 |
| TREH | -1.616619463 | 1.810571947 | -11.13957966 | 2.20E-26 | 1.61E-25 | 48.49603082 |
| SYTL4 | -1.05184717 | 3.200887226 | -11.13874307 | 2.22E-26 | 1.62E-25 | 48.48827121 |
| MAP3K14 | 1.197072569 | 4.802265776 | 11.12537954 | 2.52E-26 | 1.84E-25 | 48.3643737 |
| PFN2 | -1.390025517 | 6.655681304 | -11.12245693 | 2.59E-26 | 1.89E-25 | 48.33729045 |
| AGBL3 | 1.085127686 | 3.172704651 | 11.12198753 | 2.60E-26 | 1.89E-25 | 48.33294099 |
| PMEPA1 | 1.512067122 | 6.840354969 | 11.12158552 | 2.61E-26 | 1.90E-25 | 48.32921615 |
| TLR7 | 1.506438954 | 4.343246446 | 11.12034537 | 2.64E-26 | 1.92E-25 | 48.31772592 |
| GLB1 | -1.20842053 | 5.419437998 | -11.11534392 | 2.76E-26 | 2.01E-25 | 48.27139533 |
| GLIPR2 | 1.313851858 | 4.282694161 | 11.11042992 | 2.89E-26 | 2.10E-25 | 48.22588823 |
| GLIS1 | 1.573344372 | 3.841395215 | 11.09999763 | 3.19E-26 | 2.31E-25 | 48.12932247 |
| GJA1 | 1.638146797 | 7.353207871 | 11.09863535 | 3.23E-26 | 2.34E-25 | 48.11671707 |
| TMEM25 | -1.190746741 | 3.765380075 | -11.09039021 | 3.49E-26 | 2.52E-25 | 48.04044541 |
| WWC3 | 1.122648321 | 5.01745104 | 11.08736184 | 3.59E-26 | 2.60E-25 | 48.01244106 |
| POLG2 | 1.032507936 | 2.913679249 | 11.07284894 | 4.11E-26 | 2.96E-25 | 47.87830581 |
| TAP2 | 1.333208876 | 4.108549332 | 11.07228155 | 4.13E-26 | 2.98E-25 | 47.87306416 |
| TRAF3IP2 | 1.09844195 | 5.629352315 | 11.07224665 | 4.13E-26 | 2.98E-25 | 47.87274169 |
| PLCG2 | -1.451613242 | 4.700013258 | -11.07174801 | 4.15E-26 | 2.99E-25 | 47.86813524 |
| SLAMF8 | 1.379628016 | 2.387239898 | 11.06323004 | 4.49E-26 | 3.23E-25 | 47.78946794 |
| AGER | 1.355601539 | 3.47981065 | 11.06260849 | 4.52E-26 | 3.25E-25 | 47.78372925 |
| ALOX15B | 1.535744468 | 1.806173771 | 11.059958 | 4.63E-26 | 3.33E-25 | 47.75925995 |
| SSBP4 | 1.094158881 | 6.395554084 | 11.05816069 | 4.71E-26 | 3.38E-25 | 47.74266941 |
| IL2RG | 1.442768868 | 3.520842065 | 11.05674948 | 4.77E-26 | 3.42E-25 | 47.72964416 |
| MYBL1 | 1.000199317 | 2.444628253 | 11.05297467 | 4.94E-26 | 3.54E-25 | 47.69480862 |
| PLG | -2.784022803 | 1.999776861 | -11.04018087 | 5.56E-26 | 3.98E-25 | 47.5768013 |
| SLC28A1 | 2.289415995 | 5.998457907 | 11.03669125 | 5.75E-26 | 4.11E-25 | 47.54462965 |
| ALDOC | 1.968167894 | 5.109057094 | 11.03603886 | 5.78E-26 | 4.13E-25 | 47.53861589 |
| GOLGA7B | 1.074990698 | 2.571901179 | 11.03334781 | 5.93E-26 | 4.24E-25 | 47.51381203 |
| KLF5 | -1.443034357 | 4.450153473 | -11.02917004 | 6.16E-26 | 4.40E-25 | 47.47531299 |
| RASSF9 | -1.190477261 | 2.541392818 | -11.02808318 | 6.23E-26 | 4.44E-25 | 47.46529893 |
| SLC10A6 | 1.136245134 | 3.317228873 | 11.02785862 | 6.24E-26 | 4.45E-25 | 47.46322993 |
| CRYGS | 1.145315127 | 2.084778067 | 11.02375469 | 6.48E-26 | 4.62E-25 | 47.42542382 |
| LAG3 | 2.008042839 | 3.502085491 | 11.00584183 | 7.65E-26 | 5.44E-25 | 47.2605178 |
| ICOS | 1.118435949 | 2.238761146 | 11.00049655 | 8.04E-26 | 5.71E-25 | 47.21134401 |
| COL27A1 | 1.388025259 | 4.968218275 | 10.99959942 | 8.11E-26 | 5.75E-25 | 47.20309237 |
| LCAT | 1.399292971 | 3.648299805 | 10.99692475 | 8.31E-26 | 5.90E-25 | 47.17849418 |
| CENPT | 1.023501594 | 3.299495108 | 10.98942518 | 8.91E-26 | 6.31E-25 | 47.10954402 |
| P2RY10 | 1.330235251 | 2.843608438 | 10.98652393 | 9.15E-26 | 6.48E-25 | 47.08287878 |
| PBX1 | -1.353402826 | 4.31591926 | -10.98363002 | 9.40E-26 | 6.65E-25 | 47.05628569 |
| TRAT1 | 1.220786676 | 3.055785219 | 10.9736566 | 1.03E-25 | 7.28E-25 | 46.96467268 |
| MAP9 | -1.158106123 | 3.752623628 | -10.97301465 | 1.04E-25 | 7.32E-25 | 46.95877777 |
| PLXNC1 | 1.463448052 | 3.393776542 | 10.96360421 | 1.13E-25 | 7.98E-25 | 46.87239092 |
| APBB3 | 1.069033957 | 4.413525735 | 10.96318523 | 1.14E-25 | 8.01E-25 | 46.86854587 |
| SLC22A13 | -1.623630937 | 1.452923788 | -10.96111514 | 1.16E-25 | 8.16E-25 | 46.84954986 |
| ADAMDEC1 | 1.833936341 | 2.247233596 | 10.95187061 | 1.26E-25 | 8.88E-25 | 46.76474759 |
| NEBL | -1.477415619 | 3.872149107 | -10.93645952 | 1.45E-25 | 1.02E-24 | 46.62348509 |
| SPATA18 | 1.491404479 | 4.659332376 | 10.93288487 | 1.50E-25 | 1.06E-24 | 46.59073801 |
| GRK5 | 1.096873472 | 4.089196685 | 10.9307216 | 1.53E-25 | 1.08E-24 | 46.5709239 |
| VWA5A | -1.048072159 | 4.701152697 | -10.9280945 | 1.57E-25 | 1.10E-24 | 46.54686512 |
| EXOC3L1 | 1.232718755 | 5.20822046 | 10.92413776 | 1.63E-25 | 1.14E-24 | 46.51063683 |
| CSF2RB | 1.185486305 | 3.566650151 | 10.91571045 | 1.76E-25 | 1.23E-24 | 46.43350513 |
| FBLN1 | -1.732425519 | 4.395627764 | -10.91548006 | 1.76E-25 | 1.24E-24 | 46.43139703 |
| SEMA3G | -1.715048676 | 3.855074823 | -10.9018248 | 2.00E-25 | 1.40E-24 | 46.30650327 |
| LRBA | -1.059893867 | 4.836770378 | -10.9016781 | 2.00E-25 | 1.40E-24 | 46.30516204 |
| FSCN1 | 1.41701713 | 5.524876089 | 10.88703477 | 2.29E-25 | 1.60E-24 | 46.1713499 |
| PANK1 | -1.371344653 | 3.822871608 | -10.88370429 | 2.36E-25 | 1.65E-24 | 46.14093263 |
| EIF1B | -1.023395023 | 5.366971845 | -10.87865702 | 2.48E-25 | 1.72E-24 | 46.09484795 |
| TRAF3IP3 | 1.030064777 | 3.211627457 | 10.86574228 | 2.79E-25 | 1.94E-24 | 45.97699433 |
| KCNK9 | 1.468345349 | 1.708161583 | 10.86532538 | 2.80E-25 | 1.94E-24 | 45.97319146 |
| MTMR11 | 1.425313515 | 5.322511123 | 10.86498 | 2.81E-25 | 1.95E-24 | 45.97004109 |
| PIGH | -1.000337471 | 4.508205373 | -10.86008835 | 2.94E-25 | 2.03E-24 | 45.92542922 |
| PIK3CG | 1.094553098 | 2.758567937 | 10.85868917 | 2.98E-25 | 2.06E-24 | 45.91267118 |
| ARHGAP42 | 1.224215078 | 5.126142042 | 10.85808204 | 2.99E-25 | 2.07E-24 | 45.90713556 |
| CDCA7L | 1.364554883 | 4.546474843 | 10.84767388 | 3.29E-25 | 2.28E-24 | 45.81227024 |
| CLEC5A | 1.148105071 | 3.088872991 | 10.84251187 | 3.45E-25 | 2.39E-24 | 45.76524392 |
| CD8B | 1.411069647 | 2.800492619 | 10.83621168 | 3.66E-25 | 2.53E-24 | 45.70786938 |
| GIT2 | 1.156117925 | 3.99520746 | 10.83550032 | 3.68E-25 | 2.54E-24 | 45.70139258 |
| NRBP2 | 1.143650143 | 5.177163079 | 10.83386794 | 3.74E-25 | 2.58E-24 | 45.6865311 |
| MAN2B1 | 1.128638282 | 5.284499715 | 10.83213679 | 3.80E-25 | 2.62E-24 | 45.67077215 |
| ITGAD | 1.00581742 | 2.275542451 | 10.83000118 | 3.87E-25 | 2.67E-24 | 45.65133372 |
| ANO1 | 1.570927955 | 4.541309248 | 10.82954161 | 3.89E-25 | 2.68E-24 | 45.64715101 |
| SYNGR1 | -1.221890878 | 3.889815992 | -10.82842452 | 3.93E-25 | 2.71E-24 | 45.63698445 |
| ARHGEF3 | -1.114626883 | 4.193432804 | -10.82159824 | 4.18E-25 | 2.88E-24 | 45.57487481 |
| NLRP1 | 1.078008982 | 3.705223337 | 10.8204724 | 4.23E-25 | 2.91E-24 | 45.56463382 |
| ETNK2 | -1.432562074 | 4.577285084 | -10.81146034 | 4.59E-25 | 3.15E-24 | 45.48268318 |
| CETP | 1.111254573 | 3.862820823 | 10.79384529 | 5.39E-25 | 3.69E-24 | 45.32263601 |
| TLR6 | 1.002637486 | 2.470768315 | 10.78430038 | 5.89E-25 | 4.02E-24 | 45.23598702 |
| SLC6A8 | 1.473182141 | 7.059247144 | 10.78291458 | 5.96E-25 | 4.07E-24 | 45.22341103 |
| VAMP5 | 1.061235748 | 7.755217981 | 10.78105818 | 6.06E-25 | 4.14E-24 | 45.20656605 |
| NPDC1 | 1.205422868 | 4.656131469 | 10.77232146 | 6.57E-25 | 4.47E-24 | 45.12731592 |
| GIMAP2 | 1.465890754 | 4.617514387 | 10.77166913 | 6.61E-25 | 4.50E-24 | 45.1214004 |
| GPRC5B | -1.23429411 | 6.087161186 | -10.77094997 | 6.65E-25 | 4.52E-24 | 45.11487919 |
| NEK2 | 1.087774817 | 1.9400899 | 10.76531088 | 7.00E-25 | 4.76E-24 | 45.0637552 |
| KCNIP1 | -1.164238855 | 2.193193692 | -10.76224514 | 7.20E-25 | 4.89E-24 | 45.03596885 |
| CDCA8 | 1.106355783 | 2.417051484 | 10.7562688 | 7.61E-25 | 5.16E-24 | 44.98181776 |
| ZNF276 | 1.034207367 | 2.591459251 | 10.75048031 | 8.02E-25 | 5.44E-24 | 44.9293884 |
| MYEOV | 2.637968942 | 3.487613342 | 10.74931225 | 8.10E-25 | 5.49E-24 | 44.91881099 |
| LMO7 | -1.127721115 | 4.970964238 | -10.74187824 | 8.67E-25 | 5.86E-24 | 44.85151054 |
| TCERG1 | 1.079572232 | 3.855837175 | 10.73681146 | 9.08E-25 | 6.14E-24 | 44.805659 |
| CERKL | -1.091708726 | 2.267278446 | -10.73520009 | 9.22E-25 | 6.22E-24 | 44.79108015 |
| DCDC2 | -1.808001313 | 4.337050384 | -10.7315843 | 9.53E-25 | 6.43E-24 | 44.75837167 |
| RARA | 1.022562546 | 5.182785113 | 10.72791648 | 9.85E-25 | 6.64E-24 | 44.72520029 |
| C3orf70 | 1.147623174 | 2.735322803 | 10.72450397 | 1.02E-24 | 6.84E-24 | 44.6943448 |
| GPHN | -1.086300794 | 4.218367687 | -10.72442099 | 1.02E-24 | 6.85E-24 | 44.69359463 |
| ZNRF3 | -1.047756803 | 4.88332215 | -10.71164176 | 1.14E-24 | 7.69E-24 | 44.57810688 |
| LMTK3 | -1.108045822 | 2.862179956 | -10.71101588 | 1.15E-24 | 7.73E-24 | 44.57245318 |
| RNASEK | 1.313937038 | 2.189416297 | 10.70194609 | 1.25E-24 | 8.38E-24 | 44.4905488 |
| NGF | 1.568530237 | 3.710966478 | 10.69933591 | 1.28E-24 | 8.57E-24 | 44.46698648 |
| RCSD1 | 1.079482056 | 4.598562712 | 10.69539147 | 1.32E-24 | 8.88E-24 | 44.43138717 |
| ALPK2 | 1.688085338 | 5.163199185 | 10.68553452 | 1.45E-24 | 9.69E-24 | 44.34246569 |
| PLVAP | 1.672589355 | 8.966628302 | 10.68440949 | 1.46E-24 | 9.78E-24 | 44.33232019 |
| CD248 | 1.554998665 | 6.556754247 | 10.68194689 | 1.50E-24 | 9.99E-24 | 44.31011505 |
| ADCY7 | 1.201028812 | 2.050267301 | 10.67955871 | 1.53E-24 | 1.02E-23 | 44.28858419 |
| KSR1 | 1.650245089 | 4.142969582 | 10.67791009 | 1.55E-24 | 1.04E-23 | 44.27372292 |
| SH2B3 | 1.179857815 | 4.932335384 | 10.67700681 | 1.57E-24 | 1.04E-23 | 44.26558108 |
| MAP4K3 | -1.294209716 | 4.149876214 | -10.67182032 | 1.64E-24 | 1.09E-23 | 44.21884099 |
| ADRA2C | -1.457541091 | 3.342726891 | -10.66819244 | 1.70E-24 | 1.13E-23 | 44.18615628 |
| DSCC1 | 1.880438332 | 3.282548398 | 10.66698432 | 1.71E-24 | 1.14E-23 | 44.17527362 |
| CDC20 | 1.250633077 | 3.539554498 | 10.65769998 | 1.87E-24 | 1.24E-23 | 44.09166927 |
| PTPN7 | 1.513129926 | 2.70595643 | 10.65717093 | 1.87E-24 | 1.24E-23 | 44.0869068 |
| SLC39A14 | 1.631244941 | 6.537504998 | 10.6529993 | 1.95E-24 | 1.29E-23 | 44.04935933 |
| ATP2B2 | 1.511729316 | 2.598069648 | 10.6518478 | 1.97E-24 | 1.30E-23 | 44.03899689 |
| GABRE | 1.616331296 | 2.596564067 | 10.64427332 | 2.11E-24 | 1.39E-23 | 43.97085258 |
| GOT1 | -1.132424215 | 6.743761134 | -10.64120399 | 2.17E-24 | 1.43E-23 | 43.94324858 |
| C3orf36 | 1.368810245 | 2.648057312 | 10.63977976 | 2.20E-24 | 1.45E-23 | 43.9304417 |
| ST3GAL6 | -1.017571606 | 4.576666287 | -10.63852691 | 2.22E-24 | 1.46E-23 | 43.91917686 |
| TLR8 | 1.491480777 | 3.415174649 | 10.63547497 | 2.28E-24 | 1.50E-23 | 43.89173952 |
| ACAT1 | -1.308056849 | 6.199621147 | -10.63418927 | 2.31E-24 | 1.52E-23 | 43.88018253 |
| SVEP1 | -1.323596203 | 3.309461624 | -10.62648576 | 2.48E-24 | 1.63E-23 | 43.81095684 |
| TNFRSF9 | 1.558664961 | 1.859741923 | 10.62039774 | 2.62E-24 | 1.72E-23 | 43.75627289 |
| GRB7 | -1.230717671 | 5.500542133 | -10.61433853 | 2.76E-24 | 1.81E-23 | 43.70186915 |
| GPX2 | -1.163497244 | 2.392347236 | -10.61398976 | 2.77E-24 | 1.82E-23 | 43.6987383 |
| CADM1 | -1.418223838 | 4.261922372 | -10.60746282 | 2.94E-24 | 1.93E-23 | 43.64016021 |
| PIGR | -3.283572655 | 4.625856807 | -10.60687732 | 2.96E-24 | 1.94E-23 | 43.6349067 |
| PARP10 | 1.057767826 | 6.158663277 | 10.6049627 | 3.01E-24 | 1.97E-23 | 43.61772872 |
| IGSF3 | -1.159229671 | 4.208342249 | -10.60444666 | 3.02E-24 | 1.98E-23 | 43.61309918 |
| PSCA | -1.176525612 | 0.825193524 | -10.59731234 | 3.22E-24 | 2.10E-23 | 43.54911113 |
| C5AR1 | 1.238991787 | 4.539772583 | 10.59438814 | 3.31E-24 | 2.16E-23 | 43.52289237 |
| MYO10 | -1.115239104 | 4.554787924 | -10.59099141 | 3.41E-24 | 2.23E-23 | 43.49244319 |
| CDK5RAP3 | 1.198628098 | 1.862244604 | 10.59035506 | 3.43E-24 | 2.24E-23 | 43.48673956 |
| OR51E2 | 1.199368533 | 2.310382536 | 10.58932907 | 3.47E-24 | 2.26E-23 | 43.47754406 |
| FLI1 | 1.09669978 | 4.223043952 | 10.58662979 | 3.55E-24 | 2.31E-23 | 43.45335437 |
| CDC34 | 1.052475158 | 5.899881946 | 10.58218498 | 3.70E-24 | 2.40E-23 | 43.41353148 |
| SLC17A4 | 2.60543397 | 4.202319724 | 10.57830223 | 3.83E-24 | 2.49E-23 | 43.37875383 |
| RPS21 | 1.103274321 | 8.980781023 | 10.57694704 | 3.88E-24 | 2.52E-23 | 43.36661749 |
| GPR176 | 1.21790303 | 4.808016693 | 10.57604108 | 3.91E-24 | 2.54E-23 | 43.35850485 |
| FUT3 | -1.31737996 | 2.685478906 | -10.57524931 | 3.94E-24 | 2.55E-23 | 43.35141508 |
| SLCO2B1 | 1.556217487 | 4.291778199 | 10.56997153 | 4.13E-24 | 2.67E-23 | 43.30416579 |
| RAB15 | -1.141009448 | 3.316448592 | -10.56076211 | 4.49E-24 | 2.90E-23 | 43.22175751 |
| CPNE5 | 1.280553403 | 2.435381661 | 10.55891008 | 4.56E-24 | 2.95E-23 | 43.20519109 |
| INSR | 1.22858637 | 6.180964047 | 10.55563494 | 4.70E-24 | 3.04E-23 | 43.1758999 |
| C2 | 1.705357073 | 4.199277473 | 10.54479118 | 5.18E-24 | 3.34E-23 | 43.07896364 |
| PRLR | -1.504164412 | 3.813093741 | -10.54303945 | 5.26E-24 | 3.39E-23 | 43.06331075 |
| CCDC102B | 1.108574879 | 4.063998074 | 10.54204509 | 5.31E-24 | 3.42E-23 | 43.05442632 |
| DYSF | 1.306649745 | 6.390023047 | 10.54194343 | 5.31E-24 | 3.43E-23 | 43.05351804 |
| G6PC | -2.344333866 | 2.370535636 | -10.54079368 | 5.37E-24 | 3.46E-23 | 43.04324599 |
| PRR16 | 1.139471019 | 3.541534306 | 10.52960237 | 5.94E-24 | 3.82E-23 | 42.9433017 |
| AIM2 | 1.396048338 | 1.919446581 | 10.5278278 | 6.03E-24 | 3.88E-23 | 42.92746063 |
| NRARP | 1.370015026 | 4.510466429 | 10.52616402 | 6.13E-24 | 3.94E-23 | 42.91261024 |
| ANK3 | -1.318765195 | 4.028735409 | -10.522609 | 6.32E-24 | 4.06E-23 | 42.88088466 |
| TUBB2A | -1.019010991 | 4.835478097 | -10.52204226 | 6.36E-24 | 4.08E-23 | 42.87582763 |
| RERGL | -1.56782256 | 2.566583748 | -10.51812794 | 6.59E-24 | 4.22E-23 | 42.84090558 |
| DCLK1 | 1.297788624 | 3.312277907 | 10.51198756 | 6.96E-24 | 4.45E-23 | 42.78614156 |
| KLHL21 | -1.021712704 | 4.891117179 | -10.50850783 | 7.18E-24 | 4.59E-23 | 42.7551169 |
| ATP8B3 | 1.487729437 | 3.331550965 | 10.50782809 | 7.22E-24 | 4.62E-23 | 42.74905728 |
| FMO4 | -1.019369075 | 3.823222421 | -10.50727822 | 7.26E-24 | 4.64E-23 | 42.74415564 |
| CYP3A5 | 1.331771029 | 3.806458955 | 10.48263363 | 9.06E-24 | 5.76E-23 | 42.52465124 |
| C19orf38 | 1.182367757 | 2.765753354 | 10.47428086 | 9.76E-24 | 6.20E-23 | 42.45033634 |
| OR7E47P | 1.340010025 | 2.782394723 | 10.47341284 | 9.84E-24 | 6.25E-23 | 42.44261589 |
| XAF1 | 1.249096019 | 3.447736679 | 10.47294731 | 9.88E-24 | 6.27E-23 | 42.43847551 |
| ADAM19 | 1.210111687 | 3.222649172 | 10.46448441 | 1.07E-23 | 6.76E-23 | 42.36322955 |
| EPHX2 | -1.345136781 | 5.870824249 | -10.4622041 | 1.09E-23 | 6.89E-23 | 42.34296192 |
| CLCN5 | -1.381683023 | 4.787414699 | -10.46070005 | 1.10E-23 | 6.98E-23 | 42.3295955 |
| F2RL3 | 1.878118453 | 3.922672253 | 10.45915898 | 1.12E-23 | 7.07E-23 | 42.31590145 |
| CTLA4 | 1.136988494 | 2.822623178 | 10.45818487 | 1.13E-23 | 7.13E-23 | 42.30724619 |
| GBP1 | 1.488696378 | 6.147988064 | 10.45065797 | 1.21E-23 | 7.62E-23 | 42.24038622 |
| LZTR1 | 1.009525606 | 3.080837802 | 10.44721588 | 1.24E-23 | 7.85E-23 | 42.20982202 |
| CYTIP | 1.136210992 | 3.562551133 | 10.4463958 | 1.25E-23 | 7.91E-23 | 42.20254113 |
| GPR34 | 1.61054337 | 5.589287628 | 10.42538536 | 1.51E-23 | 9.51E-23 | 42.01614077 |
| STAT2 | 1.007929109 | 6.144456113 | 10.42493891 | 1.52E-23 | 9.55E-23 | 42.01218286 |
| NFE2L3 | 1.140079226 | 3.653688242 | 10.42253001 | 1.55E-23 | 9.75E-23 | 41.99082896 |
| ANG | 1.341655285 | 3.94398435 | 10.41996701 | 1.59E-23 | 9.97E-23 | 41.9681129 |
| CDCA7 | 1.087620185 | 3.143934786 | 10.41911785 | 1.60E-23 | 1.00E-22 | 41.96058754 |
| VMP1 | 1.115373113 | 5.842414335 | 10.41703677 | 1.63E-23 | 1.02E-22 | 41.94214674 |
| RPS5 | 1.030387584 | 8.37295762 | 10.4169737 | 1.63E-23 | 1.02E-22 | 41.94158784 |
| OAS2 | 1.194200304 | 5.265517504 | 10.41595317 | 1.65E-23 | 1.03E-22 | 41.93254575 |
| PLLP | -1.073571145 | 3.334740441 | -10.4155219 | 1.65E-23 | 1.03E-22 | 41.92872479 |
| PDCD1 | 1.594146303 | 3.307028153 | 10.40585629 | 1.80E-23 | 1.13E-22 | 41.84311838 |
| STYK1 | -1.038082462 | 1.427556227 | -10.39877667 | 1.92E-23 | 1.20E-22 | 41.78045097 |
| CCNL2 | 1.361759956 | 5.633881559 | 10.39592688 | 1.97E-23 | 1.23E-22 | 41.75523361 |
| KCNJ16 | -1.756070342 | 6.34346025 | -10.38896079 | 2.09E-23 | 1.30E-22 | 41.69361213 |
| ACLY | 1.230644824 | 6.935617655 | 10.38845956 | 2.10E-23 | 1.31E-22 | 41.68917938 |
| ADAM28 | 1.248552638 | 2.942238715 | 10.38711201 | 2.13E-23 | 1.32E-22 | 41.67726283 |
| SH3PXD2B | 1.190341751 | 3.43196732 | 10.38610084 | 2.15E-23 | 1.33E-22 | 41.66832159 |
| PCK1 | -2.865831939 | 5.996695956 | -10.36899398 | 2.50E-23 | 1.55E-22 | 41.51714794 |
| HIGD1B | 1.661265968 | 3.854301992 | 10.36565586 | 2.58E-23 | 1.60E-22 | 41.48766927 |
| BIRC7 | 2.572079809 | 2.700701081 | 10.36082564 | 2.69E-23 | 1.66E-22 | 41.44502586 |
| PTHLH | 2.579273068 | 2.853846234 | 10.35689548 | 2.79E-23 | 1.72E-22 | 41.41033889 |
| CHST15 | 1.442719122 | 4.793478679 | 10.34514021 | 3.09E-23 | 1.90E-22 | 41.30664402 |
| ASPDH | -1.673200181 | 3.180055325 | -10.33529833 | 3.38E-23 | 2.07E-22 | 41.21989113 |
| FLT1 | 1.65527507 | 5.975758198 | 10.32910162 | 3.57E-23 | 2.19E-22 | 41.16529901 |
| TAGLN2 | 1.016181731 | 9.070892967 | 10.32767535 | 3.61E-23 | 2.21E-22 | 41.15273703 |
| COBL | -1.183481265 | 3.14484919 | -10.32588119 | 3.67E-23 | 2.25E-22 | 41.13693656 |
| P4HA3 | 1.398952269 | 3.290635781 | 10.32356641 | 3.75E-23 | 2.29E-22 | 41.11655414 |
| WDFY4 | 1.241151395 | 2.684312544 | 10.31317549 | 4.11E-23 | 2.51E-22 | 41.02509793 |
| GPR174 | 1.179337467 | 2.876163895 | 10.30997907 | 4.23E-23 | 2.59E-22 | 40.99697755 |
| CEBPB | 1.193459057 | 6.903799555 | 10.30715419 | 4.34E-23 | 2.65E-22 | 40.97213091 |
| RASL11A | -1.360779663 | 4.528982345 | -10.30619058 | 4.37E-23 | 2.67E-22 | 40.9636564 |
| CDON | 1.322870749 | 2.573223207 | 10.30391891 | 4.46E-23 | 2.72E-22 | 40.94368045 |
| NLRP3 | 1.013231365 | 3.599445454 | 10.30247231 | 4.52E-23 | 2.76E-22 | 40.93096133 |
| RPL35A | 1.221735284 | 8.39933344 | 10.30241352 | 4.52E-23 | 2.76E-22 | 40.93044449 |
| CREB3L3 | 2.223192966 | 3.62378456 | 10.29788217 | 4.71E-23 | 2.87E-22 | 40.89061129 |
| SNX10 | 1.107881653 | 5.888613049 | 10.29669015 | 4.76E-23 | 2.90E-22 | 40.88013478 |
| SDC3 | 1.215568542 | 5.530656015 | 10.29085705 | 5.01E-23 | 3.05E-22 | 40.82888082 |
| LPIN3 | 1.186291335 | 4.873719869 | 10.29077255 | 5.01E-23 | 3.05E-22 | 40.8281385 |
| IGF2BP2 | -1.467803308 | 2.506188585 | -10.28122543 | 5.46E-23 | 3.32E-22 | 40.74429519 |
| DOCK8 | 1.374851632 | 5.217023029 | 10.27533175 | 5.75E-23 | 3.49E-22 | 40.69256411 |
| VWCE | 1.611695864 | 3.269700176 | 10.2685466 | 6.10E-23 | 3.70E-22 | 40.63303412 |
| GPR141 | 1.237151436 | 1.908802361 | 10.26537974 | 6.28E-23 | 3.80E-22 | 40.60525893 |
| CAPN5 | -1.088011095 | 3.803888587 | -10.26190851 | 6.47E-23 | 3.92E-22 | 40.57482128 |
| ALDH5A1 | -1.160619301 | 5.441082012 | -10.25780455 | 6.71E-23 | 4.06E-22 | 40.53884488 |
| TGFBI | 2.64312044 | 8.222956474 | 10.25478401 | 6.89E-23 | 4.17E-22 | 40.51237261 |
| ICAM1 | 1.297854261 | 6.761726421 | 10.25143588 | 7.10E-23 | 4.29E-22 | 40.48303568 |
| C14orf132 | -1.418046315 | 3.018507781 | -10.24703648 | 7.38E-23 | 4.45E-22 | 40.44449757 |
| KAZN | -1.011778936 | 3.345977583 | -10.24393959 | 7.59E-23 | 4.57E-22 | 40.41737641 |
| GYPC | 1.005323875 | 5.713388263 | 10.23822684 | 7.98E-23 | 4.81E-22 | 40.36736184 |
| ADRA1B | 1.19612022 | 4.07613706 | 10.23350086 | 8.32E-23 | 5.01E-22 | 40.32600131 |
| SH2D4A | -1.051742445 | 4.949906229 | -10.22304849 | 9.13E-23 | 5.47E-22 | 40.23457292 |
| PHGDH | -1.602700401 | 3.758277582 | -10.21012441 | 1.02E-22 | 6.13E-22 | 40.12161591 |
| BDKRB2 | -1.622691151 | 3.691221717 | -10.20977356 | 1.03E-22 | 6.14E-22 | 40.11855091 |
| FILIP1 | 1.063852697 | 2.792478161 | 10.20938994 | 1.03E-22 | 6.16E-22 | 40.1151997 |
| PLAUR | 1.417524054 | 3.865635609 | 10.20603064 | 1.06E-22 | 6.33E-22 | 40.08585733 |
| SOX11 | 1.130750016 | 3.132152033 | 10.20468955 | 1.07E-22 | 6.40E-22 | 40.0741453 |
| MEF2C | 1.125933261 | 5.319114129 | 10.20445372 | 1.08E-22 | 6.42E-22 | 40.07208586 |
| HYAL1 | -1.115477089 | 5.694790163 | -10.20350003 | 1.08E-22 | 6.47E-22 | 40.06375788 |
| LAMC2 | -1.84861269 | 3.663666696 | -10.19715719 | 1.15E-22 | 6.83E-22 | 40.00838382 |
| APOL6 | 1.014273455 | 5.376852846 | 10.19501408 | 1.17E-22 | 6.95E-22 | 39.98967966 |
| THEMIS | 1.199470415 | 1.939704197 | 10.18211412 | 1.31E-22 | 7.77E-22 | 39.87715324 |
| PLEKHB2 | -1.09494531 | 6.157782448 | -10.17670881 | 1.37E-22 | 8.14E-22 | 39.83003284 |
| SLC2A5 | 2.008128276 | 5.631119105 | 10.17589825 | 1.38E-22 | 8.19E-22 | 39.8229684 |
| SMOX | 1.12914057 | 5.695977999 | 10.15533692 | 1.66E-22 | 9.77E-22 | 39.64389936 |
| MMP9 | 2.085092489 | 4.530869647 | 10.14531836 | 1.81E-22 | 1.06E-21 | 39.55674106 |
| CCR7 | 1.141464615 | 2.247780741 | 10.14187929 | 1.86E-22 | 1.10E-21 | 39.52683647 |
| GPR171 | 1.025359885 | 2.04136181 | 10.14040084 | 1.89E-22 | 1.11E-21 | 39.51398267 |
| APOD | -1.66404226 | 4.062889409 | -10.13692552 | 1.95E-22 | 1.14E-21 | 39.48377332 |
| ETV4 | -1.180170775 | 2.680487744 | -10.13665745 | 1.95E-22 | 1.15E-21 | 39.48144341 |
| HOXD10 | -1.337823429 | 3.498820767 | -10.13342295 | 2.01E-22 | 1.18E-21 | 39.45333454 |
| LAMA2 | -1.200934142 | 2.835728246 | -10.12844589 | 2.10E-22 | 1.23E-21 | 39.41009476 |
| PHYH | -1.010315425 | 6.783539045 | -10.12773077 | 2.11E-22 | 1.24E-21 | 39.40388317 |
| PLA2G4C | 1.054510709 | 3.211277997 | 10.12334924 | 2.19E-22 | 1.28E-21 | 39.3658317 |
| ANO4 | 1.561456992 | 3.024710243 | 10.12240168 | 2.21E-22 | 1.29E-21 | 39.35760411 |
| RAP1GAP2 | -1.148280495 | 3.250598902 | -10.12152193 | 2.23E-22 | 1.30E-21 | 39.34996583 |
| TEX11 | 1.675504448 | 3.003314063 | 10.12118996 | 2.23E-22 | 1.30E-21 | 39.34708367 |
| UBXN11 | 1.01452452 | 4.805546069 | 10.12048474 | 2.25E-22 | 1.31E-21 | 39.34096121 |
| XCL1 | 1.208158627 | 3.100017614 | 10.11854156 | 2.29E-22 | 1.33E-21 | 39.32409282 |
| ACTG2 | 1.872949821 | 4.639213716 | 10.11696414 | 2.32E-22 | 1.35E-21 | 39.31040124 |
| DSP | -1.808099528 | 5.764231437 | -10.09913342 | 2.71E-22 | 1.58E-21 | 39.15574128 |
| TUBA1A | 1.192126276 | 7.047469286 | 10.09568134 | 2.79E-22 | 1.62E-21 | 39.12582121 |
| PLA1A | 1.279815507 | 6.167924352 | 10.08685375 | 3.02E-22 | 1.75E-21 | 39.04934348 |
| ADAMTSL2 | -1.359488567 | 3.828109179 | -10.08017353 | 3.20E-22 | 1.85E-21 | 38.99150142 |
| ATP8B1 | -1.120481958 | 3.630707333 | -10.06439696 | 3.67E-22 | 2.12E-21 | 38.85500555 |
| GCAT | -1.091660085 | 3.52162503 | -10.06334312 | 3.70E-22 | 2.14E-21 | 38.84589334 |
| PGM5 | -1.179078103 | 4.188585807 | -10.05582431 | 3.96E-22 | 2.28E-21 | 38.7809009 |
| TACR1 | -1.05384547 | 2.054591273 | -10.04607188 | 4.31E-22 | 2.47E-21 | 38.69665299 |
| LCN2 | -1.848097011 | 2.729379857 | -10.0443711 | 4.37E-22 | 2.51E-21 | 38.68196652 |
| PRRT2 | 1.01804235 | 1.645777384 | 10.03726753 | 4.65E-22 | 2.67E-21 | 38.62064563 |
| TNFRSF25 | 1.232385201 | 2.584024399 | 10.03469331 | 4.76E-22 | 2.73E-21 | 38.59843163 |
| FGFBP2 | 1.239041765 | 3.010233132 | 10.03359927 | 4.80E-22 | 2.75E-21 | 38.58899191 |
| TMCO6 | 1.099666163 | 4.090612678 | 10.03116453 | 4.90E-22 | 2.81E-21 | 38.56798697 |
| KCNJ11 | -1.044121135 | 1.685015258 | -10.02952913 | 4.97E-22 | 2.85E-21 | 38.55388004 |
| SLC17A2 | 1.512682665 | 2.056751286 | 10.0281301 | 5.04E-22 | 2.88E-21 | 38.54181341 |
| LAPTM4B | -1.054247662 | 7.41994967 | -10.02382766 | 5.23E-22 | 2.99E-21 | 38.50471247 |
| GGT8P | 1.532580481 | 3.535353376 | 10.01801826 | 5.50E-22 | 3.14E-21 | 38.45463485 |
| NPM2 | -1.992973955 | 2.425437407 | -10.01088277 | 5.85E-22 | 3.33E-21 | 38.3931547 |
| RGN | -1.285023129 | 5.105252083 | -9.99183903 | 6.90E-22 | 3.92E-21 | 38.22922637 |
| IRF9 | 1.186507141 | 1.881279055 | 9.991325294 | 6.93E-22 | 3.94E-21 | 38.22480725 |
| BCKDK | 1.376887257 | 6.689584446 | 9.990508422 | 6.98E-22 | 3.96E-21 | 38.21778091 |
| CCND2 | 1.58639976 | 4.676054486 | 9.98824567 | 7.12E-22 | 4.04E-21 | 38.19831998 |
| TEK | -1.520612434 | 4.620239043 | -9.982938049 | 7.46E-22 | 4.22E-21 | 38.15268394 |
| FRY | -1.013678769 | 4.766517967 | -9.982152716 | 7.51E-22 | 4.25E-21 | 38.14593296 |
| AMDHD2 | 1.17871005 | 3.535449632 | 9.974873137 | 8.00E-22 | 4.52E-21 | 38.08337361 |
| DHPS | 1.062322131 | 2.576316105 | 9.973476494 | 8.10E-22 | 4.57E-21 | 38.07137488 |
| CXCL13 | 2.026493206 | 3.590368585 | 9.969434808 | 8.38E-22 | 4.73E-21 | 38.0366591 |
| LIPG | -1.223055836 | 2.581360794 | -9.964982798 | 8.71E-22 | 4.91E-21 | 37.99843063 |
| CYBB | 1.657646309 | 6.084867564 | 9.961099108 | 9.01E-22 | 5.07E-21 | 37.96509228 |
| DLK2 | 1.019628357 | 2.134460326 | 9.953293784 | 9.64E-22 | 5.42E-21 | 37.89811824 |
| NID1 | 1.322519781 | 6.105937886 | 9.948832256 | 1.00E-21 | 5.62E-21 | 37.85985287 |
| CLMP | -1.68957577 | 2.47093177 | -9.94354243 | 1.05E-21 | 5.88E-21 | 37.81449948 |
| ECSCR | 1.040572707 | 2.888423014 | 9.942841958 | 1.06E-21 | 5.92E-21 | 37.80849514 |
| SNAP25 | 1.358396207 | 2.613308466 | 9.940669476 | 1.08E-21 | 6.03E-21 | 37.78987493 |
| DOCK6 | 1.008540648 | 4.053665373 | 9.937968223 | 1.10E-21 | 6.17E-21 | 37.76672676 |
| ACE | 1.25055813 | 4.579848489 | 9.93682264 | 1.11E-21 | 6.23E-21 | 37.75691115 |
| PIPOX | -1.831618631 | 4.384985402 | -9.90904012 | 1.41E-21 | 7.87E-21 | 37.51911517 |
| EFNA1 | 1.082719382 | 7.527668697 | 9.905860175 | 1.45E-21 | 8.09E-21 | 37.49192816 |
| LRRC66 | 1.082236509 | 1.850925816 | 9.904183308 | 1.47E-21 | 8.20E-21 | 37.4775943 |
| SLMAP | -1.047189019 | 3.560474819 | -9.893318653 | 1.62E-21 | 8.98E-21 | 37.38476584 |
| HLA-DPB2 | 1.378060342 | 2.084120544 | 9.89153332 | 1.64E-21 | 9.12E-21 | 37.36951889 |
| RAB32 | 1.078233237 | 5.919309829 | 9.889374212 | 1.68E-21 | 9.28E-21 | 37.35108252 |
| VKORC1 | 1.086581574 | 4.499417713 | 9.884444696 | 1.75E-21 | 9.67E-21 | 37.3090009 |
| KLHL15 | -1.031683652 | 3.321873538 | -9.879222693 | 1.83E-21 | 1.01E-20 | 37.26443904 |
| GYG2 | -1.184162468 | 2.797253592 | -9.876312757 | 1.87E-21 | 1.04E-20 | 37.23961458 |
| SIPA1L2 | 1.083199156 | 4.268219742 | 9.873357206 | 1.92E-21 | 1.06E-20 | 37.21440641 |
| SCAMP5 | -1.026486038 | 5.128691028 | -9.853090502 | 2.29E-21 | 1.26E-20 | 37.04169754 |
| PLS3 | -1.08383437 | 6.856975776 | -9.842543295 | 2.51E-21 | 1.38E-20 | 36.95191843 |
| SH3RF3 | 1.050476653 | 3.703782264 | 9.839572056 | 2.57E-21 | 1.41E-20 | 36.92663951 |
| ANKDD1A | 1.073666725 | 3.192945858 | 9.833709459 | 2.70E-21 | 1.48E-20 | 36.8767776 |
| HEYL | 1.483046954 | 5.790247283 | 9.829113555 | 2.81E-21 | 1.54E-20 | 36.83770416 |
| PAQR6 | 1.442055768 | 3.514801058 | 9.829002882 | 2.82E-21 | 1.54E-20 | 36.8367634 |
| MPEG1 | 1.565041643 | 5.404269526 | 9.819734048 | 3.05E-21 | 1.67E-20 | 36.75800279 |
| SEMA3F | 1.060380827 | 6.299828996 | 9.81722233 | 3.11E-21 | 1.70E-20 | 36.73666915 |
| FN1 | 1.641922054 | 7.033858936 | 9.812384109 | 3.25E-21 | 1.77E-20 | 36.69558623 |
| RASGRP3 | 1.042840637 | 4.514095189 | 9.811203334 | 3.28E-21 | 1.79E-20 | 36.68556212 |
| GJB1 | -1.526588395 | 4.014691769 | -9.810449719 | 3.30E-21 | 1.80E-20 | 36.67916482 |
| IRF1 | 1.062689743 | 4.888447999 | 9.801155809 | 3.57E-21 | 1.95E-20 | 36.60029998 |
| FGFR2 | -1.257879988 | 4.484319107 | -9.801058404 | 3.58E-21 | 1.95E-20 | 36.59947372 |
| LIX1 | -1.854736662 | 2.690059642 | -9.799155281 | 3.64E-21 | 1.98E-20 | 36.58333134 |
| ABCA12 | 1.138652743 | 2.853674021 | 9.79798255 | 3.67E-21 | 2.00E-20 | 36.57338531 |
| SCNN1D | 1.109352553 | 2.556194987 | 9.795695668 | 3.75E-21 | 2.03E-20 | 36.55399258 |
| NDRG1 | 1.212685412 | 7.96644603 | 9.793565897 | 3.81E-21 | 2.07E-20 | 36.53593513 |
| MET | 1.083507952 | 7.442993651 | 9.793102144 | 3.83E-21 | 2.08E-20 | 36.53200354 |
| EYA2 | -1.083898173 | 2.818834694 | -9.788076488 | 4.00E-21 | 2.17E-20 | 36.4894059 |
| CKMT2 | -1.999221716 | 3.8926473 | -9.786445611 | 4.05E-21 | 2.20E-20 | 36.47558596 |
| HLA-DMB | 1.180925802 | 4.956823638 | 9.776784524 | 4.40E-21 | 2.39E-20 | 36.39375308 |
| CPVL | -1.404945988 | 6.517079834 | -9.768358722 | 4.73E-21 | 2.56E-20 | 36.32243175 |
| LTC4S | 1.259997154 | 3.389994043 | 9.751403856 | 5.47E-21 | 2.95E-20 | 36.17905136 |
| SCGN | 2.816909384 | 5.388931337 | 9.748490716 | 5.61E-21 | 3.02E-20 | 36.15443448 |
| CTF1 | -1.170688607 | 3.356456953 | -9.745037889 | 5.77E-21 | 3.11E-20 | 36.12526407 |
| PDCD6IP | -1.090922082 | 5.359556524 | -9.73854963 | 6.10E-21 | 3.28E-20 | 36.07046997 |
| CR1 | -1.007475746 | 1.247745044 | -9.736859576 | 6.19E-21 | 3.33E-20 | 36.05620166 |
| TUBA1B | 1.0976393 | 5.5171086 | 9.734387129 | 6.32E-21 | 3.40E-20 | 36.03533126 |
| DDX39B | 1.352451681 | 4.853592029 | 9.720536552 | 7.12E-21 | 3.81E-20 | 35.91848772 |
| CPEB4 | -1.045549368 | 4.614804837 | -9.717316377 | 7.31E-21 | 3.92E-20 | 35.89133978 |
| GLRB | -1.480865389 | 2.97630208 | -9.716027842 | 7.39E-21 | 3.96E-20 | 35.88047855 |
| C16orf74 | 1.483676485 | 3.206901722 | 9.703708586 | 8.21E-21 | 4.38E-20 | 35.77669118 |
| TNFAIP2 | 1.189774434 | 6.381094107 | 9.691473995 | 9.11E-21 | 4.85E-20 | 35.67371284 |
| SH3D21 | 1.221633378 | 3.82712604 | 9.67827482 | 1.02E-20 | 5.42E-20 | 35.56272271 |
| PAH | -2.50731626 | 2.849343185 | -9.676162629 | 1.04E-20 | 5.52E-20 | 35.5449719 |
| NGFR | 1.689907271 | 4.480548108 | 9.666776081 | 1.12E-20 | 5.96E-20 | 35.46612203 |
| PPL | -1.230561483 | 4.461717648 | -9.649664325 | 1.30E-20 | 6.88E-20 | 35.32252309 |
| SLPI | -3.167357446 | 5.086287407 | -9.647254897 | 1.33E-20 | 7.02E-20 | 35.30231864 |
| LPAR6 | 1.188741082 | 5.88173133 | 9.645021684 | 1.35E-20 | 7.14E-20 | 35.28359517 |
| AIF1 | 1.147667384 | 6.479362883 | 9.644792443 | 1.35E-20 | 7.15E-20 | 35.28167337 |
| PTAFR | 1.226790494 | 4.567677605 | 9.643702524 | 1.37E-20 | 7.22E-20 | 35.27253671 |
| HLA-DOB | 1.230949684 | 3.908284045 | 9.635081508 | 1.47E-20 | 7.75E-20 | 35.20029458 |
| CDH4 | 1.99468251 | 3.631165948 | 9.627405364 | 1.57E-20 | 8.27E-20 | 35.13601038 |
| ARHGAP17 | 1.027884659 | 4.362294647 | 9.623730679 | 1.62E-20 | 8.52E-20 | 35.10524996 |
| CLK2 | 1.059017675 | 4.592099212 | 9.61722091 | 1.71E-20 | 8.99E-20 | 35.05077861 |
| CEP170 | 1.17903446 | 3.13797963 | 9.613115906 | 1.77E-20 | 9.30E-20 | 35.01644344 |
| ICA1 | -1.29694466 | 3.930750514 | -9.609196887 | 1.83E-20 | 9.61E-20 | 34.983674 |
| NKAIN1 | 1.073513861 | 1.381640134 | 9.60608808 | 1.88E-20 | 9.86E-20 | 34.95768629 |
| MCHR1 | 2.085226007 | 3.394985418 | 9.599886408 | 1.98E-20 | 1.04E-19 | 34.90586269 |
| PRKD1 | -1.076089509 | 4.815088718 | -9.595018501 | 2.06E-20 | 1.08E-19 | 34.8652019 |
| MFHAS1 | -1.036808311 | 4.65965112 | -9.589847325 | 2.15E-20 | 1.13E-19 | 34.82202465 |
| C1QL1 | 2.474881794 | 3.933346302 | 9.585630813 | 2.23E-20 | 1.17E-19 | 34.78683122 |
| TSPAN18 | 1.360549689 | 5.179711118 | 9.580053737 | 2.34E-20 | 1.22E-19 | 34.74029932 |
| KCNK3 | 1.995055201 | 4.18713614 | 9.575932676 | 2.42E-20 | 1.26E-19 | 34.70592846 |
| BST2 | 1.252149974 | 7.804461423 | 9.573077556 | 2.48E-20 | 1.29E-19 | 34.68212234 |
| IFI27 | 1.2006869 | 6.020580554 | 9.569386119 | 2.56E-20 | 1.33E-19 | 34.65135077 |
| PLEKHA6 | -1.032145162 | 4.783583293 | -9.569309671 | 2.56E-20 | 1.33E-19 | 34.65071359 |
| IL6R | 1.138776742 | 4.761044133 | 9.56587285 | 2.64E-20 | 1.37E-19 | 34.62207255 |
| CST6 | -1.365028279 | 1.576516077 | -9.563203003 | 2.70E-20 | 1.40E-19 | 34.59982841 |
| NFASC | -1.333592374 | 3.489628388 | -9.553602889 | 2.92E-20 | 1.51E-19 | 34.51988199 |
| FSTL1 | 1.287109692 | 6.925240846 | 9.548567106 | 3.05E-20 | 1.58E-19 | 34.47796955 |
| ADH6 | -1.505222838 | 3.627717254 | -9.545173943 | 3.14E-20 | 1.62E-19 | 34.44973775 |
| CPA3 | 1.656046235 | 4.14852839 | 9.532795561 | 3.48E-20 | 1.80E-19 | 34.34681025 |
| DBNL | 1.168982444 | 4.887629825 | 9.531339483 | 3.52E-20 | 1.82E-19 | 34.33470934 |
| APOLD1 | 1.755923656 | 6.390494564 | 9.52967568 | 3.57E-20 | 1.84E-19 | 34.32088378 |
| NOTCH4 | 1.311929001 | 5.015428417 | 9.529600261 | 3.58E-20 | 1.84E-19 | 34.32025712 |
| ACOX2 | -1.204546011 | 5.026589336 | -9.519316485 | 3.90E-20 | 2.01E-19 | 34.2348432 |
| FRAS1 | -1.351381241 | 4.082495877 | -9.515166125 | 4.04E-20 | 2.08E-19 | 34.20039098 |
| PDGFD | 1.536667773 | 6.255381838 | 9.507633093 | 4.30E-20 | 2.21E-19 | 34.13788768 |
| SLC16A6 | 1.132172367 | 2.542455033 | 9.50483952 | 4.40E-20 | 2.26E-19 | 34.11471812 |
| SCN4B | 1.432973403 | 3.708699271 | 9.504720809 | 4.40E-20 | 2.26E-19 | 34.11373365 |
| CXCR2P1 | 1.564451676 | 1.891270367 | 9.500620056 | 4.56E-20 | 2.34E-19 | 34.07973199 |
| CYP4F3 | -1.59129665 | 2.343279576 | -9.499288603 | 4.61E-20 | 2.36E-19 | 34.0686945 |
| C1orf210 | -1.794097137 | 3.988824451 | -9.497893007 | 4.66E-20 | 2.39E-19 | 34.05712653 |
| CD200R1 | 1.075498924 | 3.335592867 | 9.487527052 | 5.09E-20 | 2.60E-19 | 33.97124357 |
| EGFR | 1.236655355 | 6.641641612 | 9.479176804 | 5.45E-20 | 2.79E-19 | 33.90211174 |
| EME2 | 1.076184053 | 3.199196844 | 9.476370243 | 5.58E-20 | 2.85E-19 | 33.87888636 |
| MAF | 1.128991848 | 6.961747657 | 9.471946688 | 5.79E-20 | 2.96E-19 | 33.84229014 |
| TMEM200A | 1.460190163 | 4.228569771 | 9.465342952 | 6.12E-20 | 3.13E-19 | 33.7876809 |
| JPH2 | 1.137568769 | 2.714456793 | 9.461507953 | 6.32E-20 | 3.23E-19 | 33.75598062 |
| FSTL3 | 1.788555974 | 5.841636466 | 9.439000946 | 7.63E-20 | 3.88E-19 | 33.5701299 |
| ZP3 | 1.302529568 | 3.182676545 | 9.437741249 | 7.71E-20 | 3.92E-19 | 33.55973777 |
| RCN3 | 1.334006983 | 4.764285384 | 9.435673932 | 7.84E-20 | 3.99E-19 | 33.54268525 |
| C6orf132 | -1.395385987 | 2.549428614 | -9.428638059 | 8.32E-20 | 4.22E-19 | 33.48466992 |
| LDLR | -1.219631354 | 4.035651375 | -9.419087898 | 9.00E-20 | 4.57E-19 | 33.40597441 |
| F3 | -1.543021779 | 2.91667932 | -9.402966317 | 1.03E-19 | 5.21E-19 | 33.27326426 |
| LCK | 1.228542397 | 4.788790963 | 9.402679501 | 1.03E-19 | 5.22E-19 | 33.27090479 |
| CD69 | 1.14657021 | 4.693700692 | 9.396381297 | 1.09E-19 | 5.49E-19 | 33.21910644 |
| RRAD | 1.535119105 | 5.786995895 | 9.369684281 | 1.36E-19 | 6.83E-19 | 32.99983088 |
| CD44 | 1.259365442 | 4.890384027 | 9.36646628 | 1.39E-19 | 7.01E-19 | 32.97343146 |
| NTN1 | -1.222943359 | 1.759787228 | -9.357141671 | 1.51E-19 | 7.56E-19 | 32.8969739 |
| CLK1 | 1.014849498 | 6.337922998 | 9.354858831 | 1.53E-19 | 7.70E-19 | 32.87826436 |
| SIGLEC14 | 1.172985926 | 2.973436573 | 9.354154499 | 1.54E-19 | 7.74E-19 | 32.87249254 |
| CRNDE | 1.005607585 | 3.285984567 | 9.347600906 | 1.63E-19 | 8.17E-19 | 32.81880315 |
| CYP1B1 | -1.656676624 | 5.017434532 | -9.345040505 | 1.66E-19 | 8.34E-19 | 32.7978351 |
| NRP1 | 1.196494039 | 6.592434147 | 9.332095626 | 1.85E-19 | 9.27E-19 | 32.69189091 |
| PCDHB14 | 1.004682968 | 3.811910271 | 9.323909405 | 1.98E-19 | 9.89E-19 | 32.62494978 |
| IRS1 | -1.168649372 | 2.9085876 | -9.315173707 | 2.13E-19 | 1.06E-18 | 32.55356418 |
| S100A8 | 1.350770056 | 4.214116253 | 9.303729044 | 2.34E-19 | 1.17E-18 | 32.46011792 |
| RNF182 | -1.017211171 | 1.420447682 | -9.302419504 | 2.37E-19 | 1.18E-18 | 32.44943098 |
| ARL4C | 1.506681567 | 6.620763829 | 9.301736857 | 2.38E-19 | 1.18E-18 | 32.44386045 |
| IL7R | 1.415942977 | 4.406897291 | 9.288800216 | 2.65E-19 | 1.31E-18 | 32.33835337 |
| RIC3 | -1.120814295 | 2.253261424 | -9.288581419 | 2.65E-19 | 1.32E-18 | 32.33656988 |
| SORCS2 | -1.761549349 | 3.926701491 | -9.281586757 | 2.81E-19 | 1.39E-18 | 32.27957079 |
| PCDH1 | -1.084502083 | 6.307354635 | -9.281343027 | 2.82E-19 | 1.40E-18 | 32.27758524 |
| WDR54 | 1.061475357 | 5.181491762 | 9.268890306 | 3.12E-19 | 1.54E-18 | 32.17619094 |
| DOC2B | -1.040538415 | 4.284878625 | -9.267036709 | 3.17E-19 | 1.57E-18 | 32.16110711 |
| QPRT | -1.4439165 | 5.153124981 | -9.25992316 | 3.36E-19 | 1.66E-18 | 32.10324102 |
| MASP1 | 1.469568215 | 4.042174614 | 9.253683766 | 3.54E-19 | 1.74E-18 | 32.05251349 |
| LY6E | 1.580722295 | 7.193364447 | 9.251548579 | 3.60E-19 | 1.77E-18 | 32.03515993 |
| GPT2 | -1.036953909 | 3.26527028 | -9.243803406 | 3.84E-19 | 1.88E-18 | 31.97223704 |
| ALDH1B1 | -1.207595541 | 4.901148045 | -9.239822239 | 3.96E-19 | 1.95E-18 | 31.93990897 |
| CEACAM1 | -1.105954792 | 4.302940163 | -9.228519911 | 4.35E-19 | 2.13E-18 | 31.84818867 |
| CHD3 | -1.232845462 | 5.174710897 | -9.221493186 | 4.61E-19 | 2.25E-18 | 31.79120844 |
| RASL12 | 1.153956867 | 4.988061511 | 9.212471983 | 4.96E-19 | 2.42E-18 | 31.71810302 |
| PROM1 | -2.507985996 | 4.542271934 | -9.210967315 | 5.02E-19 | 2.45E-18 | 31.70591486 |
| CA4 | -1.962457103 | 3.475567111 | -9.195448867 | 5.70E-19 | 2.77E-18 | 31.5802999 |
| CPN2 | -1.881501078 | 1.523839661 | -9.194057488 | 5.77E-19 | 2.80E-18 | 31.56904515 |
| TOX | -1.266736522 | 2.511549865 | -9.190320589 | 5.94E-19 | 2.89E-18 | 31.53882407 |
| TST | -1.003680631 | 5.892688819 | -9.182310743 | 6.35E-19 | 3.08E-18 | 31.47407818 |
| ASAP2 | -1.108394881 | 4.201715228 | -9.179113214 | 6.51E-19 | 3.16E-18 | 31.4482436 |
| SCG2 | 1.603259171 | 2.93013755 | 9.177757498 | 6.59E-19 | 3.19E-18 | 31.43729209 |
| TLR3 | 1.35857136 | 5.570215865 | 9.17594429 | 6.68E-19 | 3.24E-18 | 31.42264687 |
| SNX19 | -1.044027215 | 4.51337286 | -9.170714856 | 6.98E-19 | 3.38E-18 | 31.3804212 |
| APOC3 | -1.43879783 | 2.274505929 | -9.169708728 | 7.03E-19 | 3.40E-18 | 31.37229921 |
| KCNAB1 | 1.052716455 | 3.699122144 | 9.16965863 | 7.04E-19 | 3.40E-18 | 31.37189481 |
| MYO5B | -1.078650261 | 4.640132686 | -9.16888769 | 7.08E-19 | 3.42E-18 | 31.36567185 |
| AHCYL2 | -1.08903373 | 4.461625027 | -9.161475372 | 7.52E-19 | 3.63E-18 | 31.30586063 |
| IL20RB | 2.282334945 | 3.264732766 | 9.156268681 | 7.85E-19 | 3.79E-18 | 31.26386896 |
| MAMDC4 | 1.140285681 | 3.959580752 | 9.148207811 | 8.38E-19 | 4.04E-18 | 31.19889428 |
| APOL1 | 1.473881756 | 7.590253671 | 9.148005761 | 8.40E-19 | 4.04E-18 | 31.19726621 |
| SLC16A11 | -1.043490781 | 2.628471877 | -9.145108776 | 8.60E-19 | 4.14E-18 | 31.17392607 |
| DPY19L2P2 | 1.034318642 | 3.577821728 | 9.144100528 | 8.67E-19 | 4.17E-18 | 31.16580424 |
| MALL | 1.174055132 | 3.754214743 | 9.142819299 | 8.76E-19 | 4.21E-18 | 31.15548442 |
| SSPN | 1.104594879 | 4.888160939 | 9.140465685 | 8.93E-19 | 4.29E-18 | 31.13652981 |
| WFDC2 | -2.044133909 | 5.750360353 | -9.13053614 | 9.68E-19 | 4.65E-18 | 31.05660403 |
| LGR4 | -1.054288617 | 5.057199249 | -9.128672324 | 9.83E-19 | 4.71E-18 | 31.04160901 |
| CD163 | 1.622223451 | 5.16934284 | 9.118850395 | 1.06E-18 | 5.09E-18 | 30.96262682 |
| PROS1 | 1.355174898 | 6.649540672 | 9.116549436 | 1.08E-18 | 5.18E-18 | 30.94413322 |
| SPON2 | 1.545150367 | 6.117435326 | 9.110618703 | 1.14E-18 | 5.43E-18 | 30.89648227 |
| BTNL9 | 1.586132545 | 5.239044756 | 9.106229428 | 1.18E-18 | 5.63E-18 | 30.86123152 |
| SPARCL1 | 1.423183737 | 9.159422897 | 9.104365926 | 1.20E-18 | 5.71E-18 | 30.84626945 |
| GGH | -1.00978448 | 3.748530263 | -9.099180183 | 1.25E-18 | 5.95E-18 | 30.80464535 |
| NPY1R | -1.338213396 | 4.01585919 | -9.086325945 | 1.39E-18 | 6.59E-18 | 30.70154702 |
| MEST | -1.487986077 | 3.005289693 | -9.068931793 | 1.60E-18 | 7.55E-18 | 30.56221315 |
| PABPC1L | 1.395637327 | 3.535613912 | 9.068253016 | 1.60E-18 | 7.59E-18 | 30.55678003 |
| LRRC17 | 1.093241648 | 3.870003369 | 9.062830098 | 1.68E-18 | 7.92E-18 | 30.51338455 |
| CNTFR | -1.04013291 | 1.156306113 | -9.061785575 | 1.69E-18 | 7.99E-18 | 30.50502831 |
| BHLHA15 | 1.097940662 | 3.102715998 | 9.059556036 | 1.72E-18 | 8.13E-18 | 30.48719434 |
| FOXJ3 | 1.25370526 | 5.697365186 | 9.052052311 | 1.83E-18 | 8.63E-18 | 30.42719708 |
| PC | -1.088660861 | 4.673502657 | -9.051255974 | 1.84E-18 | 8.68E-18 | 30.42083207 |
| OSTC | 1.025076129 | 7.040196907 | 9.050579016 | 1.85E-18 | 8.72E-18 | 30.41542158 |
| MARVELD1 | 1.047114489 | 5.137046292 | 9.04571787 | 1.93E-18 | 9.07E-18 | 30.37657862 |
| EPHA3 | 1.200305731 | 3.832405136 | 9.043156382 | 1.97E-18 | 9.25E-18 | 30.35611749 |
| FOLR2 | 1.348034035 | 5.151727602 | 9.040136756 | 2.01E-18 | 9.47E-18 | 30.33200245 |
| CCL20 | 2.114727535 | 4.222955345 | 9.036632173 | 2.07E-18 | 9.73E-18 | 30.30402223 |
| CD82 | -1.125852222 | 4.621679863 | -9.024209065 | 2.29E-18 | 1.07E-17 | 30.20490439 |
| REC8 | -1.060518481 | 2.631317942 | -9.022928698 | 2.31E-18 | 1.08E-17 | 30.19469491 |
| KCNN1 | 1.226532895 | 1.533971353 | 9.017317668 | 2.42E-18 | 1.13E-17 | 30.14996638 |
| UGT3A1 | -2.084542289 | 4.68560474 | -9.01512569 | 2.46E-18 | 1.15E-17 | 30.13249874 |
| MATN2 | -1.173024964 | 4.735922266 | -9.014255305 | 2.48E-18 | 1.16E-17 | 30.12556363 |
| ANGPTL2 | 1.318782671 | 6.452933103 | 9.013938986 | 2.49E-18 | 1.16E-17 | 30.12304338 |
| VCAN | 1.736049462 | 4.88037283 | 9.013789501 | 2.49E-18 | 1.16E-17 | 30.12185238 |
| FBLN7 | 1.114543653 | 2.677298086 | 8.998507121 | 2.82E-18 | 1.31E-17 | 30.00017308 |
| COL21A1 | 1.324566605 | 2.916991781 | 8.997101047 | 2.85E-18 | 1.33E-17 | 29.98898578 |
| DACT2 | -1.19039433 | 2.422170021 | -8.985449023 | 3.13E-18 | 1.45E-17 | 29.89632918 |
| CEBPA | 1.082866879 | 3.214257603 | 8.975441112 | 3.39E-18 | 1.57E-17 | 29.81682015 |
| TMEM92 | 1.655142871 | 2.784121093 | 8.97382119 | 3.44E-18 | 1.59E-17 | 29.80395689 |
| PCDH7 | -1.42145479 | 1.762847312 | -8.967099379 | 3.63E-18 | 1.68E-17 | 29.75060029 |
| ZNF204P | -1.26442527 | 4.246479008 | -8.957432227 | 3.92E-18 | 1.81E-17 | 29.67391797 |
| SPINK13 | 1.858937754 | 3.02199794 | 8.952028845 | 4.09E-18 | 1.89E-17 | 29.63108468 |
| FOXO4 | -1.056793956 | 5.009702934 | -8.946435605 | 4.28E-18 | 1.97E-17 | 29.58676732 |
| RDH12 | -1.121213013 | 2.888638508 | -8.931832904 | 4.81E-18 | 2.21E-17 | 29.4711651 |
| FPR1 | 1.341004143 | 3.451744747 | 8.931613737 | 4.82E-18 | 2.22E-17 | 29.46943118 |
| TLN2 | -1.110235713 | 3.810523357 | -8.929367538 | 4.91E-18 | 2.26E-17 | 29.45166239 |
| BCL6 | 1.07902419 | 4.817282896 | 8.924726312 | 5.09E-18 | 2.34E-17 | 29.41495842 |
| MUC13 | -1.44307238 | 1.603624625 | -8.909358695 | 5.76E-18 | 2.64E-17 | 29.29353247 |
| VSIG4 | 1.408042656 | 5.407878765 | 8.901842949 | 6.11E-18 | 2.80E-17 | 29.23420621 |
| CCDC85B | 1.158744912 | 5.075387993 | 8.898051207 | 6.30E-18 | 2.88E-17 | 29.2042904 |
| SLC37A4 | 1.116929407 | 5.8385031 | 8.897176657 | 6.35E-18 | 2.90E-17 | 29.19739183 |
| GSDMB | 1.033746955 | 3.487181566 | 8.893740032 | 6.52E-18 | 2.98E-17 | 29.17028835 |
| COL5A1 | 1.524063639 | 5.188872343 | 8.888592775 | 6.80E-18 | 3.10E-17 | 29.12970882 |
| HTR6 | 1.568916616 | 2.049011644 | 8.88561879 | 6.96E-18 | 3.17E-17 | 29.10627103 |
| PDZRN3 | -1.14364889 | 2.960806477 | -8.8845612 | 7.02E-18 | 3.20E-17 | 29.09793769 |
| GPR155 | -1.237241433 | 4.136134037 | -8.881810716 | 7.17E-18 | 3.27E-17 | 29.07626868 |
| TDRD9 | -1.125009643 | 1.87815155 | -8.873535404 | 7.66E-18 | 3.49E-17 | 29.01110496 |
| CCNO | -1.073588543 | 2.308311801 | -8.871738218 | 7.77E-18 | 3.54E-17 | 28.99695927 |
| RELN | -1.062899842 | 1.916794225 | -8.86978115 | 7.90E-18 | 3.59E-17 | 28.98155766 |
| LAYN | 1.045309713 | 4.920671997 | 8.868015467 | 8.01E-18 | 3.64E-17 | 28.96766446 |
| HECW2 | 1.25137552 | 3.072862442 | 8.860257349 | 8.52E-18 | 3.87E-17 | 28.90664539 |
| FAM83F | -1.043050525 | 2.124706974 | -8.856688618 | 8.76E-18 | 3.97E-17 | 28.87859052 |
| CLDN7 | -1.369438758 | 5.727643088 | -8.855346901 | 8.86E-18 | 4.01E-17 | 28.86804513 |
| IFI44L | 1.229765672 | 4.823651968 | 8.855329879 | 8.86E-18 | 4.01E-17 | 28.86791136 |
| SERPINE1 | 1.8484797 | 8.52171437 | 8.828360763 | 1.10E-17 | 4.95E-17 | 28.65620715 |
| CRYL1 | -1.004602508 | 6.042595437 | -8.828111496 | 1.10E-17 | 4.96E-17 | 28.65425277 |
| RARRES2 | 1.325841464 | 8.949702508 | 8.816623466 | 1.20E-17 | 5.42E-17 | 28.56422712 |
| CXCL12 | -1.373304734 | 6.288049442 | -8.803737228 | 1.33E-17 | 5.99E-17 | 28.46335267 |
| WWC1 | -1.01022635 | 5.25442941 | -8.803260653 | 1.34E-17 | 6.01E-17 | 28.4596242 |
| CHDH | -1.1844951 | 4.659664569 | -8.793977589 | 1.44E-17 | 6.46E-17 | 28.38702968 |
| CYP24A1 | -1.826751237 | 2.058604188 | -8.791856959 | 1.47E-17 | 6.56E-17 | 28.37045449 |
| SPHK1 | 1.032350706 | 2.910745391 | 8.787390441 | 1.52E-17 | 6.80E-17 | 28.33555359 |
| SELE | -1.140989445 | 2.942598407 | -8.786539725 | 1.53E-17 | 6.84E-17 | 28.32890775 |
| SCTR | -1.081893185 | 2.768112975 | -8.772497121 | 1.71E-17 | 7.62E-17 | 28.21927839 |
| CCR2 | 1.116066286 | 3.59718748 | 8.753375509 | 1.99E-17 | 8.84E-17 | 28.07021698 |
| EXPH5 | -1.110934916 | 2.314174111 | -8.750532536 | 2.03E-17 | 9.03E-17 | 28.04807636 |
| VWA1 | 1.126394865 | 6.649810242 | 8.729696728 | 2.39E-17 | 1.06E-16 | 27.88598146 |
| APOH | -1.633422648 | 1.763930267 | -8.721711969 | 2.55E-17 | 1.13E-16 | 27.8239428 |
| ASMTL-AS1 | 1.320407051 | 3.313831914 | 8.720768757 | 2.57E-17 | 1.13E-16 | 27.81661732 |
| THOC1 | 1.128442935 | 3.189975492 | 8.714943169 | 2.69E-17 | 1.19E-16 | 27.77138639 |
| GIMAP7 | 1.283954899 | 6.910261707 | 8.714373074 | 2.70E-17 | 1.19E-16 | 27.76696133 |
| HLA-DRB6 | 1.421500948 | 5.715081949 | 8.714217665 | 2.70E-17 | 1.19E-16 | 27.76575509 |
| CDHR1 | 1.72514835 | 2.98139822 | 8.713498495 | 2.72E-17 | 1.20E-16 | 27.76017332 |
| GOLGA8A | 1.550983644 | 3.572543871 | 8.712663001 | 2.74E-17 | 1.20E-16 | 27.75368917 |
| PREX2 | 1.337522095 | 4.416095388 | 8.701688645 | 2.98E-17 | 1.31E-16 | 27.66856372 |
| LENG8 | 1.212572999 | 6.065623148 | 8.696399521 | 3.11E-17 | 1.37E-16 | 27.62756717 |
| POSTN | 1.749645791 | 5.106955772 | 8.684142435 | 3.42E-17 | 1.50E-16 | 27.53263607 |
| CENPW | 1.076411511 | 3.486546984 | 8.682057176 | 3.48E-17 | 1.52E-16 | 27.51649616 |
| FZD1 | 1.111437113 | 6.144854773 | 8.66947366 | 3.84E-17 | 1.68E-16 | 27.41916403 |
| GLB1L | 1.06587431 | 5.922037982 | 8.665546825 | 3.96E-17 | 1.73E-16 | 27.388813 |
| GUCA2B | 1.721898199 | 2.872389878 | 8.663618943 | 4.02E-17 | 1.76E-16 | 27.37391607 |
| COL15A1 | 1.488988147 | 6.459461177 | 8.660611736 | 4.11E-17 | 1.80E-16 | 27.35068429 |
| CKB | -1.363901853 | 6.648452286 | -8.657167738 | 4.23E-17 | 1.85E-16 | 27.32408588 |
| ANXA2 | 1.016672868 | 7.00626891 | 8.648060616 | 4.54E-17 | 1.98E-16 | 27.25379041 |
| CX3CR1 | 1.426669235 | 5.07786289 | 8.647440126 | 4.56E-17 | 1.99E-16 | 27.24900312 |
| PARM1 | -1.125320625 | 5.581693061 | -8.629393152 | 5.25E-17 | 2.28E-16 | 27.10988236 |
| NCOA7 | -1.036460712 | 4.930454547 | -8.62181139 | 5.57E-17 | 2.41E-16 | 27.05150393 |
| TCF19 | 1.057968107 | 4.101611048 | 8.614603572 | 5.89E-17 | 2.55E-16 | 26.99604216 |
| ABCB4 | 1.02212169 | 1.897753692 | 8.607708801 | 6.22E-17 | 2.69E-16 | 26.94302322 |
| SPINK1 | -1.956542181 | 3.290273425 | -8.605045733 | 6.35E-17 | 2.74E-16 | 26.92255387 |
| TGM2 | 1.248270258 | 7.614369391 | 8.603127261 | 6.44E-17 | 2.78E-16 | 26.90781084 |
| SPRY1 | 1.231886977 | 6.637929166 | 8.598700002 | 6.67E-17 | 2.88E-16 | 26.87379821 |
| SLIT2 | -1.214150105 | 2.214196388 | -8.597130268 | 6.75E-17 | 2.91E-16 | 26.86174195 |
| IFI27L2 | 1.235513256 | 5.91539389 | 8.58670901 | 7.32E-17 | 3.15E-16 | 26.7817459 |
| CLRN3 | 1.915875367 | 5.528484349 | 8.579201328 | 7.76E-17 | 3.33E-16 | 26.72416241 |
| ATP9A | -1.073321728 | 5.255568995 | -8.576979425 | 7.89E-17 | 3.38E-16 | 26.70712813 |
| CSAD | 1.021458767 | 3.766602944 | 8.561150509 | 8.92E-17 | 3.81E-16 | 26.58587579 |
| SPRY4 | 1.099364192 | 5.875901288 | 8.561128989 | 8.93E-17 | 3.81E-16 | 26.58571106 |
| FLRT3 | -1.701697183 | 4.513930095 | -8.547735094 | 9.90E-17 | 4.22E-16 | 26.48324933 |
| VCAM1 | 1.607628422 | 6.906572764 | 8.536799018 | 1.08E-16 | 4.58E-16 | 26.39968334 |
| PKHD1 | -1.431911919 | 4.39894597 | -8.53410174 | 1.10E-16 | 4.68E-16 | 26.37908555 |
| RAB11FIP1 | -1.02127028 | 4.473268645 | -8.527835119 | 1.15E-16 | 4.91E-16 | 26.33125026 |
| AQP7 | -1.113684621 | 3.66373829 | -8.525003063 | 1.18E-16 | 5.01E-16 | 26.30964129 |
| SCRN1 | -1.157286491 | 6.385326587 | -8.523392583 | 1.20E-16 | 5.07E-16 | 26.29735562 |
| SMTNL2 | -1.402541842 | 3.942655548 | -8.52286524 | 1.20E-16 | 5.09E-16 | 26.29333316 |
| EVC | -1.174666031 | 4.488208353 | -8.522594039 | 1.20E-16 | 5.10E-16 | 26.29126456 |
| IFNG | 1.025518093 | 2.856037992 | 8.522539151 | 1.20E-16 | 5.10E-16 | 26.2908459 |
| C8orf58 | 1.035911501 | 4.23758137 | 8.518787952 | 1.24E-16 | 5.24E-16 | 26.26223898 |
| CCDC8 | -1.617042082 | 4.3265362 | -8.48611638 | 1.59E-16 | 6.70E-16 | 26.0135036 |
| IRX3 | 1.395910277 | 5.35149852 | 8.482927841 | 1.63E-16 | 6.86E-16 | 25.98926905 |
| B3GNT4 | 1.103595989 | 2.103372843 | 8.480710027 | 1.66E-16 | 6.97E-16 | 25.97241675 |
| TSPYL2 | 1.007687426 | 4.273348057 | 8.476968616 | 1.71E-16 | 7.17E-16 | 25.94399514 |
| VNN2 | 1.085085785 | 3.975034505 | 8.465861294 | 1.86E-16 | 7.80E-16 | 25.85967685 |
| C10orf99 | 2.495446847 | 3.101672869 | 8.465266861 | 1.87E-16 | 7.83E-16 | 25.85516684 |
| DNAJB13 | 1.013811046 | 1.99020021 | 8.459790252 | 1.95E-16 | 8.16E-16 | 25.81362713 |
| SELP | -1.223236205 | 4.066288766 | -8.452642442 | 2.06E-16 | 8.61E-16 | 25.75944347 |
| CDC37L1 | -1.179568305 | 4.353531888 | -8.448345958 | 2.13E-16 | 8.89E-16 | 25.72689162 |
| MYL3 | -1.270836617 | 2.536646806 | -8.436049049 | 2.34E-16 | 9.75E-16 | 25.63379787 |
| LUM | -2.266321874 | 5.184434437 | -8.431990242 | 2.41E-16 | 1.00E-15 | 25.60309427 |
| PNPLA7 | 1.044773918 | 3.354645852 | 8.426560609 | 2.52E-16 | 1.05E-15 | 25.56203911 |
| P2RY12 | 1.022947505 | 2.381134865 | 8.415746332 | 2.73E-16 | 1.13E-15 | 25.48033146 |
| SHANK3 | 1.056091109 | 5.484571896 | 8.414096048 | 2.77E-16 | 1.15E-15 | 25.46787 |
| CFD | 1.220200031 | 5.196581439 | 8.399653184 | 3.09E-16 | 1.28E-15 | 25.35889329 |
| TMEM200B | 1.00996084 | 4.122103031 | 8.383228606 | 3.51E-16 | 1.45E-15 | 25.23514456 |
| FTH1 | 1.07525533 | 6.9860626 | 8.377679527 | 3.66E-16 | 1.51E-15 | 25.1933793 |
| SDC1 | -1.184424276 | 6.499666714 | -8.366858133 | 3.97E-16 | 1.63E-15 | 25.1119951 |
| LGALS12 | 1.513304311 | 1.939092431 | 8.355855855 | 4.32E-16 | 1.77E-15 | 25.02933634 |
| ITPKC | 1.181533423 | 5.269447555 | 8.353673238 | 4.39E-16 | 1.80E-15 | 25.01294891 |
| PILRB | 1.00140191 | 1.9845684 | 8.352684583 | 4.42E-16 | 1.81E-15 | 25.00552705 |
| IL2RA | 1.0304471 | 2.848416263 | 8.350372077 | 4.50E-16 | 1.85E-15 | 24.98816974 |
| APLNR | 1.527575756 | 5.816311729 | 8.347469103 | 4.60E-16 | 1.89E-15 | 24.96638589 |
| RNF152 | -1.13030525 | 4.288156879 | -8.346640292 | 4.63E-16 | 1.90E-15 | 24.96016761 |
| ALOX5AP | 1.062734636 | 4.816323228 | 8.34433364 | 4.71E-16 | 1.93E-15 | 24.94286422 |
| TSPAN1 | -1.841657094 | 7.196816095 | -8.319471719 | 5.69E-16 | 2.32E-15 | 24.756604 |
| ARC | -1.008679797 | 2.679858501 | -8.314747581 | 5.90E-16 | 2.40E-15 | 24.72126188 |
| SORL1 | -1.21640181 | 4.877627314 | -8.282779298 | 7.52E-16 | 3.04E-15 | 24.4825227 |
| NRXN2 | 1.372751439 | 3.350559637 | 8.271655208 | 8.18E-16 | 3.30E-15 | 24.39962035 |
| SLC4A3 | -1.106880586 | 2.075806659 | -8.265112818 | 8.59E-16 | 3.46E-15 | 24.35090477 |
| HAVCR1 | 2.025129534 | 5.776582316 | 8.251429766 | 9.52E-16 | 3.84E-15 | 24.2491185 |
| CD38 | 1.025811648 | 2.207938127 | 8.249803585 | 9.64E-16 | 3.88E-15 | 24.23703056 |
| DDAH1 | -1.064054012 | 7.209996133 | -8.247226512 | 9.83E-16 | 3.96E-15 | 24.21787821 |
| CAPN6 | -1.93534794 | 3.693235268 | -8.239333882 | 1.04E-15 | 4.19E-15 | 24.15925142 |
| ITPKB | -1.000886302 | 5.193537585 | -8.232273309 | 1.10E-15 | 4.41E-15 | 24.1068433 |
| ADM2 | 1.306488713 | 4.560693268 | 8.210330382 | 1.30E-15 | 5.19E-15 | 23.94419873 |
| GDF15 | -1.227047482 | 6.446921019 | -8.191282765 | 1.50E-15 | 5.97E-15 | 23.80329716 |
| UPK1B | -1.577403286 | 2.765588906 | -8.186689628 | 1.55E-15 | 6.18E-15 | 23.76935953 |
| SPSB1 | 1.071074947 | 5.632059869 | 8.182727553 | 1.60E-15 | 6.36E-15 | 23.74009696 |
| KL | -1.58019037 | 5.917746399 | -8.17964651 | 1.63E-15 | 6.50E-15 | 23.71734927 |
| SLC12A7 | 1.047044145 | 6.922342457 | 8.172260116 | 1.73E-15 | 6.86E-15 | 23.66284272 |
| CLIC3 | 1.071831336 | 2.365846228 | 8.161264635 | 1.87E-15 | 7.43E-15 | 23.58177694 |
| PKIA | -1.312255737 | 2.523775842 | -8.160478337 | 1.88E-15 | 7.47E-15 | 23.57598321 |
| GJA4 | 1.180719949 | 6.425906786 | 8.160229945 | 1.89E-15 | 7.48E-15 | 23.57415306 |
| BEX2 | -1.384974532 | 5.094699302 | -8.148456691 | 2.06E-15 | 8.14E-15 | 23.48745924 |
| GPR35 | 1.061726901 | 3.375679382 | 8.136436565 | 2.25E-15 | 8.89E-15 | 23.39905155 |
| UNC5A | 1.058382726 | 1.430795852 | 8.132700834 | 2.32E-15 | 9.13E-15 | 23.37159677 |
| PBX4 | 1.047642676 | 2.42092274 | 8.123474046 | 2.48E-15 | 9.76E-15 | 23.30383044 |
| ATP11A | 1.212697101 | 6.168607873 | 8.114019897 | 2.66E-15 | 1.05E-14 | 23.23445858 |
| COL1A2 | 1.443918468 | 8.515721325 | 8.10705169 | 2.81E-15 | 1.10E-14 | 23.18336958 |
| RNF180 | -1.007957004 | 3.813947913 | -8.105042965 | 2.85E-15 | 1.12E-14 | 23.16864873 |
| SH2D3C | 1.010308159 | 4.563860722 | 8.100880582 | 2.94E-15 | 1.15E-14 | 23.13815426 |
| BEX1 | -1.881475589 | 3.161022085 | -8.100625071 | 2.94E-15 | 1.15E-14 | 23.13628274 |
| IL22RA1 | 1.134435421 | 2.971921961 | 8.079521777 | 3.44E-15 | 1.34E-14 | 22.98187431 |
| PECAM1 | 1.066428093 | 8.353309202 | 8.072274653 | 3.63E-15 | 1.41E-14 | 22.92892369 |
| EDNRA | 1.041569702 | 5.041564204 | 8.045549094 | 4.43E-15 | 1.71E-14 | 22.73398738 |
| CIB4 | 1.198484303 | 2.164833197 | 8.043679388 | 4.49E-15 | 1.73E-14 | 22.72036932 |
| ITPKA | 1.098277609 | 2.087044404 | 8.026255009 | 5.10E-15 | 1.97E-14 | 22.59358161 |
| TNFRSF11B | -1.316672601 | 6.167568254 | -8.021954745 | 5.27E-15 | 2.03E-14 | 22.5623252 |
| PTGES | -1.394922098 | 3.006297792 | -8.018487755 | 5.41E-15 | 2.08E-14 | 22.53713533 |
| DSCAML1 | 1.002832386 | 3.811409857 | 8.007790845 | 5.85E-15 | 2.24E-14 | 22.45947116 |
| RPL23AP64 | 1.019890476 | 3.5007601 | 8.003864817 | 6.02E-15 | 2.31E-14 | 22.43098759 |
| FHL1 | 1.332520467 | 6.874834225 | 8.00376368 | 6.02E-15 | 2.31E-14 | 22.43025399 |
| CSDC2 | -1.820666031 | 3.55119082 | -7.999113005 | 6.23E-15 | 2.39E-14 | 22.39652809 |
| TIMD4 | 1.113517887 | 4.582840527 | 7.987277274 | 6.80E-15 | 2.60E-14 | 22.31076917 |
| TTR | -1.086176094 | 1.560944849 | -7.962142121 | 8.18E-15 | 3.11E-14 | 22.12898794 |
| ETS1 | 1.043509583 | 7.54857681 | 7.913344785 | 1.17E-14 | 4.40E-14 | 21.77740961 |
| NAPSA | -1.593276026 | 4.124882076 | -7.910654918 | 1.19E-14 | 4.49E-14 | 21.75808069 |
| KCNJ15 | -1.459764705 | 5.841734534 | -7.896582515 | 1.32E-14 | 4.96E-14 | 21.65704615 |
| LY6H | 1.112072813 | 1.665408014 | 7.889920954 | 1.39E-14 | 5.20E-14 | 21.60926984 |
| PODXL2 | -1.217066969 | 4.077395597 | -7.872087633 | 1.58E-14 | 5.90E-14 | 21.48153239 |
| VANGL2 | -1.100504609 | 2.688503501 | -7.868681103 | 1.62E-14 | 6.04E-14 | 21.45715878 |
| GULP1 | -1.045652019 | 3.491066301 | -7.850307182 | 1.85E-14 | 6.88E-14 | 21.32584265 |
| KLHDC7A | -1.112028489 | 5.609544927 | -7.848311443 | 1.87E-14 | 6.97E-14 | 21.31159446 |
| FOXS1 | 1.096994669 | 4.081968551 | 7.846251828 | 1.90E-14 | 7.08E-14 | 21.29689336 |
| OSM | 1.034670504 | 3.068012055 | 7.83657016 | 2.04E-14 | 7.58E-14 | 21.2278299 |
| FGF7 | -1.059749583 | 2.945722568 | -7.810161839 | 2.47E-14 | 9.14E-14 | 21.03980312 |
| TRIM50 | -1.125653859 | 1.002855567 | -7.799600016 | 2.67E-14 | 9.85E-14 | 20.96474873 |
| MEGF6 | 1.001186175 | 3.114595061 | 7.794610168 | 2.76E-14 | 1.02E-13 | 20.92931887 |
| ENPEP | 1.478723502 | 6.814868453 | 7.787698559 | 2.90E-14 | 1.07E-13 | 20.88027446 |
| DNAJC22 | 1.080279597 | 5.258449692 | 7.786725672 | 2.93E-14 | 1.08E-13 | 20.87337377 |
| HLA-DRB5 | 1.203966517 | 8.226240358 | 7.768189518 | 3.34E-14 | 1.23E-13 | 20.74203202 |
| HERC2P2 | 1.079141987 | 3.558816119 | 7.752980722 | 3.73E-14 | 1.37E-13 | 20.634459 |
| PADI1 | 1.399433355 | 2.884053355 | 7.744616151 | 3.96E-14 | 1.45E-13 | 20.57536958 |
| EDN1 | 1.415888545 | 6.921321797 | 7.71044365 | 5.06E-14 | 1.84E-13 | 20.33451175 |
| TUBA3D | 1.853076249 | 3.796928437 | 7.700625338 | 5.42E-14 | 1.97E-13 | 20.26547162 |
| PGBD5 | 1.507419965 | 2.892665174 | 7.683235113 | 6.14E-14 | 2.22E-13 | 20.14336551 |
| RHOBTB1 | 1.196866137 | 5.662225631 | 7.670689939 | 6.71E-14 | 2.43E-13 | 20.0554205 |
| FCN3 | 1.305768882 | 5.261199285 | 7.66274882 | 7.10E-14 | 2.56E-13 | 19.99981246 |
| CXADR | -1.051501096 | 4.599816952 | -7.658081656 | 7.34E-14 | 2.65E-13 | 19.96715262 |
| BEX5 | -1.122846143 | 4.970771865 | -7.640482796 | 8.32E-14 | 2.99E-13 | 19.84414735 |
| SCGB1D2 | -1.334760557 | 2.61283316 | -7.618987927 | 9.69E-14 | 3.47E-13 | 19.69422882 |
| CDH5 | 1.040929406 | 5.572219532 | 7.617745265 | 9.78E-14 | 3.50E-13 | 19.6855724 |
| BCL6B | 1.134802235 | 4.580377546 | 7.601369329 | 1.10E-13 | 3.92E-13 | 19.57160649 |
| SYNPO | 1.347438131 | 6.782752716 | 7.59028764 | 1.19E-13 | 4.22E-13 | 19.49460035 |
| TRIM47 | 1.440428967 | 5.414021099 | 7.580249423 | 1.27E-13 | 4.52E-13 | 19.42492562 |
| TBC1D14 | -1.118186409 | 5.067846641 | -7.573100851 | 1.34E-13 | 4.75E-13 | 19.37535437 |
| TGFA | 1.267496807 | 6.973867441 | 7.569157775 | 1.38E-13 | 4.88E-13 | 19.348028 |
| NOTCH3 | 1.082883695 | 6.448222746 | 7.551117184 | 1.56E-13 | 5.53E-13 | 19.22315345 |
| CES3 | 1.638682898 | 4.122511461 | 7.550098396 | 1.58E-13 | 5.56E-13 | 19.21610891 |
| GPC4 | -1.25375131 | 5.147763585 | -7.53461553 | 1.76E-13 | 6.19E-13 | 19.1091479 |
| METTL7B | 1.251072011 | 5.618306151 | 7.528433453 | 1.83E-13 | 6.45E-13 | 19.06649094 |
| CHI3L2 | 1.114671528 | 3.700054885 | 7.520066102 | 1.94E-13 | 6.82E-13 | 19.00880173 |
| ANK1 | 1.030481103 | 2.702018521 | 7.507747794 | 2.12E-13 | 7.43E-13 | 18.92396951 |
| OGN | -1.258238944 | 2.57432931 | -7.497656256 | 2.27E-13 | 7.95E-13 | 18.85455858 |
| CLEC14A | 1.104553343 | 6.827264684 | 7.495656925 | 2.31E-13 | 8.06E-13 | 18.84081614 |
| SLC22A6 | -2.388146031 | 3.438875347 | -7.49200012 | 2.37E-13 | 8.27E-13 | 18.81568891 |
| FGL2 | -1.03378726 | 5.803779506 | -7.488543571 | 2.42E-13 | 8.47E-13 | 18.79194711 |
| ADORA1 | -1.105983223 | 2.691670783 | -7.474569579 | 2.67E-13 | 9.31E-13 | 18.69605779 |
| SPNS2 | -1.014408062 | 5.944529306 | -7.441859441 | 3.36E-13 | 1.16E-12 | 18.47218533 |
| DMKN | -1.170164183 | 4.018470554 | -7.429437672 | 3.66E-13 | 1.26E-12 | 18.38738383 |
| EMCN | -1.229431432 | 5.76482437 | -7.424975347 | 3.77E-13 | 1.30E-12 | 18.35694911 |
| HLA-DQA2 | 1.886413533 | 6.148102323 | 7.413668388 | 4.08E-13 | 1.40E-12 | 18.27989986 |
| TCP11L1 | 1.241955734 | 3.572469557 | 7.401737464 | 4.43E-13 | 1.52E-12 | 18.19870516 |
| MAPK15 | 1.137556227 | 3.687070767 | 7.372079167 | 5.44E-13 | 1.85E-12 | 17.99734279 |
| COL14A1 | -1.249633828 | 4.404665802 | -7.364155058 | 5.74E-13 | 1.95E-12 | 17.94365738 |
| HSD11B1 | -1.151106916 | 2.565225514 | -7.356803661 | 6.04E-13 | 2.05E-12 | 17.89389531 |
| TPPP3 | 1.082269306 | 5.09690537 | 7.351192941 | 6.28E-13 | 2.13E-12 | 17.85594404 |
| STAC2 | -1.8272839 | 2.703889868 | -7.347555267 | 6.44E-13 | 2.18E-12 | 17.83135155 |
| MAB21L3 | -1.132156129 | 2.099999715 | -7.346657042 | 6.48E-13 | 2.19E-12 | 17.82528068 |
| FOLH1 | 1.206103032 | 4.57450679 | 7.331801772 | 7.18E-13 | 2.42E-12 | 17.72496794 |
| FOXQ1 | -1.052120949 | 4.816565086 | -7.314848589 | 8.06E-13 | 2.71E-12 | 17.61069693 |
| TMEM125 | -1.17313962 | 4.744395221 | -7.299578701 | 8.95E-13 | 3.00E-12 | 17.5079622 |
| EPHB3 | -1.044891108 | 2.645630522 | -7.287841428 | 9.70E-13 | 3.24E-12 | 17.42911733 |
| TRIM15 | 1.214831967 | 3.855773648 | 7.281926809 | 1.01E-12 | 3.37E-12 | 17.38942645 |
| SLC16A12 | -1.699067205 | 5.73131718 | -7.280874613 | 1.02E-12 | 3.39E-12 | 17.38236838 |
| MOGAT3 | 1.107501524 | 1.974701765 | 7.274813708 | 1.06E-12 | 3.53E-12 | 17.34172889 |
| ENTPD2 | 1.043914785 | 3.912155132 | 7.266872535 | 1.12E-12 | 3.72E-12 | 17.28852497 |
| CCR1 | 1.018159711 | 3.893281758 | 7.25684794 | 1.20E-12 | 3.97E-12 | 17.22143249 |
| PBLD | -1.216486059 | 6.006106368 | -7.252851182 | 1.23E-12 | 4.08E-12 | 17.19470479 |
| TNMD | -1.061824882 | 1.823274498 | -7.247904734 | 1.27E-12 | 4.21E-12 | 17.16164337 |
| ARHGAP5 | -1.025853932 | 3.545158147 | -7.245203076 | 1.30E-12 | 4.29E-12 | 17.14359385 |
| ST6GALNAC3 | -1.069980465 | 2.671194208 | -7.24228961 | 1.32E-12 | 4.37E-12 | 17.12413563 |
| PODXL | -1.14956479 | 7.583184792 | -7.230188802 | 1.44E-12 | 4.74E-12 | 17.04338834 |
| CLDN4 | -1.02583929 | 6.188497509 | -7.197683408 | 1.79E-12 | 5.87E-12 | 16.82704772 |
| GPM6A | -1.150018785 | 3.701373099 | -7.197076382 | 1.80E-12 | 5.89E-12 | 16.82301547 |
| CDH13 | 1.099427318 | 4.741018861 | 7.188925808 | 1.90E-12 | 6.22E-12 | 16.76890202 |
| PTGS2 | -1.130471802 | 3.721991805 | -7.185006621 | 1.95E-12 | 6.38E-12 | 16.74290013 |
| TMEM174 | -1.980089274 | 3.792903751 | -7.184835261 | 1.95E-12 | 6.39E-12 | 16.74176351 |
| FLT4 | 1.009552175 | 4.460824963 | 7.181984713 | 1.99E-12 | 6.51E-12 | 16.72285943 |
| THRSP | -1.101965576 | 1.677917759 | -7.177862118 | 2.05E-12 | 6.68E-12 | 16.69553067 |
| AQP9 | 1.324233755 | 3.447062107 | 7.175029784 | 2.09E-12 | 6.81E-12 | 16.67676277 |
| AKR7A3 | -1.318568707 | 5.371856805 | -7.154006608 | 2.40E-12 | 7.80E-12 | 16.53765272 |
| ACSM5 | 1.132927123 | 4.731452271 | 7.153635317 | 2.41E-12 | 7.82E-12 | 16.53519899 |
| CDH2 | 1.05272739 | 5.484105562 | 7.118287015 | 3.05E-12 | 9.85E-12 | 16.30208929 |
| BEST4 | 1.011037568 | 1.980950604 | 7.111413557 | 3.20E-12 | 1.03E-11 | 16.25687489 |
| CCL28 | 1.390629122 | 4.360328858 | 7.103962654 | 3.36E-12 | 1.08E-11 | 16.20790385 |
| GATM | -1.380226014 | 7.128657681 | -7.092547236 | 3.63E-12 | 1.16E-11 | 16.13296051 |
| PRND | 1.170095496 | 1.683510885 | 7.092171107 | 3.64E-12 | 1.17E-11 | 16.13049293 |
| MT1M | -1.324780834 | 4.32424549 | -7.072677497 | 4.14E-12 | 1.32E-11 | 16.00275782 |
| ENPP5 | -1.01926707 | 5.604393965 | -7.05601132 | 4.63E-12 | 1.48E-11 | 15.89378682 |
| TLL1 | 1.117373467 | 2.777233361 | 7.042975991 | 5.05E-12 | 1.60E-11 | 15.8087083 |
| MXRA5 | -1.191727207 | 4.326823933 | -7.042019571 | 5.08E-12 | 1.61E-11 | 15.80247124 |
| SNORA33 | 1.083969692 | 3.123104543 | 7.018771864 | 5.93E-12 | 1.87E-11 | 15.65108878 |
| MAOA | -1.096745434 | 6.177533005 | -7.014284285 | 6.11E-12 | 1.93E-11 | 15.62191603 |
| CHIT1 | 1.297180488 | 2.55012863 | 6.999391443 | 6.74E-12 | 2.12E-11 | 15.52521491 |
| PRELP | -1.394850615 | 3.9105585 | -6.97411036 | 7.96E-12 | 2.49E-11 | 15.36146292 |
| SULF1 | 1.091982685 | 5.278718884 | 6.926846242 | 1.09E-11 | 3.38E-11 | 15.05667872 |
| EMX2OS | -1.112839607 | 5.302847355 | -6.907158935 | 1.24E-11 | 3.82E-11 | 14.93024738 |
| HAO2 | -1.823214594 | 4.221546584 | -6.906846864 | 1.24E-11 | 3.83E-11 | 14.92824575 |
| CLEC18B | 1.356137839 | 4.823072105 | 6.899293109 | 1.30E-11 | 4.02E-11 | 14.87981938 |
| FOLR1 | -1.260222518 | 5.900593022 | -6.88506309 | 1.43E-11 | 4.40E-11 | 14.78871545 |
| DPYSL3 | 1.082676424 | 4.51373232 | 6.874931896 | 1.52E-11 | 4.70E-11 | 14.7239514 |
| COL3A1 | 1.224976346 | 7.493984268 | 6.854732101 | 1.74E-11 | 5.33E-11 | 14.59506752 |
| BAIAP2L2 | 1.355111773 | 4.704747625 | 6.838574316 | 1.93E-11 | 5.90E-11 | 14.49220778 |
| RAMP1 | -1.228232614 | 2.596938446 | -6.835469769 | 1.97E-11 | 6.01E-11 | 14.47246824 |
| MFAP4 | -1.617122931 | 4.941723343 | -6.826603629 | 2.09E-11 | 6.35E-11 | 14.41613734 |
| ZNF697 | -1.034755841 | 3.696152346 | -6.826207657 | 2.09E-11 | 6.37E-11 | 14.41362301 |
| ZNF385B | -1.116125606 | 2.569433737 | -6.816216258 | 2.23E-11 | 6.78E-11 | 14.35022125 |
| CRTAC1 | -1.315434447 | 2.119240952 | -6.802649132 | 2.44E-11 | 7.39E-11 | 14.26425705 |
| OLR1 | 1.065635315 | 4.768785636 | 6.772880806 | 2.95E-11 | 8.88E-11 | 14.07615504 |
| USH1C | 1.336008416 | 6.414540809 | 6.763668436 | 3.13E-11 | 9.41E-11 | 14.01808725 |
| TMEM176A | 1.11608558 | 8.63717712 | 6.751029056 | 3.40E-11 | 1.02E-10 | 13.93852902 |
| SLC6A19 | -2.093176697 | 3.915880428 | -6.730814677 | 3.87E-11 | 1.16E-10 | 13.81155688 |
| CD5L | 1.058163457 | 2.626677724 | 6.705525607 | 4.55E-11 | 1.35E-10 | 13.65317192 |
| STEAP4 | 1.020030641 | 4.050822069 | 6.690847309 | 4.99E-11 | 1.48E-10 | 13.56147822 |
| MAP7D2 | 1.485649226 | 3.778643962 | 6.653532565 | 6.33E-11 | 1.87E-10 | 13.32915961 |
| LTF | -1.78879414 | 3.430141719 | -6.612633211 | 8.20E-11 | 2.40E-10 | 13.07581616 |
| CBLN4 | 1.010425891 | 2.002413455 | 6.608582531 | 8.41E-11 | 2.46E-10 | 13.05079868 |
| CYP8B1 | -1.208866494 | 3.381248052 | -6.592538886 | 9.30E-11 | 2.71E-10 | 12.9518419 |
| REG1A | 2.157754453 | 4.396100189 | 6.586962635 | 9.64E-11 | 2.80E-10 | 12.91749662 |
| CRABP2 | -1.296460147 | 3.3591273 | -6.584006018 | 9.82E-11 | 2.85E-10 | 12.89929644 |
| SEMA3D | -1.057771515 | 2.231208551 | -6.567770717 | 1.09E-10 | 3.15E-10 | 12.79948258 |
| FBXL16 | 1.370933519 | 5.595635694 | 6.553265901 | 1.19E-10 | 3.43E-10 | 12.71048879 |
| AGT | 1.265940525 | 7.157459844 | 6.539312626 | 1.30E-10 | 3.74E-10 | 12.62504043 |
| HLA-DOA | 1.012500925 | 5.479150485 | 6.51823387 | 1.48E-10 | 4.25E-10 | 12.49625697 |
| C1QL4 | 1.441981492 | 2.782730172 | 6.487206638 | 1.80E-10 | 5.11E-10 | 12.30735095 |
| S100A9 | 1.019741952 | 6.519159911 | 6.478790163 | 1.89E-10 | 5.38E-10 | 12.25624362 |
| SLC22A12 | -1.966471934 | 3.779574858 | -6.47281919 | 1.97E-10 | 5.57E-10 | 12.2200212 |
| AGMAT | -1.316041274 | 5.730847814 | -6.454614623 | 2.20E-10 | 6.20E-10 | 12.10976457 |
| NEAT1 | 1.091621079 | 5.11744147 | 6.435165818 | 2.48E-10 | 6.98E-10 | 11.9922718 |
| B4GALNT1 | 1.058306045 | 2.513027602 | 6.426665743 | 2.61E-10 | 7.34E-10 | 11.94101903 |
| GXYLT2 | 1.006825847 | 3.171251726 | 6.421941244 | 2.69E-10 | 7.55E-10 | 11.9125574 |
| LRRN4 | -1.236969824 | 2.66559249 | -6.41718338 | 2.77E-10 | 7.76E-10 | 11.88391326 |
| ANXA13 | 1.311815115 | 4.63043282 | 6.392801234 | 3.22E-10 | 8.99E-10 | 11.73741502 |
| SLC17A3 | 2.02004662 | 6.205648972 | 6.376884997 | 3.55E-10 | 9.88E-10 | 11.64204691 |
| MALAT1 | 1.052561065 | 6.316821662 | 6.355965661 | 4.03E-10 | 1.12E-09 | 11.51701768 |
| MT1E | -1.334459763 | 6.060614415 | -6.349595238 | 4.19E-10 | 1.16E-09 | 11.47901492 |
| PHYHIPL | -1.130318753 | 4.325689301 | -6.304467871 | 5.52E-10 | 1.52E-09 | 11.21076529 |
| PKP3 | -1.057540876 | 1.424127542 | -6.259336341 | 7.25E-10 | 1.98E-09 | 10.94417293 |
| FCGBP | -1.020274082 | 2.605713334 | -6.257786335 | 7.32E-10 | 2.00E-09 | 10.93504696 |
| MMP7 | -1.724233009 | 6.849067418 | -6.24951668 | 7.69E-10 | 2.09E-09 | 10.88639136 |
| SPOCK1 | -1.390223563 | 5.179406064 | -6.247370045 | 7.79E-10 | 2.12E-09 | 10.87377061 |
| DES | -1.494264614 | 2.952999156 | -6.225094145 | 8.91E-10 | 2.41E-09 | 10.74302903 |
| ABO | -1.364149298 | 3.959948304 | -6.215290011 | 9.44E-10 | 2.55E-09 | 10.68561712 |
| CLEC18C | 1.303119689 | 2.733199001 | 6.198139024 | 1.05E-09 | 2.82E-09 | 10.58537482 |
| MT1X | -1.176381098 | 4.455112244 | -6.179801306 | 1.17E-09 | 3.13E-09 | 10.47846686 |
| PCDHB5 | -1.068281027 | 3.343547657 | -6.144828652 | 1.44E-09 | 3.83E-09 | 10.27535394 |
| GRIA4 | 1.183262396 | 2.736591761 | 6.133368035 | 1.54E-09 | 4.09E-09 | 10.20901512 |
| KLF15 | -1.020375455 | 4.404345897 | -6.101850215 | 1.85E-09 | 4.91E-09 | 10.02714213 |
| AQP1 | -1.131225646 | 8.564368202 | -6.08110282 | 2.10E-09 | 5.53E-09 | 9.907872753 |
| PRUNE2 | 1.039089788 | 6.205124209 | 6.080144994 | 2.11E-09 | 5.56E-09 | 9.902375251 |
| G0S2 | -1.08245857 | 6.107142991 | -6.0779101 | 2.13E-09 | 5.63E-09 | 9.889550919 |
| COL6A3 | 1.04593792 | 5.127936697 | 6.076446385 | 2.15E-09 | 5.67E-09 | 9.881154055 |
| MARCO | 1.003462548 | 3.665867585 | 6.04763037 | 2.55E-09 | 6.67E-09 | 9.716211156 |
| IGFBP5 | -1.059615835 | 8.981234864 | -6.027063094 | 2.88E-09 | 7.50E-09 | 9.598909799 |
| NR4A3 | -1.007313062 | 3.779378826 | -6.01814842 | 3.03E-09 | 7.88E-09 | 9.54817702 |
| CES1 | -1.014214243 | 3.296066085 | -6.017865969 | 3.03E-09 | 7.90E-09 | 9.546570702 |
| CDHR5 | 1.496094562 | 6.600114077 | 5.946994762 | 4.58E-09 | 1.18E-08 | 9.145642045 |
| GJB2 | 1.093144586 | 5.14020201 | 5.907133518 | 5.76E-09 | 1.47E-08 | 8.922001373 |
| CXCL5 | 1.346325079 | 2.323268658 | 5.897982897 | 6.07E-09 | 1.55E-08 | 8.870851443 |
| ALDH1L1 | -1.126250014 | 3.812506777 | -5.870258997 | 7.11E-09 | 1.80E-08 | 8.716313467 |
| ATF3 | -1.081506539 | 6.679789096 | -5.855347519 | 7.75E-09 | 1.96E-08 | 8.633463407 |
| PRIMA1 | 1.513244689 | 4.133472556 | 5.79760075 | 1.08E-08 | 2.69E-08 | 8.314394949 |
| CYP4A22 | -1.267493294 | 3.13893725 | -5.791485984 | 1.11E-08 | 2.78E-08 | 8.280774905 |
| ENPP2 | 1.103402292 | 6.429034407 | 5.770113094 | 1.26E-08 | 3.12E-08 | 8.163513029 |
| ALDH8A1 | -1.486631571 | 4.642333275 | -5.733268698 | 1.54E-08 | 3.80E-08 | 7.962280882 |
| FRZB | 1.132665876 | 5.730189389 | 5.721054331 | 1.65E-08 | 4.06E-08 | 7.895825582 |
| AOX1 | -1.238427172 | 5.82895768 | -5.702959907 | 1.83E-08 | 4.48E-08 | 7.797612591 |
| EPO | 1.47959218 | 2.186907929 | 5.693461398 | 1.93E-08 | 4.72E-08 | 7.746168565 |
| IGLON5 | 1.166721043 | 1.965804922 | 5.668378339 | 2.22E-08 | 5.39E-08 | 7.610689534 |
| MDK | 1.01526987 | 5.276490146 | 5.663554967 | 2.28E-08 | 5.54E-08 | 7.584699215 |
| SERPINA1 | 1.166957388 | 8.798967893 | 5.635211004 | 2.66E-08 | 6.45E-08 | 7.432373151 |
| SORCS3 | 1.276041848 | 3.374747882 | 5.623627033 | 2.84E-08 | 6.86E-08 | 7.37031699 |
| NPY6R | 1.110927239 | 3.5302705 | 5.610079297 | 3.06E-08 | 7.36E-08 | 7.297886972 |
| MAPT | 1.095144589 | 4.155895616 | 5.587443758 | 3.46E-08 | 8.31E-08 | 7.17722294 |
| CADM3 | 1.020927708 | 2.718679864 | 5.552628559 | 4.19E-08 | 1.00E-07 | 6.992492964 |
| PRAME | 1.193201422 | 3.480323085 | 5.432617679 | 8.01E-08 | 1.87E-07 | 6.363733144 |
| ISLR | -1.128651227 | 4.953307853 | -5.342350683 | 1.29E-07 | 2.97E-07 | 5.899035074 |
| NR4A1 | -1.09700797 | 5.126808554 | -5.341597299 | 1.30E-07 | 2.98E-07 | 5.895186456 |
| CXCL2 | 1.015776547 | 3.714632969 | 5.317592257 | 1.47E-07 | 3.37E-07 | 5.772817235 |
| MT3 | 1.438512374 | 3.508802575 | 5.308331558 | 1.55E-07 | 3.53E-07 | 5.725743833 |
| BASP1P1 | 1.045852307 | 2.593302307 | 5.268639643 | 1.90E-07 | 4.31E-07 | 5.524832972 |
| RBP4 | -1.784289856 | 5.05366825 | -5.247803334 | 2.12E-07 | 4.79E-07 | 5.419916061 |
| IGLV1-44 | 1.395857312 | 4.38853843 | 5.10323857 | 4.45E-07 | 9.81E-07 | 4.702481256 |
| SERPINF2 | 1.074614591 | 7.601651793 | 5.018815915 | 6.81E-07 | 1.48E-06 | 4.292032043 |
| LGALS2 | 1.075028632 | 7.312654239 | 4.893536068 | 1.27E-06 | 2.69E-06 | 3.694583173 |
| CYP4A11 | -1.584438365 | 4.955695338 | -4.884589502 | 1.32E-06 | 2.80E-06 | 3.652451752 |
| AZGP1 | -1.129828497 | 5.071260408 | -4.878997978 | 1.36E-06 | 2.88E-06 | 3.626156218 |
| SERPINA6 | -1.047383461 | 4.035084737 | -4.767669087 | 2.33E-06 | 4.83E-06 | 3.108416654 |
| FGG | 1.5519412 | 3.03626739 | 4.622304723 | 4.63E-06 | 9.34E-06 | 2.449122987 |
| SLC5A1 | 1.203080393 | 5.038504799 | 4.560257127 | 6.17E-06 | 1.23E-05 | 2.173506585 |
| MIOX | -1.32659808 | 5.614925924 | -4.557824443 | 6.24E-06 | 1.25E-05 | 2.162771452 |
| SLC13A1 | -1.187151402 | 3.324964572 | -4.522576288 | 7.33E-06 | 1.45E-05 | 2.007826807 |
| KRT19 | -1.373415806 | 5.561523493 | -4.417681062 | 1.18E-05 | 2.30E-05 | 1.553392822 |
| CCL21 | -1.163481826 | 4.76857192 | -4.135518069 | 4.03E-05 | 7.49E-05 | 0.380815876 |
| SLC34A2 | -1.121477529 | 3.452483489 | -3.822353617 | 0.000145616 | 0.000256617 | -0.834717597 |
| LGALS4 | 1.057263317 | 2.790565208 | 3.709435684 | 0.000226497 | 0.000392069 | -1.250637655 |
| SAA1 | 1.262547996 | 3.592645973 | 3.336408846 | 0.000899752 | 0.001461412 | -2.539553008 |
