# Supplementary material for: Exploring necrosis-associated mitochondrial gene signatures: revealing their role in prognosis and immunotherapy of renal clear cell carcinoma
Source: Clin Exp Med. 2024 Jul 18;24(1):161. doi: 10.1007/s10238-024-01426-9 (PMC11258092; doi:10.1007/s10238-024-01426-9)
Supplement: Supplementary file 7 — Supplementary file7 (DOCX 14 KB) [file 10238_2024_1426_MOESM7_ESM.docx]

| BID |  | 0.441247513 |
| --- | --- | --- |
| FKBP10 |  | 0.19482759 |
| PRELID2 |  | -0.385769052 |
| FDXR |  | -0.341766632 |
| POLG2 |  | 0.450530994 |
| ACADM |  | -0.365727422 |

**Supplementary Table S1**
